# Enantioselective construction of six- and seven-membered triorgano-substituted silicon-stereogenic heterocycles

**DOI:** 10.1038/s41467-021-21489-6

**Published:** 2021-02-23

**Authors:** Shuyou Chen, Delong Mu, Pei-Lin Mai, Jie Ke, Yingzi Li, Chuan He

**Affiliations:** Shenzhen Grubbs Institute and Department of Chemistry, Guangdong Provincial Key Laboratory of Catalysis, Southern University of Science and Technology, Shenzhen, Guangdong China

**Keywords:** Asymmetric catalysis, Homogeneous catalysis, Stereochemistry, Synthetic chemistry methodology

## Abstract

The exploitation of chirality at silicon in asymmetric catalysis is one of the most intriguing and challenging tasks in synthetic chemistry. In particular, construction of enantioenriched mediem-sized silicon-stereogenic heterocycles is highly attractive, given the increasing demand for the synthesis of novel functional-materials-oriented silicon-bridged compounds. Here, we report a rhodium-catalyzed enantioselective construction of six- and seven-membered triorgano-substituted silicon-stereogenic heterocycles. This process undergoes a direct dehydrogenative C−H silylation, giving access to a wide range of triorgano-substituted silicon-stereogenic heterocycles in good to excellent yields and enantioselectivities, that significantly enlarge the chemical space of the silicon-centered chiral molecules. Further elaboration of the chiral monohydrosilane product delivers various corresponding tetraorgano-substituted silicon-stereogenic heterocycles without the loss of enantiopurity. These silicon-bridged heterocycles exhibit bright blue fluorescence, which would have potential application prospects in organic optoelectronic materials.

## Abstract (web summary)

Enantioenriched medium-sized silicon-stereogenic heterocycles are in high demand due to the potential use in functional materials. Here, the authors show a rhodium-catalyzed enantioselective synthesis of six- and seven-membered tri-organosubstituted silicon-stereogenic heterocycles.

## Introduction

**Fig. 1:**
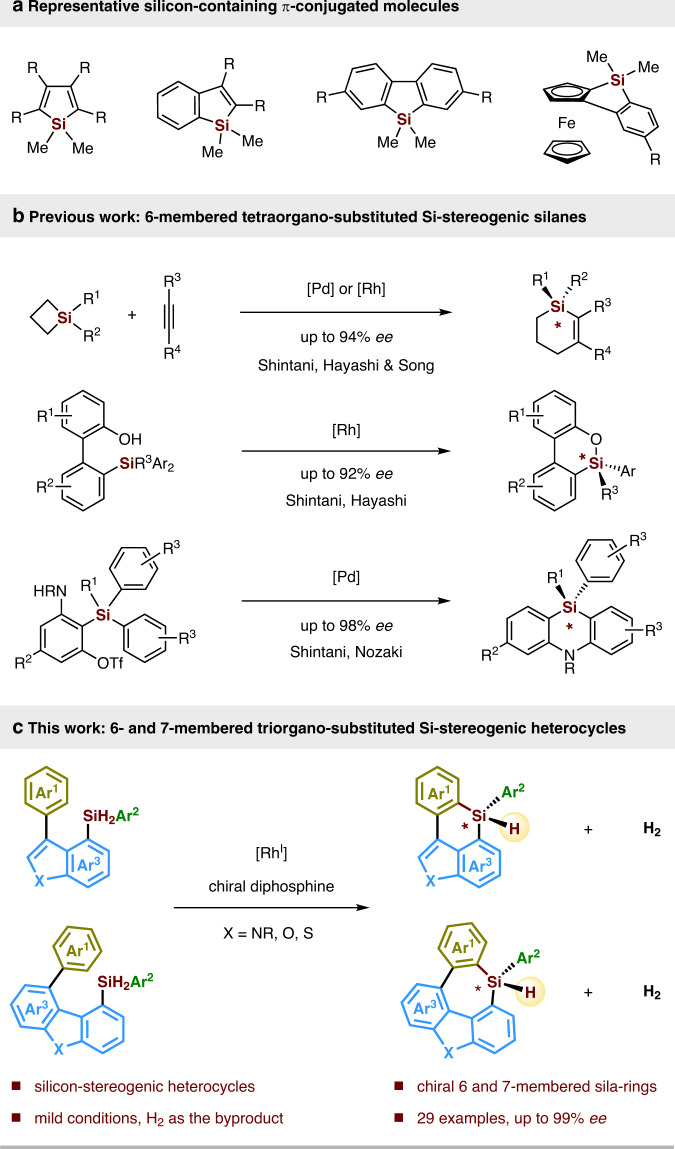
Construction of silicon-containing π-conjugated molecules. **a** Representative silicon-containing π-conjugated molecules. **b** Asymmetric synthesis of six-membered tetraorgano-substituted Si-stereogenic silanes (previous work). **c** Enantioselective construction of six- and seven-membered triorgano-substituted Si-stereogenic heterocycles (this work).

Silicon-containing π-conjugated molecules are an emerging class of optoelectronic materials, that have been widely used as key components in organic light-emitting materials, thin-film transistors, fluorescent sensors, solar cells, electroluminescent devices, and so on^1–8^. Incorporation of a silylene bridge across a π-conjugated framework lowers the LUMO of the parent conjugated system via σ*–π* conjugation, that results the unique optical and electronic properties of these silicon-containing π-conjugated molecules^9–13^. In light of this fact, development of new synthetic methods for the construction of these silylene-bridged compounds with broad structural variations is of great importance for advanced modification and exploitation of functional organic materials exhibiting unique properties. During the past decades, many endeavours have been made, delivering a variety of five-membered silicon-bridged biaryls such as siloles, benzosiloles, 9-silafluorenes, benzosilolometallocenes, and so on (Fig. 1a)^6,13–30^. Among those elegant silicon-bridged biaryls, only limited examples possess six- or seven-membered silicon-bridged π-conjugated scaffolds^31–38^, and even less examples display silicon-stereogenic centers. To the best of our knowledge, only Shintani, Hayashi, Nozaki et al. and Song have succeeded in forming six-membered tetraorgano-substituted silicon-stereogenic silanes (Fig. 1b)^39–43^, while the construction of Si-chirality on six- and seven-membered triorgano-substituted monohydrosilanes via asymmetric catalysis is still unknown to date, which is presumably due to a lack of general and efficient synthetic methods. Given the increasing demand for the synthesis of novel functional-materials-oriented silicon-bridged biaryls, construction of six- or seven-membered monohydrosilanes in a chiral version is highly attractive. These interesting chiral monohydrosilane heterocycles may find various synthetic applications in stereospecific transformations^44–49^, which could lead to their potential applications in new chiral organic materials.

Historically, enantioselective synthesis of silicon-stereogenic silanes is one of the most challenging tasks in synthetic chemistry^47,50–53^. The preparation of silicon-stereogenic silanes in enantiopure or enantioenriched form is mainly restricted to the resolution with chiral auxiliary^47,50,51^. Encouragingly, in the past two decades, a number of research efforts have been dedicated to the design and development of chiral transition-metal catalysts, that can perform catalytic asymmetric transformation of prochiral dihydrosilanes or tetraorganosilanes to enantioenriched silicon-stereogenic silanes^54–56^. However, the limited silane substrate scope and relatively poor enantiocontrol still need to be expanded and improved. Therefore, the exploration and development of efficient catalytic methods that enable the construction of new scaffold silicon-stereogenic silanes with high enantioselectivities is highly warranted.

With the continued interest in silicon-stereogenic chemistry, we questioned whether we could utilize asymmetric dehydrogenative C–H silylation toolbox^57,58^ for the construction of six- or seven-membered silicon-stereogenic silanes. To achieve this target, three major obstacles are expected: (1) the intramolecular C–H silylation must proceed through a disfavoured seven- or eight-membered-ring cyclometallated intermediate, which would be rather challenging; (2) the competing dehydrogenative homo-coupling of dihydrosilanes is plausible^59,60^; (3) the control of high enantioselectivity is elusive. Herein, we report the exploration and realization of a rhodium-catalyzed enantioselective construction of six- and seven-membered silicon-stereogenic silanes by dehydrogenative C–H silylation, which give access to a variety of highly functionalized triorgano-substituted silicon-stereogenic heterocycles (Fig. 1c).

## Results and discussion

### Investigations of reaction conditions

**Fig. 2:**
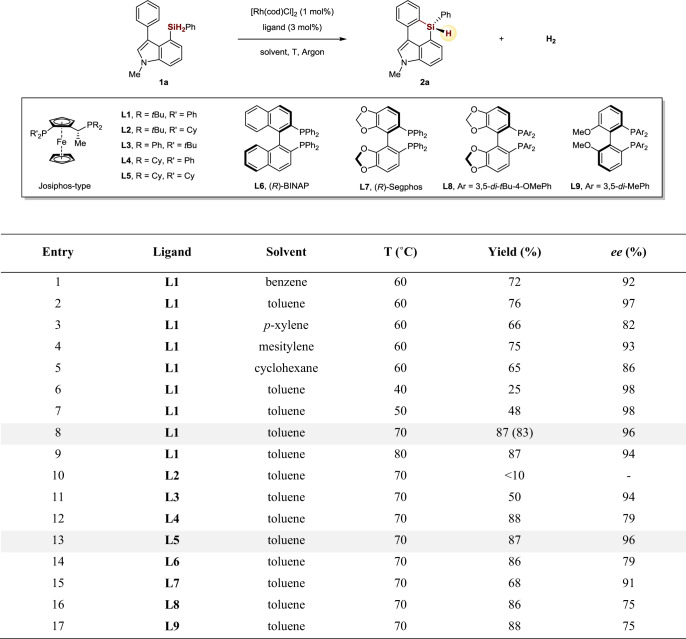
Development of reaction conditions. Conditions: **1a** (0.1 mmol), [Rh(cod)Cl]_2_ (1 mol%), ligand (3 mol%), in 1.0 mL solvent under Argon atmosphere. The yield was determined by ^1^H NMR using CH_2_Br_2_ as internal standard, yield in brackets is isolated yield. The *ee* values were determined by chiral HPLC.

We commenced our studies of the enantioselective C–H silylation using an indole based dihydrosilane substrate **1a**, which could presumably undergo C–H silylation of the C3 aryl group via a seven-membered metallocycle intermediate. The reason for choosing indole skeleton in the research is because silicon-bridged biaryls consisting of arenes and indole rings were found to exhibit intense blue fluorescence and high quantum yields, which are highly attractive as key components to be used in optoelectronic devices^61^. To our delight, we found that treatment of **1a** with [Rh(cod)Cl]_2_ (1 mol%) as the catalyst and Josiphos **L1** (3 mol%) as the chiral ligand in various nonpolar solvents at 60 °C successfully delivered the desired C–H silylation/cyclization six-membered silicon-stereogenic products in decent yields and enantioselectivities (Fig. 2, entries 1–5). The effect of the temperature on the reaction is then investigated. The reaction at 40 or 50 °C showed excellent enantioselectivity (98% *ee*), but gave a lower yield (Fig. 2, entries 6–7). Increasing the temperature to 70 °C improved the yield to 87% with 96% *ee*. When the reaction was carried out at 80 °C, a slightly lower enantioselectivity (94% *ee*) was observed (Fig. 2, entries 8–9). Further examination of the chiral ligands showed that, besides Josiphos type ligand (**L1**, **L3**, **L4**, and **L5**), other type of diphosphine ligands such as BINAP (**L6**), Segphos (**L7** and **L8**) and MeOBIPHEP (**L9**) were also effective for this transformation, albeit in a relatively lower *ee* (Fig. 2, entries 10–17).

### Substrate scope of six-membered triorgano-substituted silicon-stereogenic heterocycles

**Fig. 3:**
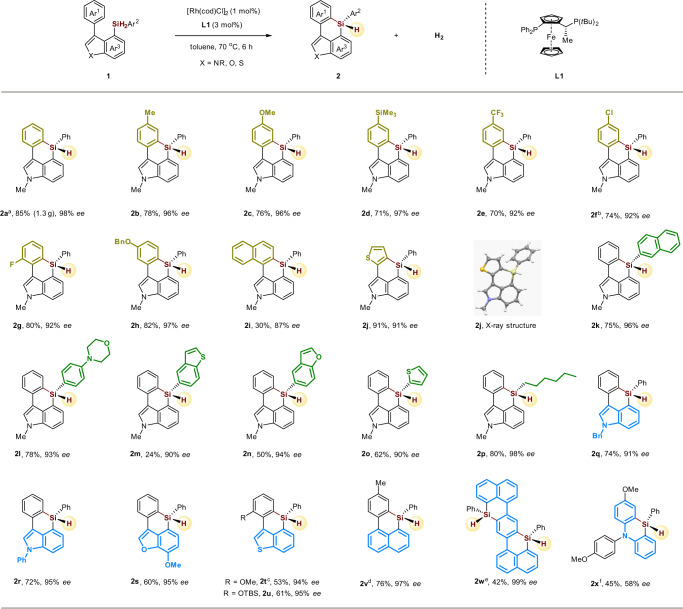
Scope of six-membered triorgano-substituted silicon-stereogenic heterocycles. Reaction conditions: **1** (0.1 mmol), [Rh(cod)Cl]_2_ (1 mol%), **L1** (3 mol%) in toluene (1 mL) at 70 °C for 6 h. **a** Reaction was performed at 5 mmol scale. **b L5** was used as ligand. **c** [Rh(cod)Cl]_2_ (4 mol%), **L1** (8 mol%) was used. **d** [Rh(cod)Cl]_2_ (2 mol%), **L1** (4 mol%) in toluene (1 mL) at 60 °C for 8 h. **e** [Rh(cod)Cl]_2_ (2 mol%), **L1** (6 mol%) was used. **f L7** was used as ligand. X-ray crystallographic analysis of **2j** allowed to determine the absolute configuration; and configurations of the products **2** were assigned by analogy.

With the optimized conditions in hand, we next examined the scope for this rhodium-catalyzed asymmetric dehydrogenative C–H silylation for the construction of six-membered triorgano-substituted silicon-stereogenic heterocycles (Fig. 3). First, the reaction of model substrate **1a** proceeded smoothly in a gram scale (85% yield, 1.3 g) with an even better enantioselectivity (**2a**). The reacting aromatic rings (Ar^1^, yellow part) bearing electron-donating methyl (**2b**), methoxy (**2c**), trimethylsilyl (**2d**), benzyloxy (**2h**) groups, and electron-withdrawing trifluoromethyl (**2e**), chloro (**2f**), fluoro (**2g**) groups, as well as naphthyl (**2i**) and thiophenyl (**2j**) groups in different positions, were all well tolerated in the transformation, affording the corresponding asymmetrically triorgano-substituted silicon-stereogenic indole based silanes in good to excellent yields and enantioselectivities. Then, the silyl group tethered aromatic rings (Ar^2^, green part) were also investigated under the standard conditions. Naphthalene, morpholine, benzothiophene, benzofuran, thiophene, and alkyl chain (**2k–2p**) were all compatible functionalities in the process, giving the desired products without the loss of enantioselectivities. For the indole based hetero-aromatic rings (Ar^3^, blue part), we found that benzyl (**2q**) and phenyl (**2r**) substituted indoles were competent substrates in the reaction. Besides indole skeleton, benzofuran (**2s**), benzothiophene (**2t, 2u**), and naphthalene (**2v, 2w**) were also well accommodated in this transformation to deliver the interesting six-membered triorgano-substituted silicon-stereogenic heterocycles in moderate to good yields with excellent enantioselectivities. It is noteworthy that the enantiopure six-membered bis-silicon-stereogenic heterocycles (**2w**) is easily constructed via this strategy, which could be highly attractive to organic material chemists, given that silicon-bridged ladder π-conjugated systems are an important class of useful skeletons in material sciences^11–13^. Finally, we found that the silicon-stereogenic phenazasiline (silicon-bridged diphenylamine) derivatives (**2x**), that usually exhibiting unique optical and electronic properties^62,63^, could also be synthesized by this rhodium-catalyzed dehydrogenative C–H silylation, albeit in a relatively lower yield and *ee*.

### Substrate scope of seven-membered triorgano-substituted silicon-stereogenic heterocycles

**Fig. 4:**
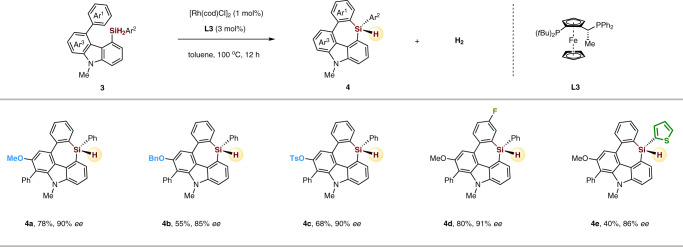
Scope of seven-membered triorgano-substituted silicon-stereogenic heterocycles. Reaction conditions: **3** (0.1 mmol), [Rh(cod)Cl]_2_ (1 mol%), **L3** (3 mol%) in toluene (1 mL) at 100 °C for 12 h.

Moreover, the scope for this rhodium-catalyzed enantioselective C–H silylation for the construction of seven-membered triorgano-substituted silicon-stereogenic heterocycles is explored. On the basis of the established reaction conditions, simply changing Josiphos ligand from **L1** to **L3** (see Supplementary Table 1 for details), several seven-membered carbazole-based silicon-stereogenic heterocycles displaying a variety of substituents were obtained in good yields and enantioselectivities (Fig. 4). Methoxy, OBn, OTs, phenyl, fluoro, and thiophenyl functional groups were all well tolerated in the transformation (**4a**–**4e**). These results demonstrate the facile enantioselective construction of silicon-stereogenic centers in medium-sized rings.

### Synthetic applications

**Fig. 5:**
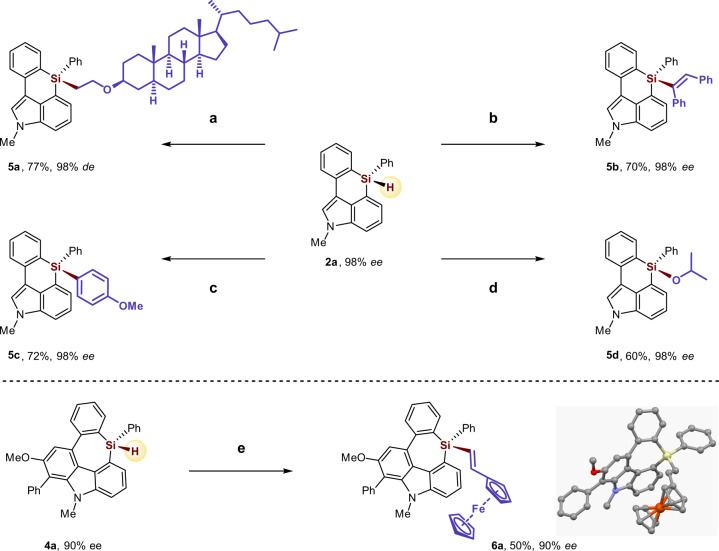
Derivatization of the Si-stereogenic heterocycles. Reaction conditions: **a** [Rh(cod)Cl]_2_ (2 mol%), (*racemic*)-Josiphos **L1** (4 mol%), vinyl ether (2.0 equiv), toluene, 60 °C, 12 h, *de* = diastereomic excess. **b** Pt(dvds) (10 µL, 0.1 M in xylene), 1,2-diphenylacetylene (2.0 equiv), toluene, 40 °C, 12 h. **c** Pd(*t*Bu_3_P)_2_ (2 mol%), 4-iodoanisole (2.0 equiv), K_3_PO_4_ (3.0 equiv), NMP, rt, 12 h. **d** [Rh(cod)Cl]_2_ (2 mol%), (*racemic*)-Josiphos **L1** (4 mol%), acetone (2.0 equiv), toluene, 60 °C, 12 h. **e** [Rh(cod)Cl]_2_ (2 mol%), (*racemic*)-Josiphos **L1** (4 mol%), vinyl ferrocene (2.0 equiv), toluene, 60 °C, 12 h. X-ray crystallographic analysis of **6a** allowed to determine the absolute configuration; and configurations of the products **4** were assigned by analogy.

To further probe the synthetic utility of the enantioenriched six- and seven-membered silicon-stereogenic heterocycle products, several elaborate stereospecific transformations were performed (Fig. 5). Hydrosilylation of vinyl ether, or internal alkyne, or acetone with the enantioenriched six-membered monohydrosilane compound **2a** in the presence of the Rh/(racemic)-Josiphos **L1** catalyst or Pt(dvds) (dvds = 1,1,3,3-tetramethyl-1,3-divinyldisiloxane) catalyst gave the corresponding tetraorgano-substituted functionalized silane product **5a**, **5b**, **5d**, respectively, without the loss of enantiopurity. Coupling of **2a** with 4-iodoanisole in the presence of the Pd(*t*Bu_3_P)_2_ catalyst afforded the arylation silane product **5c** in 72% yield and excellent enantiomeric purity. Moreover, treatment of the enantioenriched seven-membered monohydrosilane compound **4a** (90% *ee*) with 2 equivalent vinyl ferrocene produced the oxidative alkenylation product **6a** in 50% yield and 90% *ee*.

### Photophysical properties investigations

**Fig. 6:**
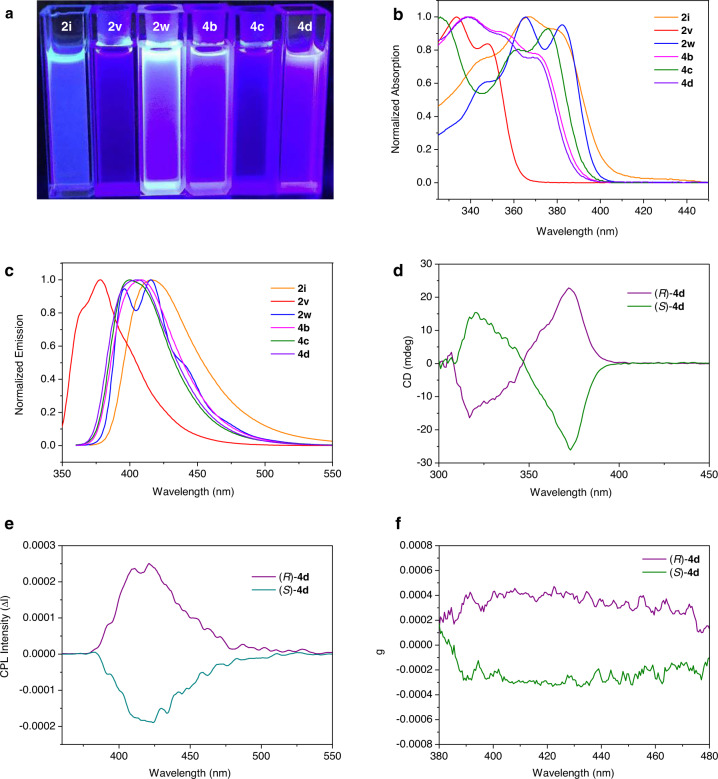
Photophysical properties investigations. **a** Fluorescence images of selected silicon-bridged heterocycles (λex = 365 nm). **b** Absorption spectra of selected silicon-bridged heterocycles in CHCl_3_ (10^−5^ M). **c** Emission spectra of selected silicon-bridged heterocycles in CHCl_3_ (10^−5^ M). **d** CD (circular dichroism) spectra of compound (*R*)-**4d** (purple line, 91% *ee*) and (*S*)-**4d** (green line, 88% *ee*) in CHCl_3_ (2.0 × 10^−4^ M) at room temperature. **e** CPL (circular polarized luminescence) spectra of compound (*R*)-**4d** (purple line, 91% *ee*) and (*S*)-**4d** (green line, 88% *ee*) in CHCl_3_ (2.0 × 10^−3^ M) at room temperature, excited at 339 nm. **f** *g*_lum_ (Luminescence dissymmetry factor) values-wavelength curve for (*R*)-**4d** (purple line, 91% *ee*) and (*S*)-**4d** (green line, 88% *ee*).

Having established the asymmetric construction of various six- and seven-membered chiral silicon-bridged heterocycle compounds, we finally examined the photophysical properties of selected products (Fig. 6). Apparently, most of these compounds display bright blue fluorescence under UV light irradiation (365 nm) (Fig. 6a). The absorption and emission maxima of these compounds alter from 333 to 382 nm, and 378 to 417 nm, respectively (Fig. 6b, c) (see Supplementary Table 2 for details). A remarkable red-shift was observed from **2v** to **2w** for both the absorption and emission maxima, due to the extended π-conjugated system. The seven-membered silicon-bridged heterocycle with electron-donating OBn group exhibits blue-shift than the one with electron-withdrawing OTs group in absorption maxima (**4b** vs **4c**). Then, the chiroptical properties of the seven-membered silicon-bridged heterocycles (*R*)-**4d** and (*S*)-**4d** were investigated based on circular dichroism (CD) and circular polarized luminescence (CPL) spectroscopies. The CD spectra of (*R*)-**4d** and (*S*)-**4d** was a mirror image and displayed clear Cotton effects at around 317 and 372 nm, respectively (Fig. 6d). The CPL maxima of (*R*)-**4d** and (*S*)-**4d** in CHCl_3_ solution were at 423 and 419 nm with the corresponding dissymmetry factors at emission maxima (*g*_lum_) being 4.7 × 10^−4^ and −3.2 × 10^−4^, respectively (Fig. 6e, f).

In summary, we have developed a rhodium-catalyzed enantioselective dehydrogenative C–H silylation methodology, which enables the construction of a wide range of six- and seven-membered triorgano-substituted silicon-stereogenic heterocycles in good to excellent yields and enantioselectivities. Most of these silicon-bridged heterocycles exhibit bright blue fluorescence. We believe that these previously inaccessible asymmetrically silicon-stereogenic heterocycles will find widespread use among synthetic chemistry and optoelectronic materials science.

## Methods

### General Procedure for the enantioselective construction of six-membered silicon-stereogenic heterocycle 2a

A 5 mL microwave tube was charged with **1a** (0.1 mmol, 31.3 mg), [Rh(cod)Cl]_2_ (0.5 mg, 1 mol%), **L1** (1.6 mg, 3 mol%), and toluene (1 mL) in glovebox. The tube was sealed, then removed from the glovebox, and the mixture was stirred at 70 °C for 6 h. After the completion of the reaction, the solvent was evaporated under reduced pressure and the residue was purified by flash column chromatography on a silica gel to give **2a** as a white solid (25.9 mg, 83% yield).

## Supplementary information

Supplementary Information

## Data Availability

The data that support the findings of this study are available within the paper and its supplementary information files. Raw data are available from the corresponding author on reasonable request. Materials and methods, experimental procedures, characterization data, ^1^H, ^13^C, ^19^F NMR spectra and mass spectrometry data are available in the Supplementary Information. The X-ray crystallographic coordinates for structures reported in this study have been deposited at the Cambridge Crystallographic Data Centre (CCDC), under deposition numbers CCDC 2031952 (**2j**), and CCDC 2031700 (**6a**). These data can be obtained free of charge from The Cambridge Crystallographic Data Centre via www.ccdc.cam.ac.uk/data_request/cif.
