# Supplementary material for: Enantioselective construction of six- and seven-membered triorgano-substituted silicon-stereogenic heterocycles
Source: Nat Commun. 2021 Feb 23;12:1249. doi: 10.1038/s41467-021-21489-6 (PMC7902825; doi:10.1038/s41467-021-21489-6)
Supplement: Supplementary file 1 — Supplementary Information [file 41467_2021_21489_MOESM1_ESM.pdf]

Supplementary Information

Enantioselective construction of six- and seven-membered triorgano-substituted silicon-stereogenic heterocycles

Shuyou Chen,† Delong Mu,† Pei-Lin Mai, Jie Ke, Yingzi Li, and Chuan He*

Shenzhen Grubbs Institute and Department of Chemistry, Guangdong Provincial Key Laboratory of Catalysis, Southern University of Science and Technology, Shenzhen, Guangdong 518055, China

hec@sustech.edu.cn

I. General Information....................2

II. Preparation of Dihydrosilane Substrates....................3

III. Optimization for 7-Membered Si-Stereogenic Heterocycles....................20

IV. Enantioselective Construction of Si-Stereogenic Heterocycles....................22

V. Gram-Scale Reaction....................39

VI. Photophysical Data of Selected Si-Stereogenic Heterocycles....................40

VII. Derivatization of Si-Stereogenic Heterocycles....................41

VIII. Single Crystal X-Ray Diffraction....................45

IX. NMR Spectra....................49

X. Chiral HPLC Spectra....................118

XI. Supplementary References....................153

I. General Information

Regular reactions were carried out under argon atmosphere with magnetic stirring. Catalysis reactions were performed in colorless 5 mL microwave reaction tube under an inert atmosphere of argon. Anhydrous solvents were obtained from Inert Pure Solv solvent purification system or purchased from Energy Chemical. TLC were performed on silica gel Huanghai HSGF254 plates and visualization of the developed chromatogram was performed by fluorescence quenching (λ_{max} = 254 nm). Column chromatography was performed using GENERAL-REAGENT silica gel (200-300 mesh). Unless otherwise noted, all reagents were obtained from commercial suppliers (Bide Pharmatech, Aladdin, Energy Chemical, Adamas, and TCI) and used without further purification.

NMR spectra were obtained on Bruker DPX 400 (400 MHz) or Bruker DPX 600 (600 MHz) instruments. The ^{1}H NMR (400 or 600 MHz) chemical shifts were referenced to tetramethylsilane signal (TMS: δ 0 ppm). The ^{13}C NMR (100 or 150 MHz) chemical shifts were given using $CDCl_3$ as the internal standard ($CDCl_3$: δ 77.0 ppm). High resolution mass spectra (HRMS) were obtained with an Agilent Technologies 6230 TOF LC/MS or Waters Premier GC-TOF MS by electrospray ionization (ESI) or electron impact ionization (EI). X-Ray single-crystal diffraction data were collected on Bruker D8 VENTURE. Absorption spectra were measured with a UV-Vis spectrophotometer (Shimadzu, UV3600). Emission spectra were measured with a Shimadzu RF-6000 spectrometer. Circular polarized luminescence (CPL) spectra were measured on a JASCO CPL-300 spectrometer. Circular dichroism (CD) spectra were measured on an APPLIED PHOTOPHYSICS Chirascan CD Spectrometer. Chiral HPLC chromatograms were obtained from an Agilent 1260 system. Optical rotation was measured on Rudolph Automatic Polarmeter at concentrations of 0.1 g/100 mL in $CHCl_3$.

II. Preparation of Dihydrosilane Substrates

1. Indole Dihydrosilane Substrates[1,2,3]

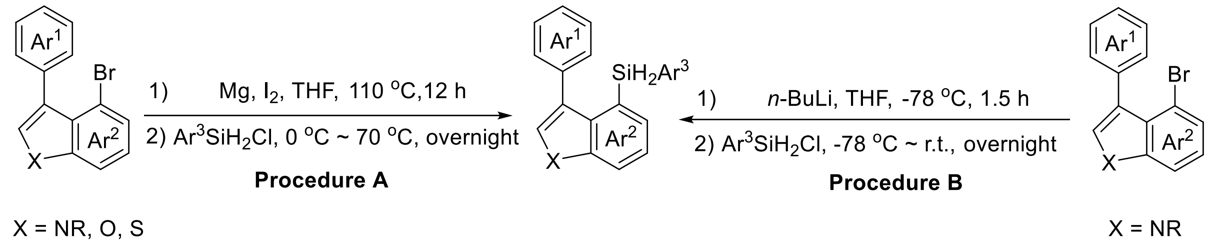

Procedure A: Under argon atmosphere, a 50 mL Schlenk tube was charged with brominated heterocycle[4,5,6] (1 equiv), Mg powder (5 equiv), I_2 (2 granules), and THF (0.5 M). The tube was then sealed and the mixture was stirred at 110 °C for 12 h. The Grignard reagent was used for next step without further purification.

Under argon atmosphere, Ar^3SiH_2Cl (1.5 eq) was added to a well stirred solution of Grignard reagent slowly at 0 °C. Then the reaction mixture was stirred at 70 °C overnight. After cooling the solution to room temperature, saturated NH_4Cl was added. The mixture was extracted with EtOAc and the combined organic layer was dried over anhydrous Na_2SO_4, filtered, concentrated under reduced pressure and purified by silica gel flash column chromatography to provide the desired compound.

Procedure B: Under argon atmosphere, a 50 mL Schlenk tube was charged with brominated heterocycle[4] (1 equiv) and THF (0.5 M). The mixture was cooled down to -78 °C and *n*-BuLi (1.1 equiv) was added slowly. After 1.5 h, Ar^3SiH_2Cl (1.5 equiv) was added dropwise. Then the reaction mixture was allowed to warm to room temperature and stirred overnight. After cooling the solution to room temperature, saturated NH_4Cl was added. The mixture was extracted with EtOAc and the combined organic layer was dried over anhydrous Na_2SO_4, filtered, concentrated under reduced pressure and purified by silica gel flash column chromatography to provide the desired compound.

2. Carbazole Dihydrosilane Substrates[1,7]

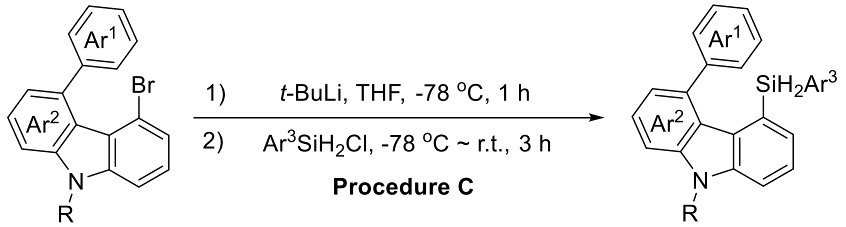

Procedure C: Under argon atmosphere, a 50 mL Schlenk tube was charged with brominated heterocycle[8] (1 equiv) and THF (0.1 M). The mixture was cooled down to -78 °C and *t*-BuLi (2.2 equiv) was added slowly. After 1 h, Ar^3SiH_2Cl (1.5 equiv) was added dropwise. Then the reaction mixture was allowed to warm to room temperature and stirred for additional 3 h. Then, saturated NH_4Cl was added. The mixture was extracted with EtOAc and the combined organic layer was dried over anhydrous Na_2SO_4, filtered, concentrated under reduced pressure and purified by silica gel flash column chromatography to provide the desired compound.

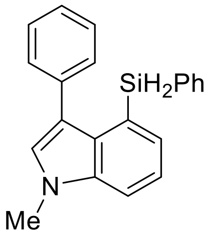

1-Methyl-3-phenyl-4-(phenylsilyl)-1*H*-indole (1a)

The reaction was performed at 3 mmol scale according to procedure A. Purification via silica gel column chromatography (petroleum ether/ethyl acetate = 40/1, v/v) afforded the product **1a** as a white solid (526 mg, 56% yield). ^{1}H NMR (400 MHz, $CDCl_3$): δ = 7.47 (dd, J = 8.2, 0.9 Hz, 1H), 7.36 (dd, J = 6.9, 1.0 Hz, 1H), 7.32 – 7.25 (m, 6H), 7.25 – 7.18 (m, 5H), 7.04 (s, 1H), 4.64 (s, 2H), 3.84 (s, 3H) ppm. ^{13}C NMR (100 MHz, $CDCl_3$): δ = 136.0, 135.8, 135.5, 132.7, 131.7, 131.0, 131.0, 129.1, 128.1, 127. 8, 127. 6, 126.7, 123.0, 121.2, 119.4, 111.7, 32.8 ppm. HRMS (ESI): calcd for $C_{21}H_{20}NSi$ $[M+H]^+$ 314.1360, found 314.1357.

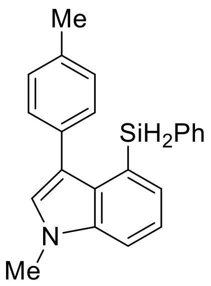

1-Methyl-4-(phenylsilyl)-3-(*p*-tolyl)-1*H*-indole (1b)

The reaction was performed at 3.7 mmol scale according to procedure A. Purification via silica gel column chromatography (petroleum ether/ethyl acetate = 40/1, v/v) afforded the product **1b** as a white solid (556 mg, 46% yield).^{1}H NMR (400 MHz, $CDCl_3$): δ = 7.45 (d, J = 8.0 Hz, 1H), 7.34 (d, J = 6.9 Hz, 1H), 7.31 – 7.17 (m, 6H), 7.15 (d, J = 7.9 Hz, 2H), 7.04 (d, J = 7.8 Hz, 2H), 7.01 (s, 1H), 4.65 (s, 2H), 3.82 (s, 3H), 2.38 (s, 3H) ppm. ^{13}C NMR (100 MHz, $CDCl_3$): δ = 136.4, 135.8, 135.5, 132.9, 132. 8, 131.8, 130.9, 130.8, 129.1, 128.5, 128.1, 127.5, 123.0, 121.1, 119.3, 111.6, 32.7, 21.2 ppm. HRMS (ESI): calcd for $C_{22}H_{22}NSi$ [M+H]$^+$ 328.1516, found 328.1513.

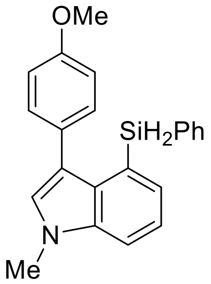

3-(4-Methoxyphenyl)-1-methyl-4-(phenylsilyl)-1*H*-indole (1c)

The reaction was performed at 3.6 mmol scale according to procedure A. Purification via silica gel column chromatography (petroleum ether/ethyl acetate = 80/1, v/v) afforded the product **1c** as a white solid (501 mg, 40% yield).^{1}H NMR (400 MHz, $CDCl_3$): δ = 7.46 (d, J = 8.2 Hz, 1H), 7.39 – 7.32 (m, 1H), 7.32 – 7.25 (m, 3H), 7.25 – 7.18 (m, 3H), 7.15 (dd, J = 8.5, 2.0 Hz, 2H), 7.00 (s, 1H), 6.77 (dd, J = 8.5, 1.9 Hz, 2H), 4.64 (d, J = 2.6 Hz, 2H), 3.88 – 3.80 (m, 6H) ppm. ^{13}C NMR (100 MHz, $CDCl_3$): δ = 158.8, 135.7, 135.4, 132.8, 132.1, 132.0, 130.8, 129.1, 128.1, 128.1 127.5, 122.9, 121.1, 118.9, 113.2, 111.6, 55.3, 32.7 ppm. HRMS (ESI): calcd for $C_{22}H_{22}NOSi$ [M+H]$^+$ 344.1465, found 344.1462.

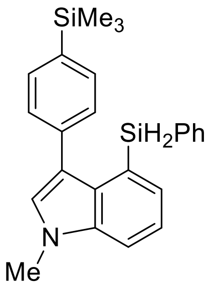

1-Methyl-4-(phenylsilyl)-3-(4-(trimethylsilyl)phenyl)-1*H*-indole (1d)

The reaction was performed at 1.7 mmol scale according to procedure A. Purification via silica gel column chromatography (petroleum ether/ethyl acetate = 40/1, v/v) afforded the product **1d** as a pale yellow oil (332 mg, 50% yield).^{1}H NMR (400 MHz, $CDCl_3$): δ = 7.47 (d, J = 8.2 Hz, 1H), 7.41 (dd, J = 6.9, 0.9 Hz, 1H), 7.36 (d, J = 7.9 Hz, 2H), 7.30 – 7.25 (m, 2H), 7.21 (d, J = 7.9 Hz, 2H), 7.19 – 7.11 (m, 4H), 7.02 (s, 1H), 4.68 (s, 2H), 3.83 (s, 3H), 0.31 (s, 9H) ppm. ^{13}C NMR (100 MHz, $CDCl_3$): δ = 138.2, 136.3, 135.9, 135.3, 132.8, 132.8, 131.6, 131.2, 130.2, 129.0, 128.1, 127.5, 123.0, 121.2, 119.5, 111.7, 32.8, -1.0 ppm. HRMS (ESI): calcd for $C_{24}H_{28}NSi_2$ $[M+H]^+$ 386.1755, found 386.1749.

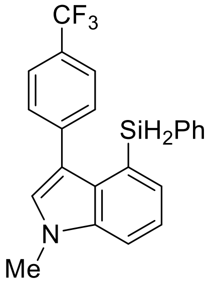

1-Methyl-4-(phenylsilyl)-3-(4-(trifluoromethyl)phenyl)-1*H*-indole (1e)

The reaction was performed at 1.5 mmol scale according to procedure A. Purification via silica gel column chromatography (petroleum ether/ethyl acetate =80/1, v/v) afforded the product **1e** as a white solid (184 mg, 32% yield). ^{1}H NMR (400 MHz, $CDCl_3$): δ = 7.50 (dd, J = 8.3, 0.9 Hz, 1H), 7.47 (dd, J = 7.0, 1.0 Hz, 1H), 7.41 (d, J = 8.0 Hz, 2H), 7.34 – 7.24 (m, 4H), 7.15 (t, J = 7.6 Hz, 2H), 7.09 (dd, J = 8.0, 1.4 Hz, 2H), 7.03 (s, 1H), 4.71 (s, 2H), 3.85 (s, 3H) ppm. ^{13}C NMR (100 MHz, $CDCl_3$): δ = 139.9, 136.0, 135.1, 132.1, 131.7, 131.2, 130.8, 129.2, 128.6 (q, J_{C-F} = 32.1 Hz), 128.4, 127.6, 124.6 (q, J_{C-F} = 3.7 Hz), 124.5 (q, J_{C-F} = 270.2 Hz), 122.7, 121.6, 118.1, 112.0, 32.9 ppm. ^{19}F NMR (565 MHz, $CDCl_3$): δ = -62.32 ppm. HRMS (ESI): calcd for $C_{22}H_{19}F_3NSi$ $[M+H]^+$ 382.1233, found 382.1229.

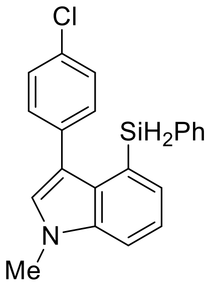

3-(4-Chlorophenyl)-1-methyl-4-(phenylsilyl)-1*H*-indole (1f)

The reaction was performed at 1.4 mmol scale according to procedure B. Purification via silica gel column chromatography (petroleum ether) afforded the product **1f** as a white solid (208 mg, 43% yield). ^{1}H NMR (400 MHz, $CDCl_3$): δ = 7.47 (dd, J = 8.2, 0.9 Hz, 1H), 7.41 (dd, J = 7.0, 1.0 Hz, 1H), 7.31 – 7.25 (m, 2H), 7.23 – 7.17 (m, 4H), 7.16 – 7.09 (m, 4H), 7.00 (s, 1H), 4.69 (s, 2H), 3.83 (s, 3H) ppm. ^{13}C NMR (100 MHz, $CDCl_3$): δ = 135.9, 135.3, 134.4, 132.7, 132.3, 132.1, 131.5, 131.3, 129.2, 128.2, 127.8, 127.6, 122.8, 121.4, 118.1, 111.8, 32.8 ppm. HRMS (ESI): calcd for $C_{21}H_{19}ClNSi$ $[M+H]^+$ 348.0970, found 348.0967.

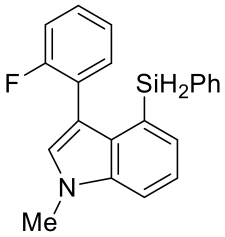

3-(2-Fluorophenyl)-1-methyl-4-(phenylsilyl)-1*H*-indole (1g)

The reaction was performed at 3.3 mmol scale according to procedure A. Purification via silica gel column chromatography (petroleum ether/ethyl acetate =100/1, v/v) afforded the product **1g** as a pale yellow oil (624 mg, 57% yield). ^{1}H NMR (600 MHz, $CDCl_3$): δ = 7.47 (d, J = 8.2 Hz, 1H), 7.36 (d, J = 6.9 Hz, 1H), 7.30 – 7.22 (m, 3H), 7.22 – 7.16 (m, 5H), 7.07 (s, 1H), 7.02 – 6.93 (m, 2H), 4.65 (s, 2H), 3.83 (s, 3H) ppm. ^{13}C NMR (150 MHz, $CDCl_3$): δ = 161.2 (d, J_{C-F} = 243.5 Hz), 135.9, 135.4, 133.9 (d, J_{C-F} = 2.4 Hz), 132.3, 132.0, 130.9, 129.1, 128.9, 128.9, 127.5, 123.6 (d, J_{C-F} = 16.5 Hz), 123.3 (d, J_{C-F} = 3.5 Hz), 122.9, 121.3, 115.2 (d, J_{C-F} = 22.4 Hz), 111.8, 111.4, 32.9 ppm. ^{19}F NMR (565 MHz, $CDCl_3$): δ = -113.21 ppm. HRMS (ESI): calcd for $C_{21}H_{19}FNSi$ $[M+H]^+$ 332.1265, found 332.1262.

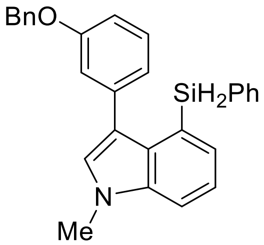

3-(3-(Benzyloxy)phenyl)-1-methyl-4-(phenylsilyl)-1*H*-indole (1h)

The reaction was performed at 2.0 mmol scale according to procedure A. Purification via silica gel column chromatography (petroleum ether/ethyl acetate =40/1, v/v) afforded the product **1h** as a white solid (433 mg, 52% yield). ^{1}H NMR (400 MHz, $CDCl_3$): δ = 7.47 (d, J = 8.2 Hz, 1H), 7.44 – 7.34 (m, 5H), 7.34 – 7.24 (m, 4H), 7.24 – 7.13 (m, 4H), 7.03 (s, 1H), 6.90 (t, J = 9.5 Hz, 2H), 6.85 (s, 1H), 4.88 (s, 2H), 4.69 (d, J = 1.6 Hz, 2H), 3.83 (s, 3H) ppm. ^{13}C NMR (100 MHz, $CDCl_3$): δ = 158.3, 137.4, 137.2, 135.8, 135.4, 132.7, 131.6, 131.1, 129.0, 128.8, 128.5, 128.1, 127.8, 127.6, 127.5, 123.9, 122.9, 121.3, 119.3, 116.7, 113.9, 111.7, 69.6, 32.8 ppm. HRMS (ESI): calcd for $C_{28}H_{26}NOSi$ $[M+H]^+$ 420.1778, found 420.1772.

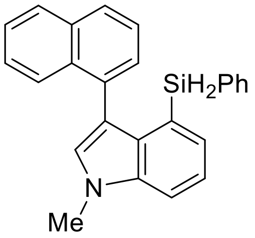

1-Methyl-3-(naphthalen-1-yl)-4-(phenylsilyl)-1*H*-indole (1i)

The reaction was performed at 3.3 mmol scale according to procedure A. Purification via silica gel column chromatography (petroleum ether/ethyl acetate =80/1, v/v) afforded the product **1i** as a white solid (740 mg, 62% yield). ^{1}H NMR (400 MHz, $CDCl_3$): δ = 7.84 – 7.77 (m, 2H), 7.61 (d, J = 8.5 Hz, 1H), 7.47 (dd, J = 8.1, 1.1 Hz, 1H), 7.39 – 7.34 (m, 1H), 7.33 – 7.23 (m, 4H), 7.22 – 7.17 (m, 1H), 7.15 – 7.09 (m, 1H), 7.05 (s, 1H), 7.05 – 6.97 (m, 4H), 4.18 (d, J = 6.0 Hz, 1H), 4.11 (d, J = 6.0 Hz, 1H), 3.80 (s, 3H) ppm. The “SiH_2” group of this compound displays two doublet peaks, which suggests the protons on Si are diastereotopic. ^{13}C NMR (100 MHz, $CDCl_3$): δ = 135.8, 135.1, 134.5, 133.4, 133.4, 133.0, 132.4, 130.6, 129.7, 129.1, 128.8, 127.8, 127.6, 127.3, 126.9, 125.6, 125.5, 125.1, 123.4, 121.3, 116.1, 111.6, 32.8 ppm. HRMS (ESI): calcd for $C_{25}H_{22}NSi$ $[M+H]^+$ 364.1516, found 364.1513.

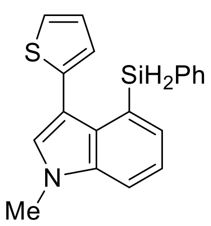

1-Methyl-4-(phenylsilyl)-3-(thiophen-2-yl)-1*H*-indole (1j)

The reaction was performed at 2.0 mmol scale according to procedure A. Purification via silica gel column chromatography (petroleum ether/ethyl acetate =80/1, v/v) afforded the product **1j** as a brown oil (224 mg, 35% yield). ^{1}H NMR (600 MHz, $CDCl_3$): δ = 7.46 (d, J = 8.2 Hz, 1H), 7.35 – 7.31 (m, 4H), 7.28 – 7.25 (m, 4H), 7.15 (s, 1H), 7.00 – 6.98 (m, 1H), 6.86 (d, J = 2.7 Hz, 1H), 4.65 (s, 2H), 3.83 (s, 3H) ppm. ^{13}C NMR (150 MHz, $CDCl_3$): δ = 136.3, 135.7, 135.5, 132.8, 132.6, 131.1, 129.7, 129.1, 129.1, 127.6, 126.9, 125.6, 123.2, 121.5, 111.7, 110.3, 32.9 ppm. HRMS (ESI): calcd for $C_{19}H_{18}NSSi$ $[M+H]^+$ 320.0924, found 320.0921.

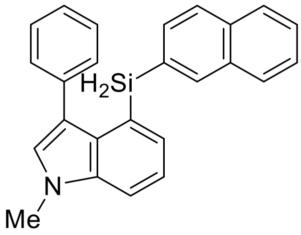

1-Methyl-4-(naphthalen-2-ylsilyl)-3-phenyl-1*H*-indole (1k)

The reaction was performed at 2.5 mmol scale according to procedure A. Purification via silica gel column chromatography (petroleum ether/ethyl acetate =40/1, v/v) afforded the product **1k** as a colorless oil (618 mg, 68% yield). ^{1}H NMR (600 MHz, $CDCl_3$): δ = 7.75 (d, J = 7.7 Hz, 1H), 7.72 (s, 1H), 7.69 (d, J = 7.7 Hz, 1H), 7.66 (d, J = 8.0 Hz, 1H), 7.47 – 7.40 (m, 4H), 7.30 – 7.24 (m, 5H), 7.20 – 7.17 (m, 2H), 7.01 (s, 1H), 4.78 (s, 2H), 3.80 (s, 3H) ppm. ^{13}C NMR (150 MHz, $CDCl_3$): δ = 136.5, 136.0, 135.9, 133.7, 132.8, 131.7, 131.3, 131.1, 131.0, 130.3, 128.2, 128.1, 127.8, 127.6, 126.8, 126.7, 126.3, 125.7, 122.9, 121.2, 119.4, 111.7, 32.8 ppm. HRMS (ESI): calcd for $C_{25}H_{22}NSi$ $[M+H]^+$ 364.1516, found 364.1510.

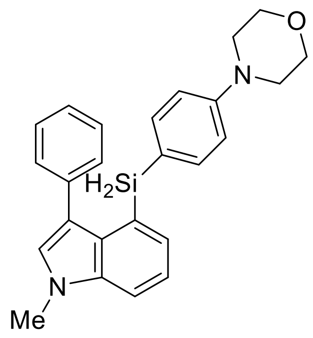

4-(4-((1-Methyl-3-phenyl-1*H*-indol-4-yl)silyl)phenyl)morpholine (1l)

The reaction was performed at 1.0 mmol scale according to procedure A. Purification via silica gel column chromatography (petroleum ether/ethyl acetate =20/1, v/v) afforded the product **1l** as a white solid (251 mg, 63% yield). ^{1}H NMR (600 MHz, $CDCl_3$): δ = 7.43 (d, J = 8.1 Hz, 1H), 7.33 – 7.23 (m, 7H), 7.18 (d, J = 8.5 Hz, 2H), 7.03 (s, 1H), 6.77 (d, J = 8.5 Hz, 2H), 4.59 (s, 2H), 3.84 – 3.82 (m, 4H), 3.81 (s, 3H), 3.15 – 3.13 (m, 4H) ppm. ^{13}C NMR (150 MHz, $CDCl_3$): δ = 151.8, 136.7, 136.2, 135.8, 131.6, 131.0, 130.7, 128.1, 127.8, 126.7, 123.8, 121.9, 121.2, 119.5, 114.7, 111.4, 66.8, 48.7, 32.7 ppm. HRMS (ESI): calcd for $C_{25}H_{27}N_2OSi$ $[M+H]^+$ 399.1887, found 399.1884.

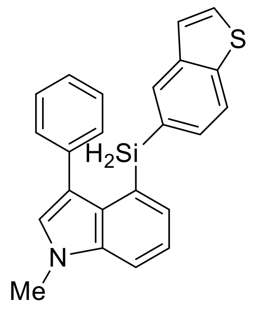

4-(Benzo[*b*]thiophen-5-ylsilyl)-1-methyl-3-phenyl-1*H*-indole (1m)

The reaction was performed at 2.5 mmol scale according to procedure A. Purification via silica gel column chromatography (petroleum ether/ethyl acetate =80/1, v/v) afforded the product **1m** as an orange solid (240 mg, 26% yield). ^{1}H NMR (600 MHz, $CDCl_3$): δ = 7.53 (d, J = 2.1 Hz, 1H), 7.44 (t, J = 4.0 Hz, 2H), 7.38 (d, J = 6.7 Hz, 1H), 7.34 (d, J = 8.2 Hz, 1H), 7.27 – 7.23 (m, 4H), 7.21 – 7.18 (m, 2H), 7.14 (d, J = 8.2 Hz, 1H), 6.99 (s, 1H), 6.62 (d, J = 1.5 Hz, 1H), 4.74 (s, 2H), 3.78 (s, 3H) ppm. ^{13}C NMR (150 MHz, $CDCl_3$): δ = 155.8, 144.6, 136.0, 135.9, 131.6, 131.2, 131.0, 128.9, 128.2, 127.7, 127.1, 126.7, 126.0, 123.5, 121.2, 119.4, 111.7, 110.8, 106.3,

32.7 ppm. HRMS (ESI): calcd for $C_{23}H_{20}NSSi$ [M+H]$^+$ 370.1080, found 370.1077.

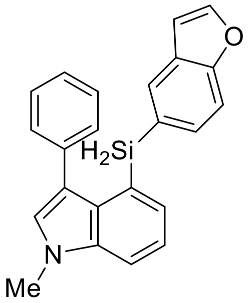

4-(Benzofuran-5-ylsilyl)-1-methyl-3-phenyl-1*H*-indole (1n)

The reaction was performed at 2.5 mmol scale according to procedure A. Purification via silica gel column chromatography (petroleum ether/ethyl acetate =80/1, v/v) afforded the product **1n** as an orange solid (274 mg, 31% yield). ^{1}H NMR (600 MHz, $CDCl_3$): δ = 7.56 (d, J = 2.1 Hz, 1H), 7.47 (d, J = 8.2 Hz, 1H), 7.45 (s, 1H), 7.38 (d, J = 6.9 Hz, 1H), 7.35 (d, J = 8.2 Hz, 1H), 7.29 – 7.23 (m, 4H), 7.21 – 7.19 (m, 2H), 7.14 (d, J = 8.2 Hz, 1H), 7.02 (s, 1H), 6.65 (d, J = 2.0 Hz, 1H), 4.73 (s, 2H), 3.83 (s, 3H) ppm. ^{13}C NMR (150 MHz, $CDCl_3$): δ = 155.8, 144.6, 136.1, 135.9, 131.6, 131.2, 131.0, 131.0, 129.0, 128.2, 127.7, 127.1, 126.7, 126.1, 123.5, 121.2, 119.4, 111.7, 110.8, 106.4, 32.8 ppm. HRMS (ESI): calcd for $C_{23}H_{20}NOSi$ [M+H]$^+$ 354.1309, found 354.1304.

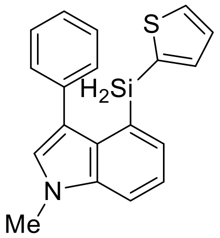

1-Methyl-3-phenyl-4-(thiophen-2-ylsilyl)-1*H*-indole (1o)

The reaction was performed at 2.5 mmol scale according to procedure A. Purification via silica gel column chromatography (petroleum ether/ethyl acetate =80/1, v/v) afforded the product **1o** as a pale yellow solid (176 mg, 22% yield). ^{1}H NMR (600 MHz, $CDCl_3$): δ = 7.57 (d, J = 4.5 Hz, 1H), 7.47 (d, J = 8.2 Hz, 1H), 7.37 – 7.31 (m, 6H), 7.26 (d, J = 7.4 Hz, 1H), 7.10 – 7.08 (m, 2H), 7.06 (s, 1H), 4.72 (s, 2H), 3.84 (s, 3H) ppm. ^{13}C NMR (150 MHz, $CDCl_3$): δ = 137.5, 136.1, 135.8, 131.9, 131.6, 130.9, 130.5, 128.2, 128.1, 127.9, 126.9, 122.6, 121.3, 119.3, 111.8,

32.8 ppm. HRMS (ESI): calcd for $C_{19}H_{18}NSSi$ $[M+H]^+$ 320.0924, found 320.0921.

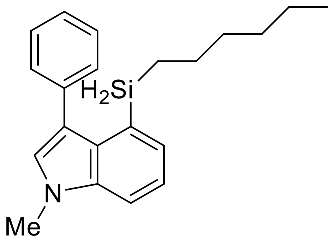

4-(Hexylsilyl)-1-methyl-3-phenyl-1*H*-indole (1p)

The reaction was performed at 1.0 mmol scale according to literature[4,9]. Purification via silica gel column chromatography (petroleum ether/ethyl acetate =80/1, v/v) afforded the product **1p** as a colorless oil (226 mg, 70% yield). ^{1}H NMR (600 MHz, $CDCl_3$): δ = 7.47 – 7.41 (m, 4H), 7.39 (t, J = 7.3 Hz, 2H), 7.36 – 7.32 (m, 1H), 7.27 – 7.23 (m, 1H), 7.06 (s, 1H), 4.12 (t, J = 3.8 Hz, 2H), 3.83 (s, 1H), 1.22 – 1.15 (m, 2H), 1.13 – 1.06 (m, 6H), 0.82 (t, J = 7.3 Hz, 3H), 0.37 – 0.31 (m, 2H) ppm. ^{13}C NMR (150 MHz, $CDCl_3$): δ = 136.3, 135.8, 131.5, 130.9, 130.2, 128.0, 127.9, 126.8, 124.7, 121.1, 119.3, 111.3, 32.6, 32.3, 31.4, 25.1, 22.5, 14.1, 10.3 ppm. HRMS (ESI): calcd for $C_{21}H_{28}NSi$ $[M+H]^+$ 322.1986, found 322.1984.

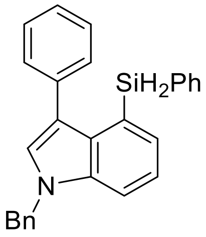

1-Benzyl-3-phenyl-4-(phenylsilyl)-1*H*-indole (1q)

The reaction was performed at 2.6 mmol scale according to procedure A. Purification via silica gel column chromatography (petroleum ether) afforded the product **1q** as a white solid (428 mg, 42% yield). ^{1}H NMR (400 MHz, $CDCl_3$): δ = 7.43 (dd, J = 8.3, 0.9 Hz, 1H), 7.36 – 7.14 (m, 17H), 7.11 (s, 1H), 5.35 (s, 2H), 4.64 (s, 2H) ppm. ^{13}C NMR (100 MHz, $CDCl_3$): δ = 137.1, 136.0, 135.5, 135.5, 132.7, 132.0, 131.2, 131.0, 129.1, 128.8, 127.8, 127.7, 127.6, 127.5, 127.0, 126.8, 123.2, 121.4, 120.0, 112.1, 50.1 ppm. HRMS (ESI): calcd for $C_{27}H_{24}NSi$ $[M+H]^+$ 390.1673, found 390.1668.

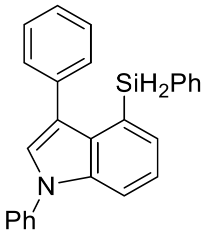

1,3-Diphenyl-4-(phenylsilyl)-1*H*-indole (1r)

The reaction was performed at 1.3 mmol scale according to procedure A. Purification via silica gel column chromatography (petroleum ether) afforded the product **1r** as a white solid (243 mg, 50% yield). ^{1}H NMR (400 MHz, $CDCl_3$): δ = 7.69 (d, J = 8.3 Hz, 1H), 7.57 – 7.48 (m, 4H), 7.43 – 7.20 (m, 14H) ppm. ^{13}C NMR (100 MHz, $CDCl_3$): δ = 139.4, 135.5, 135.5, 135.2, 132.6, 132.6, 131.9, 131.0, 129.7, 129.2, 127.9, 127.6, 127.2, 127.1, 126.8, 124.7, 123.4, 122.0, 121.6, 112.9 ppm. HRMS (ESI): calcd for $C_{26}H_{22}NSi$ $[M+H]^+$ 376.1516, found 376.1515.

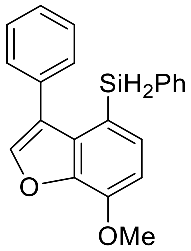

(7-Methoxy-3-phenylbenzofuran-4-yl)(phenyl)silane (1s)

The reaction was performed at 1.0 mmol scale according to procedure A. Purification via silica gel column chromatography (petroleum ether/ethyl acetate =80/1, v/v) afforded the product **1s** as a white solid (220 mg, 67% yield). ^{1}H NMR (400 MHz, $CDCl_3$): δ = 7.59 (s, 1H), 7.40 (d, J = 7.9 Hz, 1H), 7.37 – 7.16 (m, 10H), 6.85 (d, J = 7.9 Hz, 1H), 4.59 (s, 2H), 4.05 (s, 3H) ppm. ^{13}C NMR (100 MHz, $CDCl_3$): δ = 147.5, 144.2, 142.6, 135.3, 134.9, 134.1, 132.2, 131.8, 130.5, 129.3, 128.1, 127.9, 127.7, 124.8, 115.1, 106.2, 56.0 ppm. HRMS (ESI): calcd for $C_{21}H_{19}O_2Si$ $[M+H]^+$ 331.1149, found 331.1146.

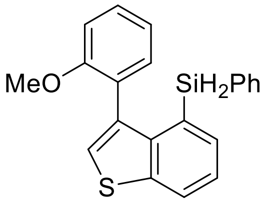

(3-(2-Methoxyphenyl)benzo[*b*]thiophen-4-yl)(phenyl)silane (1t)

The reaction was performed at 3.1 mmol scale according to procedure A. Purification via silica gel column chromatography (petroleum ether/ethyl acetate =80/1, v/v) afforded the product **1t** as a colorless viscous oil (717 mg, 67% yield). ^{1}H NMR (400 MHz, $CDCl_3$): δ = 7.99 (dd, J = 8.1, 1.1 Hz, 1H), 7.50 (dd, J = 7.0, 1.1 Hz, 1H), 7.41 – 7.27 (m, 4H), 7.26 – 7.22 (m, 4H), 7.10 (dd, J = 7.4, 1.8 Hz, 1H), 6.85 (td, J = 7.4, 1.0 Hz, 1H), 6.79 (d, J = 8.3 Hz, 1H), 4.37 (d, J = 6.2 Hz, 1H), 4.32 (d, J = 6.2 Hz, 1H), 3.48 (s, 3H) ppm. The "SiH_2" group of this compound displays two doublet peaks, which suggests the protons on Si are diastereotopic. ^{13}C NMR (100 MHz, $CDCl_3$): δ = 158.3, 143.4, 140.3, 136.5, 135.7, 135.5, 133.0, 132.5, 129.8, 129.2, 127.6, 126.8, 125.6, 125.5, 125.1, 123.2, 120.0, 110.5, 54.9 ppm. HRMS (ESI): calcd for $C_{21}H_{19}OSSi$ $[M+H]^+$ 347.0920, found 347.0918.

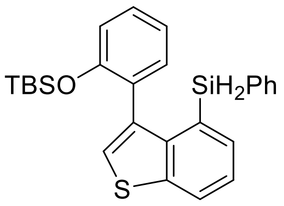

***tert*-Butyldimethyl(2-(4-(phenylsilyl)benzo[*b*]thiophen-3-yl)phenoxy)silane (1u)**

The reaction was performed at 1.6 mmol scale according to procedure A. Purification via silica gel column chromatography (petroleum ether/ethyl acetate =80/1, v/v) afforded the product **1u** as a colorless oil (350 mg, 45% yield). ^{1}H NMR (400 MHz, $CDCl_3$): δ = 7.95 (dd, J = 8.1, 1.1 Hz, 1H), 7.44 (dd, J = 7.1, 1.1 Hz, 1H), 7.35 – 7.22 (m, 8H), 7.12 – 7.07 (m, 1H), 6.87 – 6.80 (m, 2H), 4.49 (d, J = 6.1 Hz, 1H), 4.33 (d, J = 6.1 Hz, 1H), 0.50 (s, 9H), 0.04 (s, 3H), -0.10 (s, 3H) ppm. The "SiH_2" group of this compound displays two doublet peaks, which suggests the protons on Si are diastereotopic. ^{13}C NMR (100 MHz, $CDCl_3$): δ = 154.8, 143.5, 140.1, 137.2, 135.6, 135.3, 132.9, 132.7, 129.5, 129.3, 128.7, 127.7, 127.2, 125.4, 124.8, 123.1, 120.8, 119.1, 25.0, 17. 6, -4.2, -4.8 ppm. HRMS (ESI): calcd for $C_{26}H_{31}OSSi_2$ $[M+H]^+$ 447.1629, found 447.1627.

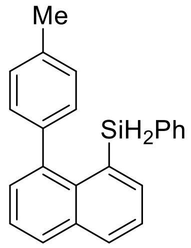

Phenyl(8-(*p*-tolyl)naphthalen-1-yl)silane (1v)

The reaction was performed at 3.4 mmol scale according to literature[1,2,10]. Purification via silica gel column chromatography (petroleum ether) afforded the product **1v** as a white solid (648 mg, 64% yield). ^{1}H NMR (600 MHz, $CDCl_3$): δ = 7.96 (d, J = 8.1 Hz, 1H), 7.87 (dd, J = 8.1, 1.1 Hz, 1H), 7.72 (dd, J = 6.8, 1.2 Hz, 1H), 7.49 (t, J = 7.8 Hz, 1H), 7.41 (t, J = 7.8 Hz, 1H), 7.36 (dd, J = 7.0, 1.3 Hz, 1H), 7.33 – 7.29 (m, 1H), 7.26 – 7.19 (m, 4H), 7.14 (d, J = 7.9 Hz, 2H), 7.09 (d, J = 7.7 Hz, 2H), 4.16 (s, 2H), 2.41 (s, 3H) ppm. ^{13}C NMR (150 MHz, $CDCl_3$): δ = 142.0, 139.8, 139.5, 137.6, 136.6, 135.3, 134.7, 134.5, 131.3, 131.2, 129.9, 129.4, 129.1, 129.1, 128.7, 127.6, 124.9, 124.7, 21.3 ppm.

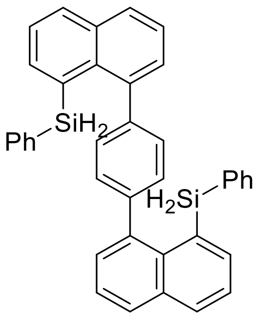

1,4-Bis(8-(phenylsilyl)naphthalen-1-yl)benzene (1w)

The reaction was performed at 1.0 mmol scale according to literature[1,2,10]. Purification via silica gel column chromatography (petroleum ether) afforded the product **1w** as a white solid (266 mg, 49% yield). ^{1}H NMR (400 MHz, $CDCl_3$): δ = 8.04 (d, J = 8.0 Hz, 2H), 7.97 (d, J = 7.9 Hz, 2H), 7.82 (d, J = 6.5 Hz, 2H), 7.62 (t, J = 7.5 Hz, 2H), 7.54 (d, J = 6.8 Hz, 2H), 7.49 (t, J = 7.4 Hz, 2H), 7.33 – 7.27 (m, 10H), 7.20 (s, 4H), 4.34 (s, 4H) ppm. ^{13}C NMR (100 MHz, $CDCl_3$): δ = 142.2, 141.8, 140.0, 136.4, 135.2, 134.5, 131.5, 131.2, 129.8, 129.8, 129.2, 129.0, 127.7, 125.0, 124.8 ppm.

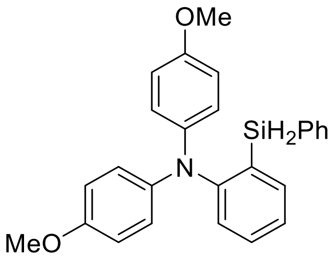

***N*,*N*-Bis(4-methoxyphenyl)-2-(phenylsilyl)aniline (1x)**

The reaction was performed at 5.5 mmol scale according to literature[1,2,11]. Purification via silica gel column chromatography (petroleum ether) afforded the product **1x** as a pale yellow solid (766 mg, 34% yield). ^{1}H NMR (400 MHz, $CDCl_3$): δ = 7.40 (dd, J = 7.3, 1.2 Hz, 1H), 7.34 (d, J = 6.6 Hz, 2H), 7.26 – 7.18 (m, 2H), 7.15 (t, J = 7.1 Hz, 2H), 6.98 (t, J = 7.3 Hz, 1H), 6.92 (d, J = 8.0 Hz, 1H), 6.67 (d, J = 9.0 Hz, 4H), 6.58 (d, J = 9.0 Hz, 4H), 4.39 (s, 2H), 3.60 (s, 6H) ppm. ^{13}C NMR (100 MHz, $CDCl_3$): δ = 154.7, 154.4, 142.4, 138.7, 135.6, 132.0, 131.7, 130.9, 129.4, 127.8, 127.7, 124.2, 124.2, 114.2, 55.3 ppm. HRMS (ESI): calcd for $C_{26}H_{26}NO_2Si$ $[M+H]^+$ 412.1727, found 412.1719.

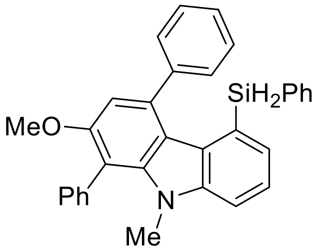

2-Methoxy-9-methyl-1,4-diphenyl-5-(phenylsilyl)-9*H*-carbazole (3a)

The reaction was performed at 3.0 mmol scale according to procedure C. Purification via silica gel column chromatography (petroleum ether/ethyl acetate =80/1, v/v) afforded the product **3a** as a white solid (985 mg, 70% yield). ^{1}H NMR (400 MHz, $CDCl_3$): δ = 7.64 – 7.58 (m, 2H), 7.51 – 7.46 (m, 4H), 7.45 – 7.40 (m, 3H), 7.40 – 7.34 (m, 1H), 7.32 – 7.18 (m, 7H), 7.05 (dd, J = 6.9, 1.0 Hz, 1H), 6.76 (s, 1H), 3.97 (s, 2H), 3.81 (s, 3H), 3.22 (s, 3H) ppm. ^{13}C NMR (150 MHz, $CDCl_3$): δ = 155.7, 143.0, 142.3, 141.5, 137.8, 135.9, 135.7, 134.8, 131.6, 130.1, 129.9, 129.4, 129.0, 127.9, 127.7, 127.6, 127.3, 125.0, 123.8, 116.7, 112.2, 110.0, 107.5, 56.6, 32.6 ppm. HRMS (ESI): calcd for $C_{32}H_{28}NOSi$ $[M+H]^+$ 470.1935, found 470.1922.

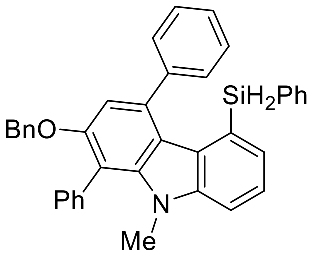

2-(Benzyloxy)-9-methyl-1,4-diphenyl-5-(phenylsilyl)-9*H*-carbazole (3b)

The reaction was performed at 1.0 mmol scale according to procedure C. Purification via silica gel column chromatography (petroleum ether/ethyl acetate =80/1, v/v) afforded the product **3b** as a white solid (283 mg, 52% yield). ^{1}H NMR (600 MHz, $CDCl_3$): δ = 7.58 (d, J = 7.3 Hz, 2H), 7.51 (d, J = 6.9 Hz, 2H), 7.48 (t, J = 7.4 Hz, 2H), 7.45 – 7.40 (m, 3H), 7.36 (t, J = 7.3 Hz, 1H), 7.32 – 7.24 (m, 6H), 7.23 – 7.19 (m, 4H), 7.12 (d, J = 7.1 Hz, 2H), 7.07 (d, J = 7.0 Hz, 1H), 6.83 (s, 1H), 5.08 (s, 2H), 3.97 (s, 2H), 3.27 (s, 3H) ppm. ^{13}C NMR (150 MHz, $CDCl_3$): δ = 154.7, 142.9, 142.3, 141.4, 137.8, 137.5, 135.9, 135.7, 134.8, 131.6, 130.1, 130.0, 129.4, 129.0, 128.2, 127.9, 127.7, 127.60, 127.4, 127.3, 126.7, 125.1, 123.9, 117.1, 113.4, 110.0, 109.7, 71.3, 32.6 ppm. HRMS (ESI): calcd for $C_{38}H_{32}NOSi$ $[M+H]^+$ 546.2248, found 546.2249.

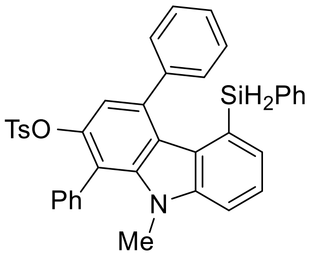

9-Methyl-1,4-diphenyl-5-(phenylsilyl)-9*H*-carbazol-2-yl 4-methylbenzenesulfonate (3c)

The reaction was performed at 1.0 mmol scale according to procedure C. Purification via silica gel column chromatography (petroleum ether/ethyl acetate =40/1, v/v) afforded the product **3c** as a white solid (340 mg, 56% yield). ^{1}H NMR (600 MHz, $CDCl_3$): δ = 7.50 (d, J = 7.8 Hz, 2H), 7.42 – 7.34 (m, 8H), 7.34 – 7.26 (m, 5H), 7.24 – 7.20 (m, 4H), 7.17 – 7.12 (m, 3H), 7.01 (d, J = 2.1 Hz, 1H), 3.96 (d, J = 1.9 Hz, 2H), 3.21 (s, 3H), 2.41 (s, 3H) ppm. ^{13}C NMR (150 MHz, $CDCl_3$): δ = 145.0, 144.7, 142.5, 141.7, 140.4, 137.7, 135.7, 134.4, 133.8, 133.2, 131.5, 130.6, 130.0, 129.5, 129.4, 129.1, 128.3, 127.9, 127.8, 127.7, 127.6, 126.8, 126.4, 125.0, 121.0, 117.6, 116.6, 110.5, 32.6, 21.6 ppm. HRMS (ESI): calcd for $C_{38}H_{32}NO_3SSi$ $[M+H]^+$ 610.1867, found

610.1865.

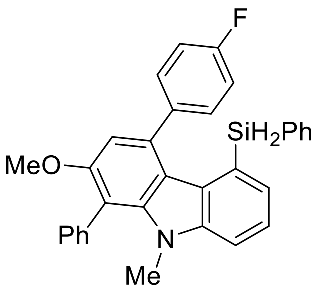

4-(4-Fluorophenyl)-2-methoxy-9-methyl-1-phenyl-5-(phenylsilyl)-9*H*-carbazole (3d)

The reaction was performed at 1.2 mmol scale according to procedure C. Purification via silica gel column chromatography (petroleum ether/ethyl acetate =80/1, v/v) afforded the product **3d** as a white solid (380 mg, 65% yield). ^{1}H NMR (600 MHz, $CDCl_3$): δ = 7.58 – 7.52 (m, 2H), 7.51 – 7.45 (m, 4H), 7.45 – 7.41 (m, 1H), 7.33 – 7.21 (m, 7H), 7.15 – 7.05 (m, 3H), 6.69 (s, 1H), 4.03 (s, 2H), 3.81 (s, 3H), 3.21 (s, 3H) ppm. ^{13}C NMR (150 MHz, $CDCl_3$): δ = 162.7 (d, $J_{C\text{-}F}$ = 245.4 Hz), 155.6, 142.3, 141.5, 139.0 (d, $J_{C\text{-}F}$ = 2.7 Hz), 136.6, 135.8, 135.7, 134.3, 131.7 (d, $J_{C\text{-}F}$ = 7.8 Hz), 131.5, 130.1, 129.1, 128.0, 127.7, 127.6, 127.4, 124.6, 124.0, 116.7, 116.3 (d, $J_{C\text{-}F}$ = 21.5 Hz), 112.4, 110.1, 107.5, 56.6, 32.5 ppm. ^{19}F NMR (565 MHz, $CDCl_3$): δ = -114.88 ppm. HRMS (ESI): calcd for $C_{32}H_{27}FNOSi$ $[M+H]^+$ 488.1840, found 488.1839.

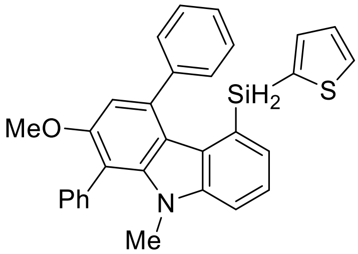

2-Methoxy-9-methyl-1,4-diphenyl-5-(thiophen-2-ylsilyl)-9*H*-carbazole (3e)

The reaction was performed at 1.0 mmol scale according to procedure C. Purification via silica gel column chromatography (petroleum ether/ethyl acetate =80/1, v/v) afforded the product **3e** as a white solid (190 mg, 40% yield). ^{1}H NMR (600 MHz, $CDCl_3$): δ = 7.64 (d, J = 7.4 Hz, 2H), 7.54 (d, J = 4.5 Hz, 1H), 7.50 – 7.42 (m, 7H), 7.40 (t, J = 7.4 Hz, 1H), 7.31 (d, J = 8.0 Hz, 1H), 7.27 (t, J = 7.5 Hz, 1H), 7.14 (d, J = 6.9 Hz, 1H), 7.11 (d, J = 2.9 Hz, 1H), 7.08 (t, J = 3.9 Hz, 1H), 6.77 (s, 1H), 4.07 (s, 2H), 3.82 (s, 3H), 3.21 (s, 3H) ppm. ^{13}C NMR (150 MHz, $CDCl_3$): δ

= 155.7, 142.8, 142.2, 141.5, 137.7, 137.4, 135.9, 133.0, 131.9, 131.6, 130.1, 129.5, 129.5, 128.0, 128.0, 127.8, 127.4, 127.3, 124.6, 123.9, 116.6, 112.3, 110.2, 107.5, 56.6, 32.6 ppm. HRMS (ESI): calcd for $C_{30}H_{26}NOSSi$ $[M+H]^+$ 476.1499, found 476.1501.

III. Optimization for 7-Membered Si-Stereogenic Heterocycles

A 5 mL microwave tube was charged with dihydrosilanes **3a** (0.1 mmol, 46.9 mg, 1.0 equiv), $[Rh(cod)Cl]_2$ (0.5 mg, 1 mol%), ligand (3 mol%), and toluene (1 mL) in glovebox. The tube was capped, then removed from the glovebox and stirred at indicated temperature for 12 h. After the system was cooled to room temperature, the reaction mixture was concentrated *in vacuo*, the crude residue was dissolved in 1 mL of deuterated chloroform for ^{1}H NMR analysis. The yields were determined by crude ^{1}H NMR analysis using dibromomethane as an internal standard. The *ee* value of desired compound **4a** was determined by HPLC analysis on a chiral stationary phase. The result of optimization of reaction conditions was listed in **Supplementary Table 1**.

Supplementary Table 1 Optimization of reaction conditions.[a]

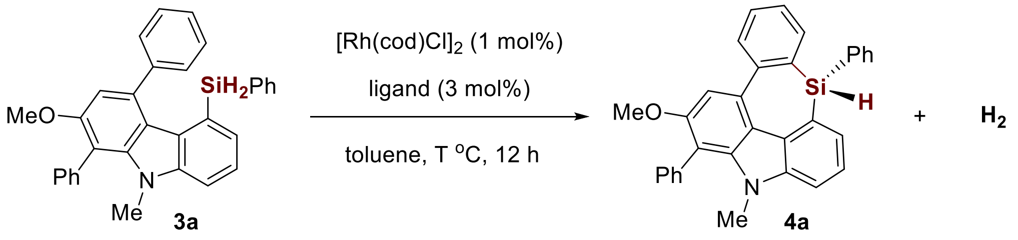

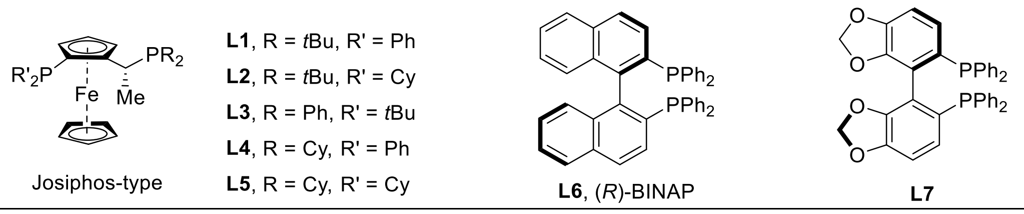

Entry	Ligand	T (°C)	Yield (%)[b]	*ee* (%)[d]
1	L1	70	50	69
2	L1	100	72	76
3	L1	120	70	72
4	L2	100	35	42
**5**	**L3**	**100**	**83 (78[c])**	**90**
6	L4	100	76	10
7	L5	100	67	3
8	L6	100	16	36
9	L7	100	25	29

[a]Reaction conditions: **3a** (46.9 mg, 0.1 mmol), $[Rh(cod)Cl]_2$ (0.5 mg, 1 mol%) and chiral ligand (3 mol%) in anhydrous toluene (1.0 mL) at indicated temperature for 12 h. [b]NMR yield. [c]Isolated yield. [d]*ee* value was based on chiral HPLC analysis: AD-3 column, wavelength = 250 nm, eluents: hexane/isopropanol = 95/5, flow rate = 1.0 mL/min, temperature = 28 °C.

IV. Enantioselective Construction of Si-Stereogenic Heterocycles

1. Scope of Six-Membered Silicon-Stereogenic Heterocycles

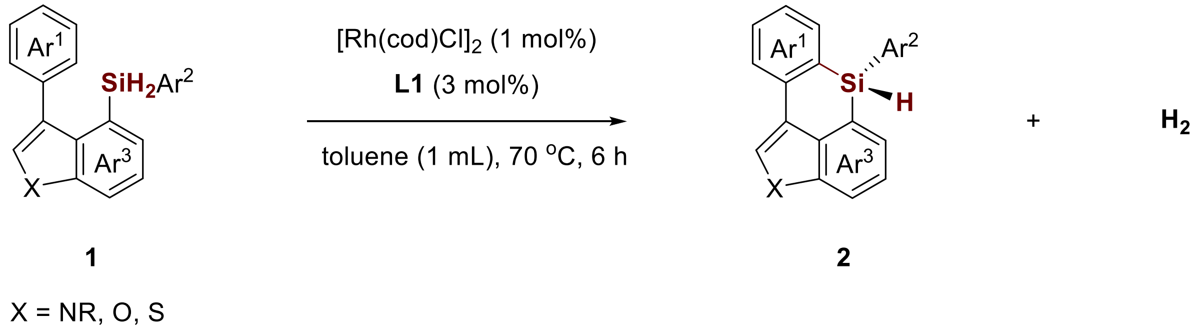

A 5 mL microwave tube was charged with **1** (0.1 mmol, 1 equiv), $[Rh(cod)Cl]_2$ (0.5 mg, 1 mol%), **L1** (1.6 mg, 3 mol%), and toluene (1 mL) in glovebox. The tube was sealed, then removed from the glovebox, and the mixture was stirred at 70 °C for 6 h. After the completion of the reaction, the solvent was evaporated under reduced pressure and the residue was purified by flash column chromatography on silica gel to give the product.

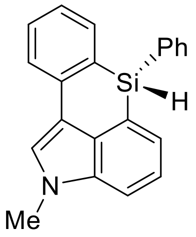

(*S*)-2-Methyl-6-phenyl-2,6-dihydrobenzo[5,6]silino[4,3,2-*cd*]indole (2a)

The product **2a** was purified via silica gel column chromatography (petroleum ether/ethyl acetate = 80/1, v/v) as a white solid (25.9 mg, 83% yield). ^{1}H NMR (600 MHz, $CDCl_3$): δ = 7.77 (d, J = 7.9 Hz, 1H), 7.64 – 7.59 (m, 3H), 7.58 (s, 1H), 7.45 (d, J = 6.8 Hz, 1H), 7.41 – 7.35 (m, 3H), 7.34 – 7.30 (m, 3H), 7.13 (t, J = 7.3 Hz, 1H), 5.71 (s, 1H), 3.84 (s, 3H) ppm. ^{13}C NMR (150 MHz, $CDCl_3$): δ = 139.5, 136.6, 135.7, 135.5, 135.4, 131.8, 129.9, 129.6, 128.0, 127.0, 126.7, 125.5, 124.7, 122.7, 122.5, 122.4, 115.7, 110.7, 33.0 ppm. HRMS (ESI): calcd for $C_{21}H_{18}Si$ $[M+H]^+$ 312.1203, found 312.1201. HPLC (Chiralpak OD-3) *i*-PrOH/hexane = 5/95, 1.0 mL/min, T = 28 °C, λ = 250 nm, t_r (major) = 11.7 min, t_r (minor) = 15.5 min, 96% *ee*. $[\alpha]_D^{28.3}$ = +132 (c = 0.1, $CHCl_3$).

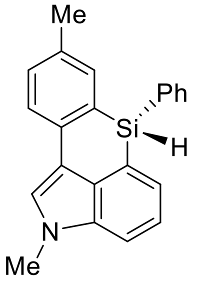

(*S*)-2,8-Dimethyl-6-phenyl-2,6-dihydrobenzo[5,6]silino[4,3,2-*cd*]indole (2b)

The product **2b** was purified via silica gel column chromatography (petroleum ether/ethyl acetate = 80/1, v/v) as a white solid (25.4 mg, 78% yield). ^{1}H NMR (400 MHz, $CDCl_3$): δ = 7.68 (d, J = 8.0 Hz, 1H), 7.65 – 7.59 (m, 2H), 7.53 (s, 1H), 7.45 – 7.40 (m, 2H), 7.39 – 7.28 (m, 5H), 7.20 (dd, J = 8.1, 1.6 Hz, 1H), 5.69 (s, 1H), 3.83 (s, 3H), 2.30 (s, 3H) ppm. ^{13}C NMR (100 MHz, $CDCl_3$): δ = 136.9, 136.8, 135.9, 135.5, 134.0, 131.8, 130.9, 129.6, 128.0, 126.9, 126.6, 125.0, 122.6, 122.5, 115.7, 110.6, 33.0, 21.1 ppm. HRMS (ESI): calcd for $C_{22}H_{20}NSi$ $[M+H]^+$ 326.1360, found 326.1361. HPLC (Chiralpak OD-3) *i*-PrOH/hexane = 5/95, 1.0 mL/min, T = 28 ^{o}C, λ = 250 nm, t_r (major) = 8.2 min, t_r (minor) = 8.9 min, 96% *ee*. $[\alpha]_D^{29.7}$ = +69 (c = 0.1, $CHCl_3$).

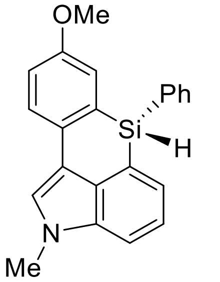

(*S*)-8-Methoxy-2-methyl-6-phenyl-2,6-dihydrobenzo[5,6]silino[4,3,2-*cd*]indole (2c)

The product **2c** was purified via silica gel column chromatography (petroleum ether/ethyl acetate = 80/1, v/v) as a white solid (26.0 mg, 76% yield). ^{1}H NMR (400 MHz, $CDCl_3$): δ = 7.71 (d, J = 8.7 Hz, 1H), 7.62 (dd, J = 7.7, 1.6 Hz, 2H), 7.46 (s, 1H), 7.43 (d, J = 6.7 Hz, 1H), 7.39 – 7.28 (m, 5H), 7.13 (d, J = 2.8 Hz, 1H), 6.96 (dd, J = 8.7, 2.8 Hz, 1H), 5.70 (s, 1H), 3.81 (s, 3H), 3.78 (s, 3H) ppm. ^{13}C NMR (100 MHz, $CDCl_3$): δ = 156.6, 135.6, 135.4, 135.4, 132.5, 131.6, 129.6, 128.4, 128.0, 126.5, 124.5, 123.9, 122.6, 122.1, 120.5, 116.5, 115.5, 110.6, 55.3, 32.9 ppm. HRMS (ESI): calcd for $C_{22}H_{20}NOSi$ $[M+H]^+$ 342.1309, found 342.1305. HPLC

(Chiralpak AD-3) *i*-PrOH/hexane = 1/99, 1.0 mL/min, T = 28 °C, λ = 250 nm, t_r (major) = 30.7 min, t_r (minor) = 48.3 min, 96% *ee*. $[\alpha]_D^{28.8}$ = +47 (c = 0.1, $CHCl_3$).

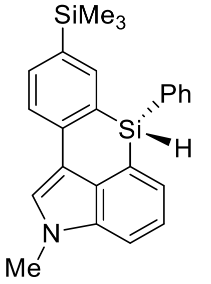

(*S*)-2-Methyl-6-phenyl-8-(trimethylsilyl)-2,6-dihydrobenzo[5,6]silino[4,3,2-*cd*]indole (2d)

The product **2d** was purified via silica gel column chromatography (petroleum ether/ethyl acetate = 40/1, v/v) as a colorless oil (27.2 mg, 71% yield). ^1H NMR (400 MHz, $CDCl_3$): δ = 7.79 (s, 1H), 7.73 (d, *J* = 7.8 Hz, 1H), 7.62 (dd, *J* = 7.6, 1.8 Hz, 2H), 7.57 (s, 1H), 7.54 (dd, *J* = 7.8, 1.3 Hz, 1H), 7.46 (d, *J* = 6.2 Hz, 1H), 7.38 – 7.29 (m, 5H), 5.73 (s, 1H), 3.81 (s, 3H), 0.24 (s, 9H) ppm. ^{13}C NMR (100 MHz, $CDCl_3$): δ = 141.8, 140.0, 135.9, 135.8, 135.5, 135.3, 134.9, 131.9, 129.5, 127.9, 126.7, 126.0, 125.7, 122.7, 122.6, 121.7, 115.7, 110.6, 33.0, -1.1 ppm. HRMS (ESI): calcd for $C_{24}H_{26}NSi_2$ $[M+H]^+$ 384.1598, found 384.1595. HPLC (Chiralpak AD-3) *i*-PrOH/hexane = 1/99, 1.0 mL/min, T = 28 °C, λ = 280 nm, t_r (major) = 7.8 min, t_r (minor) = 9.1 min, 97% *ee*. $[\alpha]_D^{29.6}$ = +23 (c = 0.1, $CHCl_3$).

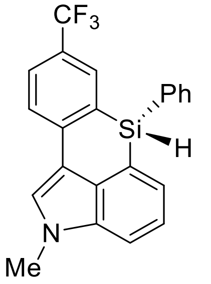

(*S*)-2-Methyl-6-phenyl-8-(trifluoromethyl)-2,6-dihydrobenzo[5,6]silino[4,3,2-*cd*]indole (2e)

The product **2e** was purified via silica gel column chromatography (petroleum ether/ethyl acetate = 10/1, v/v) as a white solid (26.5 mg, 70% yield). ^1H NMR (400 MHz, $CDCl_3$): δ = 7.82 (s, 1H), 7.79 (d, *J* = 8.3 Hz, 1H), 7.63 – 7.54 (m, 4H), 7.47 (d, *J* = 6.7 Hz, 1H), 7.42 – 7.31 (m, 5H), 5.74 (s, 1H), 3.83 (s, 3H) ppm. ^{13}C NMR (150 MHz, $CDCl_3$): δ = 142.9, 135.6, 135.4,

134.8, 133.2 (q, $J_{C\text{-}F}$ = 3.9 Hz), 131.7, 130.0, 128.2, 127.7, 127.3, 126.7, 126.6 (q, $J_{C\text{-}F}$ = 3.8 Hz), 126.2 (q, $J_{C\text{-}F}$ = 32.1 Hz), 124.5 (q, $J_{C\text{-}F}$ = 269.9 Hz), 123.1, 122.4, 121.7, 114.7, 111.0, 33.2 ppm. ^{19}F NMR (565 MHz, $CDCl_3$): δ = -62.20 ppm. HRMS (ESI): calcd for $C_{22}H_{17}F_3NSi$ $[M+H]^+$ 380.1077, found 380.1082. HPLC (Chiralpak AD-3) *i*-PrOH/hexane = 5/95, 1.0 mL/min, T = 28 °C, λ = 250 nm, t_r (major) = 7.3 min, t_r (minor) = 8.6 min, 92% *ee*. $[\alpha]_D^{29.7}$ = +196 (c = 0.1, $CHCl_3$).

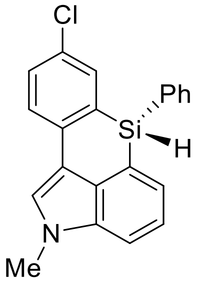

(*S*)-8-Chloro-2-methyl-6-phenyl-2,6-dihydrobenzo[5,6]silino[4,3,2-*cd*]indole (2f)

L5 (1.8 mg, 3 mol%) was used as ligand, the product **2f** was purified via silica gel column chromatography (petroleum ether/ethyl acetate = 40/1, v/v) as a white solid (25.4 mg, 74% yield). ^{1}H NMR (600 MHz, $CDCl_3$): δ = 7.57 (d, *J* = 8.5 Hz, 1H), 7.51 (d, *J* = 7.9 Hz, 2H), 7.45 (d, *J* = 2.1 Hz, 1H), 7.42 (s, 1H), 7.36 (d, *J* = 6.8 Hz, 1H), 7.32 – 7.28 (m, 2H), 7.28 – 7.20 (m, 4H), 5.61 (s, 1H), 3.72 (s, 3H) ppm. ^{13}C NMR (150 MHz, $CDCl_3$): δ = 137.8, 135.8, 135.5, 135.4, 135.0, 131.6, 130.2, 130.0, 129.9, 129.4, 128.1, 126.9, 125.7, 123.9, 122.9, 121.6, 114.8, 110.9, 33.0 ppm. HRMS (ESI): calcd for $C_{21}H_{17}ClNSi$ $[M+H]^+$ 346.0813, found 346.0819. HPLC (Chiralpak OD-3) *i*-PrOH/hexane = 5/95, 1.0 mL/min, T = 28 °C, λ = 250 nm, t_r (major) = 9.0 min, t_r (minor) = 9.5 min, 92% *ee*. $[\alpha]_D^{29.7}$ = +77 (c = 0.1, $CHCl_3$).

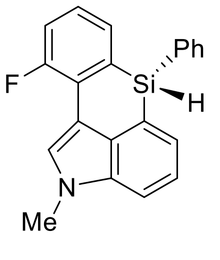

(*R*)-10-Fluoro-2-methyl-6-phenyl-2,6-dihydrobenzo[5,6]silino[4,3,2-*cd*]indole (2g)

The product **2g** was purified via silica gel column chromatography (petroleum ether/ethyl acetate = 40/1, v/v) as a white solid (26.2 mg, 80% yield). ^{1}H NMR (400 MHz, $CDCl_3$): δ =

7.84 (d, J = 3.4 Hz, 1H), 7.62 – 7.57 (m, 2H), 7.48 – 7.30 (m, 7H), 7.18 – 7.06 (m, 2H), 5.73 (s, 1H), 3.86 (s, 3H) ppm. ^{13}C NMR (100 MHz, $CDCl_3$): δ = 159.6 (d, J_{C-F} = 248.9 Hz), 135.4, 134.7, 132.2 (d, J_{C-F} = 3.3 Hz), 132.0, 130.6, 130.4, 130.2 (d, J_{C-F} = 2.0 Hz), 129.8, 128.0, 127.4 (d, J_{C-F} = 11.2 Hz), 126.9, 125.2 (d, J_{C-F} = 7.8 Hz), 122.7, 121.7, 116.9 (d, J_{C-F} = 22.3 Hz), 110.8, 110.1, 33.0 ppm. ^{19}F NMR (565 MHz, $CDCl_3$): δ = -111.36 ppm. HRMS (ESI): calcd for $C_{21}H_{17}FNSi$ $[M+H]^+$ 330.1109, found 330.1106. HPLC (Chiralpak OD-3) *i*-PrOH/hexane = 5/95, 1.0 mL/min, T = 28 °C, λ = 220 nm, t_r (major) = 5.5 min, t_r (minor) = 6.4 min, 92% *ee*. $[\alpha]_D^{29.5}$ = +152 (c = 0.1, $CHCl_3$).

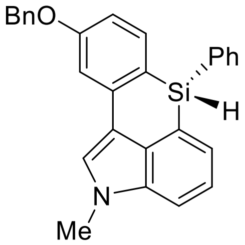

(*S*)-9-(Benzyloxy)-2-methyl-6-phenyl-2,6-dihydrobenzo[5,6]silino[4,3,2-*cd*]indole (2h)

The product **2h** was purified via silica gel column chromatography (petroleum ether/ethyl acetate = 40/1, v/v) as a white solid (34.2 mg, 82% yield). ^{1}H NMR (400 MHz, $CDCl_3$): δ = 7.61 (dd, J = 7.7, 1.6 Hz, 2H), 7.52 (t, J = 4.1 Hz, 2H), 7.45 (t, J = 7.5 Hz, 3H), 7.42 – 7.28 (m, 9H), 6.80 (dd, J = 8.2, 2.4 Hz, 1H), 5.68 (s, 1H), 5.12 (s, 2H), 3.82 (s, 3H) ppm. ^{13}C NMR (100 MHz, $CDCl_3$): δ = 160.4, 141.2, 138.0, 137.0, 136.1, 135.5, 135.4, 131.9, 129.5, 128.6, 128.0, 127.9, 127.6, 126.8, 125.6, 122.7, 118.7, 115.6, 111.7, 110.6, 108.9, 69.8, 33.0 ppm. HRMS (ESI): calcd for $C_{28}H_{24}NOSi$ $[M+H]^+$ 418.1622, found 418.1615. HPLC (Chiralpak OD-3) *i*-PrOH/hexane = 15/85, 1.0 mL/min, T = 28 °C, λ = 250 nm, t_r (minor) = 21.0 min, t_r (major) = 26.8 min, 97% *ee*. $[\alpha]_D^{29.3}$ = +98 (c = 0.1, $CHCl_3$).

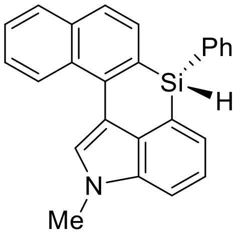

(*S*)-2-Methyl-6-phenyl-2,6-dihydronaphtho[1',2':5,6]silino[4,3,2-*cd*]indole (2i)

The product **2i** was purified via silica gel column chromatography (petroleum ether/ethyl

acetate = 40/1, v/v) as a pale yellow solid (10.9 mg, 30% yield). ^{1}H NMR (400 MHz, $CDCl_3$): δ = 8.77 (d, J = 8.6 Hz, 1H), 8.06 (s, 1H), 7.84 (d, J = 7.9 Hz, 1H), 7.69 – 7.55 (m, 5H), 7.52 (t, J = 7.1 Hz, 2H), 7.43 (d, J = 7.5 Hz, 1H), 7.40 – 7.28 (m, 4H), 5.82 (s, 1H), 3.91 (s, 3H) ppm. ^{13}C NMR (100 MHz, $CDCl_3$): δ = 138.1, 136.0, 135.5, 135.4, 134.8, 133.1, 132.1, 130.4, 130.3, 129.7, 129.0, 128.0, 126.9, 126.0, 126.0, 125.9, 124.4, 123.0, 122.7, 114.8, 110.6, 33.1 ppm. HRMS (ESI): calcd for $C_{25}H_{20}NSi$ $[M+H]^+$ 362.1360, found 362.1355. HPLC (Chiralpak OD-3) *i*-PrOH/hexane = 5/95, 1.0 mL/min, T = 28 °C, λ = 250 nm, t_r (major) = 12.1 min, t_r (minor) = 21.7 min, 87% *ee*. $[\alpha]_D^{29.4}$ = +120 (c = 0.1, $CHCl_3$).

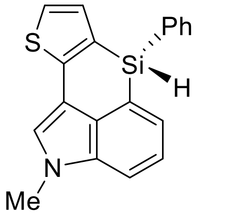

(*R*)-2-Methyl-6-phenyl-2,6-dihydrothieno[2',3':5,6]silino[4,3,2-*cd*]indole (2j)

The product **2j** was purified via silica gel column chromatography (petroleum ether/ethyl acetate = 80/1, v/v) as a dark green solid (29.0 mg, 91% yield). ^{1}H NMR (600 MHz, $CDCl_3$): δ = 7.61 – 7.59 (m, 2H), 7.49 – 7.48 (m, 1H), 7.36 – 7.30 (m, 6H), 7.16 (d, J = 4.9 Hz, 1H), 7.14 (d, J = 5.0 Hz, 1H), 5.77 (s, 1H), 3.74 (s, 3H) ppm. ^{13}C NMR (150 MHz, $CDCl_3$): δ = 145.5, 135.5, 135.2, 135.1, 132.2, 131.3, 129.6, 128.0, 127.5, 125.7, 124.9, 123.1, 122.2, 121.1, 112.0, 110.7, 32.8 ppm. HRMS (ESI): calcd for $C_{19}H_{16}NSSi$ $[M+H]^+$ 318.0767, found 318.0763. HPLC (Chiralpak OD-3) *i*-PrOH/hexane = 5/95, 1.0 mL/min, T = 28 °C, λ = 250 nm, t_r (major) = 11.7 min, t_r (minor) = 12.5 min, 91% *ee*. $[\alpha]_D^{25.0}$ = +242 (c = 0.1, $CHCl_3$).

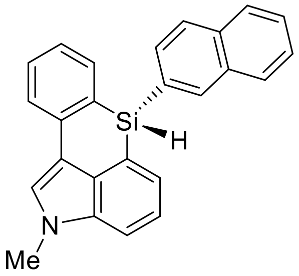

(*S*)-2-Methyl-6-(naphthalen-2-yl)-2,6-dihydrobenzo[5,6]silino[4,3,2-*cd*]indole (2k)

The product **2k** was purified via silica gel column chromatography (petroleum ether/ethyl

acetate = 40/1, v/v) as a white solid (27.1 mg, 75% yield). ^{1}H NMR (600 MHz, $CDCl_3$): δ = 8.18 (s, 1H), 7.82 – 7.73 (m, 4H), 7.62 (d, J = 7.6 Hz, 2H), 7.57 (s, 1H), 7.49 – 7.41 (m, 3H), 7.41 – 7.34 (m, 2H), 7.31 (t, J = 6.9 Hz, 1H), 7.11 (t, J = 7.0 Hz, 1H), 5.84 (s, 1H), 3.82 (s, 3H) ppm. ^{13}C NMR (150 MHz, $CDCl_3$): δ = 139.6, 136.7, 136.6, 135.5, 134.0, 133.2, 133.0, 131.8, 131.2, 130.0, 128.2, 127.7, 127.3, 127.0, 126.8, 126.6, 125.9, 125.6, 124.7, 122.7, 122.5, 122.4, 115.7, 110.7, 33.0 ppm. HRMS (ESI): calcd for $C_{25}H_{20}NSi$ $[M+H]^+$ 362.1360, found 362.1355. HPLC (Chiralpak OD-3) *i*-PrOH/hexane = 5/95, 1.0 mL/min, T = 28 °C, λ = 254 nm, t_r (major) = 13.5 min, t_r (minor) = 21.6 min, 96% *ee*. $[\alpha]_D^{25.0}$ = +133 (c = 0.1, $CHCl_3$).

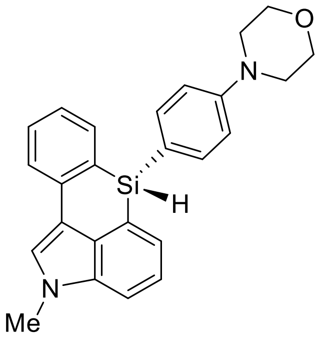

(*S*)-4-(4-(2-Methyl-2,6-dihydrobenzo[5,6]silino[4,3,2-*cd*]indol-6-yl)phenyl)morpholine (2l)

The product **2l** was purified via silica gel column chromatography (petroleum ether/ethyl acetate = 40/1, v/v) as a white solid (30.9 mg, 78% yield). ^{1}H NMR (600 MHz, $CDCl_3$): δ = 7.76 (d, J = 7.9 Hz, 1H), 7.59 (d, J = 7.2 Hz, 1H), 7.57 (s, 1H), 7.52 (d, J = 8.5 Hz, 2H), 7.43 (d, J = 6.8 Hz, 1H), 7.36 (t, J = 7.6 Hz, 2H), 7.32 – 7.30 (m, 1H), 7.12 (t, J = 7.3 Hz, 1H), 6.87 (d, J = 8.4 Hz, 2H), 5.67 (s, 1H), 3.84 (s, 3H), 3.83 – 3.81 (m, 4H), 3.17 – 3.15 (m, 4H) ppm. ^{13}C NMR (150 MHz, $CDCl_3$): δ = 139.5, 136.7, 136.6, 135.5, 131.8, 129.7, 127.7, 126.7, 125.4, 124.6, 123.1, 122.7, 122.4, 115.8, 114.8, 110.5, 66.8, 48.5, 33.0 ppm. HRMS (ESI): calcd for $C_{25}H_{25}N_2OSi$ $[M+H]^+$ 397.1731, found 397.1727. HPLC (Chiralpak AD-3) *i*-PrOH/hexane = 50/50, 1.0 mL/min, T = 28 °C, λ = 250 nm, t_r (major) = 8.7 min, t_r (minor) = 51.5 min, 93% *ee*. $[\alpha]_D^{25.0}$ = +77 (c = 0.1, $CHCl_3$).

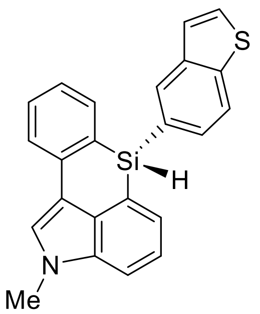

(*S*)-6-(Benzo[*b*]thiophen-5-yl)-2-methyl-2,6-dihydrobenzo[5,6]silino[4,3,2-*cd*]indole (2m)

The product **2m** was purified via silica gel column chromatography (petroleum ether/ethyl acetate = 80/1, v/v) as a pale yellow solid (8.8 mg, 24% yield). ^{1}H NMR (600 MHz, $CDCl_3$): δ = 7.87 (s, 1H), 7.78 (d, J = 7.9 Hz, 1H), 7.62 – 7.59 (m, 2H), 7.58 (d, J = 2.1 Hz, 1H), 7.55 (d, J = 8.2 Hz, 1H), 7.48 (d, J = 8.2 Hz, 1H), 7.44 (d, J = 6.8 Hz, 1H), 7.40 – 7.37 (m, 2H), 7.33 – 7.31 (m, 1H), 7.12 (t, J = 7.2 Hz, 1H), 6.70 (d, J = 1.6 Hz, 1H), 5.79 (s, 1H), 3.86 (s, 3H) ppm. ^{13}C NMR (150 MHz, $CDCl_3$): δ = 156.1, 144.8, 139.5, 136.7, 135.5, 131.8, 131.2, 129.9, 129.4, 129.0, 127.5, 126.8, 125.5, 124.7, 122.9, 122.7, 122.5, 115.7, 111.3, 110.6, 106.5, 33.0 ppm. HRMS (ESI): calcd for $C_{23}H_{18}NSSi$ $[M+H]^+$ 368.0924, found 368.0922. HPLC (Chiralpak OD-3) *i*-PrOH/hexane = 5/95, 1.0 mL/min, T = 28 ^{o}C, λ = 254 nm, t_r (major) = 14.9 min, t_r (minor) = 22.2 min, 90% *ee*. $[\alpha]_D^{25.0}$ = +176 (c = 0.1, $CHCl_3$).

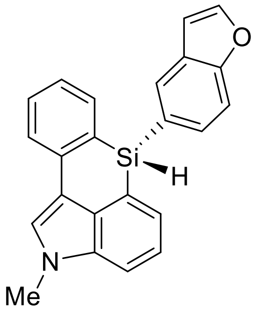

(*S*)-6-(Benzofuran-5-yl)-2-methyl-2,6-dihydrobenzo[5,6]silino[4,3,2-*cd*]indole (2n)

The product **2n** was purified via silica gel column chromatography (petroleum ether/ethyl acetate = 80/1, v/v) as a pale yellow solid (17.6 mg, 50% yield). ^{1}H NMR (600 MHz, $CDCl_3$): δ = 8.09 (s, 1H), 7.85 (d, J = 8.0 Hz, 1H), 7.78 (d, J = 7.9 Hz, 1H), 7.62 – 7.60 (m, 2H), 7.55 (d, J = 8.0 Hz, 1H), 7.45 (d, J = 6.8 Hz, 1H), 7.40 – 7.37 (m, 3H), 7.33 – 7.31 (m, 1H), 7.28 (d, J = 5.4 Hz, 1H), 7.12 (t, J = 7.3 Hz, 1H), 5.81 (s, 1H), 3.86 (s, 3H) ppm. ^{13}C NMR (150 MHz, $CDCl_3$): δ = 141.3, 139.5, 139.4, 136.7, 135.5, 131.8, 131.3, 131.0, 130.5, 130.0, 127.2, 126.8,

126.1, 125.6, 124.7, 123.9, 122.7, 122.6, 122.5, 122.2, 115.7, 110.7, 33.0 ppm. HRMS (ESI): calcd for $C_{23}H_{18}NOSi$ $[M+H]^+$ 352.1152, found 352.1150. HPLC (Chiralpak OD-3) *i*-PrOH/hexane = 5/95, 1.0 mL/min, T = 28 °C, λ = 254 nm, t_r (major) = 14.6 min, t_r (minor) = 23.4 min, 94% *ee*. $[\alpha]_D^{25.0}$ = +81 (c = 0.1, $CHCl_3$).

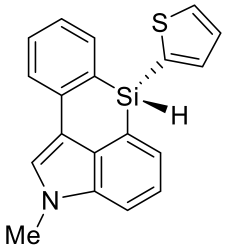

(*S*)-2-Methyl-6-(thiophen-2-yl)-2,6-dihydrobenzo[5,6]silino[4,3,2-*cd*]indole (2o)

The product **2o** was purified via silica gel column chromatography (petroleum ether/ethyl acetate = 80/1, v/v) as a white solid (19.7 mg, 62% yield). ^1H NMR (600 MHz, $CDCl_3$): δ = 7.76 (d, J = 7.9 Hz, 1H), 7.69 (d, J = 7.3 Hz, 1H), 7.65 (d, J = 4.6 Hz, 1H), 7.58 (s, 1H), 7.52 (d, J = 6.8 Hz, 1H), 7.40 (t, J = 8.0 Hz, 3H), 7.36 – 7.34 (m, 1H), 7.19 – 7.18 (m, 1H), 7.16 (d, J = 7.3 Hz, 1H), 5.85 (s, 1H), 3.85 (s, 3H) ppm. ^{13}C NMR (150 MHz, $CDCl_3$): δ = 139.4, 137.1, 136.6, 135.5, 134.3, 132.3, 131.7, 130.1, 128.4, 126.8, 126.5, 125.7, 124.8, 122.7, 122.5, 121.8, 115.5, 110.9, 33.0 ppm. HRMS (ESI): calcd for $C_{19}H_{16}NSSi$ $[M+H]^+$ 318.0767, found 318.0765. HPLC (Chiralpak OD-3) *i*-PrOH/hexane = 5/95, 1.0 mL/min, T = 28 °C, λ = 254 nm, t_r (major) = 13.1 min, t_r (minor) = 17.3 min, 90% *ee*. $[\alpha]_D^{25.0}$ = +156 (c = 0.1, $CHCl_3$).

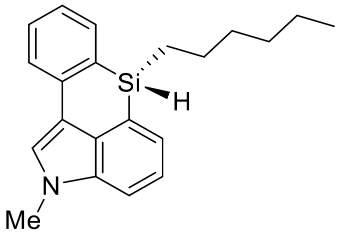

(*S*)-6-Hexyl-2-methyl-2,6-dihydrobenzo[5,6]silino[4,3,2-*cd*]indole (2p)

The product **2p** was purified via silica gel column chromatography (petroleum ether/ethyl acetate = 80/1, v/v) as a colorless oil (25.6 mg, 80% yield). ^1H NMR (400 MHz, $CDCl_3$): δ = 7.72 (d, J = 7.9 Hz, 1H), 7.62 (dd, J = 7.2, 0.6 Hz, 1H), 7.50 (s, 1H), 7.46 (dd, J = 5.7, 2.1 Hz, 1H), 7.40 – 7.29 (m, 3H), 7.16 (td, J = 7.3, 0.9 Hz, 1H), 5.16 (t, J = 2.7 Hz, 1H), 3.79 (s, 3H),

1.50 – 1.40 (m, 2H), 1.35 – 1.27 (m, 2H), 1.25 – 1.16 (m, 4H), 1.12 – 1.00 (m, 2H), 0.82 (t, *J* = 6.9 Hz, 3H) ppm. ^{13}C NMR (100 MHz, $CDCl_3$): δ = 139.5, 135.7, 135.4, 131.8, 129.6, 128.2, 126.0, 125.1, 124.5, 123.6, 122.5, 122.4, 115.9, 110.2, 32.9, 32.9, 31.4, 24.0, 22.5, 14.7, 14.1. ppm. HRMS (ESI): calcd for $C_{21}H_{26}NSi$ $[M+H]^+$ 320.1829, found 320.1830. HPLC (Chiralpak OD-3) *i*-PrOH/hexane = 5/95, 1.0 mL/min, T = 28 °C, λ = 250 nm, t_r (major) = 9.9 min, t_r (minor) = 11.3 min, 98% *ee*. $[\alpha]_D^{24.0}$ = +82 (c = 0.1, $CHCl_3$).

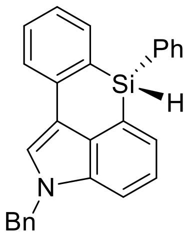

(*S*)-2-Benzyl-6-phenyl-2,6-dihydrobenzo[5,6]silino[4,3,2-*cd*]indole (2q)

The product **2q** was purified via silica gel column chromatography (petroleum ether/ethyl acetate = 80/1, v/v) as a white solid (28.5 mg, 74% yield). ^{1}H NMR (400 MHz, $CDCl_3$): δ = 7.75 (d, *J* = 7.9 Hz, 1H), 7.67 – 7.58 (m, 4H), 7.45 (d, *J* = 6.7 Hz, 1H), 7.40 – 7.23 (m, 9H), 7.20 – 7.11 (m, 3H), 5.72 (s, 1H), 5.34 (s, 2H) ppm. ^{13}C NMR (100 MHz, $CDCl_3$): δ = 139.4, 137.1, 136.6, 135.6, 135.5, 135.2, 132.0, 129.9, 129.6, 128.9, 128.0, 127.8, 127.1, 127.0, 126.9, 124.8, 122.9, 122.6, 122.6, 116.3, 111.1, 50.3 ppm. HRMS (ESI): calcd for $C_{27}H_{22}NSi$ $[M+H]^+$ 388.1516, found 388.1513. HPLC (Chiralpak OD-3) *i*-PrOH/hexane = 5/95, 1.0 mL/min, T = 28 °C, λ = 250 nm, t_r (major) = 16.7 min, t_r (minor) = 27.5 min, 91% *ee*. $[\alpha]_D^{29.3}$ = +72 (c = 0.1, $CHCl_3$).

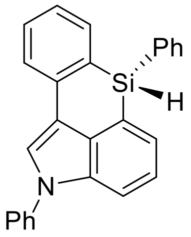

(*S*)-2,6-Diphenyl-2,6-dihydrobenzo[5,6]silino[4,3,2-*cd*]indole (2r)

The product **2r** was purified via silica gel column chromatography (petroleum ether) as a white solid (26.8 mg, 72% yield). ^{1}H NMR (400 MHz, $CDCl_3$): δ = 7.88 (s, 1H), 7.86 (d, *J* = 7.9 Hz, 1H), 7.69 – 7.60 (m, 4H), 7.59 – 7.49 (m, 5H), 7.44 – 7.29 (m, 6H), 7.18 (td, *J* = 7.4, 1.0 Hz,

1H), 5.74 (s, 1H) ppm. ^{13}C NMR (100 MHz, $CDCl_3$): δ = 139.5, 139.0, 136.6, 135.5, 134.7, 132.6, 130.0, 129.7, 129.7, 128.0, 127.8, 127.4, 126.7, 125.2, 124.4, 124.1, 123.5, 122.9, 122.9, 117.8, 112.0 ppm. HRMS (ESI): calcd for $C_{26}H_{20}NSi$ $[M+H]^+$ 374.1360, found 374.1356. HPLC (Chiralpak OD-3) *i*-PrOH/hexane = 5/95, 1.0 mL/min, T = 28 °C, λ = 250 nm, t_r (major) = 6.7 min, t_r (minor) = 7.3 min, 95% *ee*. $[\alpha]_D^{29.6}$ = +90 (c = 0.1, $CHCl_3$).

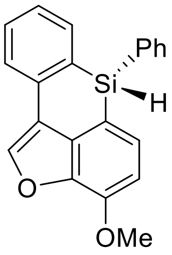

(*S*)-3-Methoxy-6-phenyl-6*H*-benzo[5,6]silino[4,3,2-*cd*]benzofuran (2s)

The product **2s** was purified via silica gel column chromatography (petroleum ether/ethyl acetate = 80/1, v/v) as a white solid (19.8 mg, 60% yield). ^{1}H NMR (400 MHz, $CDCl_3$): δ = 8.16 (s, 1H), 7.83 (d, *J* = 7.9 Hz, 1H), 7.66 (d, *J* = 7.1 Hz, 1H), 7.59 (dd, *J* = 7.8, 1.5 Hz, 2H), 7.50 (d, *J* = 7.8 Hz, 1H), 7.44 (td, *J* = 7.8, 1.3 Hz, 1H), 7.41 – 7.31 (m, 3H), 7.27 (dd, *J* = 7.4, 0.9 Hz, 1H). 6.95 (d, *J* = 7.8 Hz, 1H), 5.65 (s, 1H), 4.06 (s, 3H) ppm. ^{13}C NMR (100 MHz, $CDCl_3$): δ = 146.7, 142.9, 141.0, 136.7, 136.1, 135.4, 135.1, 133.9, 131.2, 130.0, 129.9, 129.4, 128.1, 126.8, 124.3, 121.4, 114.2, 108.6, 56.2 ppm. HRMS (ESI): calcd for $C_{21}H_{17}O_2Si$ $[M+H]^+$ 329.0992, found 329.0989. HPLC (Chiralpak OD-3) *i*-PrOH/hexane = 1/99, 1.0 mL/min, T = 28 °C, λ = 250 nm, t_r (minor) = 11.5 min, t_r (major) = 13.5 min, 95% *ee*. $[\alpha]_D^{29.6}$ = +112 (c = 0.1, $CHCl_3$).

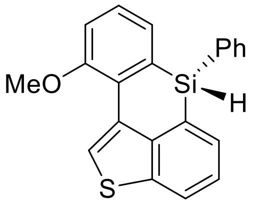

(*S*)-10-Methoxy-6-phenyl-6*H*-2-thia-6-silaaceanthrylene (2t)

$[Rh(cod)Cl]_2$ (2.0 mg, 4 mol%), **L1** (4.5 mg, 8 mol%) was used, the product **2t** was purified via silica gel column chromatography (petroleum ether) as a colorless oil (18.3 mg, 53% yield). ^{1}H NMR (400 MHz, $CDCl_3$): δ = 8.99 (s, 1H), 7.98 (dd, *J* = 8.0, 0.7 Hz, 1H), 7.66 (dd, *J* = 6.9, 0.5

Hz, 1H), 7.61 – 7.56 (m, 2H), 7.41 – 7.26 (m, 6H), 7.12 (dd, J = 7.1, 2.4 Hz, 1H), 5.67 (s, 1H), 4.04 (s, 3H) ppm. ^{13}C NMR (100 MHz, $CDCl_3$): δ = 157.6, 143.3, 138.0, 135.5, 135.2, 131.4, 131.1, 130.3, 129.9, 129.3, 128.6, 128.4, 128.1, 127.2, 125.8, 124.6, 123.9, 113.4, 55.4 ppm. HRMS (ESI): calcd for $C_{21}H_{17}OSSi$ $[M+H]^+$ 345.0764, found 345.0762. HPLC (Chiralpak OD-3) *i*-PrOH/hexane = 1/99, 1.0 mL/min, T = 28 °C, λ = 250 nm, t_r (minor) = 6.8 min, t_r (major) = 7.7 min, 94% *ee*. $[\alpha]_D^{29.5}$ = +87 (c = 0.1, $CHCl_3$).

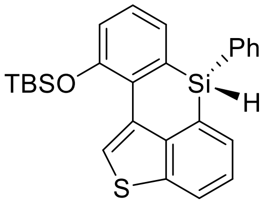

(*S*)-10-((*tert*-Butyldimethylsilyl)oxy)-6-phenyl-6*H*-2-thia-6-silaaceanthrylene (2u)

The product **2u** was purified via silica gel column chromatography (petroleum ether) as a colorless oil (26.9 mg, 61% yield). ^{1}H NMR (400 MHz, $CDCl_3$): δ = 8.93 (s, 1H), 7.98 (d, J = 8.0 Hz, 1H), 7.66 (d, J = 6.9 Hz, 1H), 7.58 (dd, J = 7.8, 1.4 Hz, 2H), 7.42 – 7.31 (m, 4H), 7.28 (d, J = 7.1 Hz, 1H), 7.15 (t, J = 7.6 Hz, 1H), 7.05 (dd, J = 8.0, 1.1 Hz, 1H), 5.66 (s, 1H), 1.05 (s, 9H), 0.35 (s, 6H) ppm. ^{13}C NMR (100 MHz, $CDCl_3$): δ = 153.8, 143.2, 138.0, 135.5, 135.2, 131.4, 131.4, 130.4, 130.2, 129.9, 129.4, 128.7, 128.1, 126.8, 125.9, 124.5, 123.9, 122.3, 26.2, 18.8, -3.4, -3.4 ppm. HRMS (ESI): calcd for $C_{26}H_{31}OSSi_2$ $[M+H]^+$ 445.1472, found 445.1469. HPLC (Chiralpak IB) *i*-PrOH/hexane = 0/100, 1.0 mL/min, T = 28 °C, λ = 250 nm, t_r (minor) = 8.6 min, t_r (major) = 15.1 min, 95% *ee*. $[\alpha]_D^{29.4}$ = +96 (c = 0.1, $CHCl_3$).

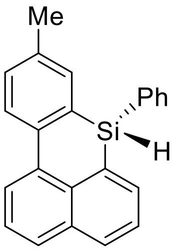

(*S*)-9-Methyl-7-phenyl-7*H*-benzo[*e*]naphtho[1,8-*bc*]siline (2v)

The reaction was performed with $[Rh(cod)Cl]_2$ (1.0 mg, 2 mol%), **L1** (2.2 mg, 4 mol%), and toluene (1 mL) at 60 °C for 8 h. The product **2v** was purified via silica gel column chromatography (petroleum ether) as a colorless oil (24.6 mg, 76% yield). ^{1}H NMR (400 MHz,

CDCl3): δ = 8.32 (d, J = 7.6 Hz, 1H), 8.11 (d, J = 8.4 Hz, 1H), 7.94 (d, J = 8.1 Hz, 1H), 7.83 (t, J = 7.4 Hz, 2H), 7.63 – 7.54 (m, 3H), 7.52 – 7.44 (m, 2H), 7.42 – 7.31 (m, 4H), 5.59 (s, 1H), 2.35 (s, 3H) ppm. ^{13}C NMR (100 MHz, $CDCl_3$): δ = 140.7, 136.5, 136.4, 135.7, 135.6, 135.0, 134.4, 134.3, 134.0, 131.7, 131.3, 129.9, 129.5, 129.0, 128.1, 127.6, 126.4, 125.9, 125.0, 124.7, 20.9 ppm. HRMS (ESI): calcd for $C_{23}H_{19}Si$ $[M+H]^+$ 323.1251, found 323.1249. HPLC (Chiralpak OD-3) *i*-PrOH/hexane = 1/99, 1.0 mL/min, T = 28 °C, λ = 250 nm, t_r (minor) = 5.6 min, t_r (major) = 8.0 min, 97% *ee*. $[\alpha]_D^{29.6}$ = +30 (c = 0.1, $CHCl_3$).

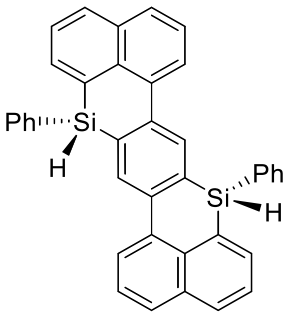

The reaction was performed with **1w** (27.1 mg, 0.05 mmol), $[Rh(cod)Cl]_2$ (0.5 mg, 2 mol%), **L1** (1.6 mg, 6 mol%), and toluene (1 mL) at 70 °C for 12 h. The product **2w** was purified via silica gel column chromatography (petroleum ether) as a pale yellow solid (11.2 mg, 42% yield). ^{1}H NMR (600 MHz, $CDCl_3$): δ = 8.46 (s, 2H), 8.30 (d, J = 7.4 Hz, 2H), 7.96 (d, J = 8.1 Hz, 2H), 7.86 (d, J = 8.1 Hz, 4H), 7.67 (d, J = 7.1 Hz, 4H), 7.57 (t, J = 7.7 Hz, 2H), 7.52 (t, J = 7.2 Hz, 2H), 7.43 – 7.41 (m, 2H), 7.38 (t, J = 7.2 Hz, 4H), 5.71 (s, 2H) ppm. ^{13}C NMR (150 MHz, $CDCl_3$): δ = 141.4, 135.9, 135.7, 134.5, 134.0, 133.9, 133.7, 131.6, 131.4, 130.1, 130.0, 128.2, 127.3, 126.0, 125.4, 125.2 ppm. HRMS (EI): calcd for $C_{38}H_{26}Si_2$ $[M]^+$ 538.1573, found 538.1574. HPLC (Chiralpak OD-3) *i*-PrOH/hexane = 1/99, 1.0 mL/min, T = 28 °C, λ = 250 nm, t_r (major) = 7.5 min, t_r (minor) = 8.2 min, 99% *ee*. $[\alpha]_D^{25.0}$ = -172 (c = 0.1, $CHCl_3$).

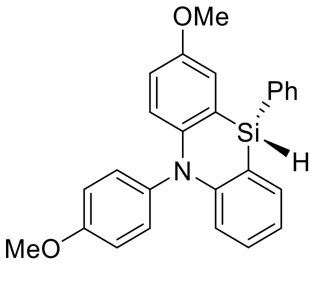

(*R*)-2-Methoxy-5-(4-methoxyphenyl)-10-phenyl-5,10-dihydrodibenzo[*b*,*e*][1,4]azasiline

(2x)

L7 (1.8 mg, 3 mol%) was used as ligand, the product **2x** was purified via silica gel column chromatography (petroleum ether) as a white solid (18.3 mg, 45% yield). ^{1}H NMR (600 MHz, $CDCl_3$): δ = 7.67 (d, J = 6.5 Hz, 2H), 7.49 (d, J = 7.2 Hz, 1H), 7.44 – 7.37 (m, 3H), 7.21 (d, J = 8.7 Hz, 2H), 7.11 – 7.17 (m, 3H), 7.00 (d, J = 3.0 Hz, 1H), 6.86 (t, J = 7.1 Hz, 1H), 6.76 (dd, J = 9.3, 3.0 Hz, 1H), 6.41 (t, J = 6.6 Hz, 2H), 5.69 (s, 1H), 3.92 (s, 3H), 3.71 (s, 3H) ppm. ^{13}C NMR (150 MHz, $CDCl_3$): δ = 159.0, 152.7, 150.2, 144.6, 136.3, 136.1, 135.6, 135.4, 132.2, 130.5, 129.9, 128.1, 119.2, 118.9, 118.8, 117.6, 116.9, 116.1, 114.3, 112.1, 55.6, 55.5 ppm. HRMS (ESI): calcd for $C_{26}H_{24}NO_2Si$ $[M+H]^+$ 410.1571, found 410.1566. HPLC (Chiralpak OD-H) *i*-PrOH/hexane = 0.5/99.5, 1.0 mL/min, T = 28 °C, λ = 250 nm, t_r (major) = 15.9 min, t_r (minor) = 21.9 min, 58% *ee*. $[\alpha]_D^{29.9}$ = -23 (c = 0.1, $CHCl_3$).

2. Scope of Seven-Membered Silicon-Stereogenic Heterocycles

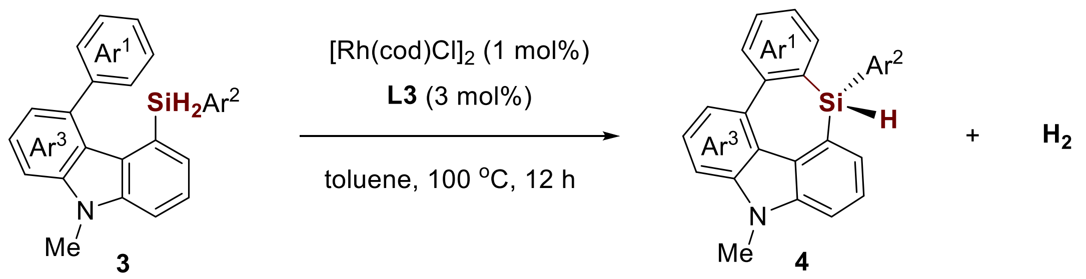

A 5 mL microwave tube was charged with **3** (0.1 mmol, 1 equiv), $[Rh(cod)Cl]_2$ (0.5 mg, 1 mol%), **L3** (1.6 mg, 3 mol%), and toluene (1 mL) in glovebox. The tube was sealed, then removed from the glovebox, and the mixture was stirred at 100 °C for 12 h. After the completion of the reaction, the solvent was evaporated under reduced pressure and the residue was purified by flash column chromatography on silica gel to give the product.

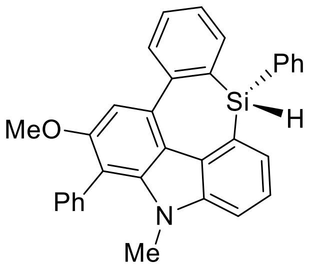

(*S*)-11-Methoxy-1-methyl-5,12-diphenyl-1,5-dihydrobenzo[6,7]silepino[2,3,4,5-

***def*]carbazole (4a)**

The product **4a** was purified via silica gel column chromatography ((petroleum ether/ethyl acetate = 80/1, v/v) as a white solid (36.3 mg, 78% yield). ^{1}H NMR (400 MHz, $CDCl_3$): δ = 7.89 (dd, J = 7.3, 1.4 Hz, 1H), 7.80 (d, J = 8.0 Hz, 1H), 7.55 (td, J = 7.7, 1.6 Hz, 1H), 7.51 – 7.39 (m, 10H), 7.34 (s, 1H), 7.32 – 7.27 (m, 2H), 7.25 – 7.22 (m, 2H), 5.62 (s, 1H), 3.90 (s, 3H), 3.19 (s, 3H) ppm. ^{13}C NMR (100 MHz, $CDCl_3$): δ = 155.7, 145.8, 141.3, 141.0, 137.4, 136.8, 135.7, 135.3, 133.6, 133.2, 132.5, 131.5, 131.4, 130.2, 129.6, 127.9, 127.9, 127.4, 127.2, 127.1, 125.3, 125.1, 116.9, 113.4, 109.4, 106.4, 56.7, 32.6 ppm. HRMS (ESI): calcd for $C_{32}H_{26}NOSi$ $[M+H]^+$ 468.1778, found 468.1767. HPLC (Chiralpak AD-3) *i*-PrOH/hexane = 5/95, 1.0 mL/min, T = 28 °C, λ = 250 nm, t_r (major) = 7.3 min, t_r (minor) = 9.2 min, 90% *ee*. $[\alpha]_D^{29.6}$ = +71 (c = 0.1, $CHCl_3$).

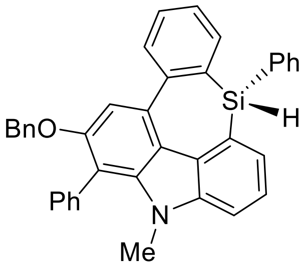

(*S*)-11-(Benzyloxy)-1-methyl-5,12-diphenyl-1,5-dihydrobenzo[6,7]silepino[2,3,4,5-*def*]carbazole (4b)

The product **4b** was purified via silica gel column chromatography ((petroleum ether/ethyl acetate = 80/1, v/v) as a white solid (29.6 mg, 55% yield). ^{1}H NMR (400 MHz, $CDCl_3$): δ = 7.88 (dd, J = 7.3, 1.2 Hz, 1H), 7.60 – 7.38 (m, 12H), 7.37 (s, 1H), 7.34 – 7.24 (m, 5H), 7.24 – 7.16 (m, 4H), 5.62 (s, 1H), 5.21 – 5.13 (m, 2H), 3.23 (s, 3H) ppm. ^{13}C NMR (150 MHz, $CDCl_3$): δ = 154.5, 145.7, 141.3, 140.8, 137.6, 137.4, 136.7, 135.7, 135.3, 133.6, 133.1, 132.5, 131.6, 131.5, 130.2, 129.6, 128.3, 127.9, 127.8, 127.8, 127.5, 127.4, 127.3, 127.0, 126.9, 125.3, 125.1, 117.2, 114.6, 109.4, 109.1, 71.5, 32.6 ppm. HRMS (ESI): calcd for $C_{38}H_{30}NOSi$ $[M+H]^+$ 544.2091, found 544.2088. HPLC (Chiralpak AD-3) *i*-PrOH/hexane = 2/98, 0.5 mL/min, T = 28 °C, λ = 250 nm, t_r (major) = 14.0 min, t_r (minor) = 20.3 min, 85% *ee*. $[\alpha]_D^{29.8}$ = +27 (c = 0.1, $CHCl_3$).

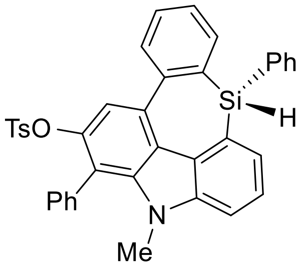

(*S*)-1-Methyl-5,12-diphenyl-1,5-dihydrobenzo[6,7]silepino[2,3,4,5-*def*]carbazol-11-yl 4-methylbenzenesulfonate (4c)

The product **4c** was purified via silica gel column chromatography ((petroleum ether/ethyl acetate = 40/1, v/v) as a white solid (41.2 mg, 68% yield). ^{1}H NMR (600 MHz, $CDCl_3$): δ = 7.90 (d, J = 7.3 Hz, 1H), 7.80 (s, 1H), 7.74 (d, J = 8.0 Hz, 1H), 7.59 – 7.52 (m, 3H), 7.45 (t, J = 7.3 Hz, 1H), 7.42 – 7.33 (m, 5H), 7.33 – 7.26 (m, 5H), 7.24 (t, J = 7.6 Hz, 2H), 7.06 (d, J = 8.0 Hz, 2H), 7.03 (d, J = 7.5 Hz, 1H), 5.65 (s, 1H), 3.16 (s, 3H), 2.38 (s, 3H) ppm. ^{13}C NMR (150 MHz, $CDCl_3$): δ = 145.3, 144.7, 144.6, 141.6, 140.0, 137.6, 136.8, 135.2, 133.4, 133.3, 133.0, 132.9, 132.9, 131.5, 131.3, 130.5, 129.7, 129.5, 128.9, 128.2, 127.9, 127.8, 127.7, 127.7, 127.5, 127.0, 126.4, 125.7, 120.9, 118.0, 115.5, 109.9, 32.7, 21.6 ppm. HRMS (ESI): calcd for $C_{38}H_{30}NO_3SSi$ $[M+H]^+$ 608.1710, found 608.1702. HPLC (Chiralpak AD-H) *i*-PrOH/hexane = 10/90, 1.0 mL/min, T = 28 ^{o}C, λ = 250 nm, t_r (major) = 9.7 min, t_r (minor) = 14.4 min, 90% *ee*. $[\alpha]_D^{29.7}$ = +78 (c = 0.1, $CHCl_3$).

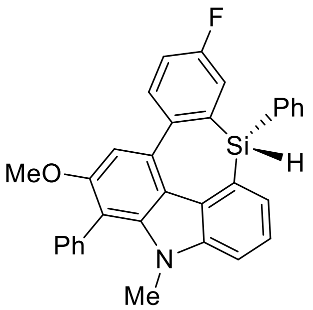

(*S*)-7-Fluoro-11-methoxy-1-methyl-5,12-diphenyl-1,5-dihydrobenzo[6,7]silepino[2,3,4,5-*def*]carbazole (4d)

The product **4d** was purified via silica gel column chromatography ((petroleum ether/ethyl acetate = 80/1, v/v) as a white solid (38.6 mg, 80% yield). ^{1}H NMR (600 MHz, $CDCl_3$): δ = 7.79 – 7.75 (m, 1H), 7.55 (dd, J = 8.4, 2.7 Hz, 1H), 7.50 – 7.41 (m, 9H), 7.35 – 7.31 (m, 2H), 7.29 – 7.20 (m, 4H), 5.57 (s, 1H), 3.90 (s, 3H), 3.20 (s, 3H) ppm. ^{13}C NMR (150 MHz, $CDCl_3$):

δ = 162.0 (d, J_{C-F} = 249.5 Hz), 155.7, 141.9 (d, J_{C-F} = 3.2 Hz), 141.3, 141.0, 136.4, 135.9 (d, J_{C-F} = 4.1 Hz), 135.6, 135.3, 134.6 (d, J_{C-F} = 6.9 Hz), 132.7, 131.4 (d, J_{C-F} = 10.4 Hz), 129.9, 128.1, 127.9 (d, J_{C-F} = 1.7 Hz), 127.8, 127.4, 126.3, 125.4, 125.2, 122.6 (d, J_{C-F} = 19.1 Hz), 117.0 (d, J_{C-F} = 20.7 Hz), 116.7, 113.4, 109.6, 106.4, 56.7, 32.6 ppm. ^{19}F NMR (565 MHz, $CDCl_3$): δ = -116.27 ppm. HRMS (ESI): calcd for $C_{32}H_{25}FNOSi$ $[M+H]^+$ 486.1684, found 486.1679. HPLC (Chiralpak AD-3) *i*-PrOH/hexane = 5/95, 1.0 mL/min, T = 28 °C, λ = 250 nm, t_r (major) = 6.2 min, t_r (minor) = 8.8 min, 91% *ee*. $[\alpha]_D^{29.8}$ = +69 (c = 0.1, $CHCl_3$).

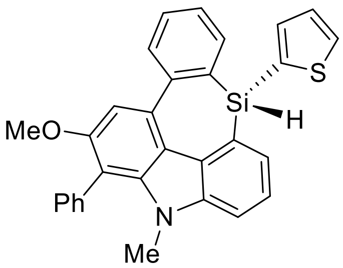

(*S*)-11-Methoxy-1-methyl-12-phenyl-5-(thiophen-2-yl)-1,5-dihydrobenzo[6,7]silepino[2,3,4,5-*def*]carbazole (4e)

The product **4e** was purified via silica gel column chromatography ((petroleum ether/ethyl acetate = 80/1, v/v) as a white solid (18.7 mg, 40% yield). ^{1}H NMR (600 MHz, $CDCl_3$): δ = 7.92 (d, *J* = 7.3 Hz, 1H), 7.80 (d, *J* = 8.0 Hz, 1H), 7.57 (d, *J* = 7.6 Hz, 1H), 7.55 (d, *J* = 4.8 Hz, 1H), 7.53 – 7.42 (m, 8H), 7.36 (s, 1H), 7.33 (d, *J* = 7.8 Hz, 1H), 7.20 (d, *J* = 3.2 Hz, 1H), 7.08 (t, *J* = 3.9 Hz, 1H), 5.75 (s, 1H), 3.92 (s, 3H), 3.21 (s, 3H) ppm. ^{13}C NMR (150 MHz, $CDCl_3$): δ = 155.8, 145.6, 141.3, 141.0, 137.4, 137.1, 136.3, 135.6, 133.1, 132.5, 132.3, 131.9, 131.5, 131.5, 130.4, 128.3, 127.9, 127.4, 127.1, 127.0, 125.4, 124.8, 116.8, 113.4, 109.6, 106.4, 56.7, 32.6 ppm. HRMS (ESI): calcd for $C_{30}H_{24}NOSSi$ $[M+H]^+$ 474.1342, found 474.1339. HPLC (Chiralpak AD-3) *i*-PrOH/hexane = 5/95, 1.0 mL/min, T = 28 °C, λ = 250 nm, t_r (major) = 7.7 min, t_r (minor) = 10.2 min, 86% *ee*. $[\alpha]_D^{29.7}$ = +59 (c = 0.1, $CHCl_3$).

V. Gram-Scale Reaction

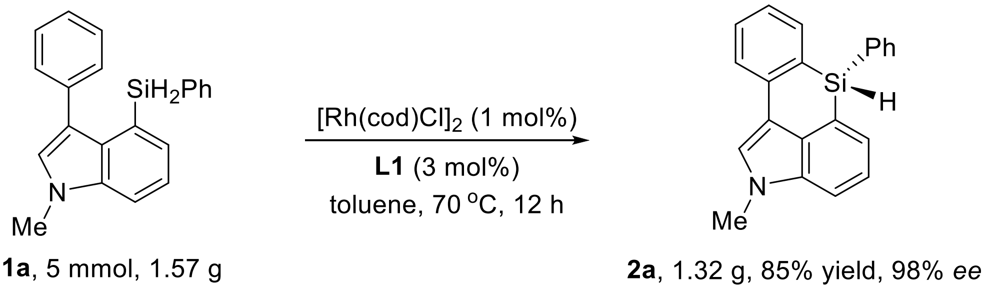

A 250 mL Schlenk tube was charged with **1a** (1.57 g, 5 mmol), $[Rh(cod)Cl]_2$ (24.7 mg, 1 mol%), **L1** (83.5 mg, 3 mol%), and toluene (50 mL) in glovebox. The tube was sealed, then removed from the glovebox, and the mixture was stirred at 70 °C for 12 h. After the completion of the reaction, the solvent was evaporated under reduced pressure and the residue was purified by flash column chromatography on silica gel (petroleum ether/ethyl acetate = 80/1, v/v) afforded the product **2a** as a white solid (1.32 g, 85% yield). HPLC (Chiralpak OD-3) *i*-PrOH/hexane = 5/95, 1.0 mL/min, T = 28 °C, λ = 220 nm, t_r (major) = 11.6 min, t_r (minor) = 16.4 min, 98% *ee*. $[\alpha]_D^{25.0}$ = +188 (c = 0.1, $CHCl_3$).

VI. Photophysical Data of Selected Si-Stereogenic Heterocycles

Supplementary Table 2 Absorption maxima, emission maxima, and Stokes shifts of Si-stereogenic heterocycles.

Compound	λ_{abs} (nm)[a]	λ_{em} (nm)[b]	Stokes Shift (cm^{-1})
**2i**	367	417	3268
**2v**	333, 348	378	3575
**2w**	365, 382	396, 416	2140
**4b**	339	409	5049
**4c**	376	400	1596
**4d**	339	405	4807

[a]Absorption maximum in $CHCl_3$ at 10^{-5} mol/L. [b]Emission maximum in $CHCl_3$ at 10^{-5} mol/L.

VII. Derivatization of Si-Stereogenic Heterocycles

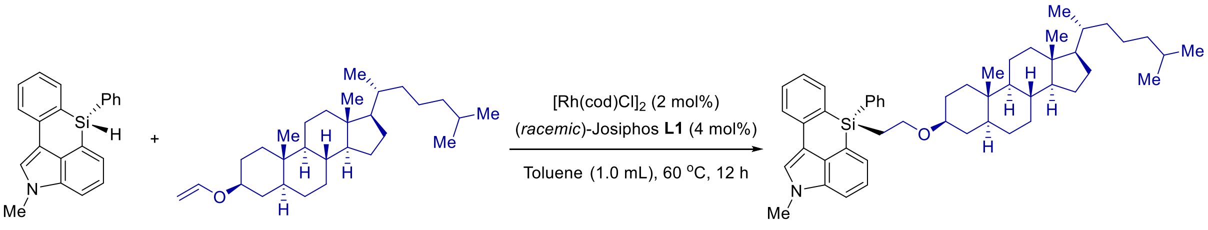

To a 5 mL tube containing **2a** (0.1 mmol, 31.1 mg, 1.0 equiv), vinyl ether (0.2 mmol, 82.9 mg, 2.0 equiv), $[Rh(cod)Cl]_2$ (1.0 mg, 2 mol%) and (*racemic*)-Josiphos **L1** (2.2 mg, 4 mol%) was added toluene (1.0 mL) under argon atmosphere (in glovebox). Then the tube was sealed with a screw cap and taken out of the glovebox. The reaction mixture stirred at 60 °C for 12 h. After the completion of the reaction, the mixture was filtered through a plug of celite with CH_2Cl_2. The filtrate was concentrated under reduced pressure, and the residue was purified by flash chromatography on silica gel to give **5a** as a white solid (56.1 mg, 77% yield). ^{1}H NMR (600 MHz, $CDCl_3$) δ = 7.75 (d, J = 7.8 Hz, 1H), 7.63 (d, J = 7.2 Hz, 1H), 7.58 – 7.52 (m, 3H), 7.45 (d, J = 6.6 Hz, 1H), 7.38 – 7.22 (m, 6H), 7.13 (t, J = 7.8 Hz, 1H), 3.82 (s, 3H), 3.54 – 3.45 (m, 2H), 3.03 – 2.95 (m, 1H), 1.92 (d, J = 12.6 Hz, 1H), 1.84 – 1.73 (m, 3H), 1.64 – 1.55 (m, 2H), 1.54 – 1.47 (m, 2H), 1.44 – 1.17 (m, 11H), 1.16 – 0.90 (m, 12H), 0.90 – 0.82 (m, 10H), 0.70 (s, 3H), 0.61 (s, 3H), 0.55 – 0.46 (m, 1H) ppm. ^{13}C NMR (150 MHz, $CDCl_3$) δ = 139.4, 137.1, 135.6, 135.3, 134.8, 131.7, 129.5, 129.3, 129.1, 127.8, 126.0, 125.2, 124.7, 124.4, 122.6, 122.3, 115.7, 110.3, 77.9, 64.2, 56.5, 56.3, 54.4, 44.8, 42.6, 40.0, 39.5, 36.9, 36.2, 35.8, 35.7, 35.4, 34.6, 33.0, 32.1, 28.8, 28.2, 28.2, 28.0, 24.2, 23.8, 22.80, 22.5, 21.2, 18.6, 17.3, 12.2, 12.0. ppm. HRMS (ESI): calcd for $C_{50}H_{68}NOSi$ $[M+H]^+$ 726.5065, found 726.5083. HPLC (Chiralpak OD-3) *i*-PrOH/hexane = 15/85, 1.0 mL/min, T = 28 °C, λ = 230 nm, t_r (minor) = 11.0 min, t_r (major) = 40.4 min, 98% *de*. $[\alpha]_D^{29.7}$ = +17 (c = 0.1, $CHCl_3$).

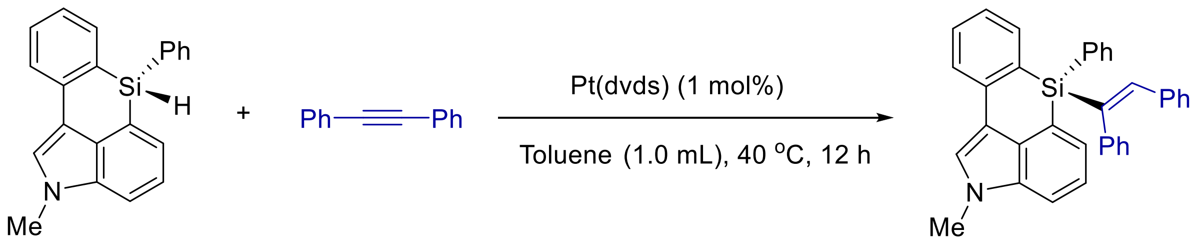

2a, 98% *ee* **5b**, 98% *ee*

To a 5 mL tube containing **2a** (0.1 mmol, 31.1 mg, 1.0 equiv), 1,2-diphenylacetylene (0.2 mmol, 35.6 mg, 2.0 equiv), and Pt(dvds) (10 µL, 0.1 M in xylene) was added toluene (1.0 mL) under argon atmosphere (in glovebox). Then the tube was sealed with a screw cap and taken out of the glovebox. The reaction mixture stirred at 40 °C for 12 h. After the completion of the reaction, the mixture was filtered through a plug of celite with CH_2Cl_2. The filtrate was concentrated under reduced pressure, and the residue was purified by flash chromatography on silica gel to give **5b** as a white solid (34.0 mg, 70% yield).[12] ^{1}H NMR (600 MHz, $CDCl_3$) δ = 7.74 (d, J = 7.9 Hz, 1H), 7.71 (dd, J = 7.4, 1.8 Hz, 2H), 7.61 (d, J = 6.8 Hz, 1H), 7.50 (s, 1H), 7.39 – 7.37 (m, 1H), 7.35 – 7.33 (m, 2H), 7.32 – 7.28 (m, 4H), 7.11 – 7.03 (m, 8H), 6.90 (dd, J = 7.4, 2.0 Hz, 2H), 6.87 (dd, J = 7.5, 1.8 Hz, 2H), 3.77 (s, 3H) ppm. ^{13}C NMR (150 MHz, $CDCl_3$) δ = 143.0, 141.9, 141.6, 139.7, 137.2, 136.8, 135.8, 135.7, 135.4, 131.6, 129.7, 129.6, 129.2, 128.5, 128.2, 127.9, 127.8, 127.8, 127.2, 126.9, 125.7, 125.3, 124.2, 123.7, 122.5, 122.4, 115.6, 110.3, 32.9 ppm. HRMS (ESI): calcd for $C_{35}H_{28}NSi$ $[M+H]^+$ 490.1986, found 490.1981. HPLC (Chiralpak AD-3) *i*-PrOH/hexane = 5/95, 1.0 mL/min, T = 28 °C, λ = 250 nm, t_r (major) = 7.4 min, t_r (minor) = 17.0 min, 98% *ee*. $[\alpha]_D^{25.0}$ = -35 (c = 0.1, $CHCl_3$).

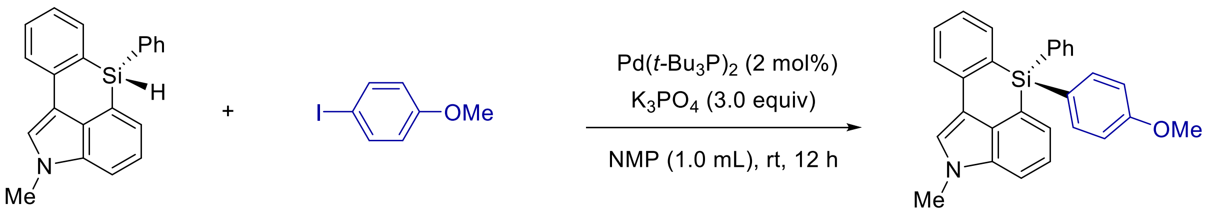

2a, 98% *ee* **5c**, 98% *ee*

To a 5 mL tube containing **2a** (0.1 mmol, 31.1 mg, 1.0 equiv), 1-iodo-4-methoxybenzene (0.2 mmol, 46.8 mg, 2.0 equiv), Pd(*t*-$Bu_3P)_2$ (1.0 mg, 2 mol%) and K_3PO_4 (0.3 mmol, 63.7 mg, 3.0 equiv) was added 1-Methyl-2-pyrrolidinone (NMP, 1.0 mL) under argon atmosphere (in glovebox). Then the tube was sealed with a screw cap and taken out of the glovebox. The

reaction mixture stirred at room temperature for 12 h. The mixture was quenched with H_2O and extracted with ethyl acetate. The organic layer was washed with brine, dried over Na_2SO_4 and concentrated under reduced pressure. The residue was purified by flash chromatography on silica gel to give **5c** as a white solid (30.1 mg, 72% yield). ^1H NMR (600 MHz, $CDCl_3$) δ = 7.79 (d, J = 7.9 Hz, 1H), 7.68 (d, J = 7.4 Hz, 1H), 7.61 (d, J = 7.7 Hz, 2H), 7.57 (s, 1H), 7.54 (d, J = 7.8 Hz, 2H), 7.51 (d, J = 6.6 Hz, 1H), 7.40 – 7.26 (m, 6H), 7.14 (t, J = 7.3 Hz, 1H), 6.86 (d, J = 7.8 Hz, 2H), 3.82 (s, 3H), 3.76 (s, 3H) ppm. ^{13}C NMR (150 MHz, $CDCl_3$) δ = 160.7, 139.6, 137.4, 136.5, 136.5, 135.8, 135.4, 131.7, 129.6, 129.2, 127.8, 126.9, 126.4, 125.4, 124.8, 124.6, 122.7, 122.4, 115.7, 113.6, 110.4, 55.0, 33.0 ppm. HRMS (ESI): calcd for $C_{28}H_{24}NOSi$ $[M+H]^+$ 418.1622, found 418.1617. HPLC (Chiralpak OD-3) *i*-PrOH/hexane = 5/95, 1.0 mL/min, T = 28 °C, λ = 210 nm, t_r (minor) = 16.4 min, t_r (major) = 21.3 min, 98% *ee*. $[\alpha]_D^{29.6}$ = -18 (c = 0.1, $CHCl_3$).

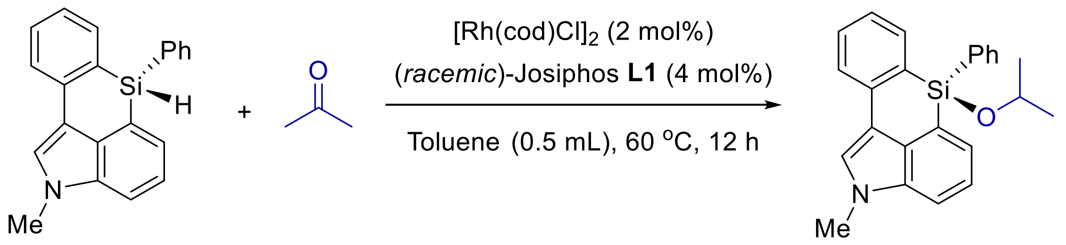

2a, 98% *ee*   **5d**, 98% *ee*

To a 5 mL tube containing **2a** (0.05 mmol, 15.6 mg, 1.0 equiv), acetone (0.1 mmol, 82.9 mg, 2.0 equiv), $[Rh(cod)Cl]_2$ (0.5 mg, 2 mol%) and (*racemic*)-Josiphos **L1** (1.1 mg, 4 mol%) was added toluene (0.5 mL) under argon atmosphere (in glovebox). Then the tube was sealed with a screw cap and taken out of the glovebox. The reaction mixture stirred at 60 °C for 12 h. After the completion of the reaction, the mixture was filtered through a plug of celite with CH_2Cl_2. The filtrate was concentrated under reduced pressure, and the residue was purified by flash chromatography on silica gel to give **5d** as a white solid (11.0 mg, 60% yield). ^1H NMR (600 MHz, $CDCl_3$) δ = 7.79 (d, J = 7.9 Hz, 1H), 7.74 (d, J = 7.4 Hz, 1H), 7.66 – 7.64 (m, 2H), 7.61 (s, 1H), 7.56 (d, J = 6.8 Hz, 1H), 7.43 (d, J = 8.0 Hz, 1H), 7.41 – 7.39 (m, 1H), 7.36 (d, J = 7.0 Hz, 1H), 7.34 – 7.29 (m, 3H), 7.16 (t, J = 7.3 Hz, 1H), 3.99 (dt, J = 12.1, 6.0 Hz, 1H), 3.86 (s, 3H), 1.06 (d, J = 1.7 Hz, 3H), 1.05 (d, J = 1.7 Hz, 3H) ppm. ^{13}C NMR (150 MHz, $CDCl_3$) δ =

140.1, 136.6, 135.8, 135.5, 134.9, 132.1, 130.0, 129.9, 129.5, 127.6, 126.3, 125.7, 125.0, 124.5, 122.5, 122.2, 115.1, 110.9, 65.9, 33.0, 25.5, 25.4 ppm. HRMS (ESI): calcd for $C_{24}H_{24}NOSi$ $[M+H]^+$ 370.1622, found 370.1616. HPLC (Chiralpak AD-3) *i*-PrOH/hexane = 5/95, 1.0 mL/min, T = 28 °C, λ = 250 nm, t_r (major) = 4.9 min, t_r (minor) = 15.8 min, 98% *ee*. $[\alpha]_D^{25.0}$ = +88 (c = 0.1, $CHCl_3$).

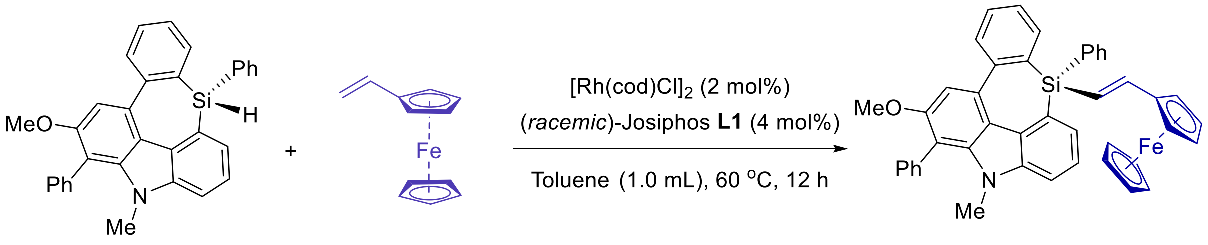

4a, 90% *ee* **6a**, 90% *ee*

To a 5 mL tube containing **4a** (0.05 mmol, 23.4 mg, 1.0 equiv), vinyl ferrocene (0.1 mmol, 22.7 mg, 2.0 equiv), $[Rh(cod)Cl]_2$ (0.5 mg, 2 mol%) and (*racemic*)-Josiphos **L1** (1.1 mg, 4 mol%) was added toluene (1.0 mL) under argon atmosphere (in glovebox). Then the tube was sealed with a screw cap and taken out of the glovebox. The reaction mixture stirred at 60 °C for 12 h. After the completion of the reaction, the mixture was filtered through a plug of celite with CH_2Cl_2. The filtrate was concentrated under reduced pressure, and the residue was purified by flash chromatography on silica gel to give **6a** as an orange solid (16.8 mg, 50% yield). ^1H NMR (600 MHz, $CDCl_3$) δ = 7.83 (d, *J* = 8.0 Hz, 1H), 7.70 (dd, *J* = 7.4, 1.2 Hz, 1H), 7.64 (d, *J* = 6.7 Hz, 2H), 7.56 (td, *J* = 7.8, 1.2 Hz, 1H), 7.49 – 7.42 (m, 4H), 7.41 – 7.35 (m, 7H), 7.35 – 7.31 (m, 2H), 6.46 (d, *J* = 18.8 Hz, 1H), 6.21 (d, *J* = 18.8 Hz, 1H), 4.30 (s, 1H), 4.26 (s, 1H), 4.17 (d, *J* = 1.2 Hz, 2H), 3.89 (s, 3H), 3.80 (s, 5H), 3.20 (s, 3H) ppm. ^{13}C NMR (150 MHz, $CDCl_3$) δ = 155.7, 148.2, 146.4, 141.4, 140.8, 138.0, 136.7, 136.4, 135.8, 134.8, 134.0, 132.7, 131.5, 131.5, 129.9, 129.5, 129.4, 128.3, 127.8, 127.8, 127.8, 127.3, 126.8, 125.5, 124.9, 118.7, 117.2, 113.3, 109.1, 106.5, 83.7, 69.3, 69.2, 69.1, 67.3, 67.0, 56.8, 32.6 ppm. HRMS (ESI): calcd for $C_{44}H_{35}FeNOSi$ $[M]^+$ 677.1837, found 677.1821. HPLC (Chiralpak AD-3) *i*-PrOH/hexane = 5/95, 1.0 mL/min, T = 28 °C, λ = 250 nm, t_r (minor) = 4.7 min, t_r (major) = 6.6 min, 90% *ee*. $[\alpha]_D^{29.7}$ = +114 (c = 0.1, $CHCl_3$).

VIII. Single Crystal X-Ray Diffraction

Single crystal suitable for X-ray diffraction of compound **2j** was obtained from a solution of the compound **2j** (91% *ee*) in dichloromethane layered with hexane. The X-ray crystal structure is deposited in the Cambridge Crystallographic Data Centre under reference number CCDC 2031952. Diffraction Data were collected on a BrukerD8 venture employing Mo-Kα radiation (λ = 0.71073 Å). The crystal structure was shown in **Supplementary Fig. 1**. The detailed information was listed in the **Supplementary Table 3**.

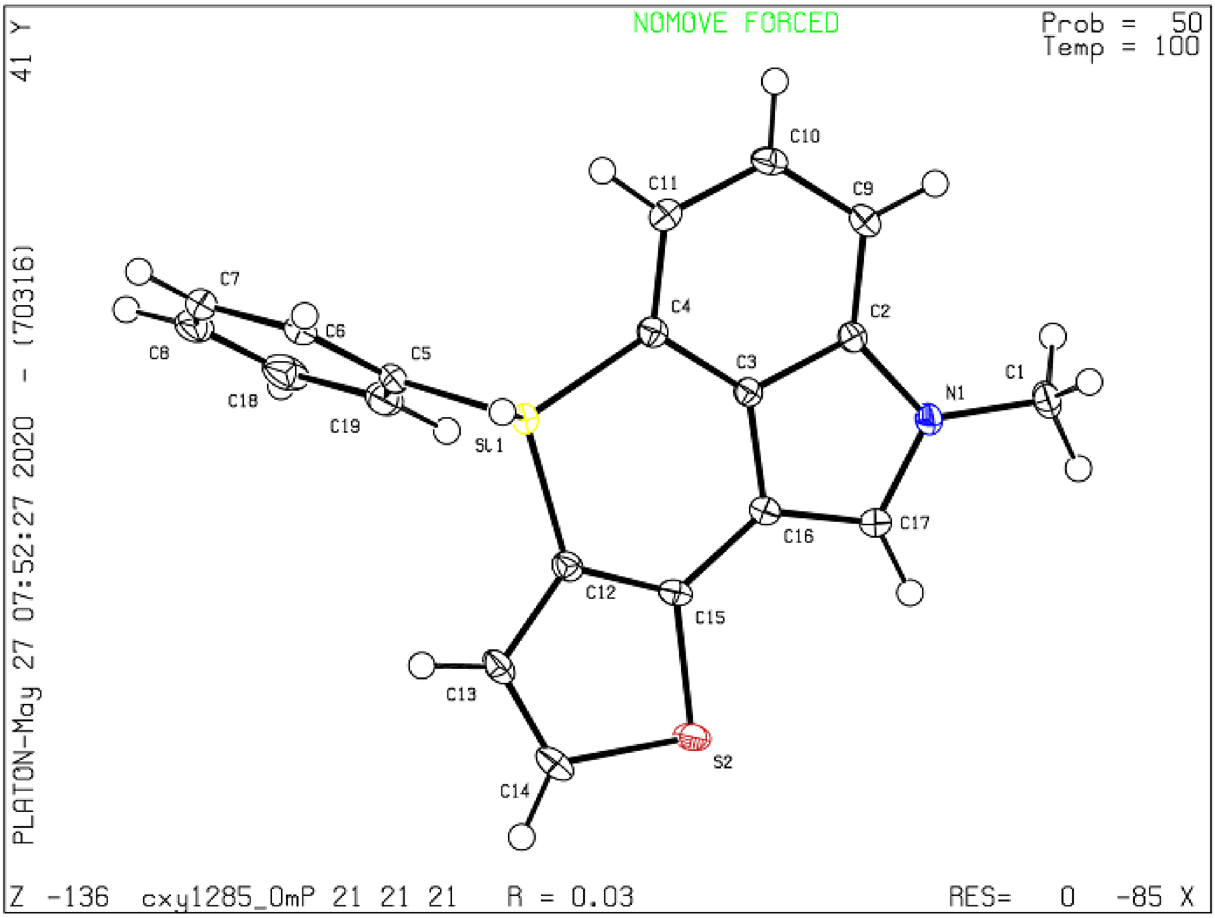

Supplementary Fig. 1 Crystal structure of 2j (CCDC 2031952).

Supplementary Table 3 Crystallographic data and structure refinement for compound 2j.

Compound	**2j**
CCDC deposition No.	2031952
Empirical formula	$C_{19}H_{15}NSSi$
Formula weight	317.47
Temperature	100 K
Crystal system, space group	orthorhombic, $P2_12_12_1$
Unit cell dimensions	a = 7.3548(3) Å alpha = 90°<br>b = 8.2124(3) Å beta = 90°<br>c = 26.1737(9) Å gamma = 90°
Volume	1580.91(10) $Å^3$
Z, Calculated density	4, 1.334 g/cm^3
Absorption coefficient	0.276 mm^{-1}
F(000)	664.0
Crystal size	0.35 × 0.31 × 0.23 mm^3
Radiation	MoKα (λ = 0.71073)
2Θ range for data collection	5.198 to 61.068°
Index ranges	$-7 \leq h \leq 10$, $-11 \leq k \leq 10$, $-37 \leq l \leq 31$
Reflections collected	16195
Independent reflections	4773 [R_{int} = 0.0374, R_{sigma} = 0.0403]
Data / restaints / parameters	4773/0/201
Goodness-of-fit on F^2	1.043
Final R indices [I>=2sigma(I)]	R_1 = 0.0345, wR_2 = 0.0748
R indices (all data)	R_1 = 0.0412, wR_2 = 0.0776
Largest diff. peak/hole	0.34 /-0.17 e $Å^{-3}$
Flack parameter	-0.02(3)

Single crystal suitable for X-ray diffraction of compound **6a** was obtained from a solution of the compound **6a** (90% *ee*) in dichloromethane layered with methyl alcohol. The X-ray crystal structure is deposited in the Cambridge Crystallographic Data Centre under reference number CCDC 2031700. Diffraction Data were collected on a BrukerD8 venture employing Cu-Kα radiation (λ = 1.54178 Å). The crystal structure was shown in **Supplementary Fig. 2**. The detailed information was listed in the **Supplementary Table 4**.

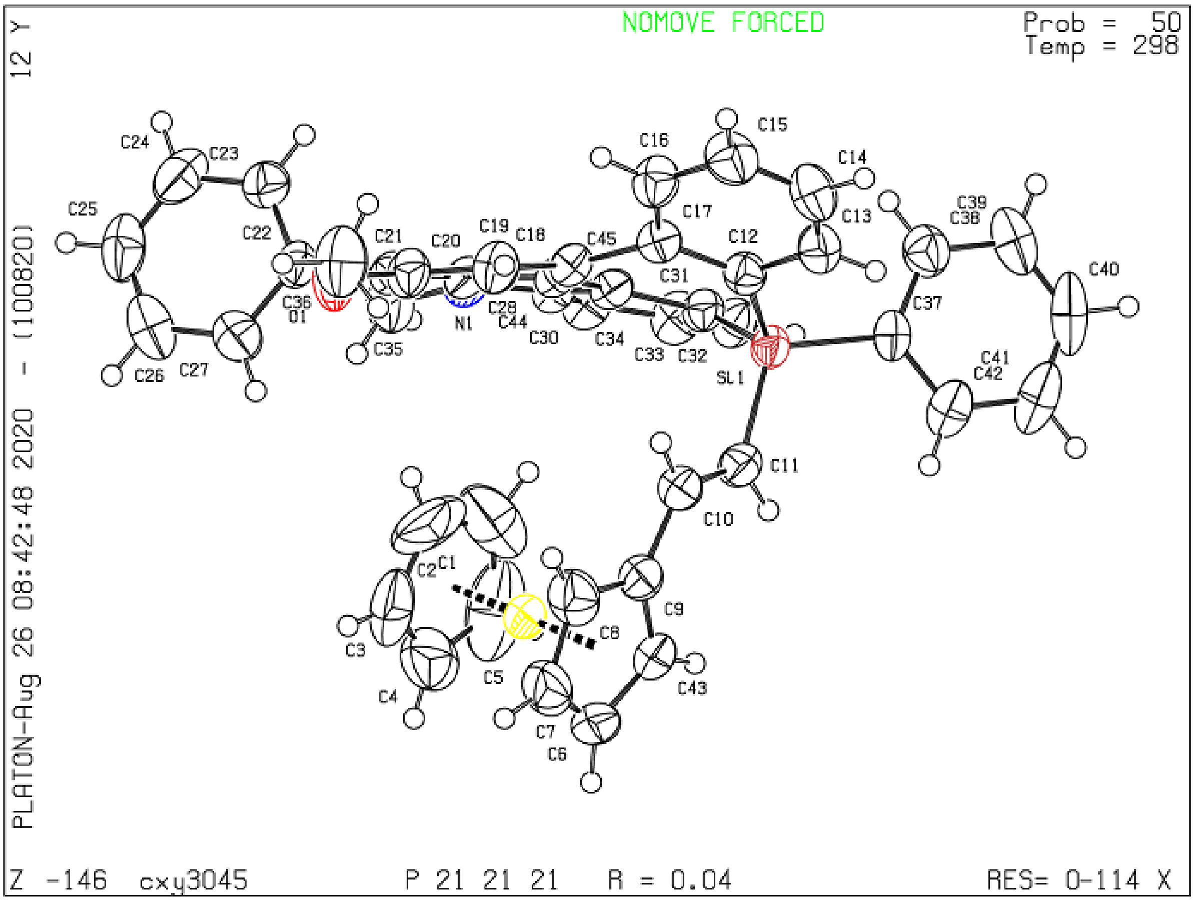

Supplementary Fig. 2 Crystal structure of 6a (CCDC 2031700).

Supplementary Table 4 Crystallographic data and structure refinement for compound 6a.

Compound	**6a**
CCDC deposition No.	2031700
Empirical formula	$C_{44}H_{35}FeNOSi$
Formula weight	677.67
Temperature	298 K
Crystal system, space group	orthorhombic, $P2_12_12_1$
Unit cell dimensions	a = 10.5746(7) Å alpha = 90°<br>b = 15.7664(9) Å beta = 90°<br>c = 20.3486(12) Å gamma = 90°
Volume	3392.60(4) $Å^3$
Z, Calculated density	4, 1.327 g/cm^3
Absorption coefficient	4.177 mm^{-1}
F(000)	1416.0
Crystal size	0.35 × 0.32 × 0.31 mm^3
Radiation	CuKα (λ = 1.54178)
2Θ range for data collection	7.092 to 136.874°
Index ranges	$-12 \leq h \leq 12$, $-18 \leq k \leq 18$, $-24 \leq l \leq 22$
Reflections collected	34876
Independent reflections	6213 [R_{int} = 0.0858, R_{sigma} = 0.0573]
Data / restaints / parameters	6213/0/436
Goodness-of-fit on F^2	0.979
Final R indices [I>=2sigma(I)]	R_1 = 0.0373, wR_2 = 0.0891
R indices (all data)	R_1 = 0.0430, wR_2 = 0.0914
Largest diff. peak/hole	0.28 /-0.28 e $Å^{-3}$
Flack parameter	-0.034(5)

IX. NMR Spectra

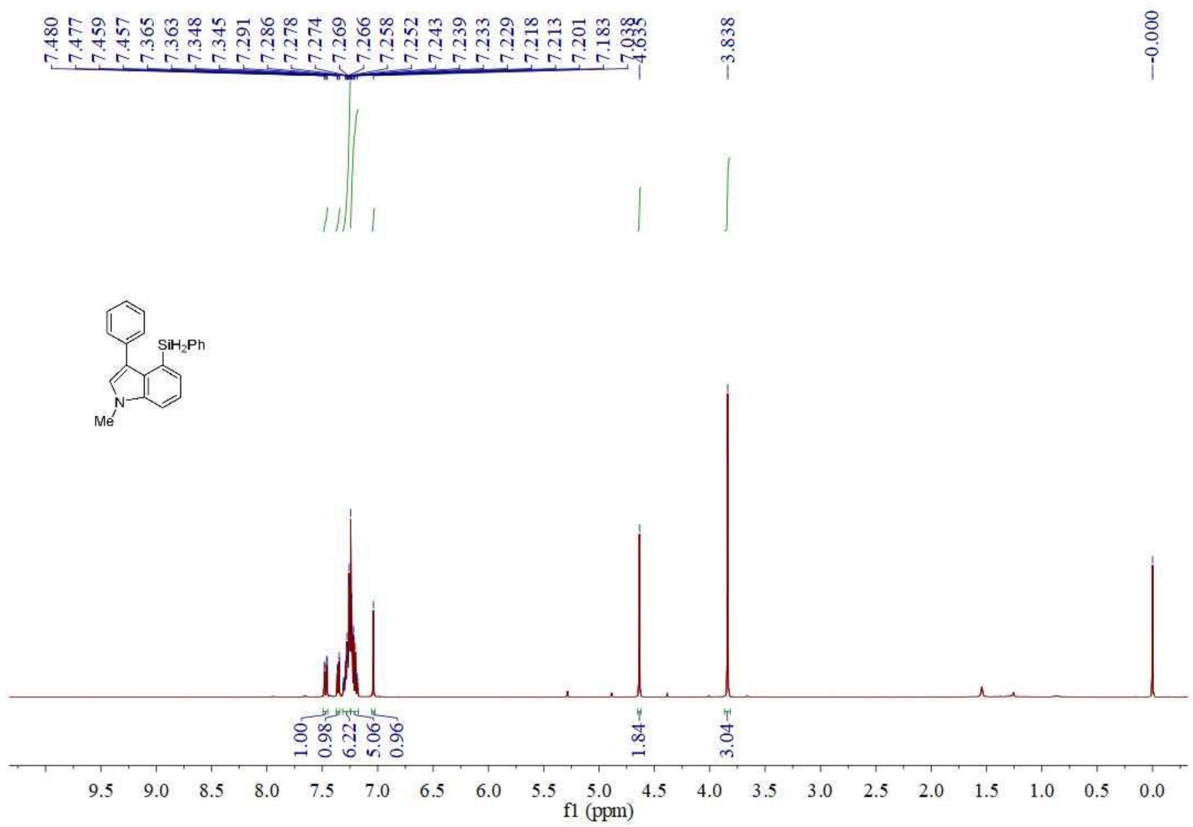

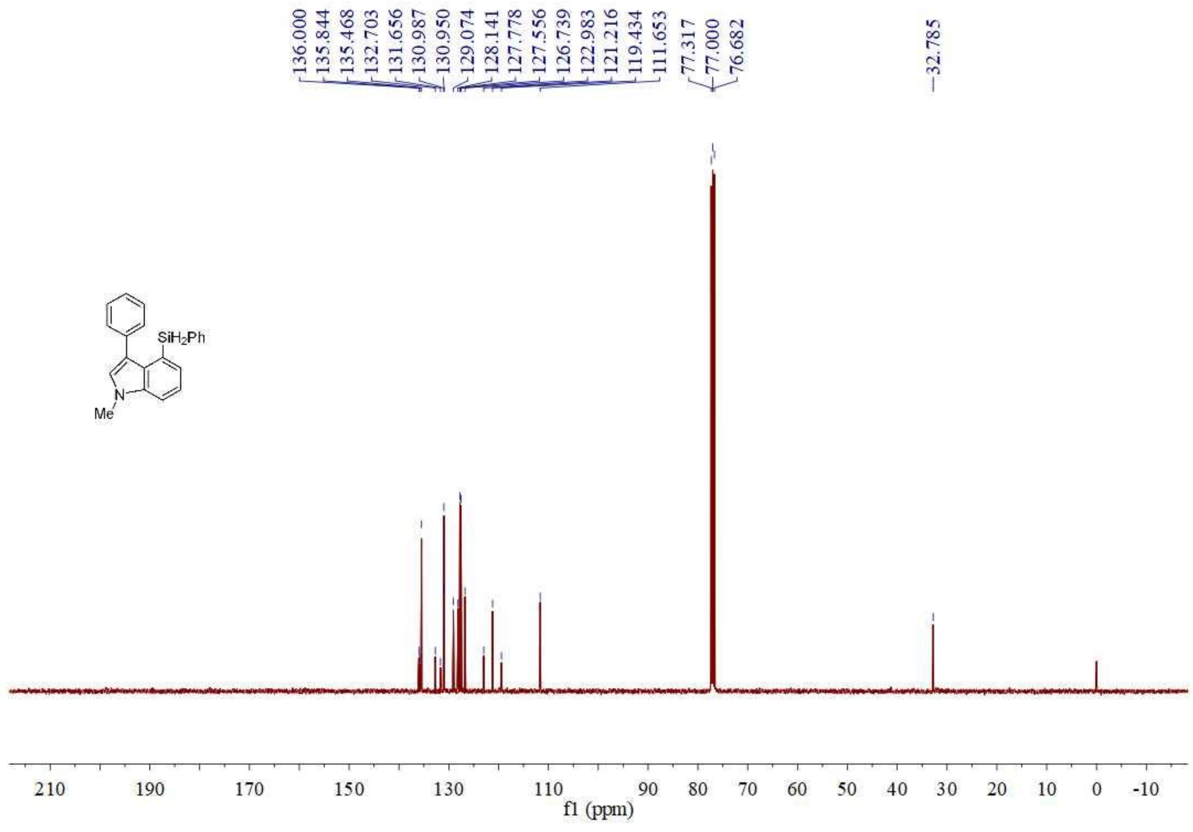

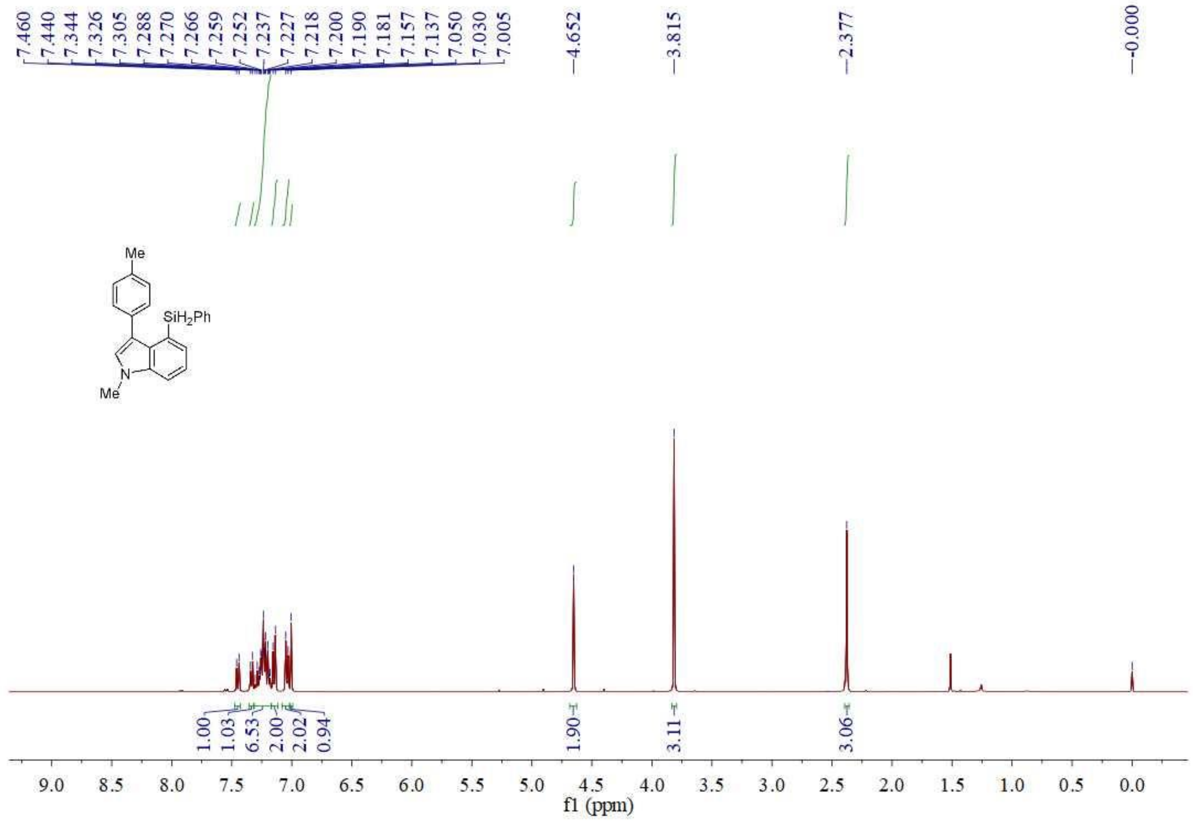

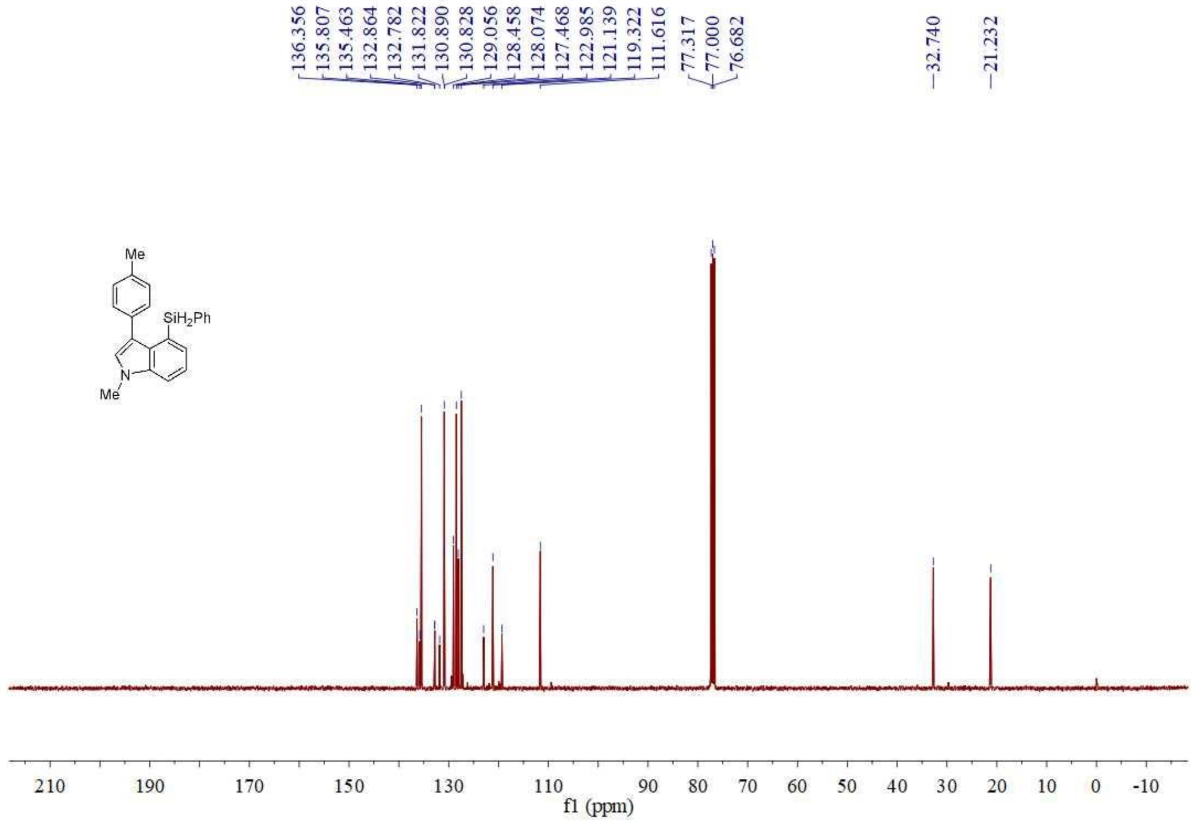

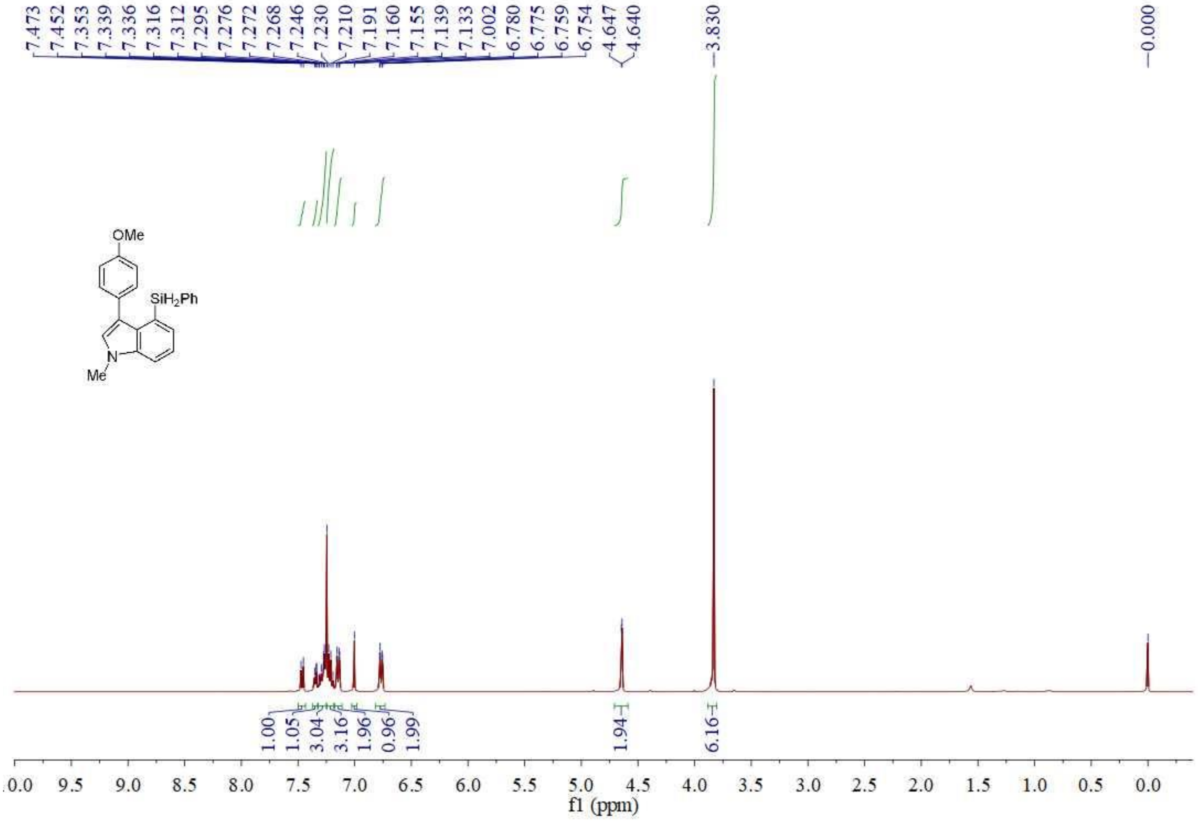
7.473
7.452
7.353
7.339
7.336
7.316
7.312
7.295
7.276
7.272
7.268
7.246
7.230
7.210
7.191
7.160
7.155
7.139
7.133
7.002
6.780
6.775
6.759
6.754
4.647
4.640
3.830
0.000
OMe
SiH2Ph
N
Me
1.00
1.05
3.04
3.16
1.96
0.96
1.99
1.94
6.16
f1 (ppm)

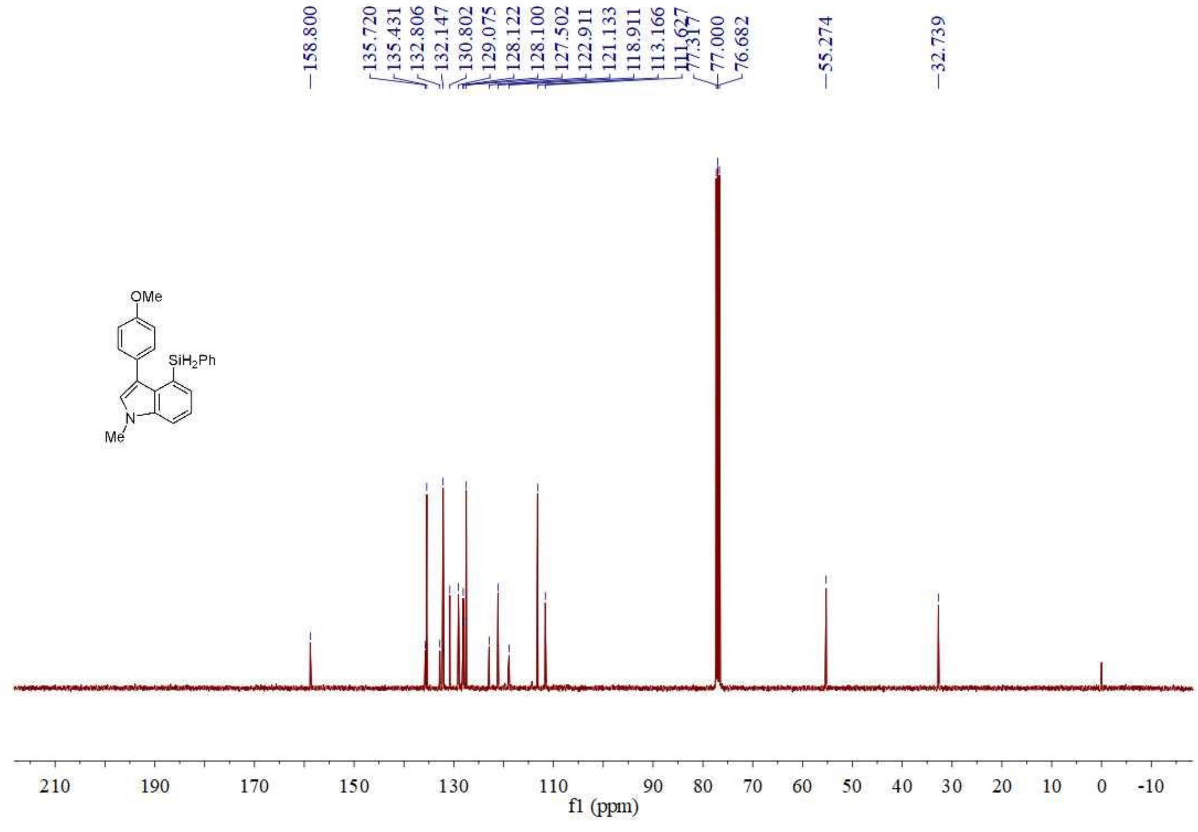
158.800
135.720
135.431
132.806
132.147
130.802
129.075
128.122
128.100
127.502
122.911
121.133
118.911
113.166
111.627
77.317
77.000
76.682
55.274
32.739
OMe
SiH2Ph
N
Me
f1 (ppm)

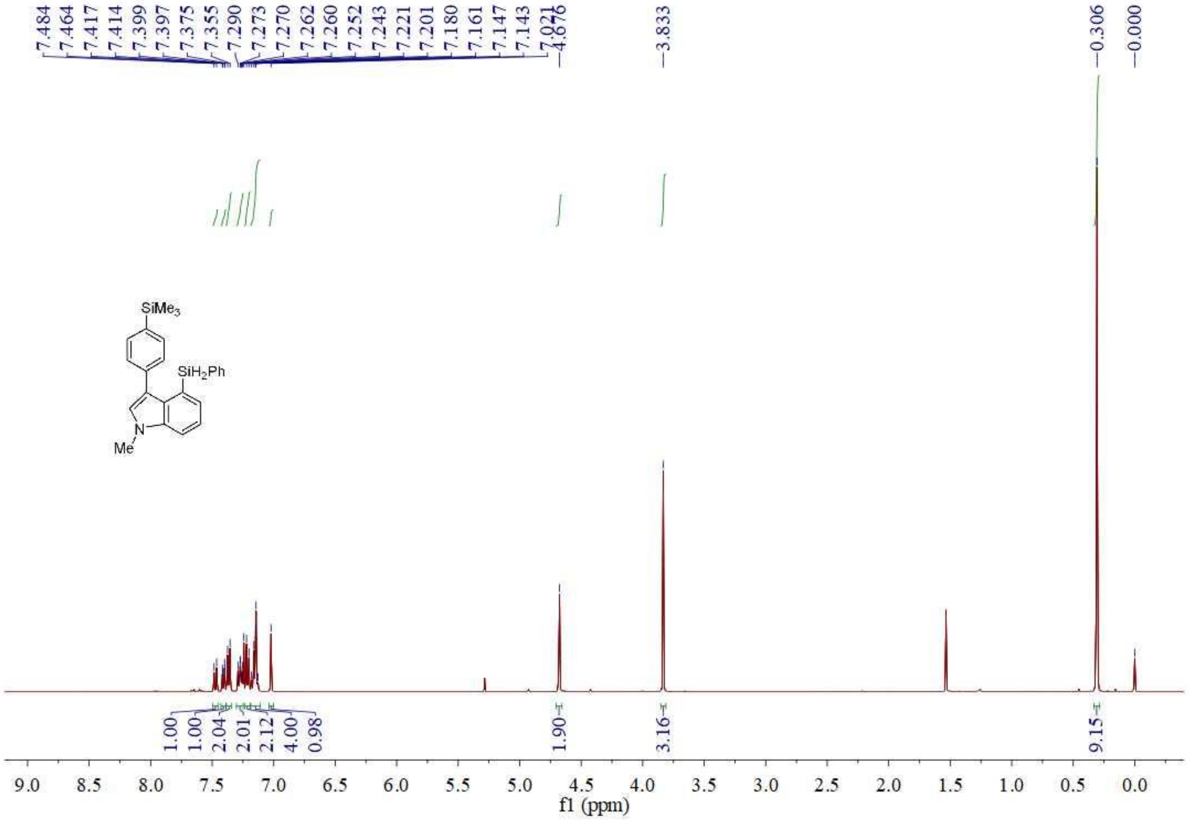
7.484
7.464
7.417
7.414
7.399
7.397
7.375
7.355
7.290
7.273
7.270
7.262
7.260
7.252
7.243
7.221
7.201
7.180
7.161
7.147
7.143
7.021
4.676
3.833
0.306
0.000
SiMe3
SiH2Ph
N
Me
1.00
1.00
2.04
2.01
2.12
4.00
0.98
1.90
3.16
9.15
9.0 8.5 8.0 7.5 7.0 6.5 6.0 5.5 5.0 4.5 4.0 3.5 3.0 2.5 2.0 1.5 1.0 0.5 0.0
f1 (ppm)

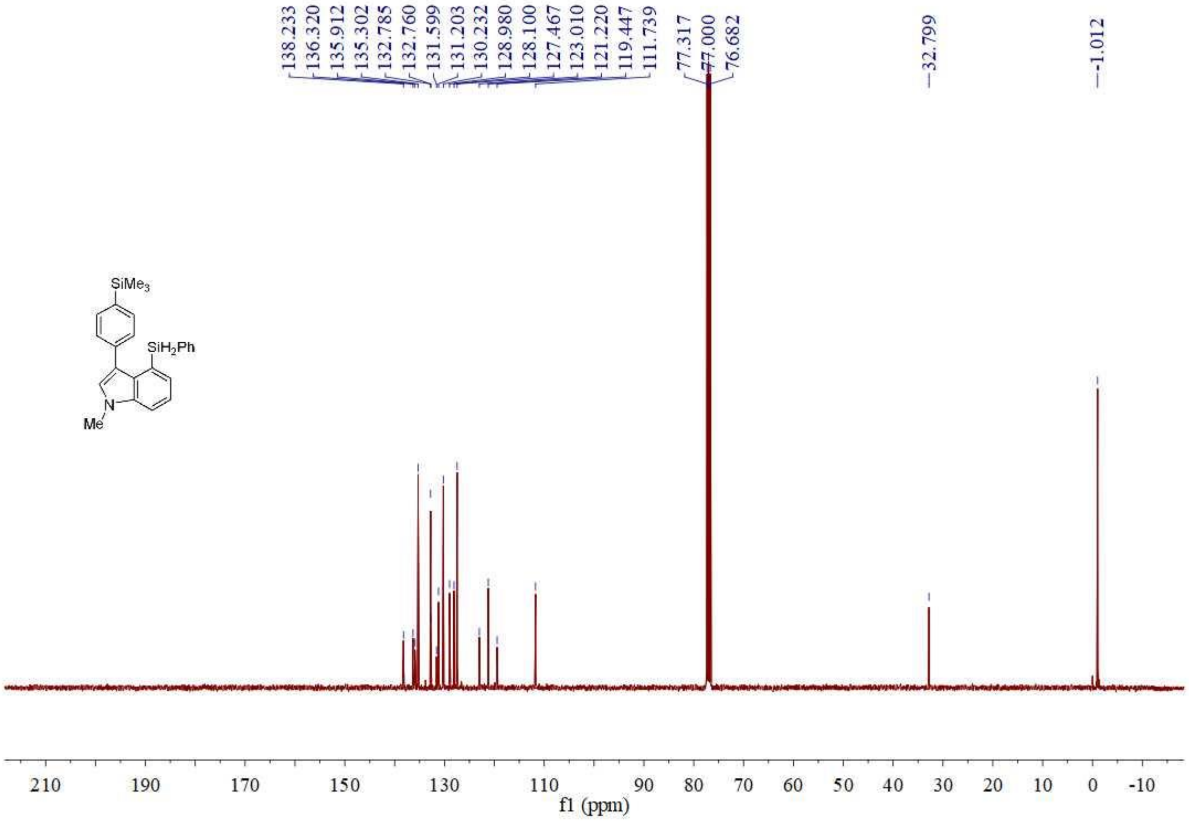
138.233
136.320
135.912
135.302
132.785
132.760
131.599
131.203
130.232
128.980
128.100
127.467
123.010
121.220
119.447
111.739
77.317
77.000
76.682
32.799
-1.012
SiMe3
SiH2Ph
N
Me
210 190 170 150 130 110 90 80 70 60 50 40 30 20 10 0 -10
f1 (ppm)

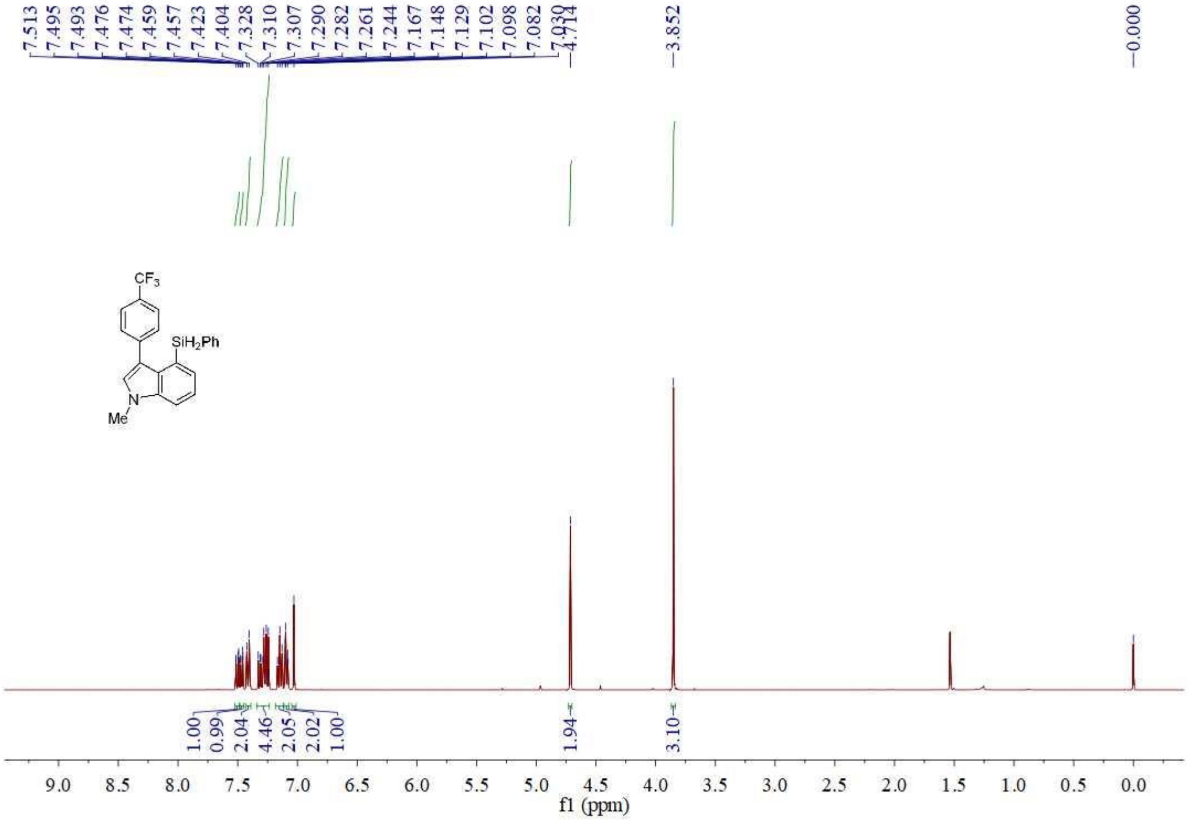
7.513
7.495
7.493
7.476
7.474
7.459
7.457
7.423
7.404
7.328
7.310
7.307
7.290
7.282
7.261
7.244
7.167
7.148
7.129
7.102
7.098
7.082
7.030
4.714
3.852
0.000
CF3
SiH2Ph
N
Me
1.00
0.99
2.04
4.46
2.05
2.02
1.00
1.94
3.10
9.0 8.5 8.0 7.5 7.0 6.5 6.0 5.5 5.0 4.5 4.0 3.5 3.0 2.5 2.0 1.5 1.0 0.5 0.0
f1 (ppm)

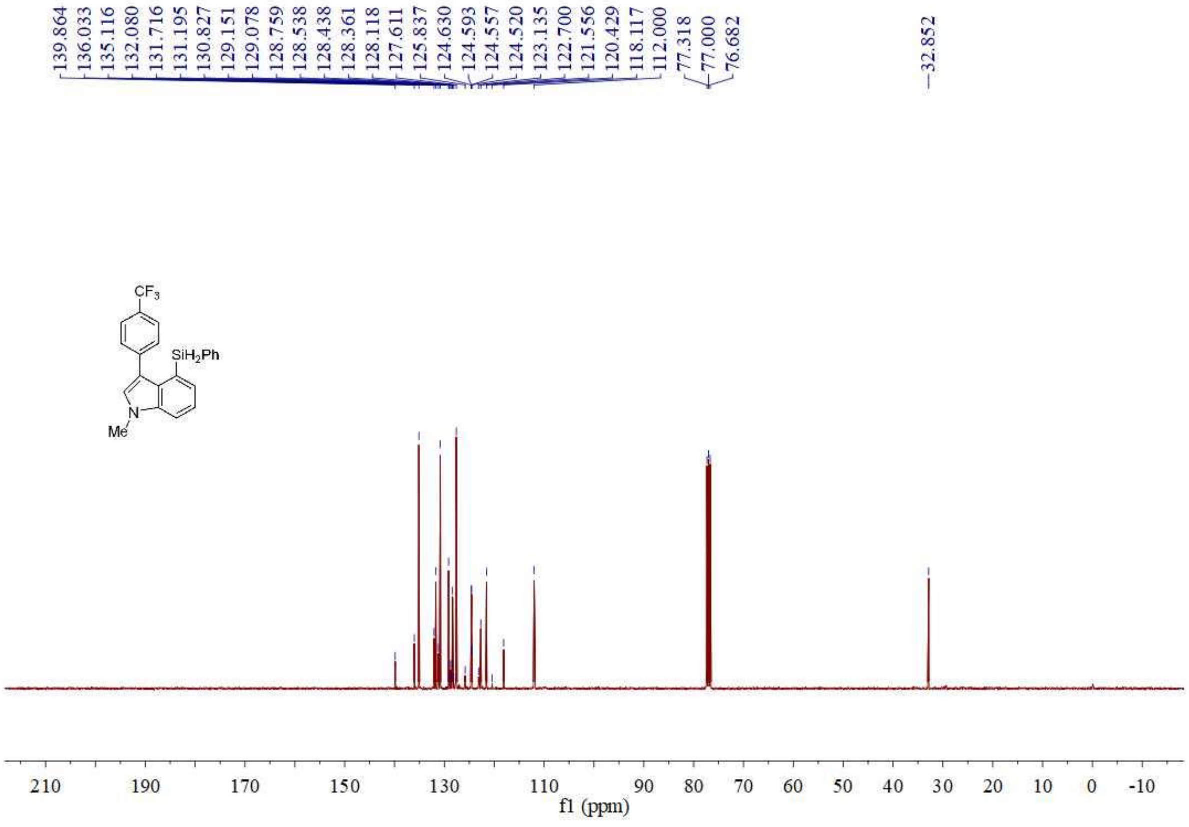
139.864
136.033
135.116
132.080
131.716
131.195
130.827
129.151
129.078
128.759
128.538
128.438
128.361
128.118
127.611
125.837
124.630
124.593
124.557
124.520
123.135
122.700
121.556
120.429
118.117
112.000
77.318
77.000
76.682
32.852
CF3
SiH2Ph
N
Me
210 190 170 150 130 110 90 80 70 60 50 40 30 20 10 0 -10
f1 (ppm)

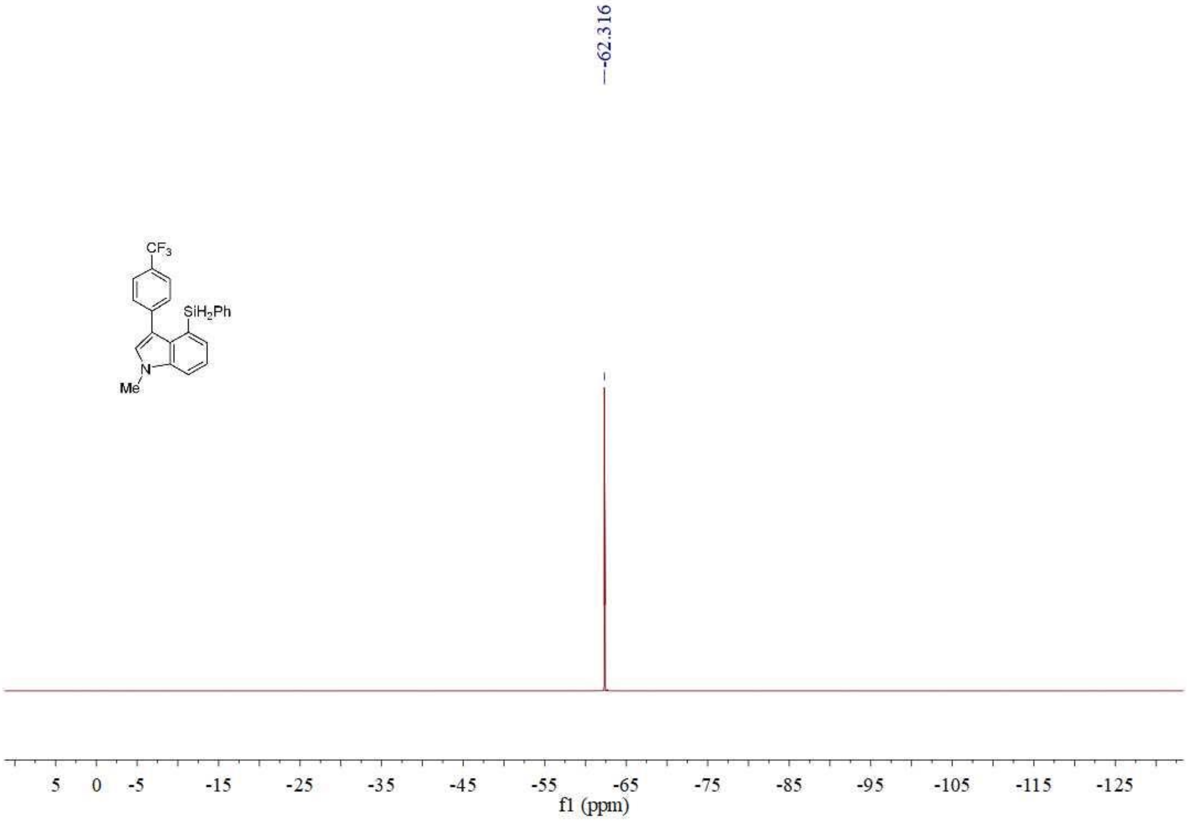
-62.316
CF3
SiH2Ph
N
Me
5
0
-5
-15
-25
-35
-45
-55
-65
-75
-85
-95
-105
-115
-125
f1 (ppm)

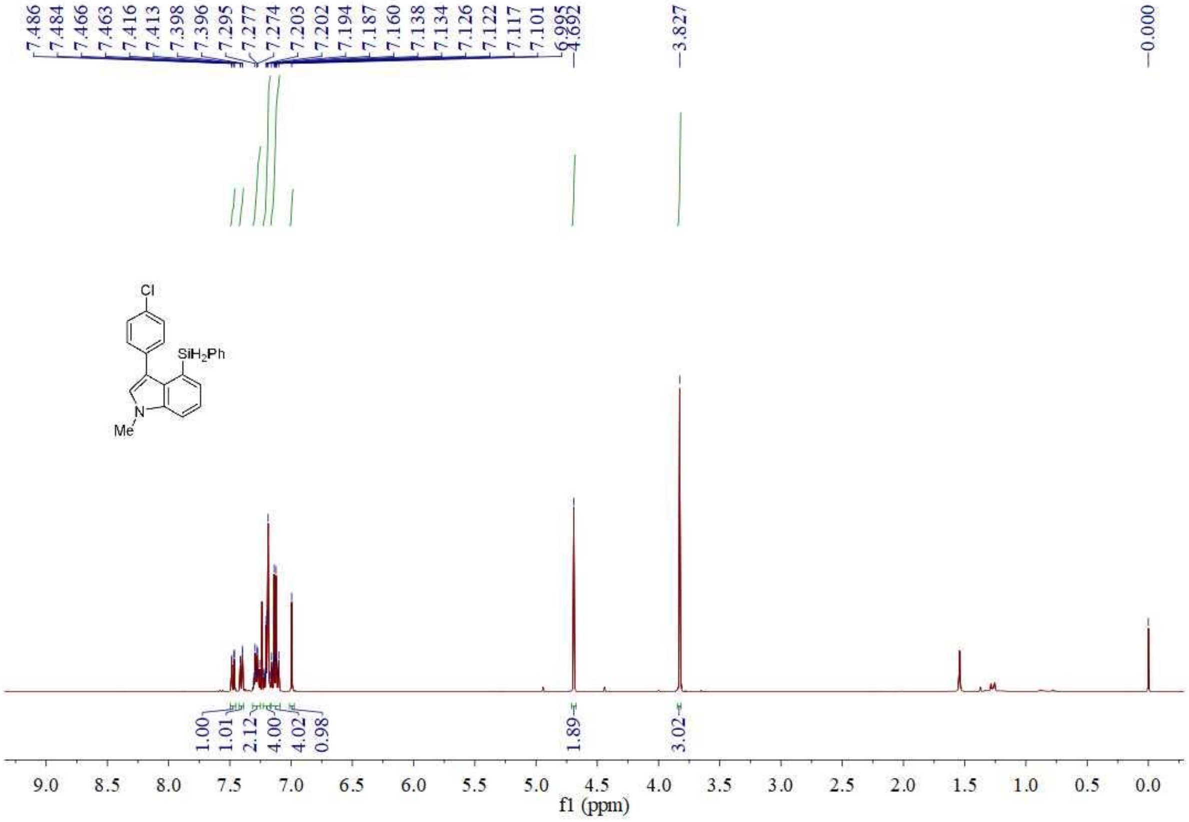
7.486
7.484
7.466
7.463
7.416
7.413
7.398
7.396
7.295
7.277
7.274
7.203
7.202
7.194
7.187
7.160
7.138
7.134
7.126
7.122
7.117
7.101
6.995
4.692
3.827
0.000
Cl
SiH2Ph
N
Me
1.00
1.01
2.12
4.00
4.02
0.98
1.89
3.02
9.0 8.5 8.0 7.5 7.0 6.5 6.0 5.5 5.0 4.5 4.0 3.5 3.0 2.5 2.0 1.5 1.0 0.5 0.0
f1 (ppm)

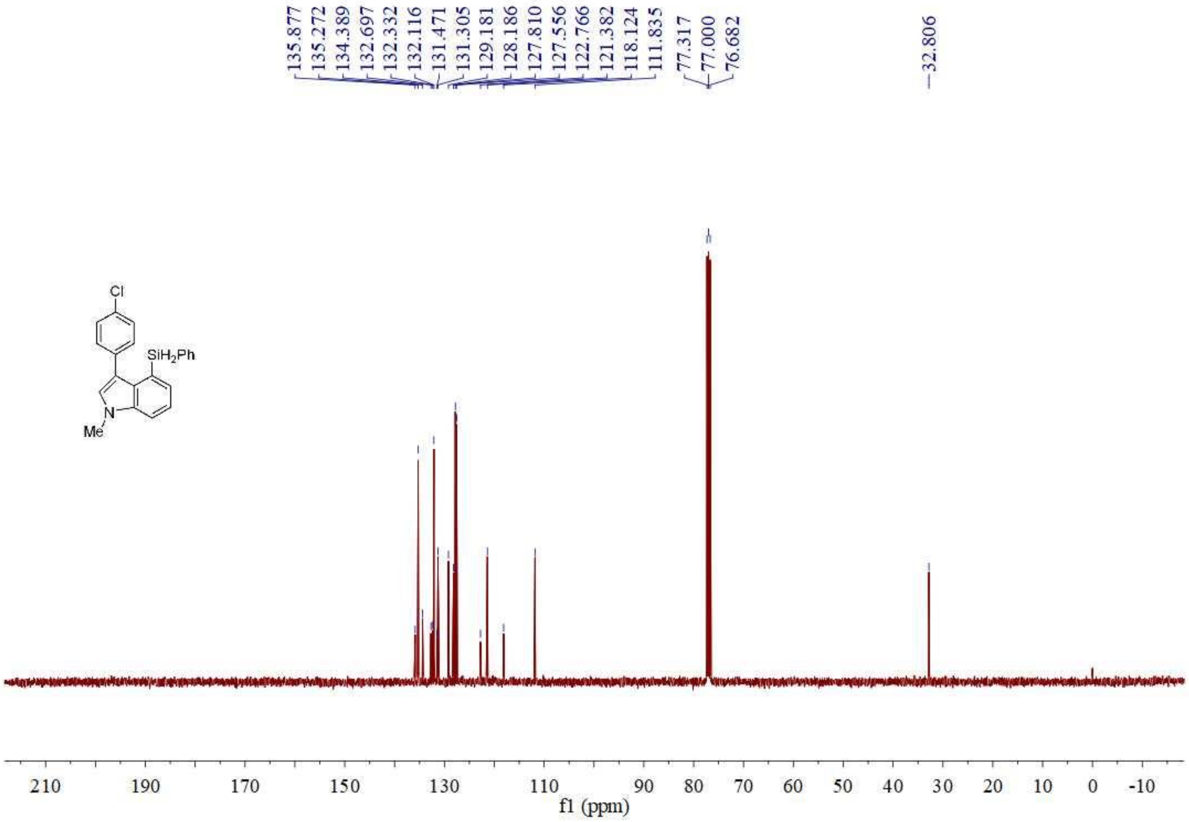
135.877
135.272
134.389
132.697
132.332
132.116
131.471
131.305
129.181
128.186
127.810
127.556
122.766
121.382
118.124
111.835
77.317
77.000
76.682
32.806
Cl
SiH2Ph
N
Me
210 190 170 150 130 110 90 80 70 60 50 40 30 20 10 0 -10
f1 (ppm)

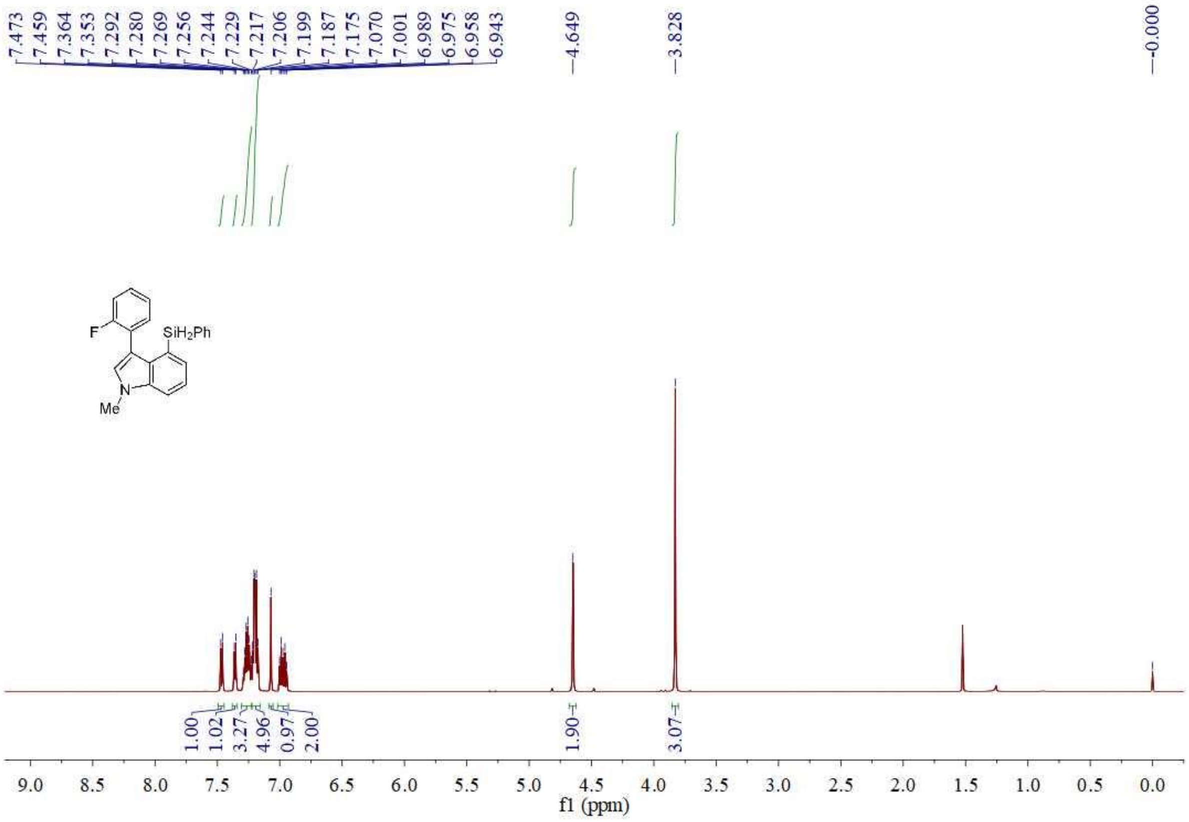
7.473
7.459
7.364
7.353
7.292
7.280
7.269
7.256
7.244
7.229
7.217
7.206
7.199
7.187
7.175
7.070
7.001
6.989
6.975
6.958
6.943
4.649
3.828
0.000
F
SiH2Ph
N
Me
1.00
1.02
3.27
4.96
0.97
2.00
1.90
3.07
9.0 8.5 8.0 7.5 7.0 6.5 6.0 5.5 5.0 4.5 4.0 3.5 3.0 2.5 2.0 1.5 1.0 0.5 0.0
f1 (ppm)

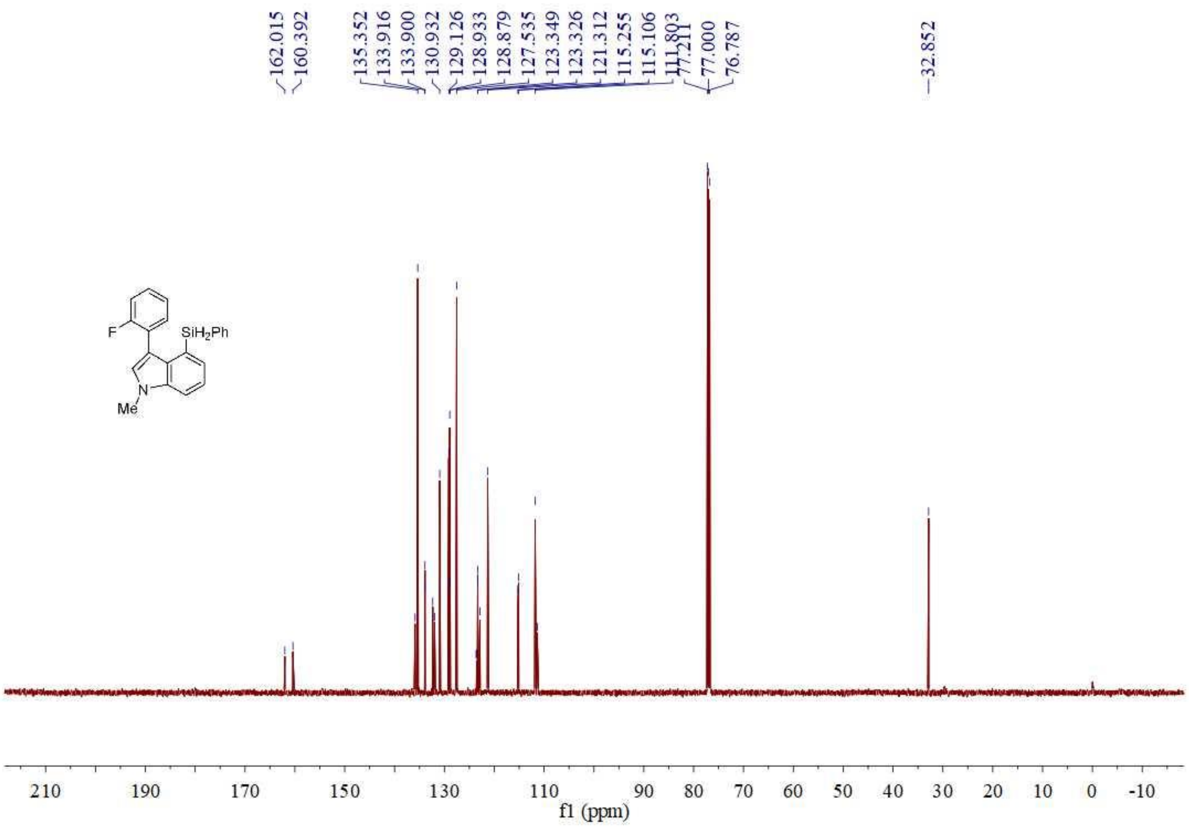
162.015
160.392
135.352
133.916
133.900
130.932
129.126
128.933
128.879
127.535
123.349
123.326
121.312
115.255
115.106
111.803
77.211
77.000
76.787
32.852
F
SiH2Ph
N
Me
210 190 170 150 130 110 90 80 70 60 50 40 30 20 10 0 -10
f1 (ppm)

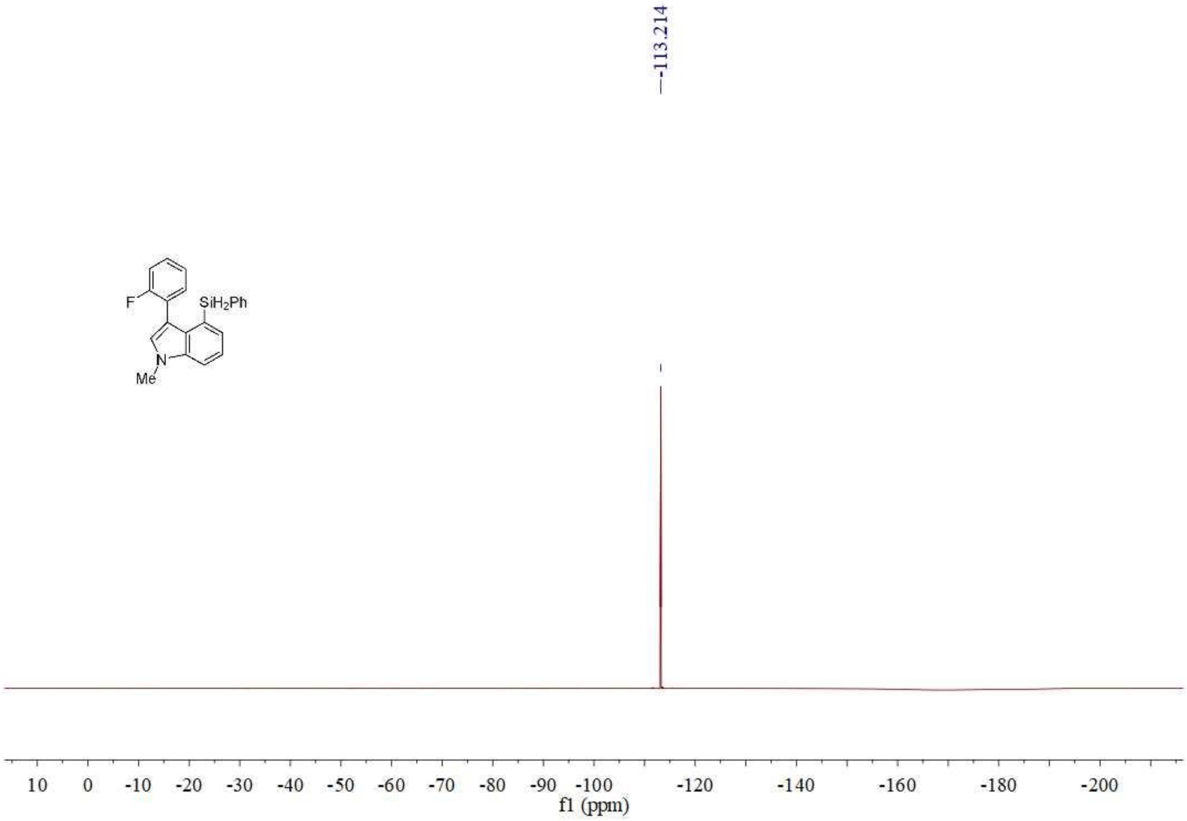

-113.214
F
SiH2Ph
N
Me
10
0
-10
-20
-30
-40
-50
-60
-70
-80
-90
-100
-120
-140
-160
-180
-200
f1 (ppm)

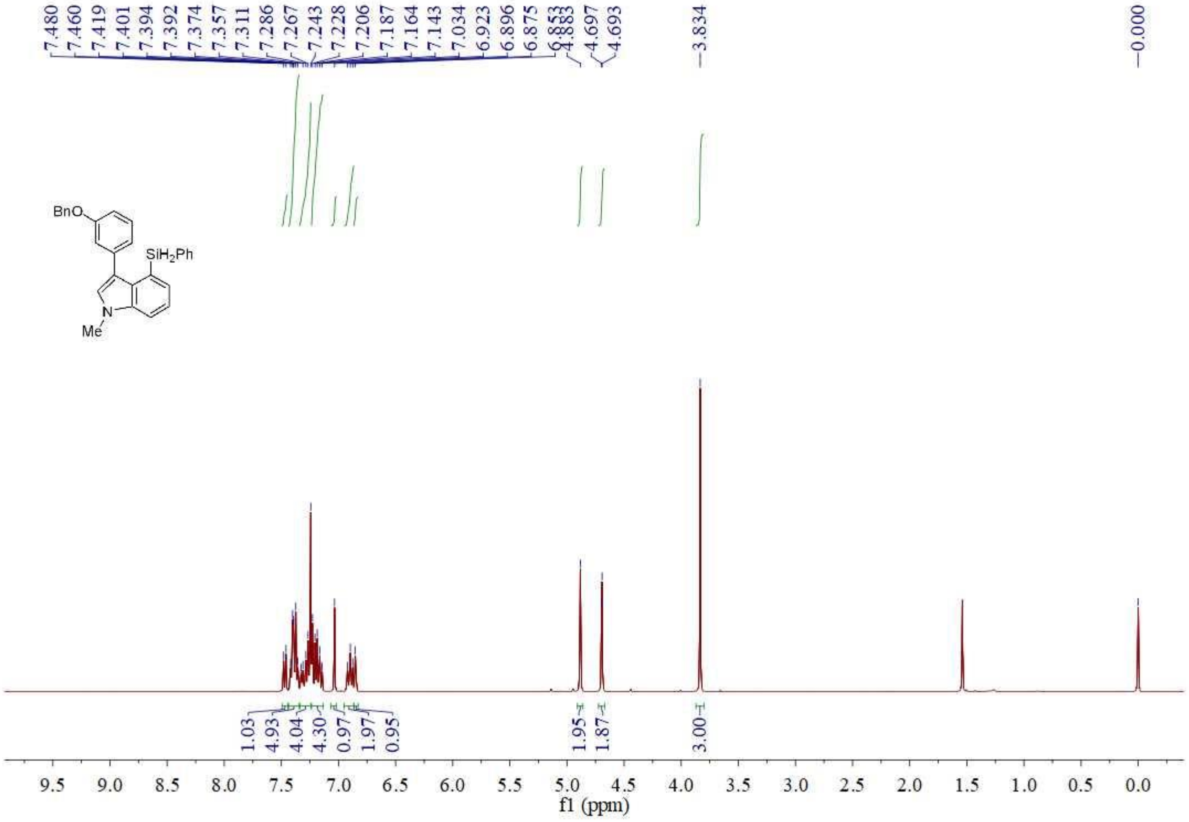

7.480
7.460
7.419
7.401
7.394
7.392
7.374
7.357
7.311
7.286
7.267
7.243
7.228
7.206
7.187
7.164
7.143
7.034
6.923
6.896
6.875
6.853
4.883
4.697
4.693
3.834
0.000
BnO
SiH2Ph
N
Me
1.03
4.93
4.04
4.30
0.97
1.97
0.95
1.95
1.87
3.00
9.5 9.0 8.5 8.0 7.5 7.0 6.5 6.0 5.5 5.0 4.5 4.0 3.5 3.0 2.5 2.0 1.5 1.0 0.5 0.0
f1 (ppm)

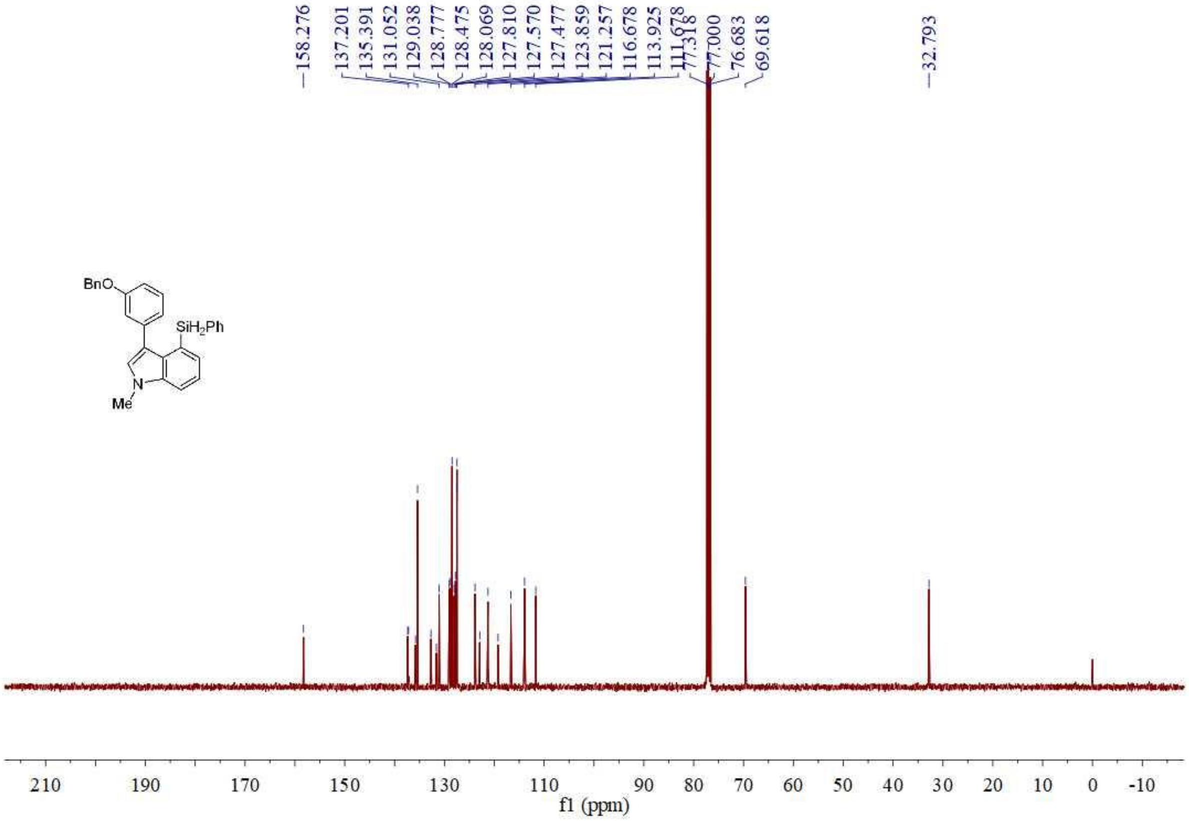

158.276
137.201
135.391
131.052
129.038
128.777
128.475
128.069
127.810
127.570
127.477
123.859
121.257
116.678
113.925
111.678
77.318
77.000
76.683
69.618
32.793
BnO
SiH2Ph
N
Me
210 190 170 150 130 110 90 80 70 60 50 40 30 20 10 0 -10
f1 (ppm)

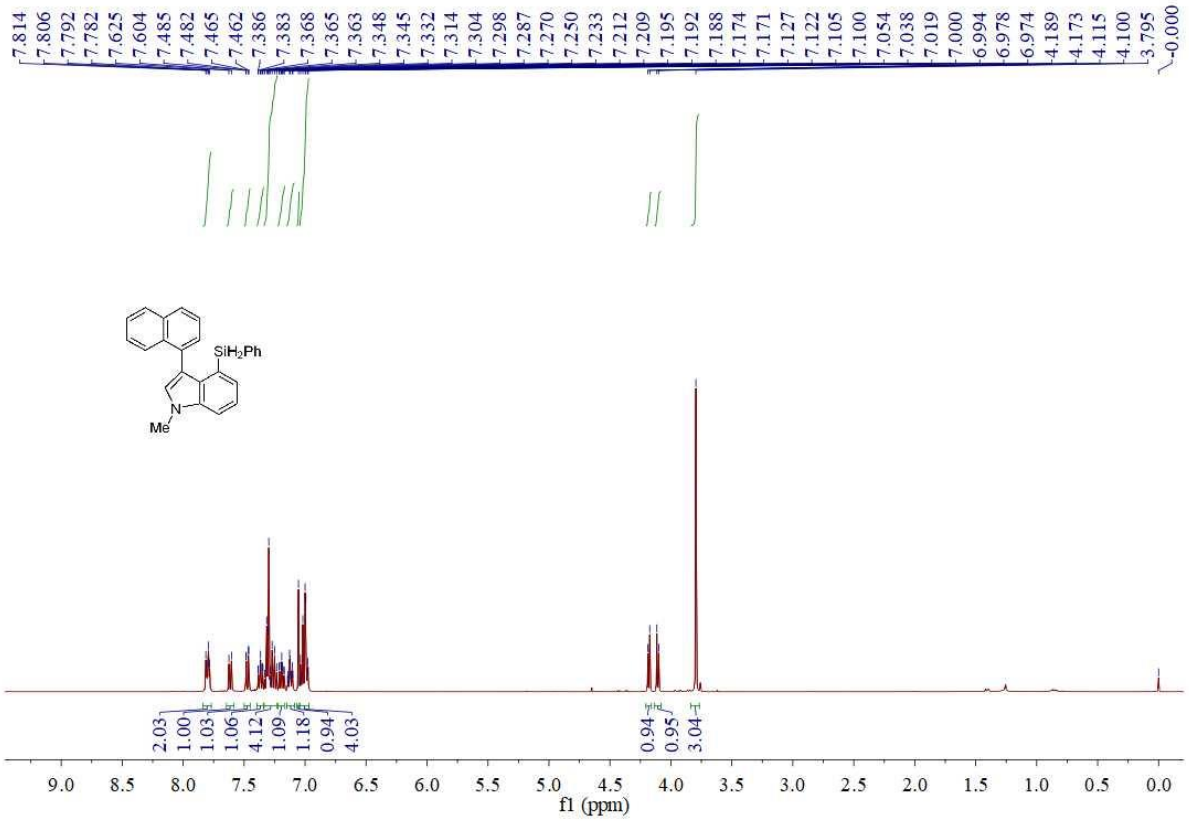
7.814 7.806 7.792 7.782 7.625 7.604 7.485 7.482 7.465 7.462 7.386 7.383 7.368 7.365 7.363 7.348 7.345 7.332 7.314 7.304 7.298 7.287 7.270 7.250 7.233 7.212 7.209 7.195 7.192 7.188 7.174 7.171 7.127 7.122 7.105 7.100 7.054 7.038 7.019 7.000 6.994 6.978 6.974 4.189 4.173 4.115 4.100 3.795 -0.000
SiH2Ph
N
Me
2.03 1.00 1.03 1.06 4.12 1.09 1.18 0.94 4.03
0.94 0.95 3.04
9.0 8.5 8.0 7.5 7.0 6.5 6.0 5.5 5.0 4.5 4.0 3.5 3.0 2.5 2.0 1.5 1.0 0.5 0.0
f1 (ppm)

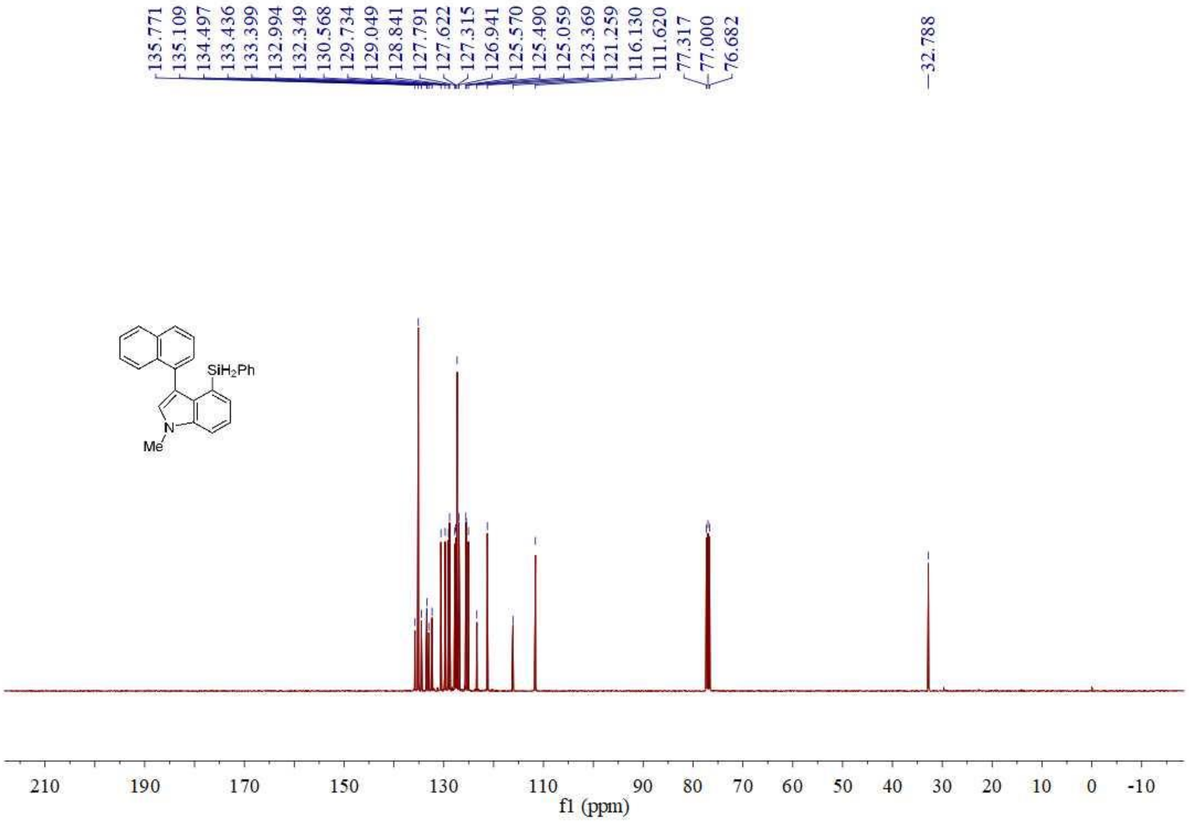
135.771 135.109 134.497 133.436 133.399 132.994 132.349 130.568 129.734 129.049 128.841 127.791 127.622 127.315 126.941 125.570 125.490 125.059 123.369 121.259 116.130 111.620 77.317 77.000 76.682
32.788
SiH2Ph
N
Me
210 190 170 150 130 110 90 80 70 60 50 40 30 20 10 0 -10
f1 (ppm)

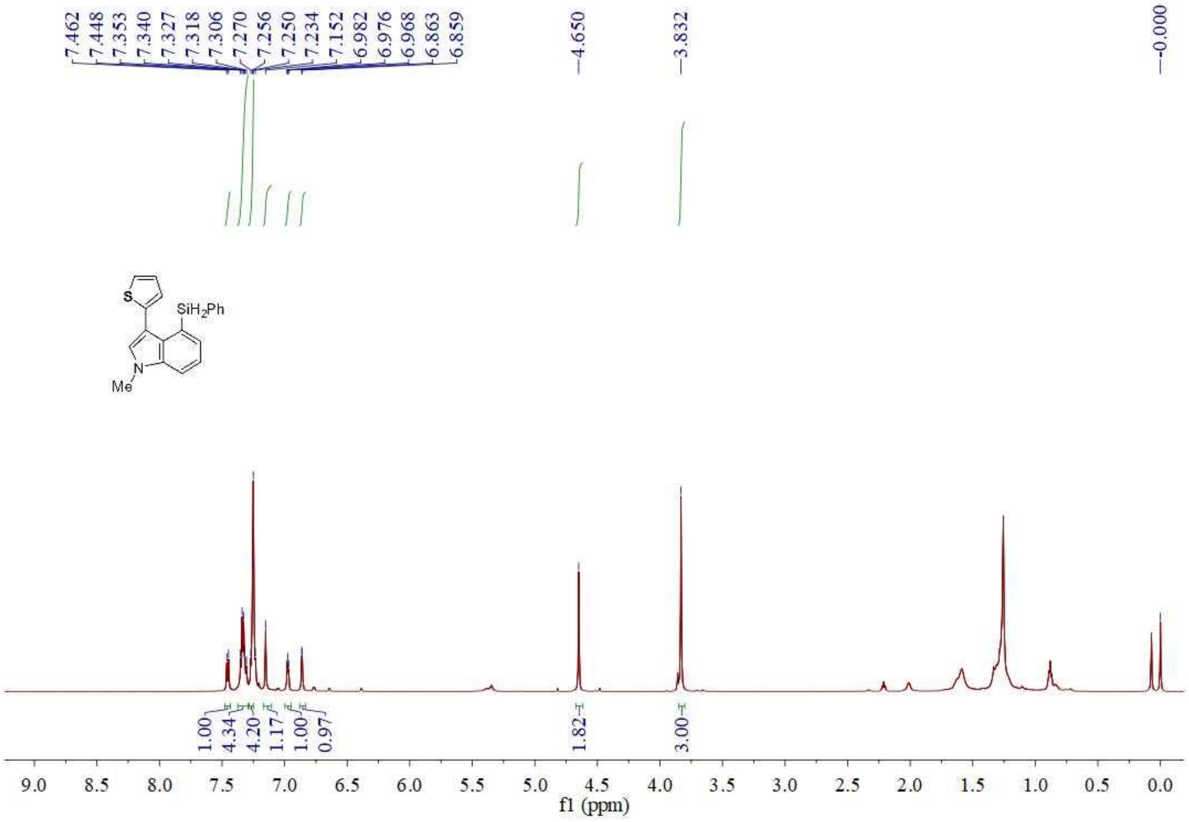

7.462
7.448
7.353
7.340
7.327
7.318
7.306
7.270
7.256
7.250
7.234
7.152
6.982
6.976
6.968
6.863
6.859
4.650
3.832
0.000
SiH2Ph
S
N
Me
1.00
4.34
4.20
1.17
1.00
0.97
1.82
3.00
9.0 8.5 8.0 7.5 7.0 6.5 6.0 5.5 5.0 4.5 4.0 3.5 3.0 2.5 2.0 1.5 1.0 0.5 0.0
f1 (ppm)

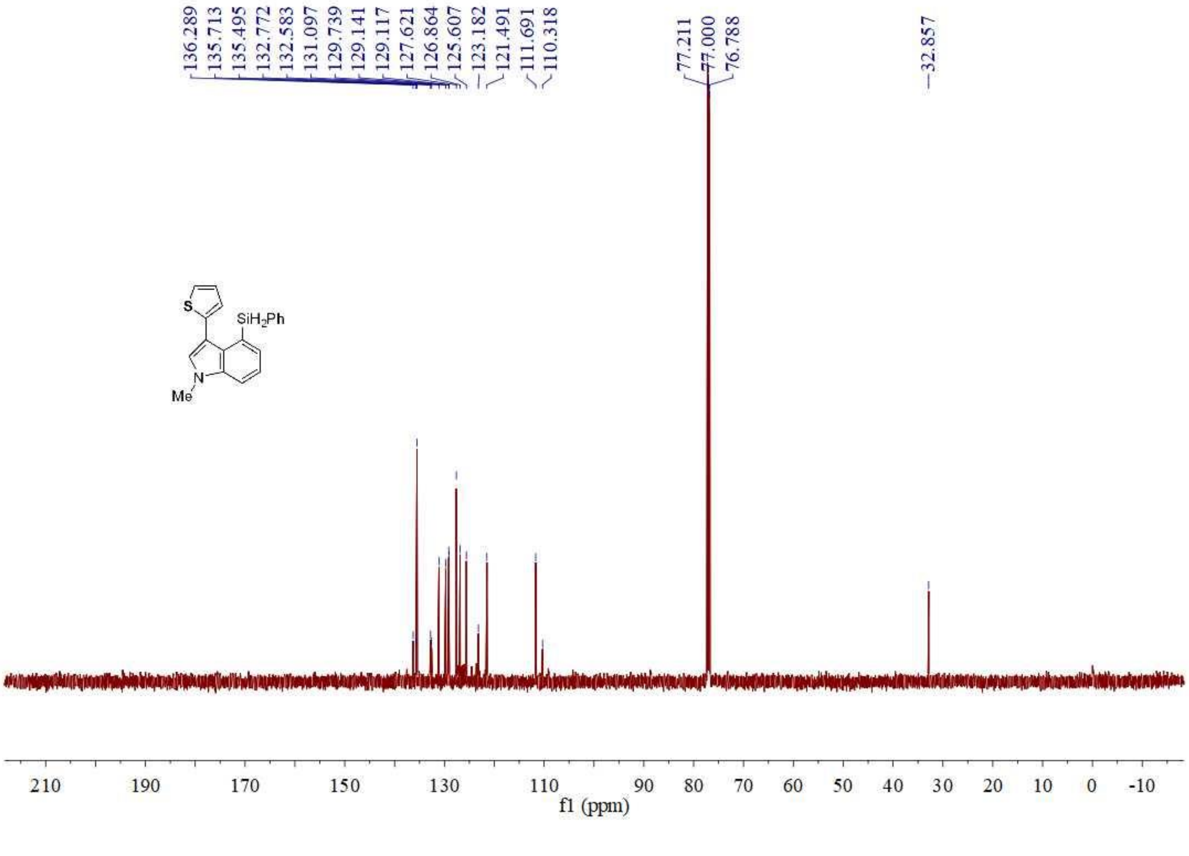

136.289
135.713
135.495
132.772
132.583
131.097
129.739
129.141
129.117
127.621
126.864
125.607
123.182
121.491
111.691
110.318
77.211
77.000
76.788
32.857
SiH2Ph
S
N
Me
210 190 170 150 130 110 90 80 70 60 50 40 30 20 10 0 -10
f1 (ppm)

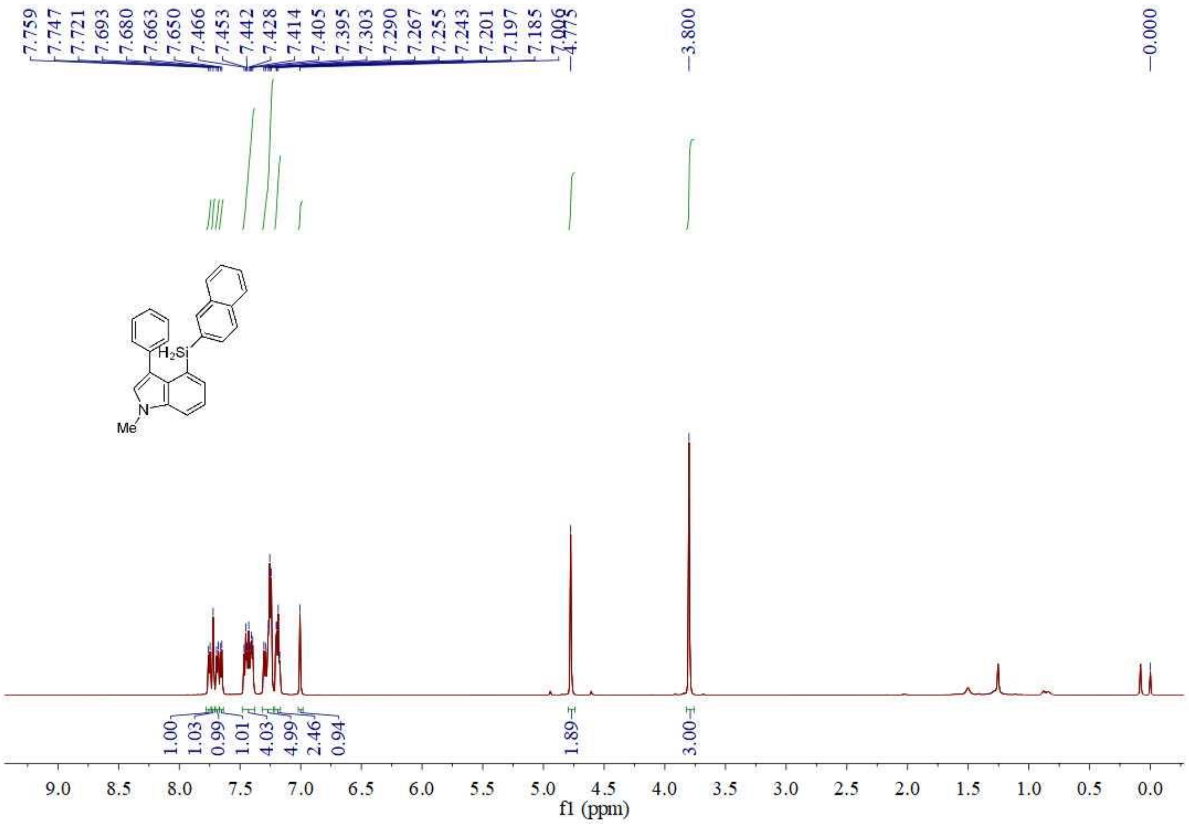

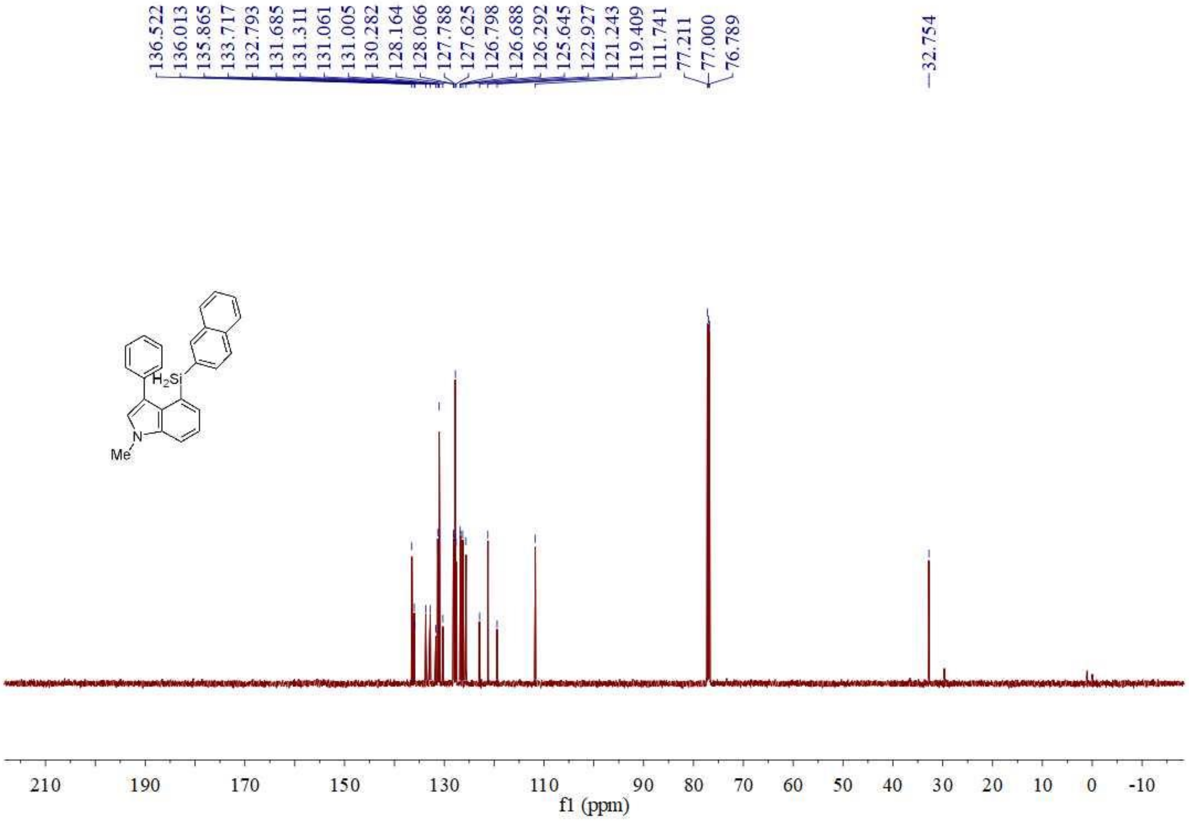

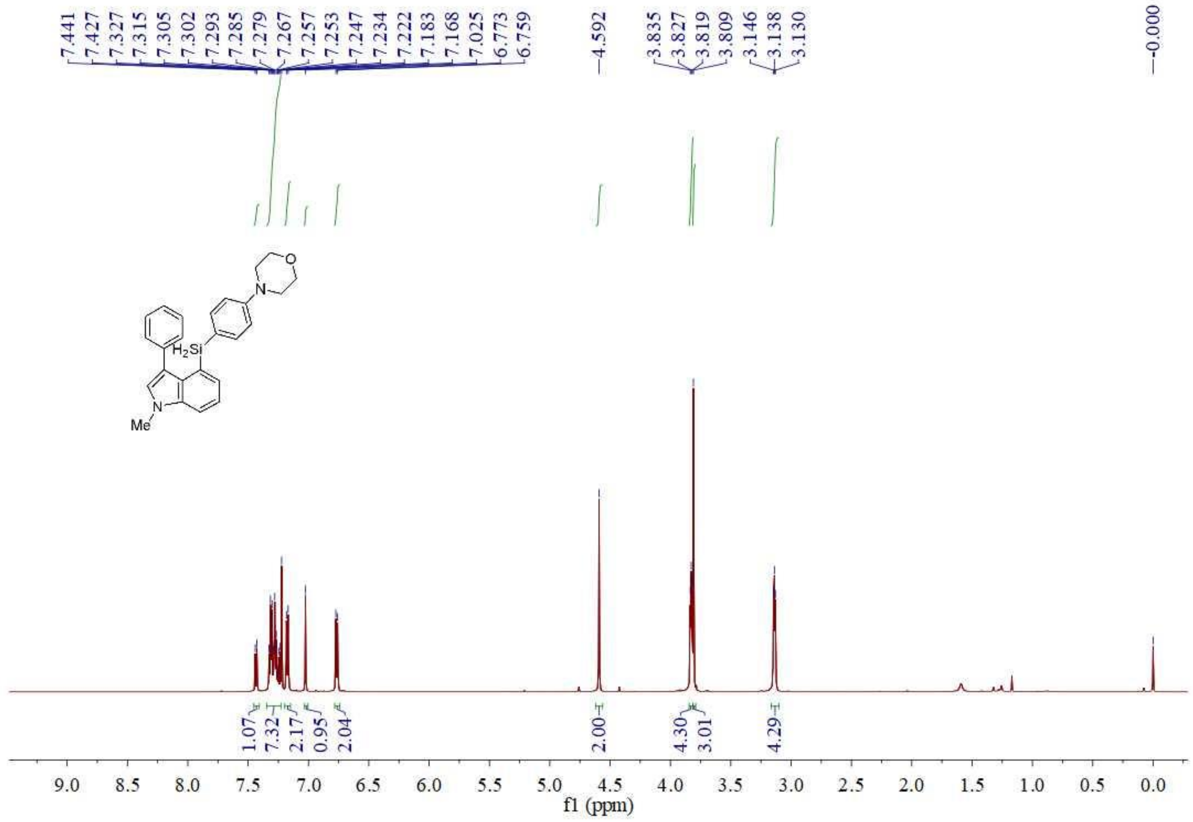
7.441 7.427 7.327 7.315 7.305 7.302 7.293 7.285 7.279 7.267 7.257 7.253 7.247 7.234 7.222 7.183 7.168 7.025 6.773 6.759
4.592
3.835 3.827 3.819 3.809 3.146 3.138 3.130
0.000
H_2Si
N
O
Me
1.07 7.32 2.17 0.95 2.04
2.00
4.30 3.01
4.29
9.0 8.5 8.0 7.5 7.0 6.5 6.0 5.5 5.0 4.5 4.0 3.5 3.0 2.5 2.0 1.5 1.0 0.5 0.0
f1 (ppm)

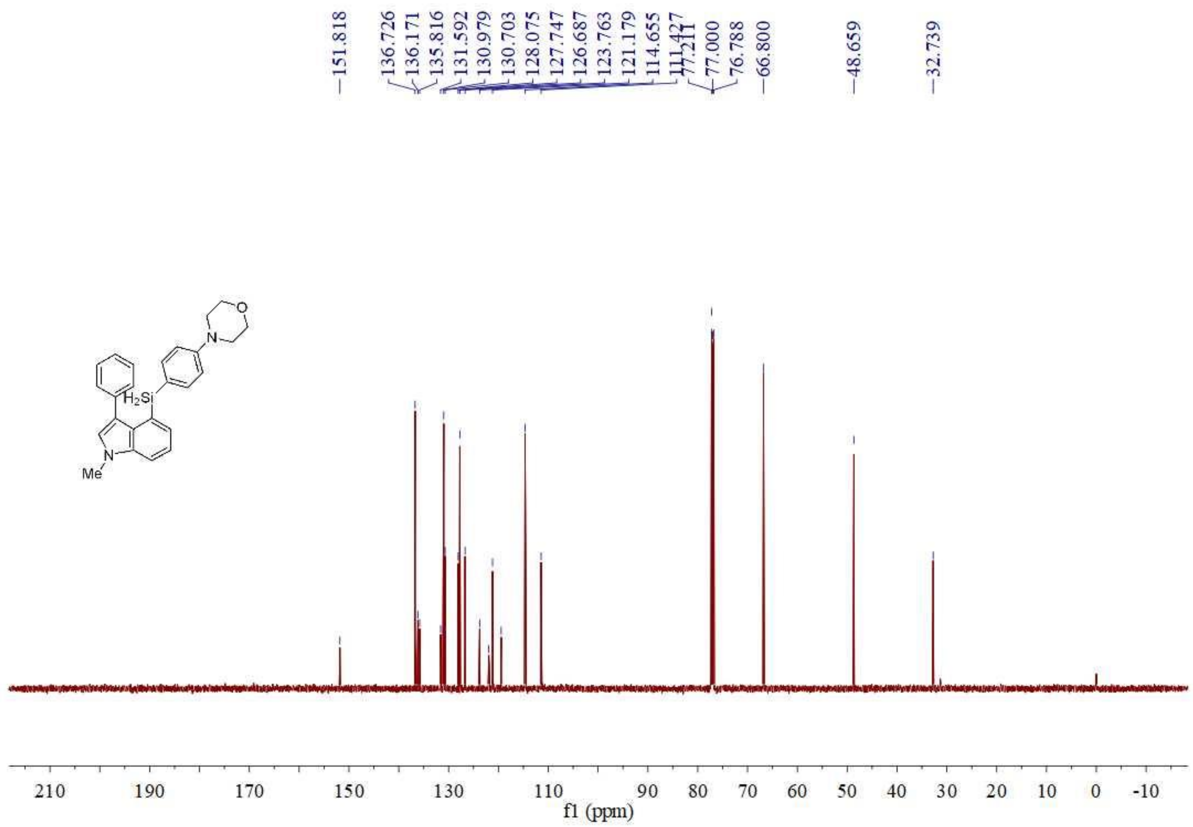
151.818
136.726 136.171 135.816 131.592 130.979 130.703 128.075 127.747 126.687 123.763 121.179 114.655 111.427 77.211 77.000 76.788
66.800
48.659
32.739
H_2Si
N
O
Me
210 190 170 150 130 110 90 80 70 60 50 40 30 20 10 0 -10
f1 (ppm)

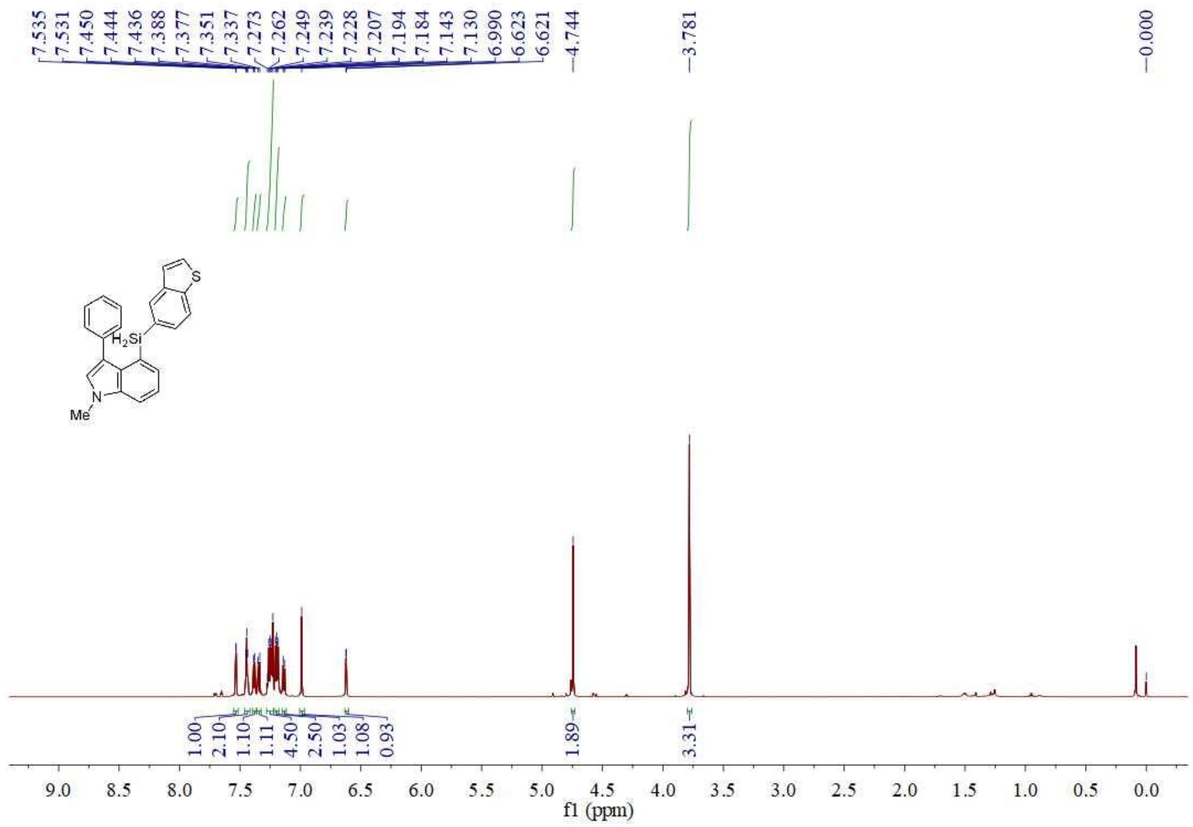

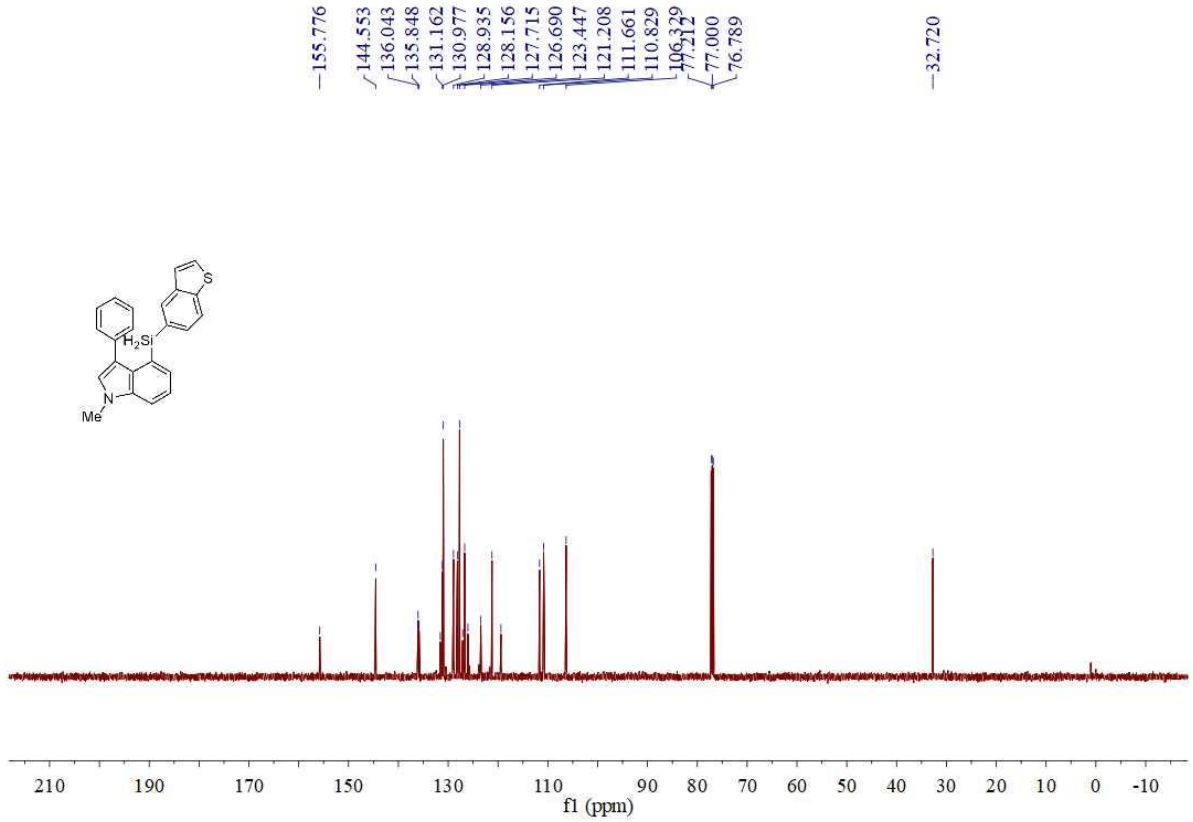

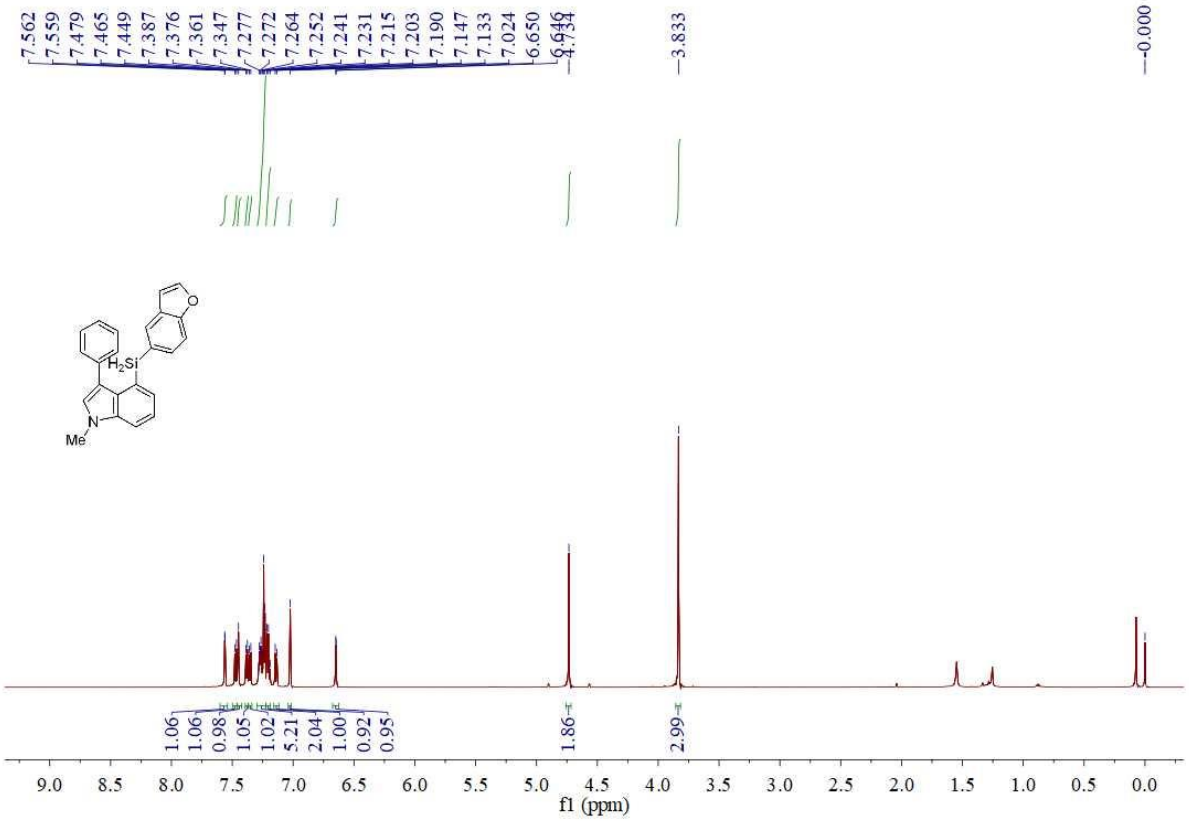
7.562
7.559
7.479
7.465
7.449
7.387
7.376
7.361
7.347
7.277
7.272
7.264
7.252
7.241
7.231
7.215
7.203
7.190
7.147
7.133
7.024
6.650
6.646
4.734
3.833
0.000
H$_2$Si
N
Me
O
1.06
1.06
0.98
1.05
1.02
5.21
2.04
1.00
0.92
0.95
1.86
2.99
f1 (ppm)

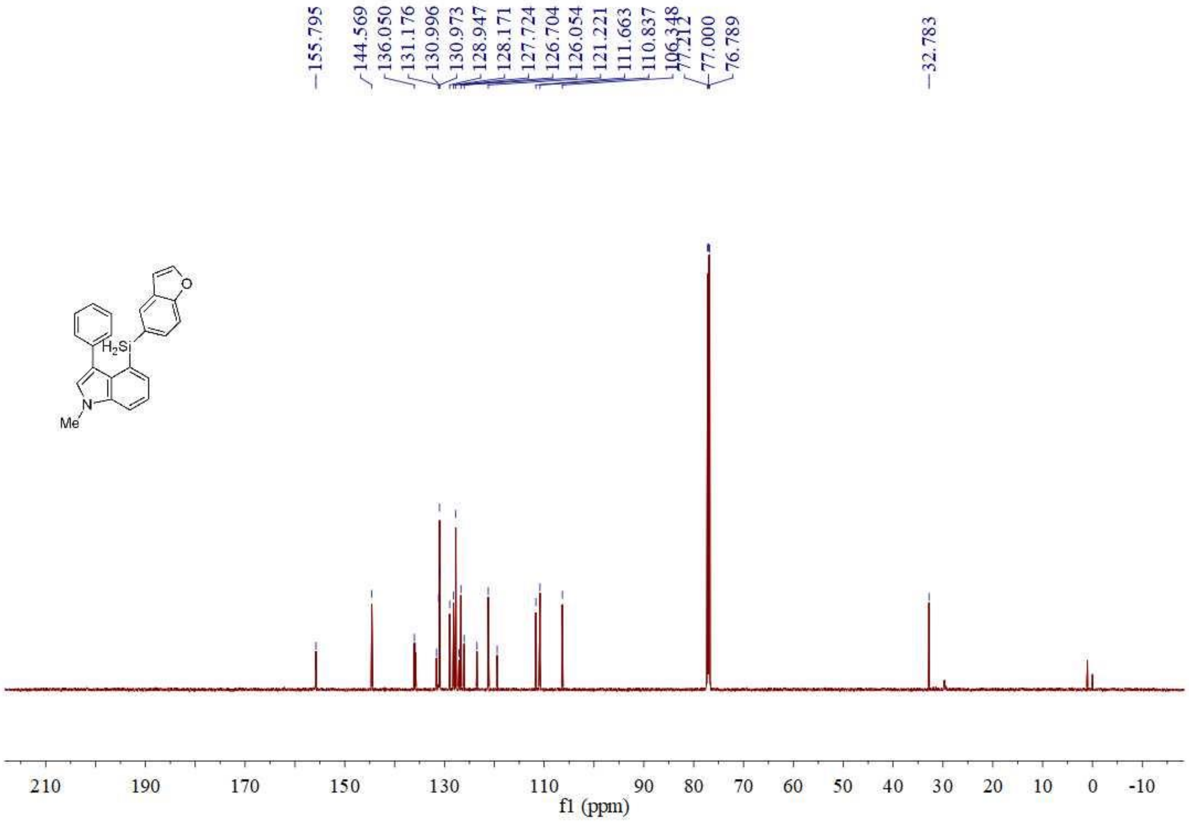
155.795
144.569
136.050
131.176
130.996
130.973
128.947
128.171
127.724
126.704
126.054
121.221
111.663
110.837
106.348
77.212
77.000
76.789
32.783
H$_2$Si
N
Me
O
f1 (ppm)

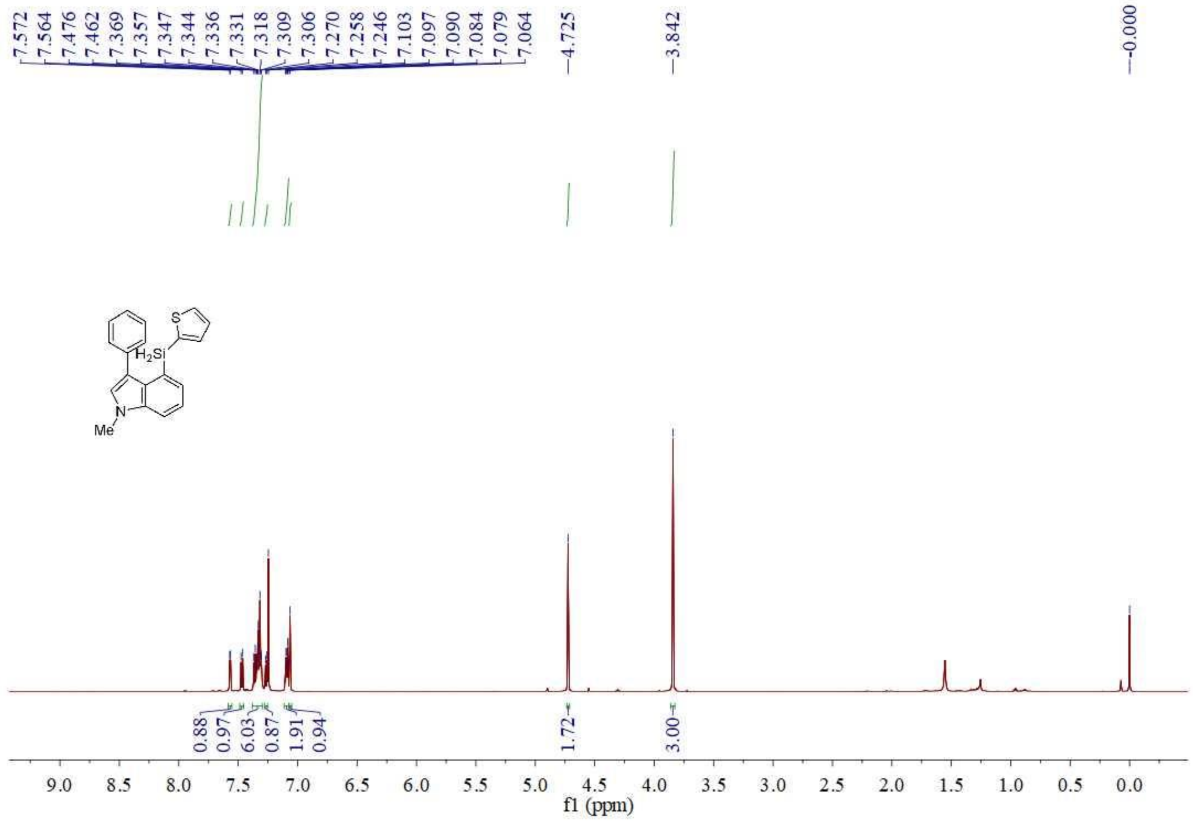

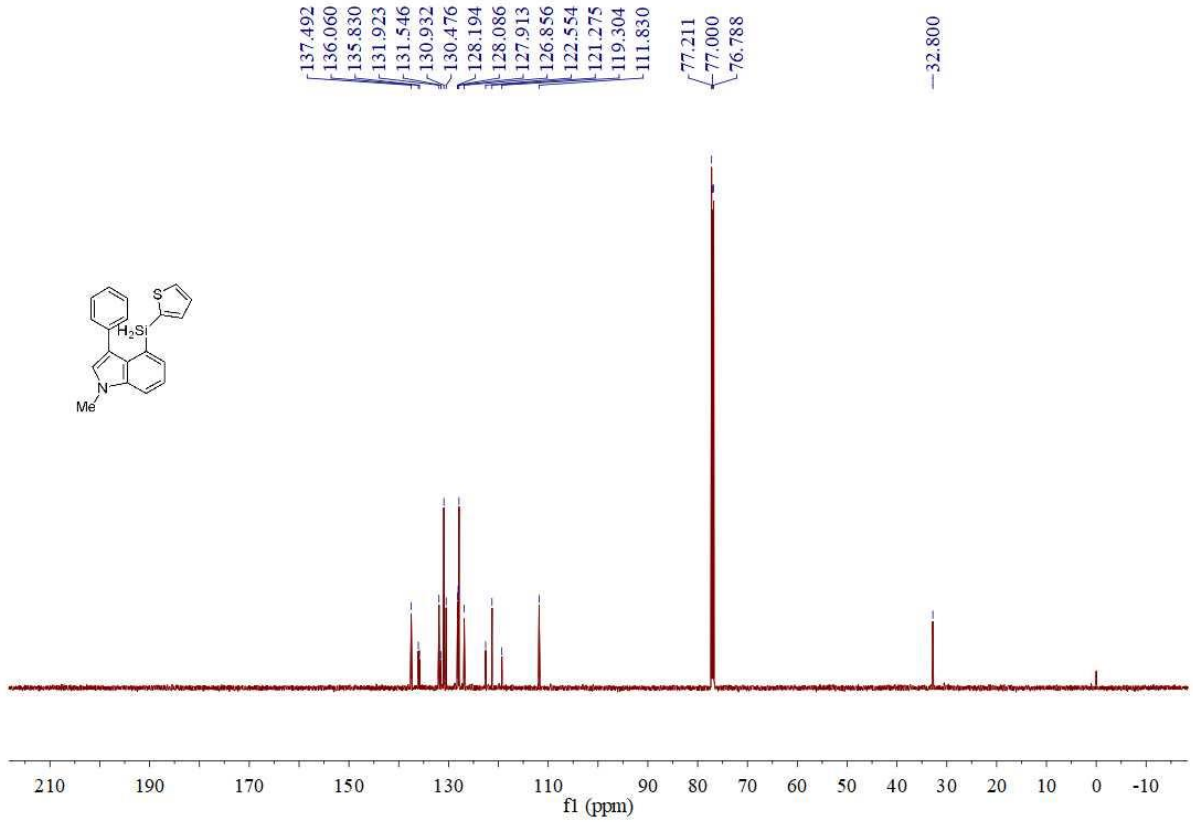

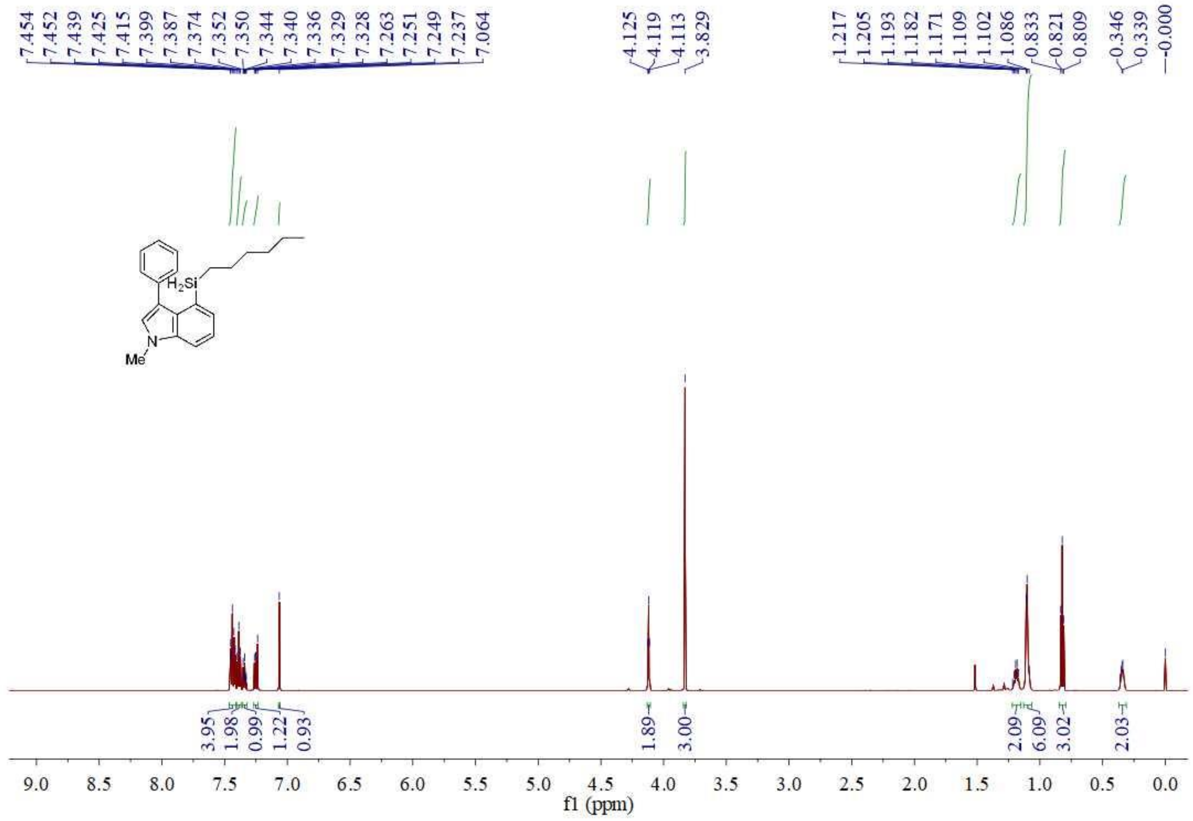
7.454 7.452 7.439 7.425 7.415 7.399 7.387 7.374 7.352 7.350 7.344 7.340 7.336 7.329 7.328 7.263 7.251 7.249 7.237 7.064
4.125 4.119 4.113 3.829
1.217 1.205 1.193 1.182 1.171 1.109 1.102 1.086 0.833 0.821 0.809
0.346 0.339 0.000
H2Si
N
Me
3.95 1.98 0.99 1.22 0.93
1.89 3.00
2.09 6.09 3.02
2.03
f1 (ppm)

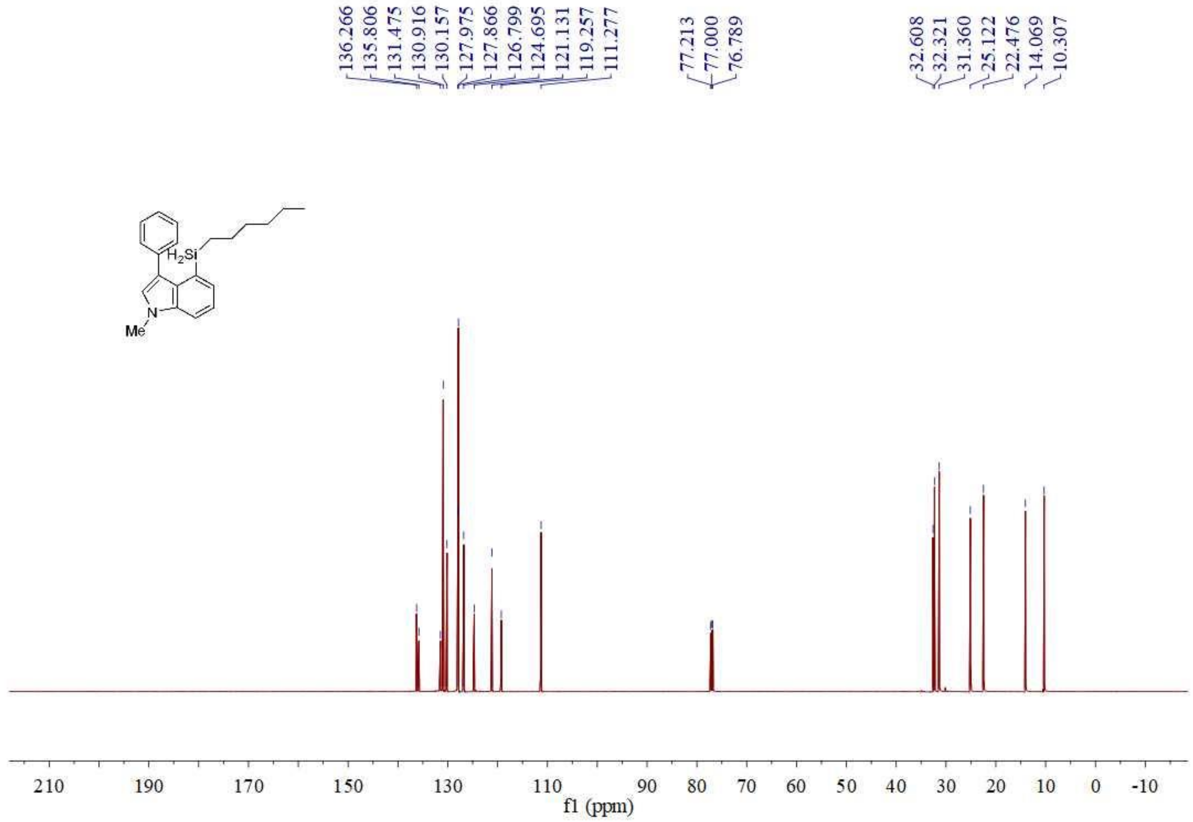
136.266 135.806 131.475 130.916 130.157 127.975 127.866 126.799 124.695 121.131 119.257 111.277
77.213 77.000 76.789
32.608 32.321 31.360 25.122 22.476 14.069 10.307
H2Si
N
Me
f1 (ppm)

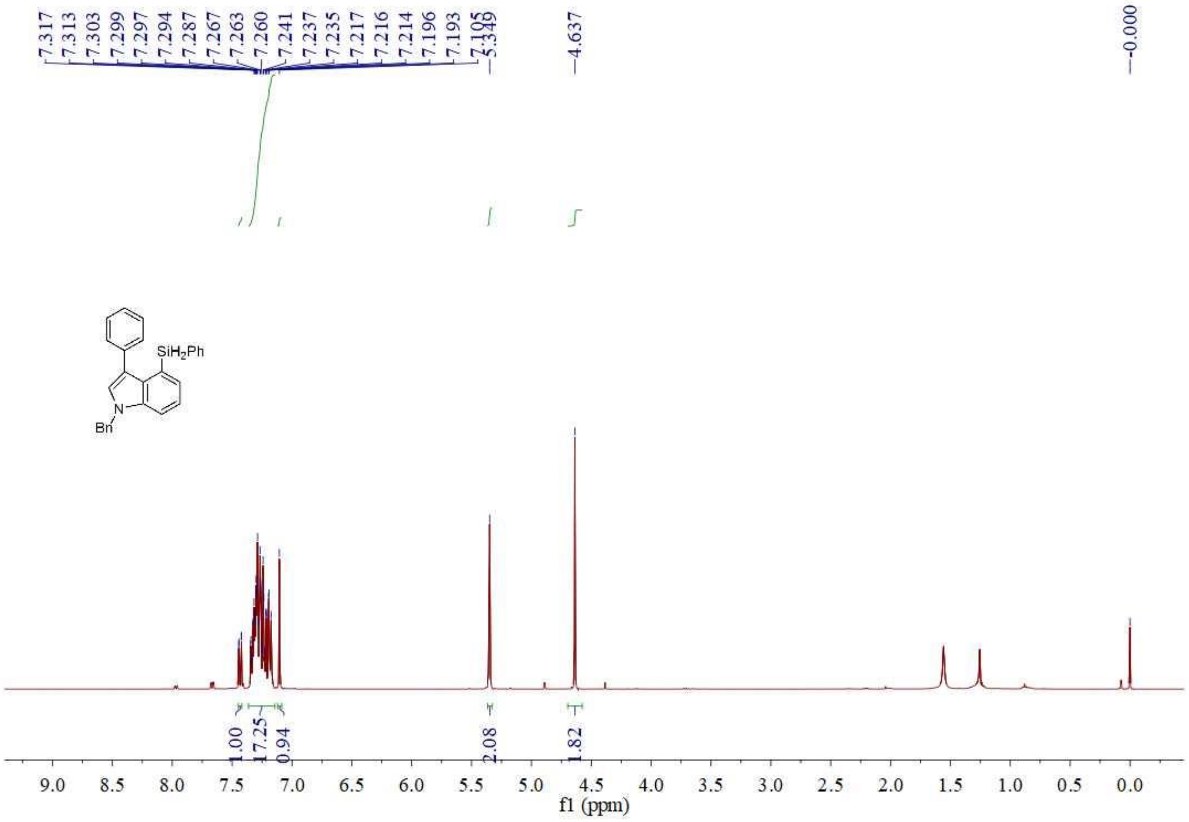
7.317
7.313
7.303
7.299
7.297
7.294
7.287
7.267
7.263
7.260
7.241
7.237
7.235
7.217
7.216
7.214
7.196
7.193
5.349
4.637
0.000
SiH2Ph
N
Bn
1.00
17.25
0.94
2.08
1.82
9.0 8.5 8.0 7.5 7.0 6.5 6.0 5.5 5.0 4.5 4.0 3.5 3.0 2.5 2.0 1.5 1.0 0.5 0.0
f1 (ppm)

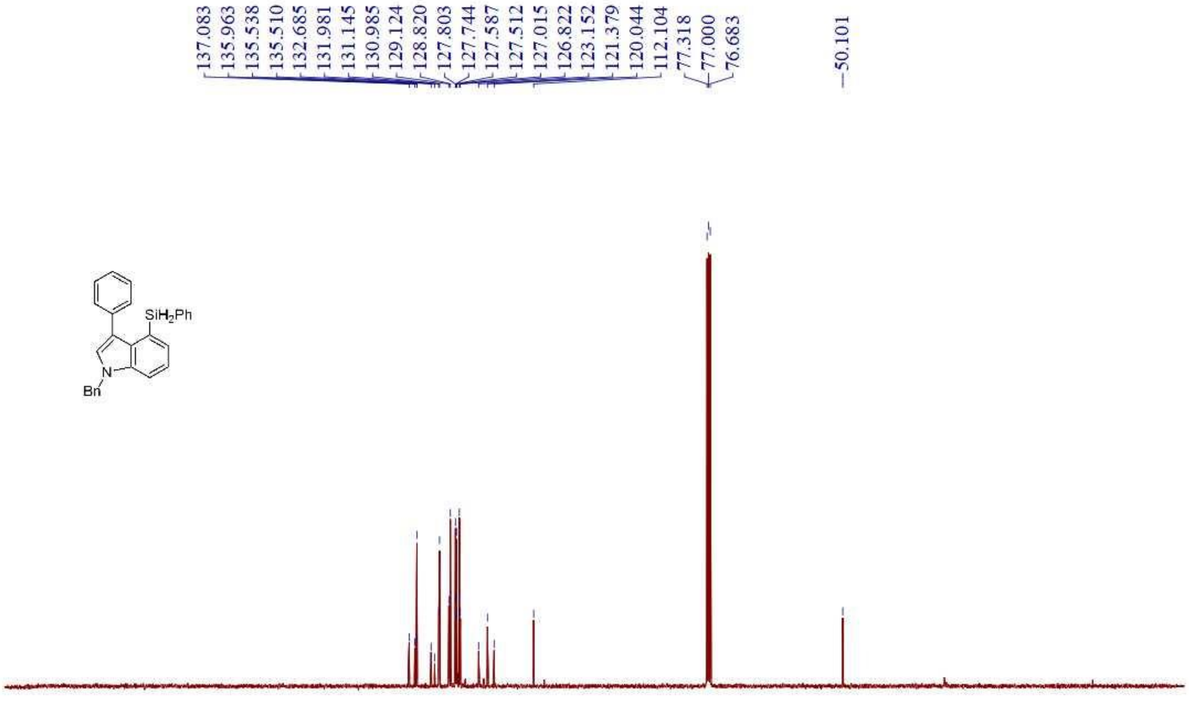
137.083
135.963
135.538
135.510
132.685
131.981
131.145
130.985
129.124
128.820
127.803
127.744
127.587
127.512
127.015
126.822
123.152
121.379
120.044
112.104
77.318
77.000
76.683
50.101
SiH2Ph
N
Bn
210 190 170 150 130 110 90 80 70 60 50 40 30 20 10 0 -10
f1 (ppm)

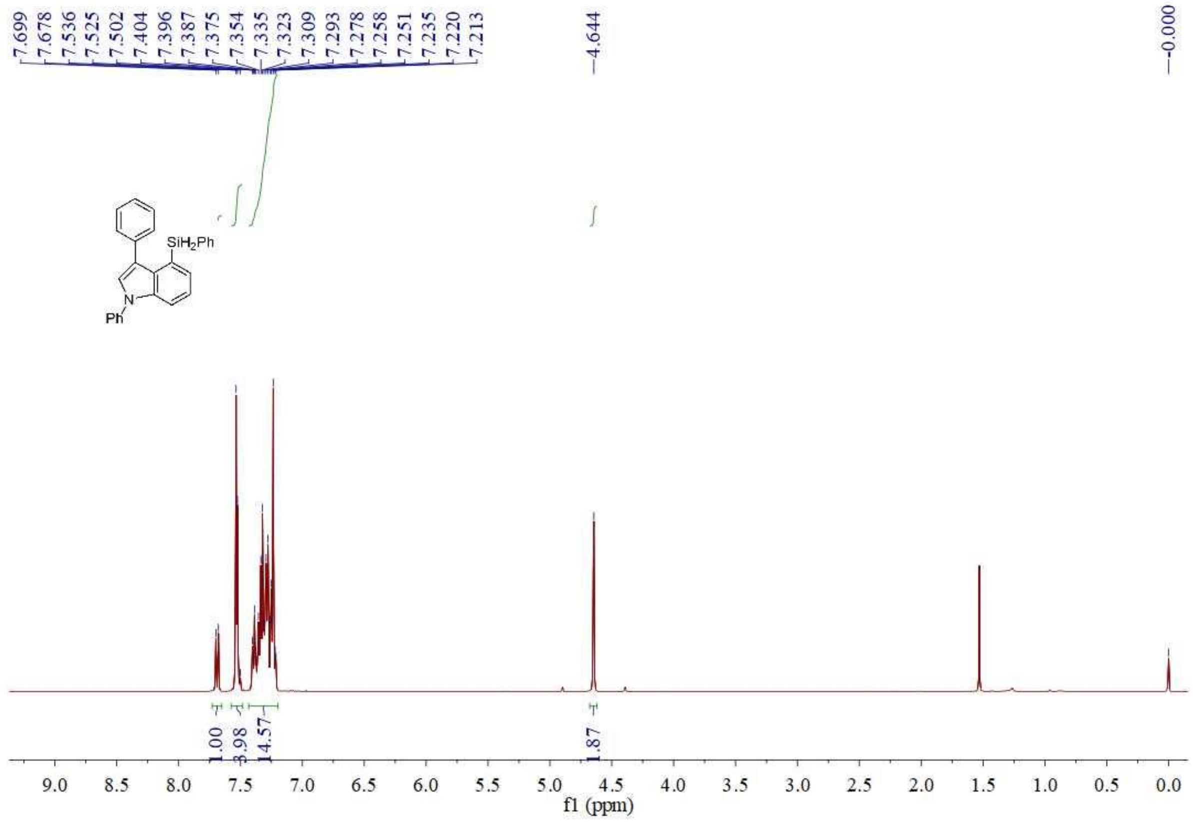

7.699
7.678
7.536
7.525
7.502
7.404
7.396
7.387
7.375
7.354
7.335
7.323
7.309
7.293
7.278
7.258
7.251
7.235
7.220
7.213
4.644
0.000
SiH2Ph
N
Ph
1.00
3.98
14.57
1.87
9.0 8.5 8.0 7.5 7.0 6.5 6.0 5.5 5.0 4.5 4.0 3.5 3.0 2.5 2.0 1.5 1.0 0.5 0.0
f1 (ppm)

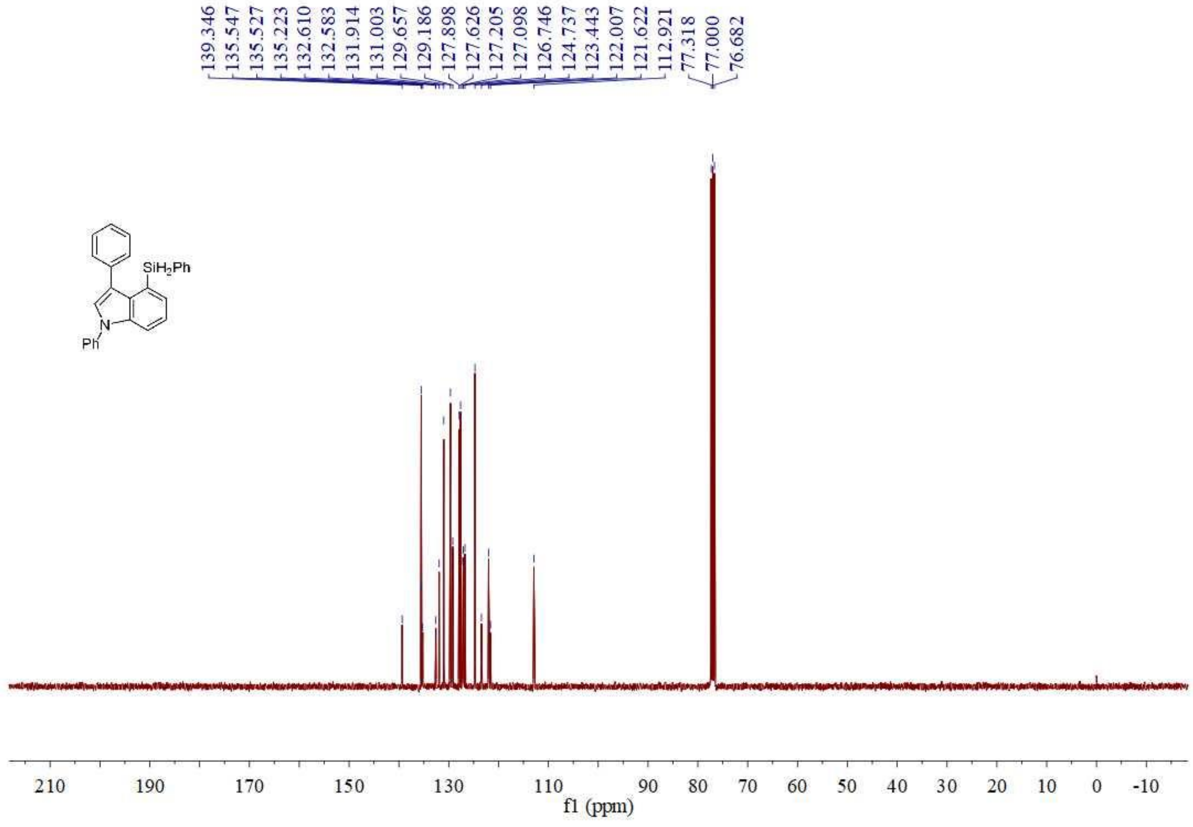

139.346
135.547
135.527
135.223
132.610
132.583
131.914
131.003
129.657
129.186
127.898
127.626
127.205
127.098
126.746
124.737
123.443
122.007
121.622
112.921
77.318
77.000
76.682
SiH2Ph
N
Ph
210 190 170 150 130 110 90 80 70 60 50 40 30 20 10 0 -10
f1 (ppm)

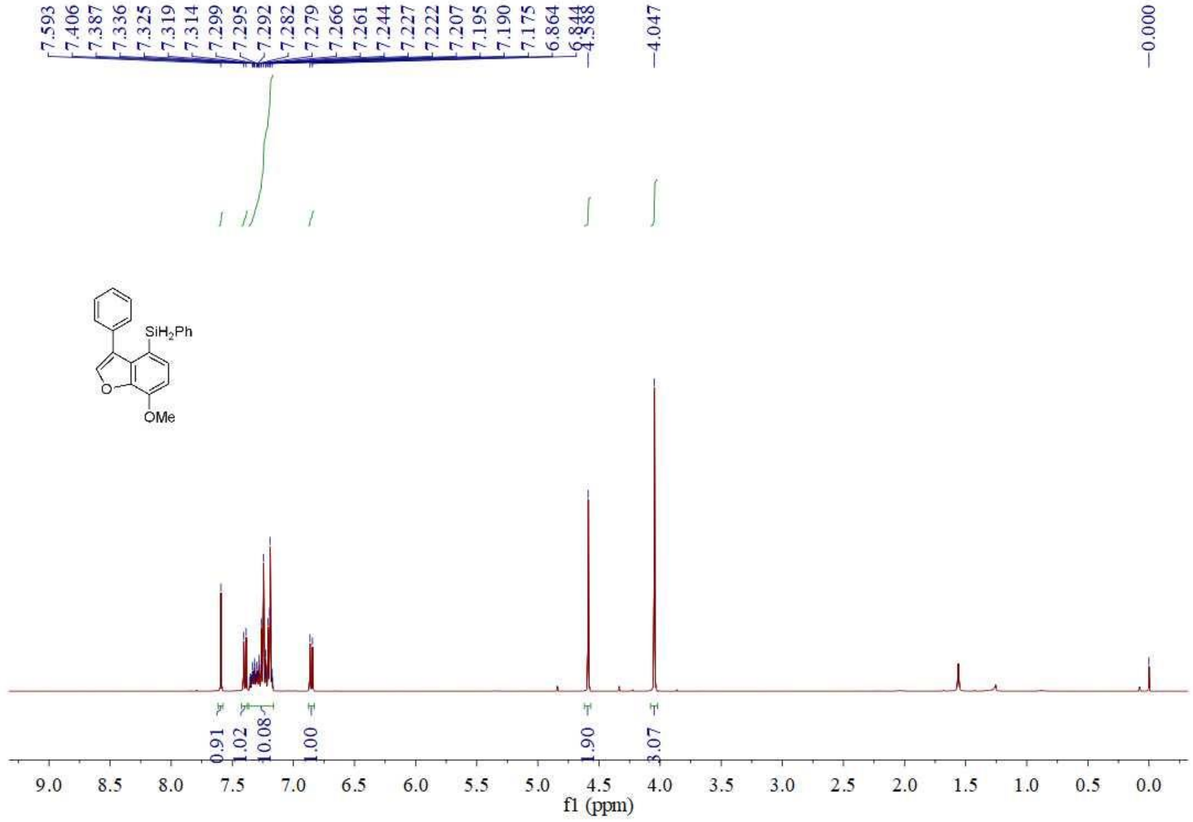
7.593
7.406
7.387
7.336
7.325
7.319
7.314
7.299
7.295
7.292
7.282
7.279
7.266
7.261
7.244
7.227
7.222
7.207
7.195
7.190
7.175
6.864
6.844
4.588
4.047
0.000
SiH2Ph
OMe
0.91
1.02
10.08
1.00
1.90
3.07
f1 (ppm)

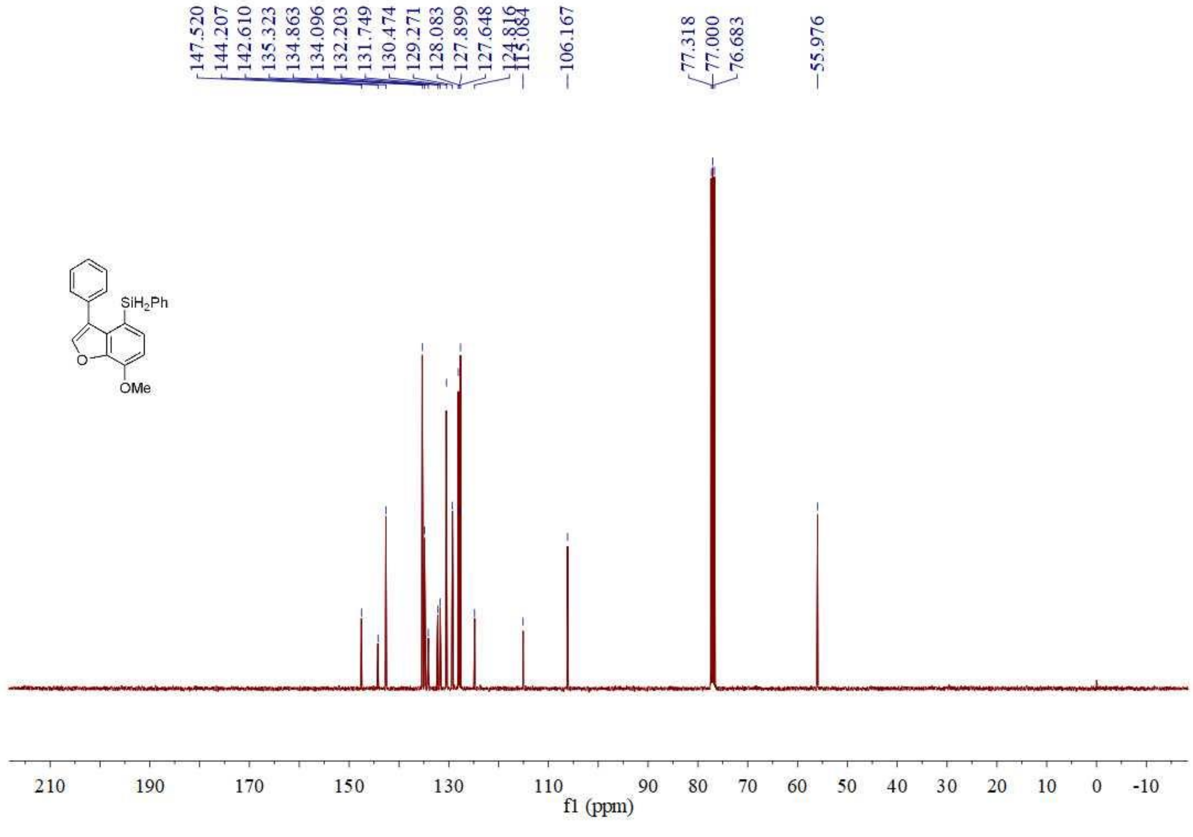
147.520
144.207
142.610
135.323
134.863
134.096
132.203
131.749
130.474
129.271
128.083
127.899
127.648
124.816
115.084
106.167
77.318
77.000
76.683
55.976
SiH2Ph
OMe
f1 (ppm)

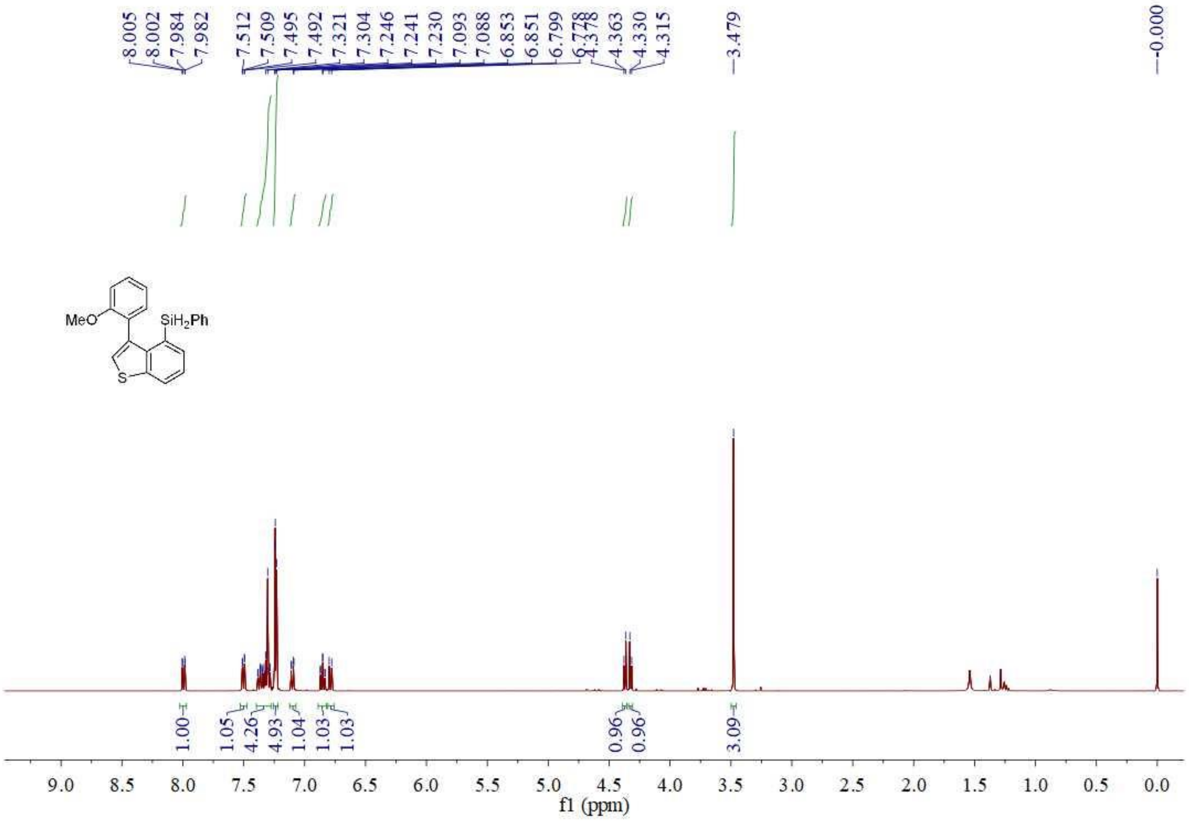
8.005
8.002
7.984
7.982
7.512
7.509
7.495
7.492
7.321
7.304
7.246
7.241
7.230
7.093
7.088
6.853
6.851
6.799
6.778
4.378
4.363
4.330
4.315
3.479
0.000
MeO
SiH2Ph
S
1.00
1.05
4.26
4.93
1.04
1.03
1.03
0.96
0.96
3.09
9.0 8.5 8.0 7.5 7.0 6.5 6.0 5.5 5.0 4.5 4.0 3.5 3.0 2.5 2.0 1.5 1.0 0.5 0.0
f1 (ppm)

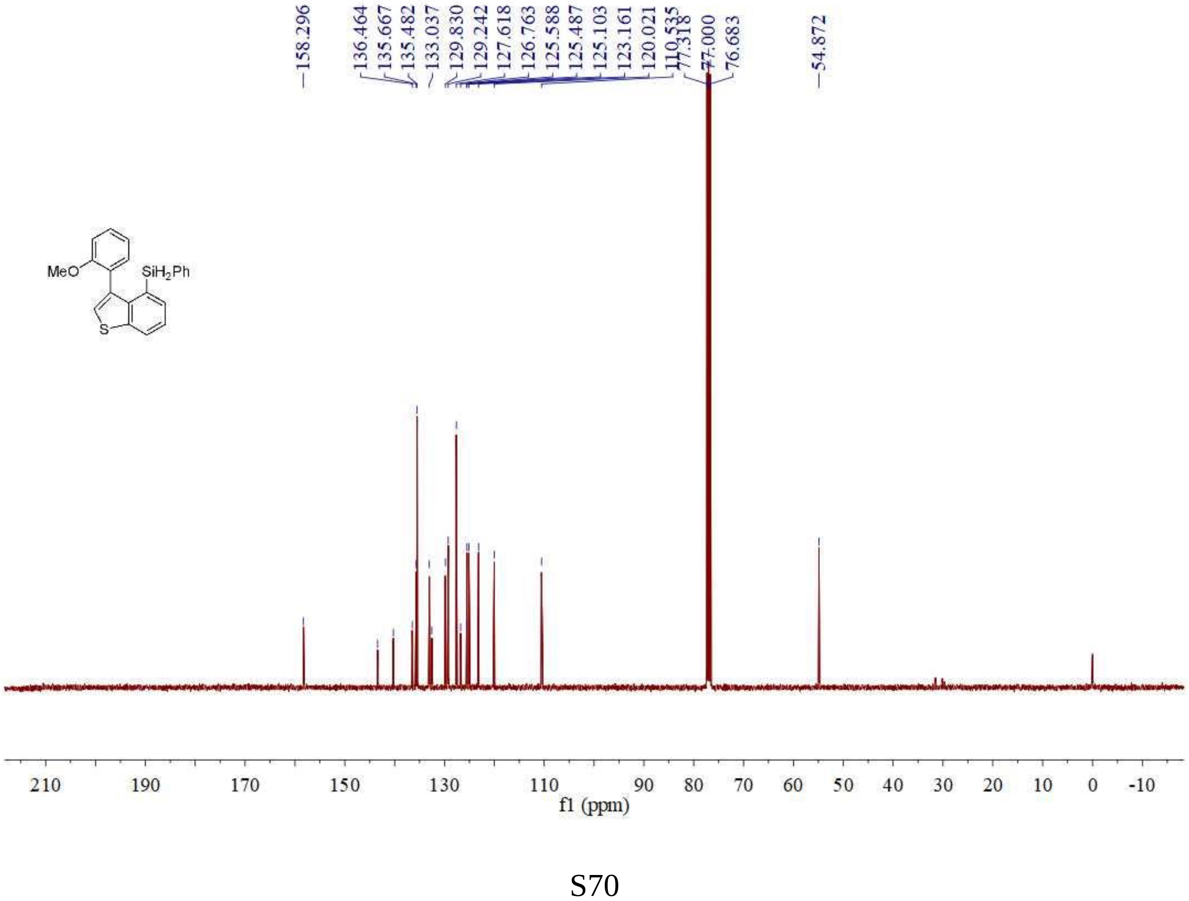
158.296
136.464
135.667
135.482
133.037
129.830
129.242
127.618
126.763
125.588
125.487
125.103
123.161
120.021
110.535
77.318
77.000
76.683
54.872
MeO
SiH2Ph
S
210 190 170 150 130 110 90 80 70 60 50 40 30 20 10 0 -10
f1 (ppm)

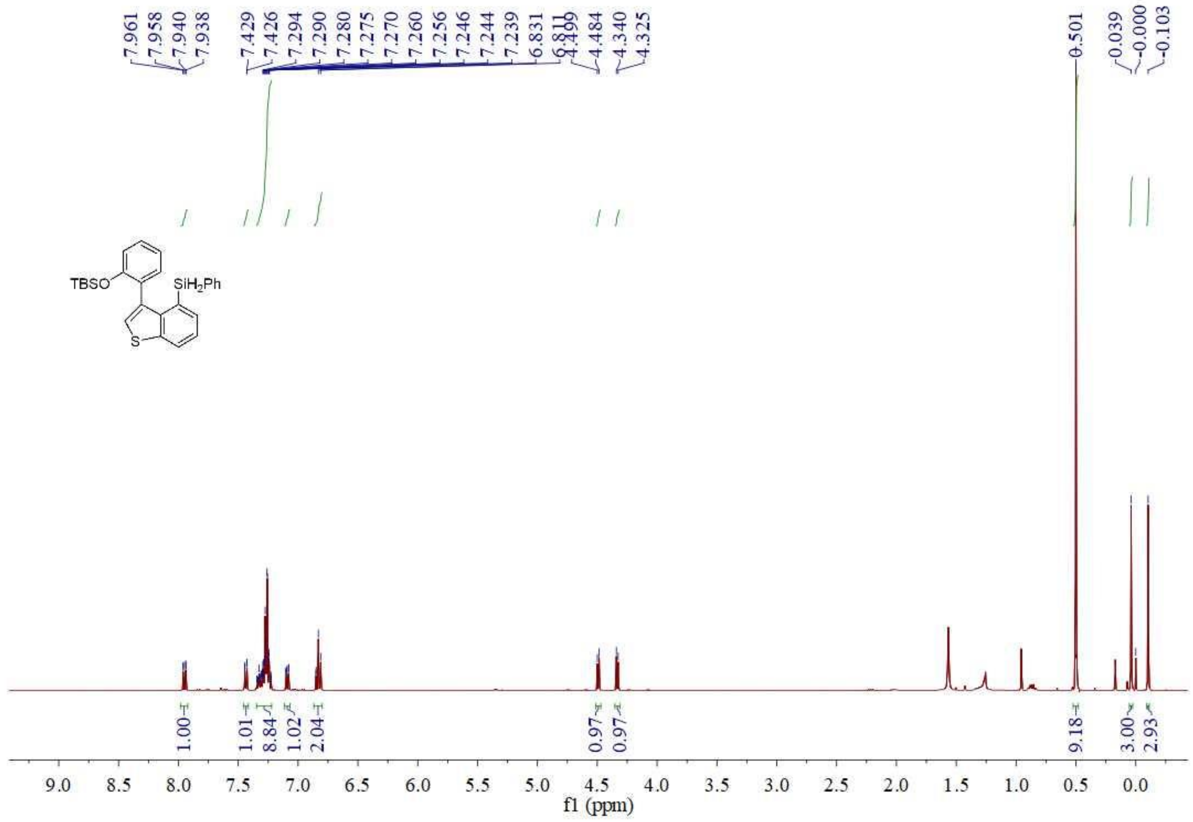
7.961
7.958
7.940
7.938
7.429
7.426
7.294
7.290
7.280
7.275
7.270
7.260
7.256
7.246
7.244
7.239
6.831
6.811
4.499
4.484
4.340
4.325
0.501
0.039
-0.000
-0.103
TBSO
SiH2Ph
S
1.00
1.01
8.84
1.02
2.04
0.97
0.97
9.18
3.00
2.93
9.0
8.5
8.0
7.5
7.0
6.5
6.0
5.5
5.0
4.5
4.0
3.5
3.0
2.5
2.0
1.5
1.0
0.5
0.0
f1 (ppm)

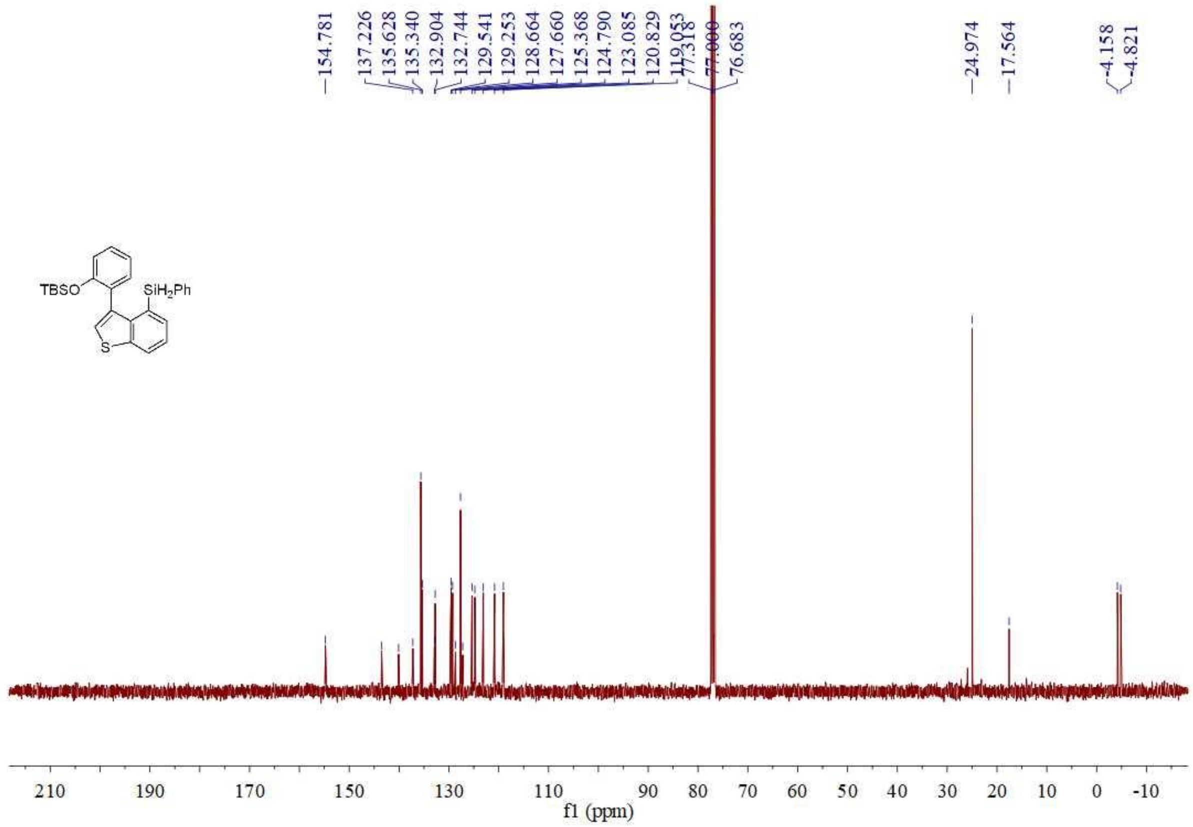
154.781
137.226
135.628
135.340
132.904
132.744
129.541
129.253
128.664
127.660
125.368
124.790
123.085
120.829
119.053
77.318
77.000
76.683
24.974
17.564
-4.158
-4.821
TBSO
SiH2Ph
S
210
190
170
150
130
110
90
80
70
60
50
40
30
20
10
0
-10
f1 (ppm)

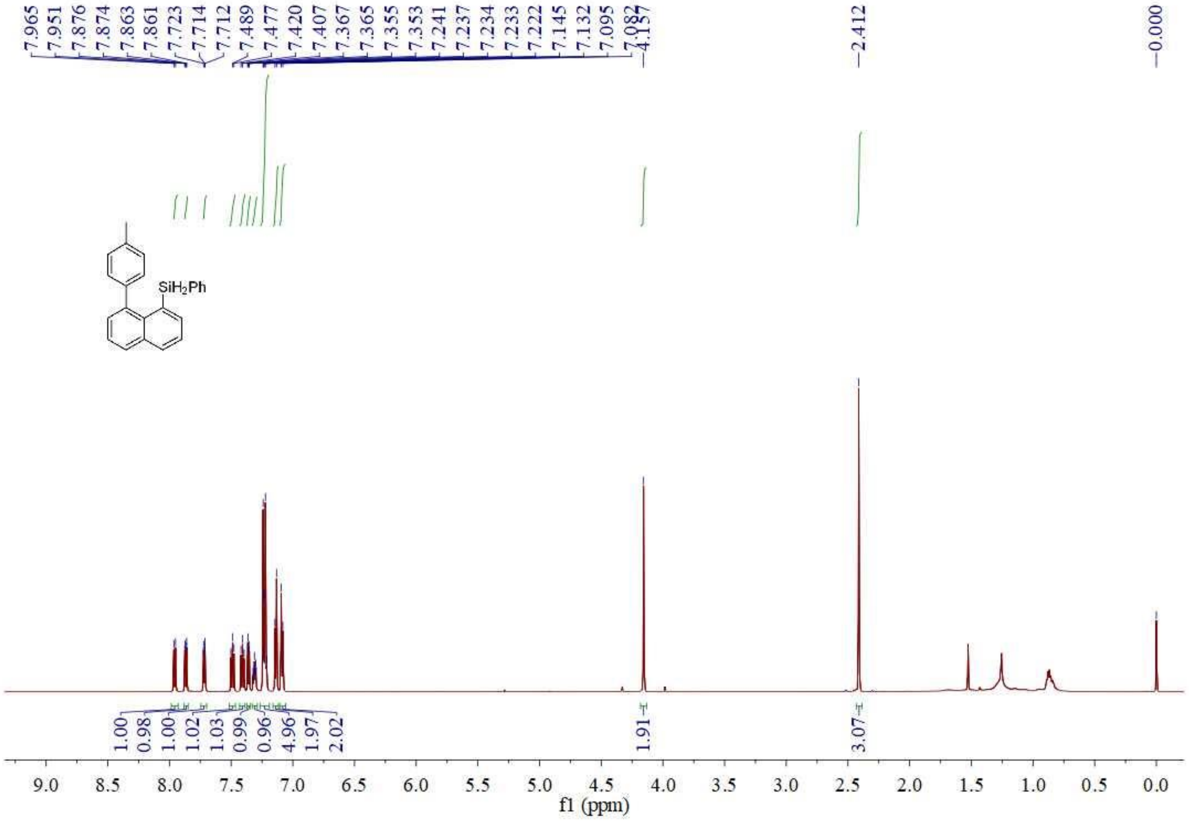

7.965
7.951
7.876
7.874
7.863
7.861
7.723
7.714
7.712
7.489
7.477
7.420
7.407
7.367
7.365
7.355
7.353
7.241
7.237
7.234
7.233
7.222
7.145
7.132
7.095
7.082
4.157
2.412
0.000
SiH2Ph
1.00
0.98
1.00
1.02
1.03
0.99
0.96
4.96
1.97
2.02
1.91
3.07
f1 (ppm)

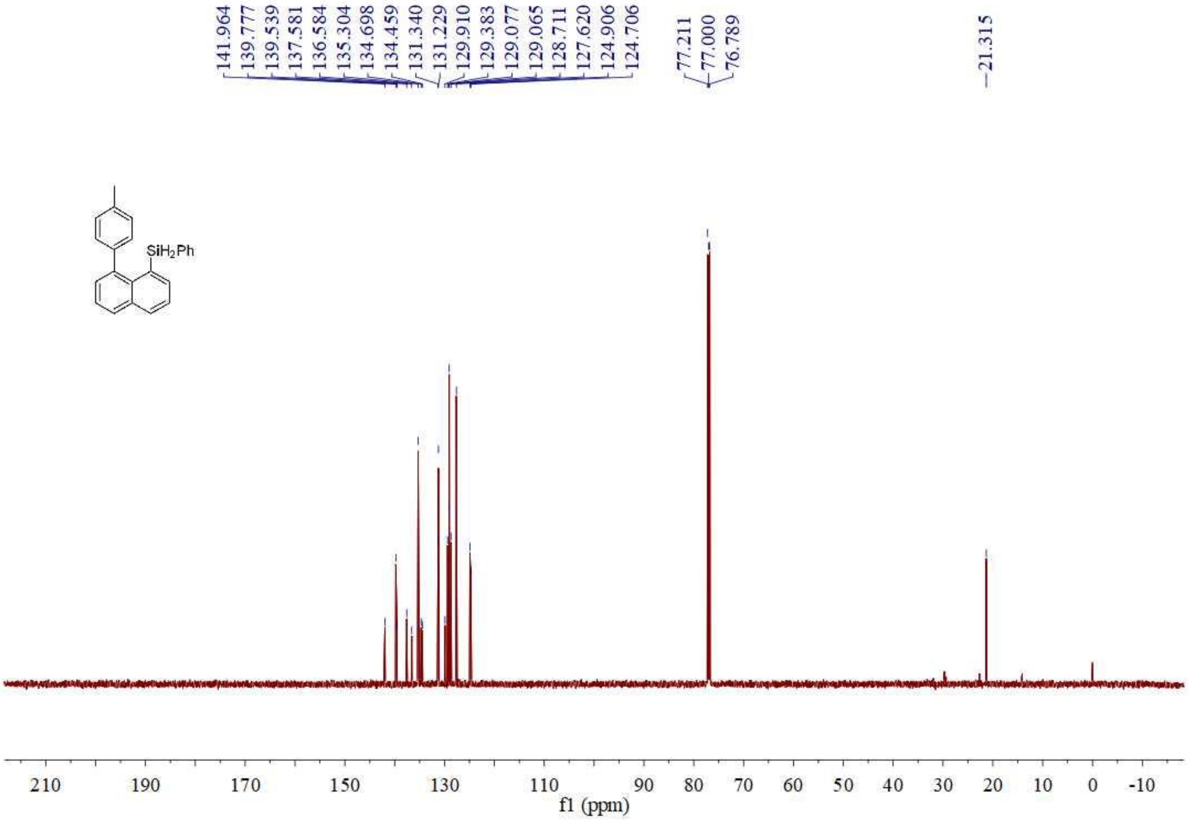

141.964
139.777
139.539
137.581
136.584
135.304
134.698
134.459
131.340
131.229
129.910
129.383
129.077
129.065
128.711
127.620
124.906
124.706
77.211
77.000
76.789
21.315
SiH2Ph
f1 (ppm)

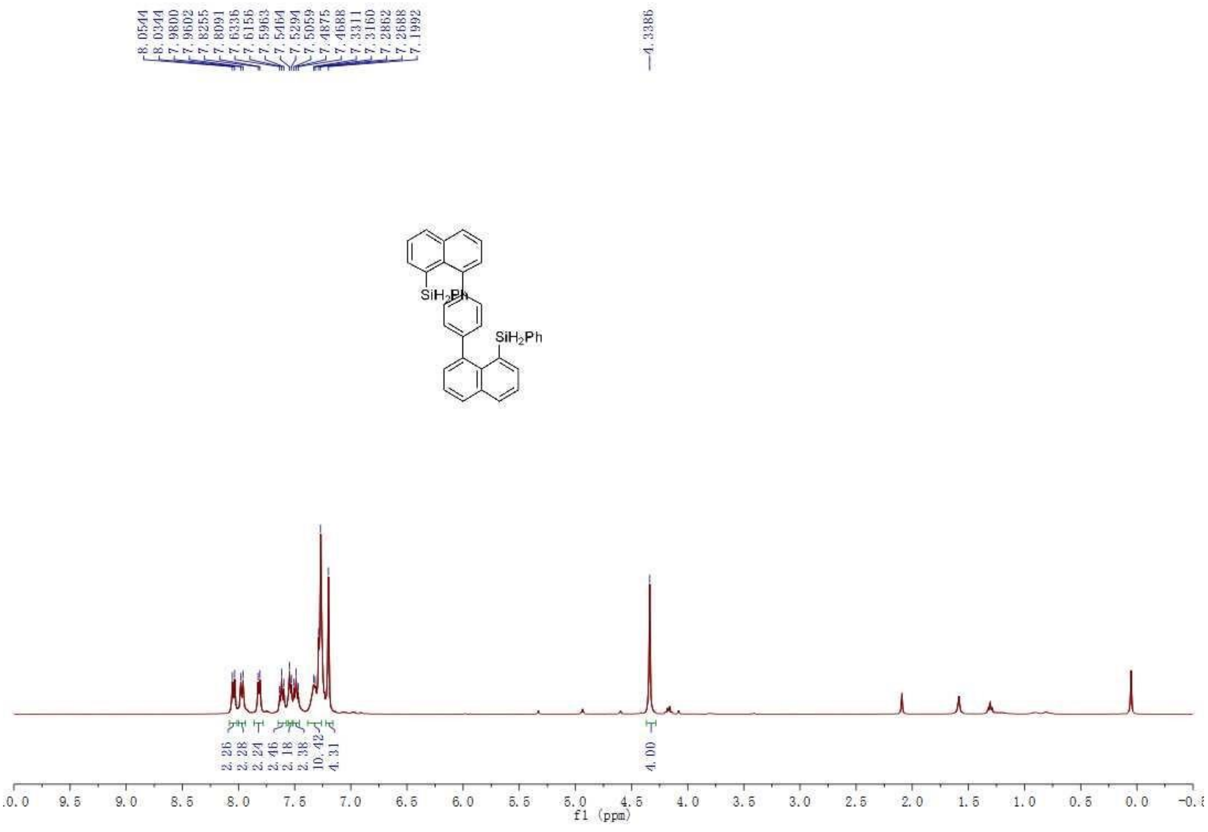
8.0544
8.0344
7.9800
7.9602
7.8255
7.8091
7.6336
7.6156
7.5963
7.5464
7.5294
7.5059
7.4875
7.4688
7.3311
7.3160
7.2862
7.2688
7.1992
4.3386
SiH2Ph
SiH2Ph
2.26
2.28
2.24
2.46
2.18
2.38
10.42
4.31
4.00
f1 (ppm)

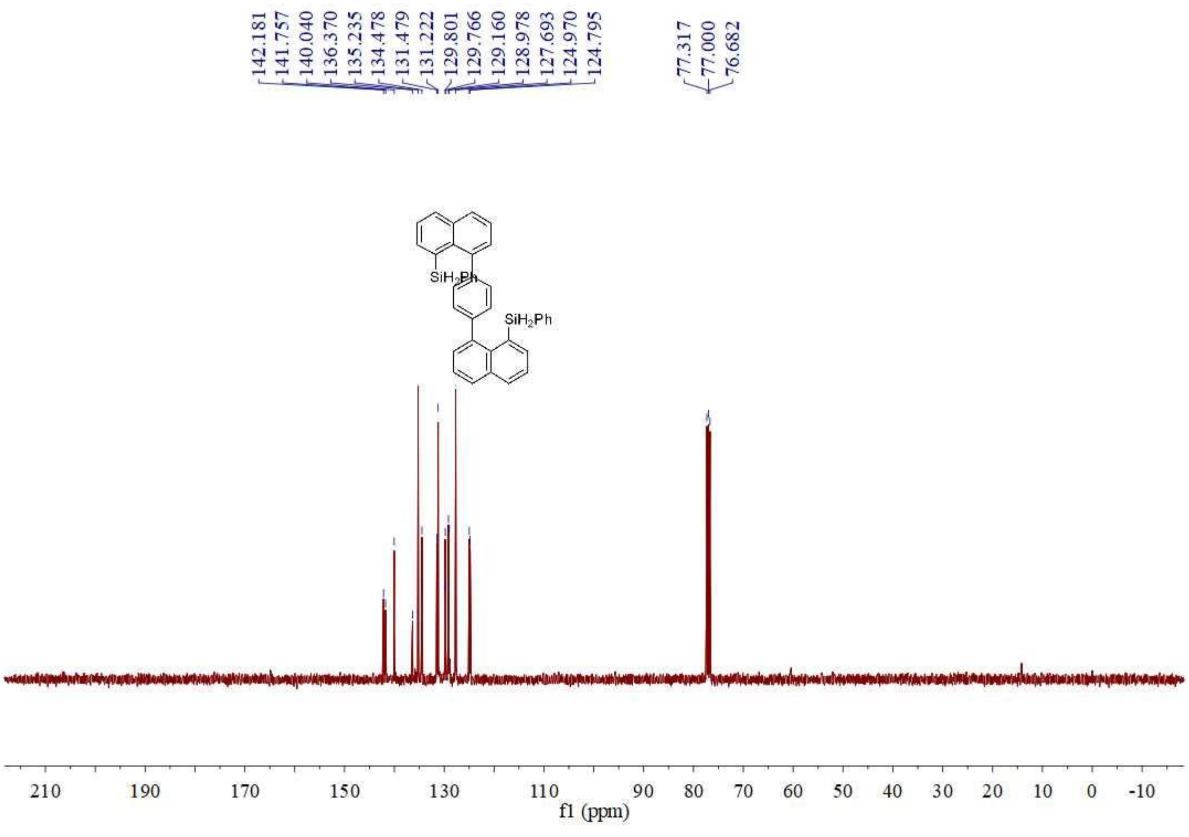
142.181
141.757
140.040
136.370
135.235
134.478
131.479
131.222
129.801
129.766
129.160
128.978
127.693
124.970
124.795
77.317
77.000
76.682
SiH2Ph
SiH2Ph
f1 (ppm)

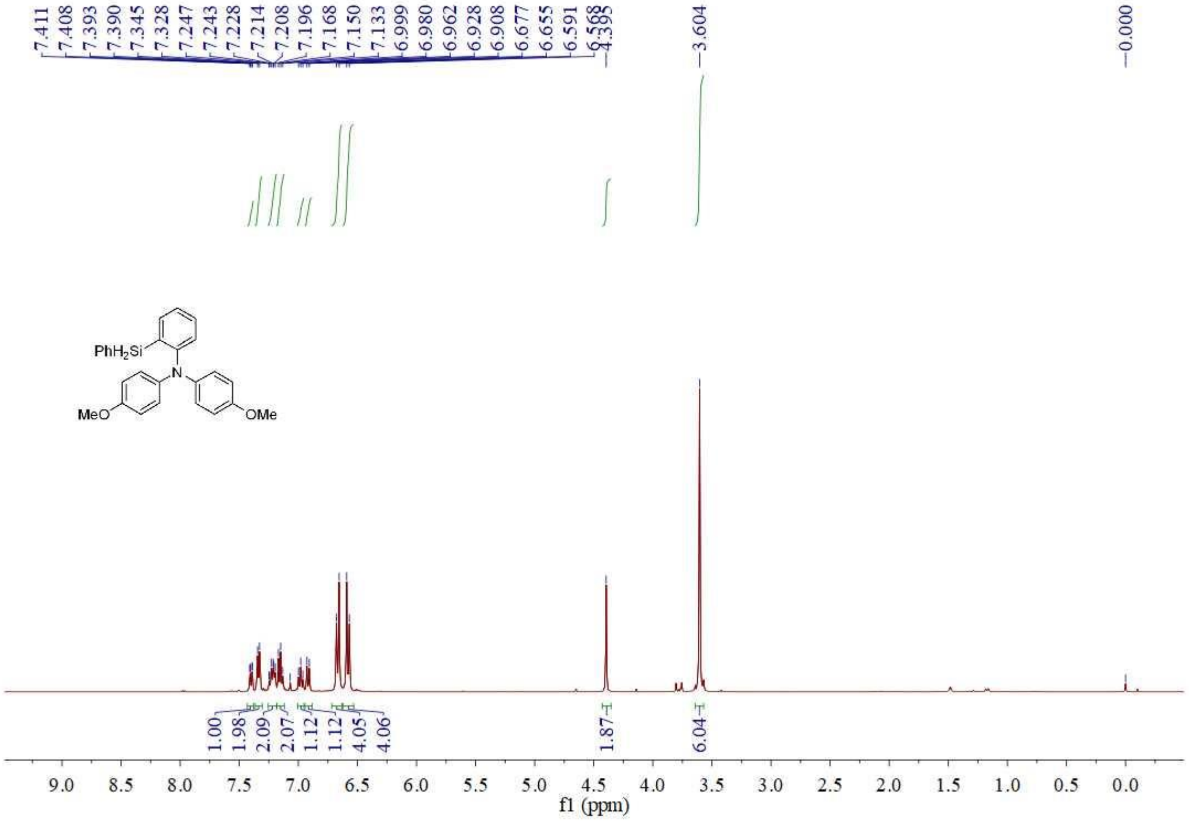

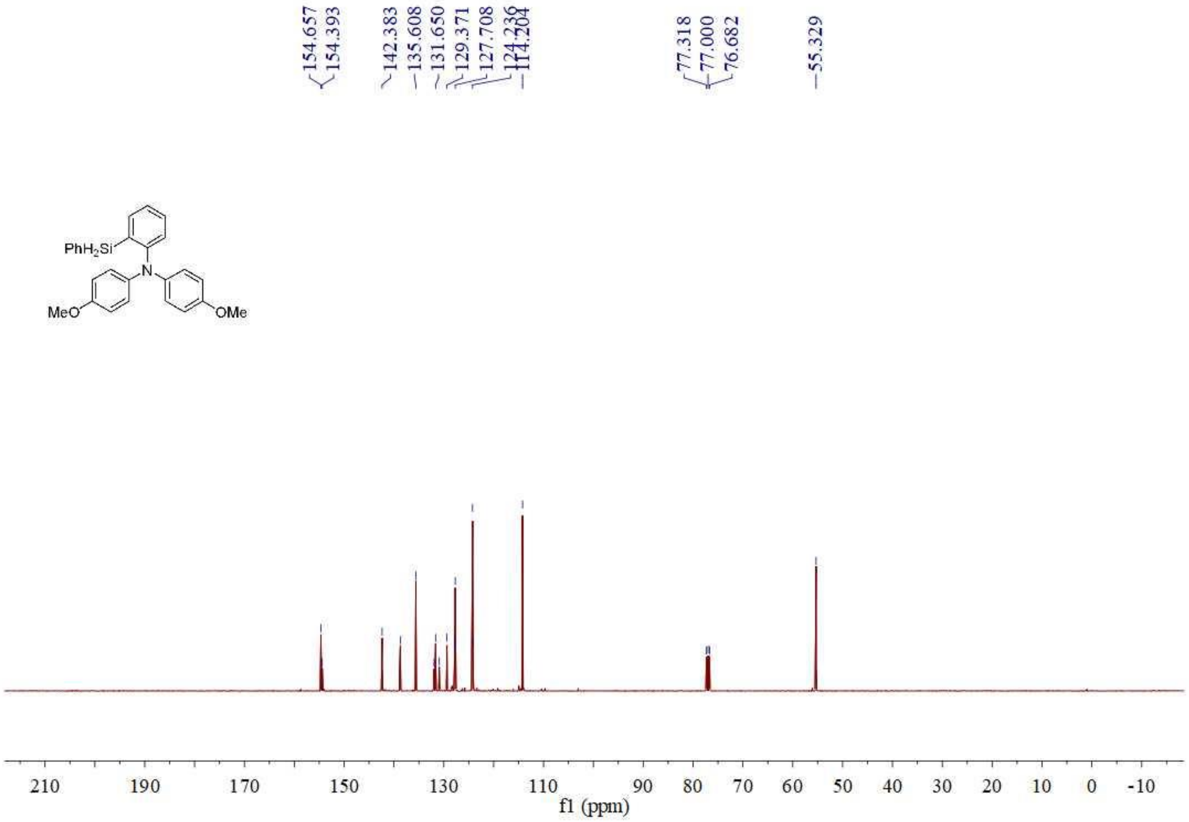

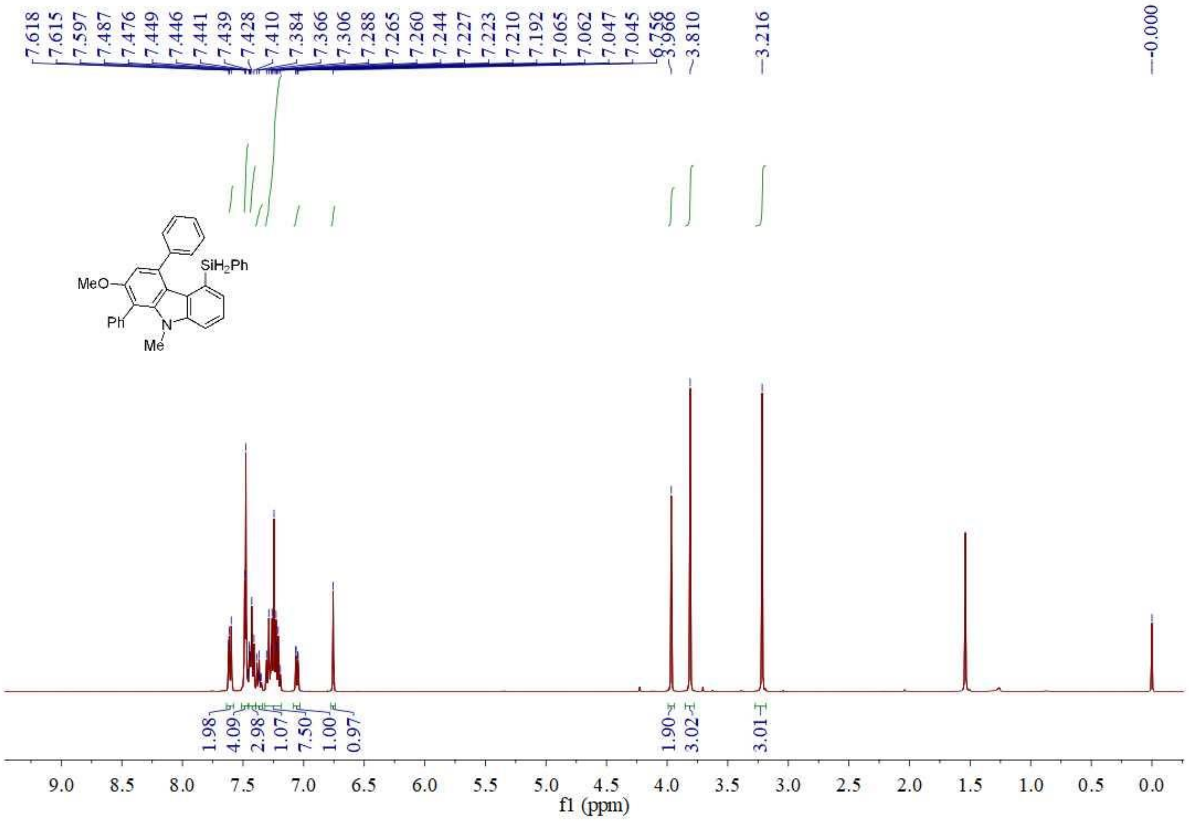
7.618
7.615
7.597
7.487
7.476
7.449
7.446
7.441
7.439
7.428
7.410
7.384
7.366
7.306
7.288
7.265
7.260
7.244
7.227
7.223
7.210
7.192
7.065
7.062
7.047
7.045
6.756
3.966
3.810
3.216
0.000
MeO
SiH2Ph
Ph
N
Me
1.98
4.09
2.98
1.07
7.50
1.00
0.97
1.90
3.02
3.01
9.0 8.5 8.0 7.5 7.0 6.5 6.0 5.5 5.0 4.5 4.0 3.5 3.0 2.5 2.0 1.5 1.0 0.5 0.0
f1 (ppm)

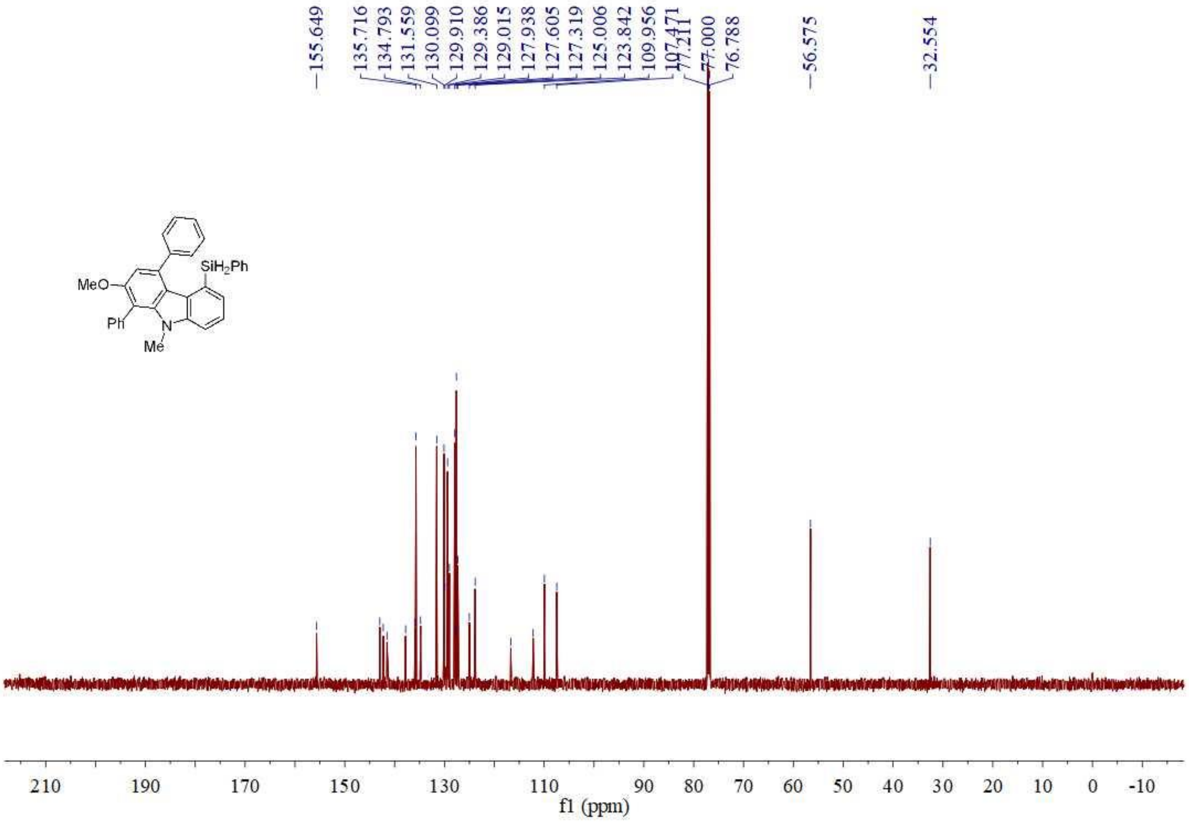
155.649
135.716
134.793
131.559
130.099
129.910
129.386
129.015
127.938
127.605
127.319
125.006
123.842
109.956
107.471
77.211
77.000
76.788
56.575
32.554
MeO
SiH2Ph
Ph
N
Me
210 190 170 150 130 110 90 80 70 60 50 40 30 20 10 0 -10
f1 (ppm)

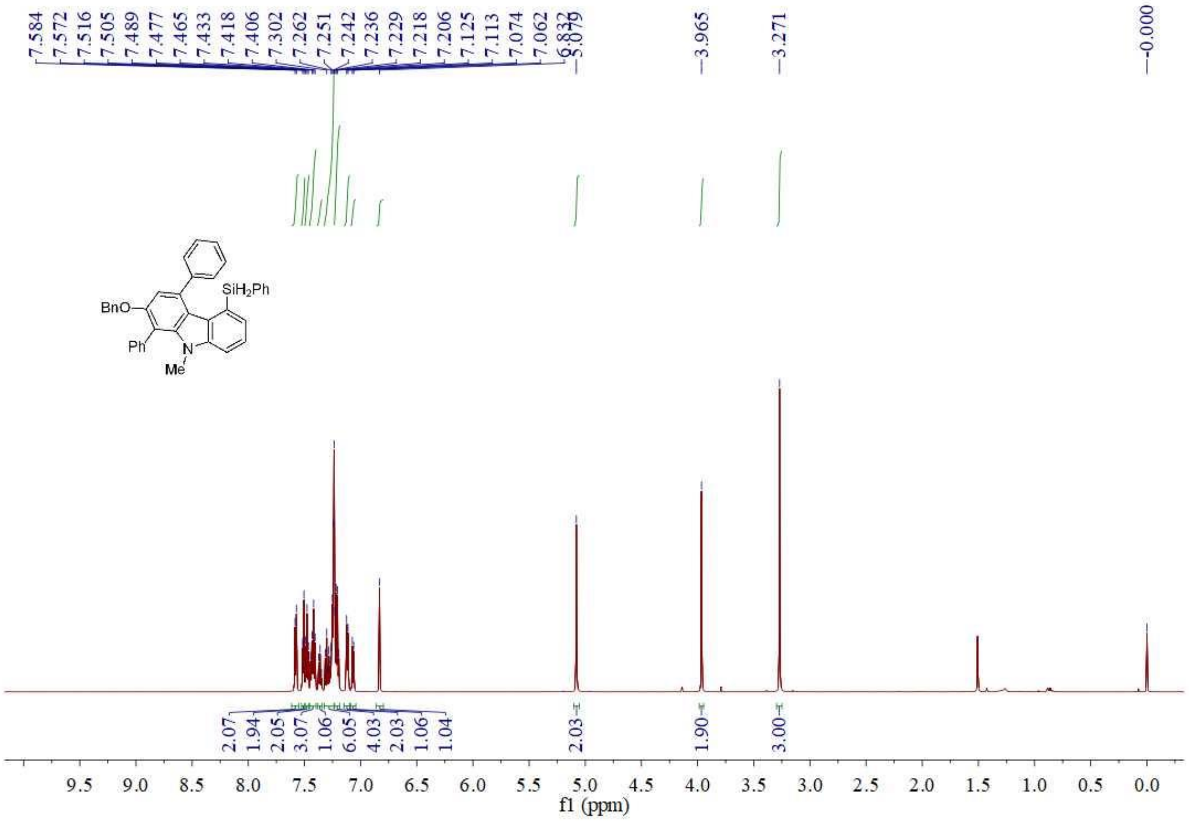
7.584
7.572
7.516
7.505
7.489
7.477
7.465
7.433
7.418
7.406
7.302
7.262
7.251
7.242
7.236
7.229
7.218
7.206
7.125
7.113
7.074
7.062
6.832
5.079
3.965
3.271
0.000
SiH2Ph
BnO
Ph
N
Me
2.07
1.94
2.05
3.07
1.06
6.05
4.03
2.03
1.06
1.04
2.03
1.90
3.00
9.5 9.0 8.5 8.0 7.5 7.0 6.5 6.0 5.5 5.0 4.5 4.0 3.5 3.0 2.5 2.0 1.5 1.0 0.5 0.0
f1 (ppm)

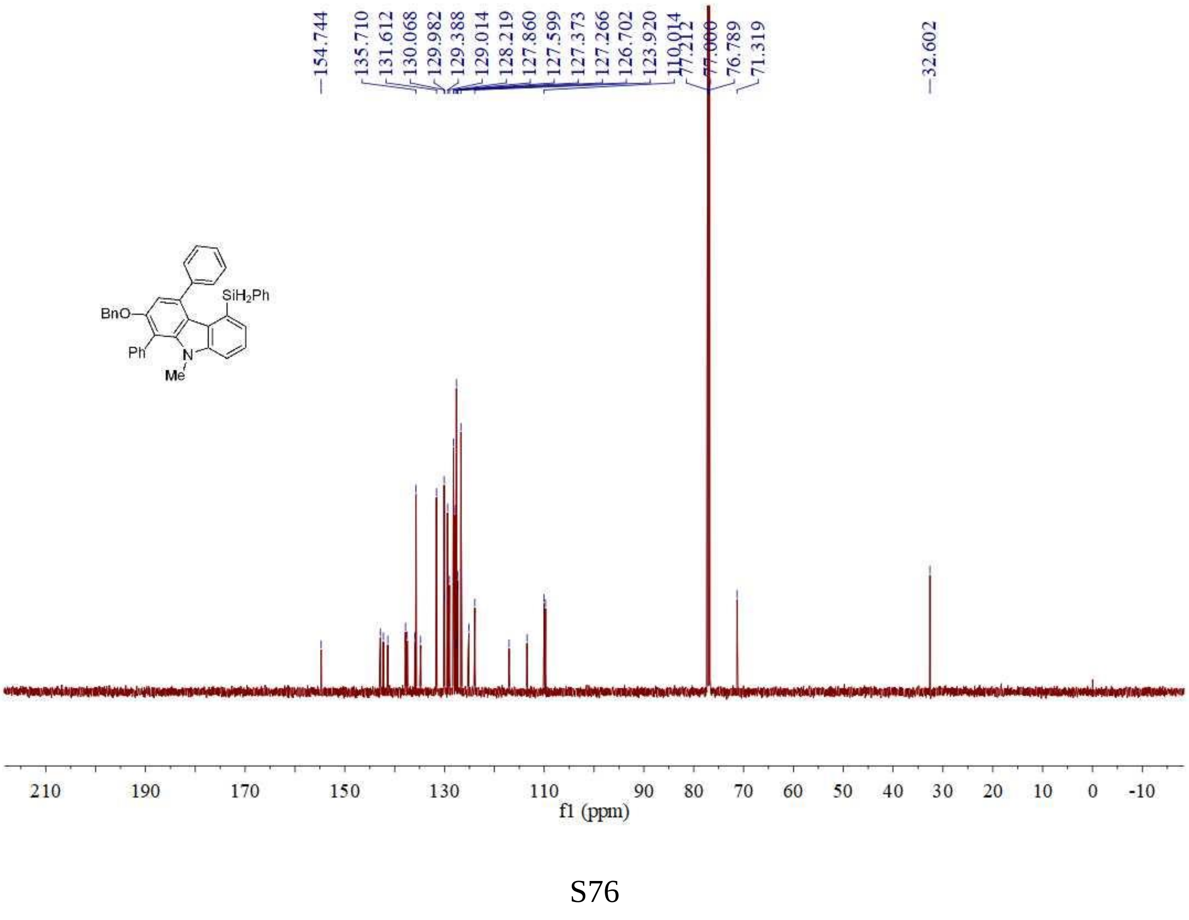
154.744
135.710
131.612
130.068
129.982
129.388
129.014
128.219
127.860
127.599
127.373
127.266
126.702
123.920
110.014
77.212
77.000
76.789
71.319
32.602
SiH2Ph
BnO
Ph
N
Me
210 190 170 150 130 110 90 80 70 60 50 40 30 20 10 0 -10
f1 (ppm)

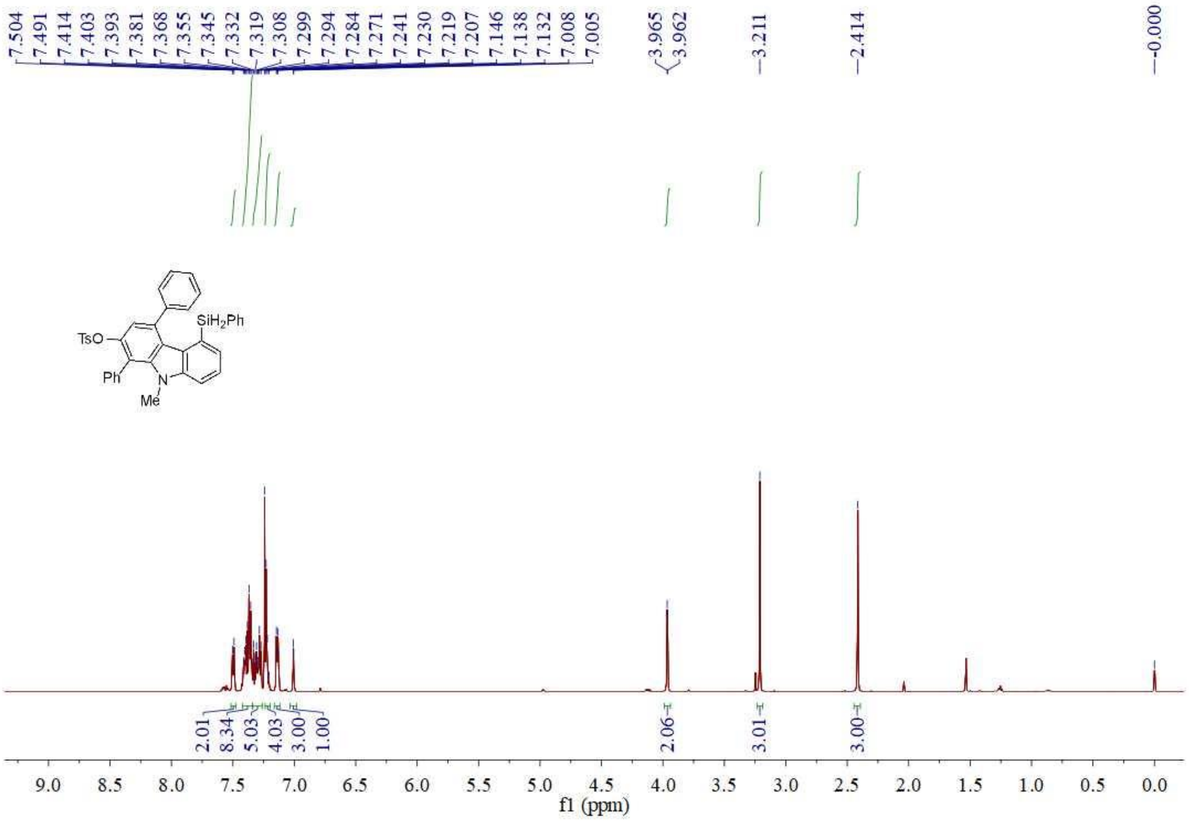

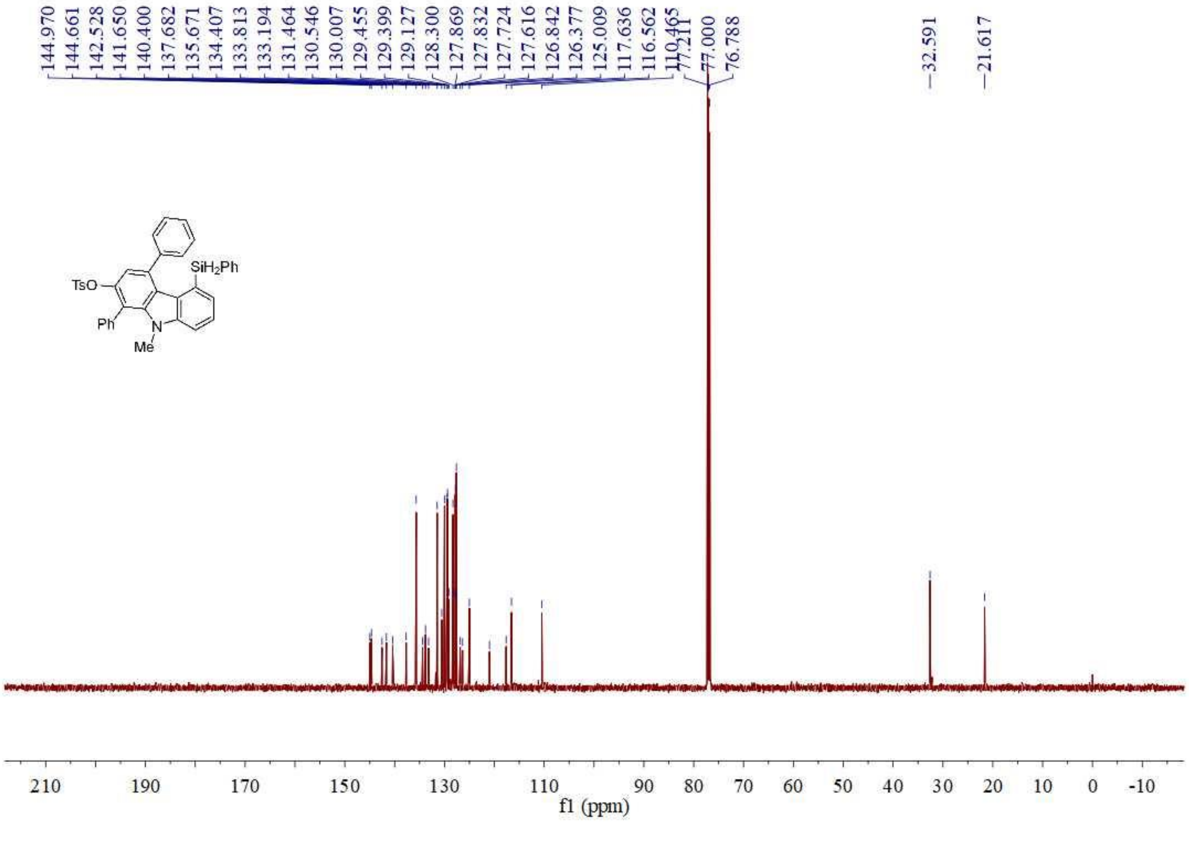

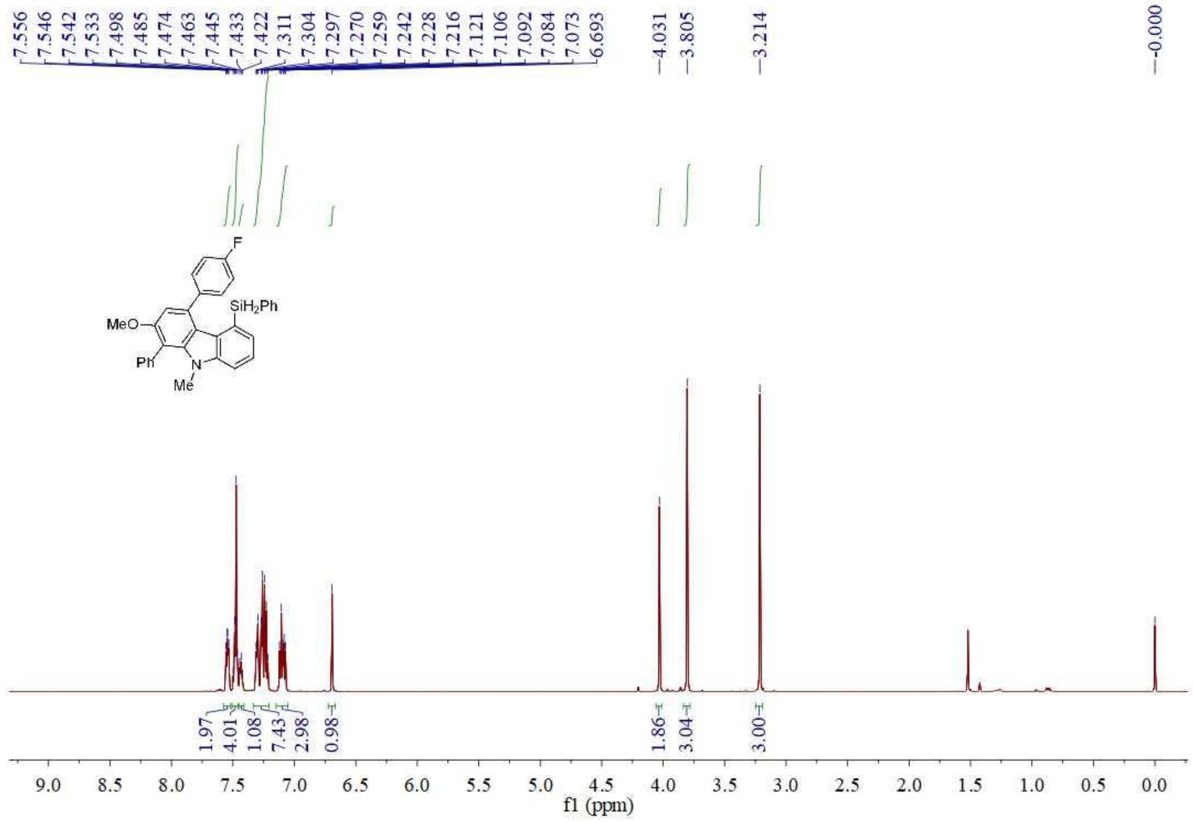
7.556
7.546
7.542
7.533
7.498
7.485
7.474
7.463
7.445
7.433
7.422
7.311
7.304
7.297
7.270
7.259
7.242
7.228
7.216
7.121
7.106
7.092
7.084
7.073
6.693
4.031
3.805
3.214
0.000
F
SiH2Ph
MeO
Ph
N
Me
1.97
4.01
1.08
7.43
2.98
0.98
1.86
3.04
3.00
9.0 8.5 8.0 7.5 7.0 6.5 6.0 5.5 5.0 4.5 4.0 3.5 3.0 2.5 2.0 1.5 1.0 0.5 0.0
f1 (ppm)

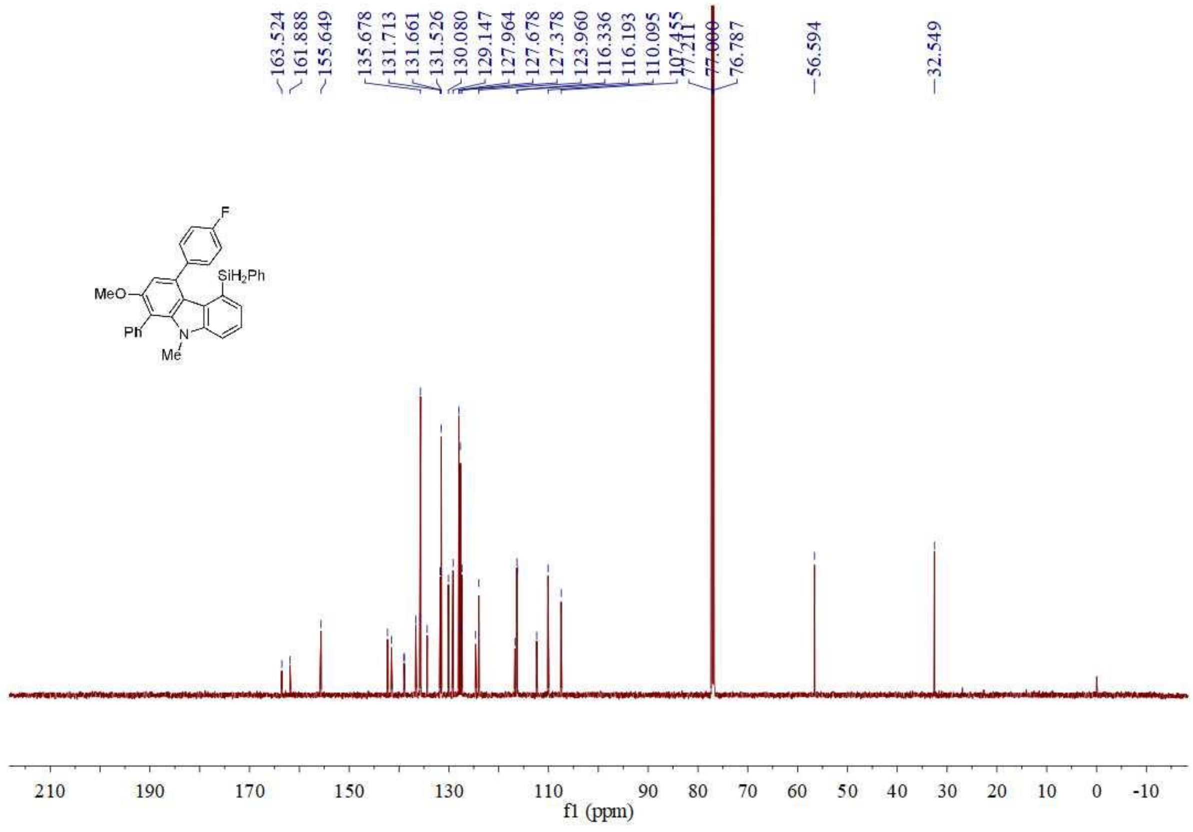
163.524
161.888
155.649
135.678
131.713
131.661
131.526
130.080
129.147
127.964
127.678
127.378
123.960
116.336
116.193
110.095
107.455
77.211
77.000
76.787
56.594
32.549
F
SiH2Ph
MeO
Ph
N
Me
210 190 170 150 130 110 90 80 70 60 50 40 30 20 10 0 -10
f1 (ppm)

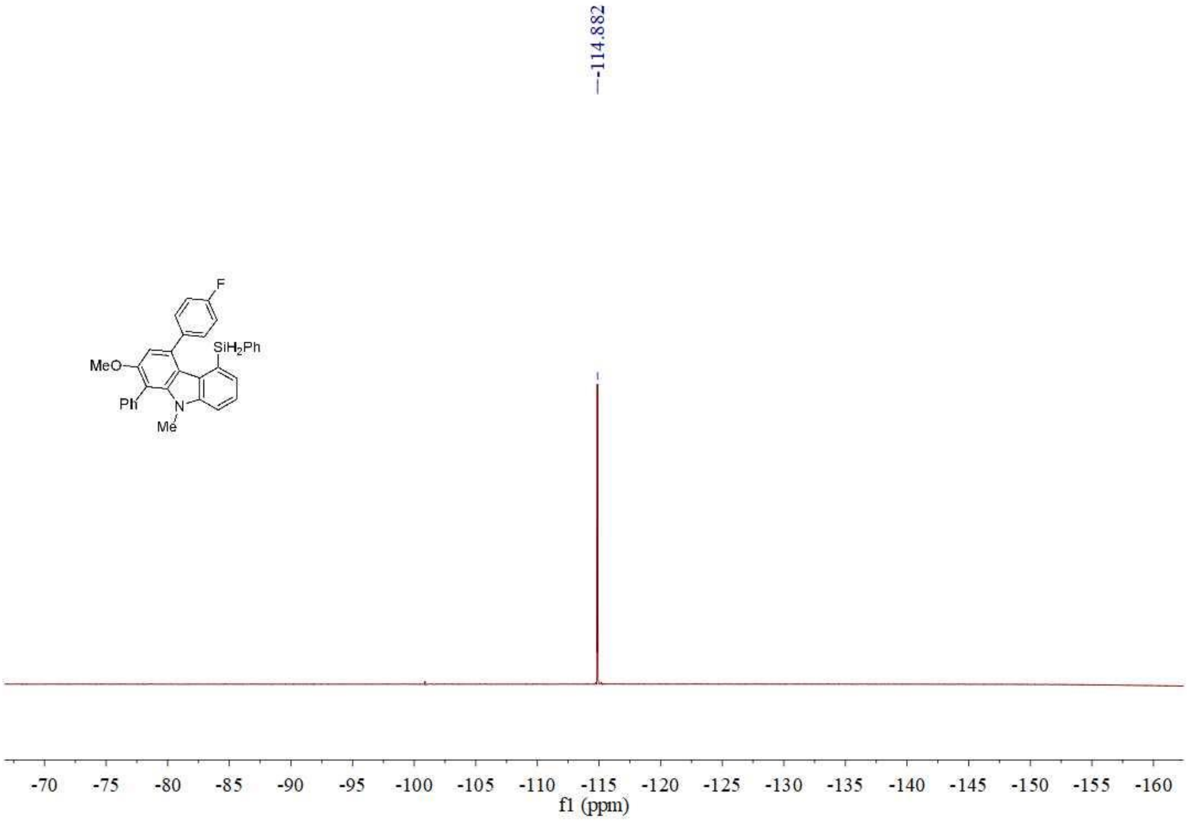
-114.882
F
SiH2Ph
MeO
Ph
N
Me
-70
-75
-80
-85
-90
-95
-100
-105
-110
-115
-120
-125
-130
-135
-140
-145
-150
-155
-160
f1 (ppm)

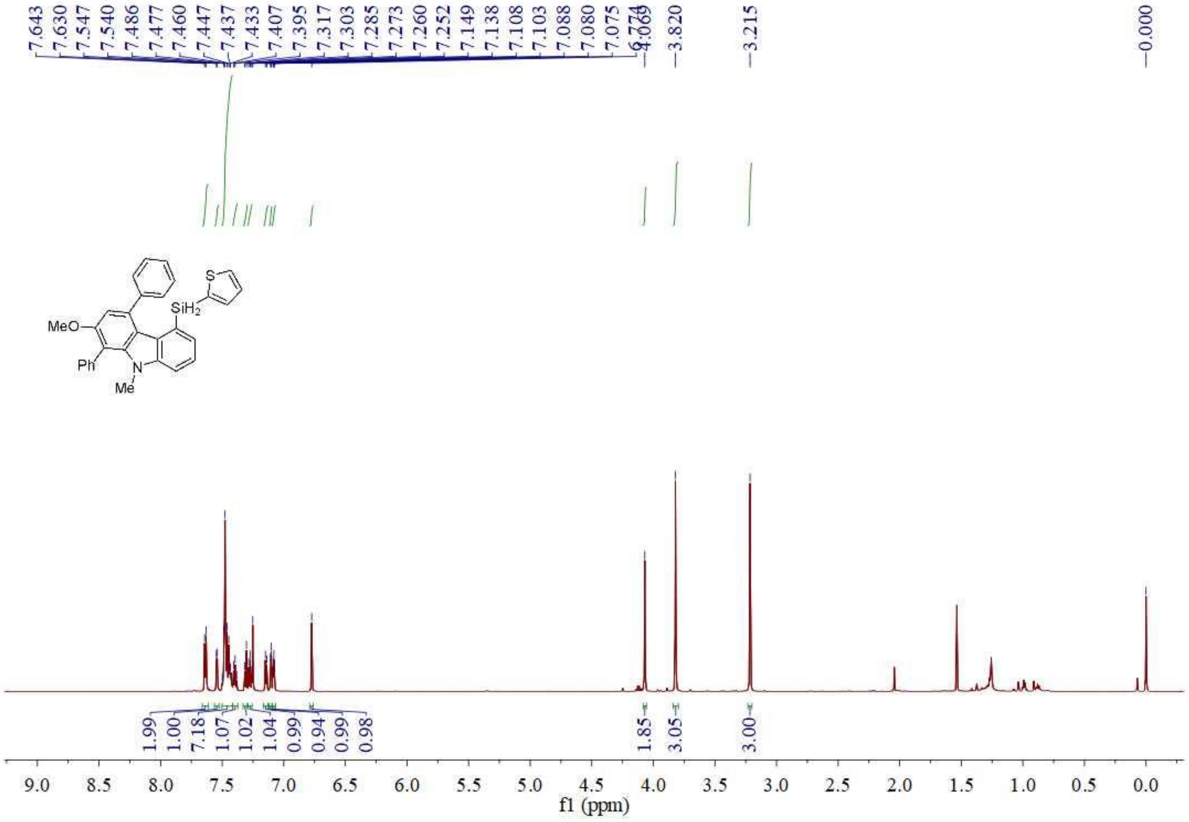
7.643
7.630
7.547
7.540
7.486
7.477
7.460
7.447
7.437
7.433
7.407
7.395
7.317
7.303
7.285
7.273
7.260
7.252
7.149
7.138
7.108
7.103
7.088
7.080
7.075
6.774
4.069
3.820
3.215
0.000
SiH2
S
MeO
Ph
N
Me
1.99
1.00
7.18
1.07
1.02
1.04
0.99
0.94
0.99
0.98
1.85
3.05
3.00
9.0 8.5 8.0 7.5 7.0 6.5 6.0 5.5 5.0 4.5 4.0 3.5 3.0 2.5 2.0 1.5 1.0 0.5 0.0
f1 (ppm)

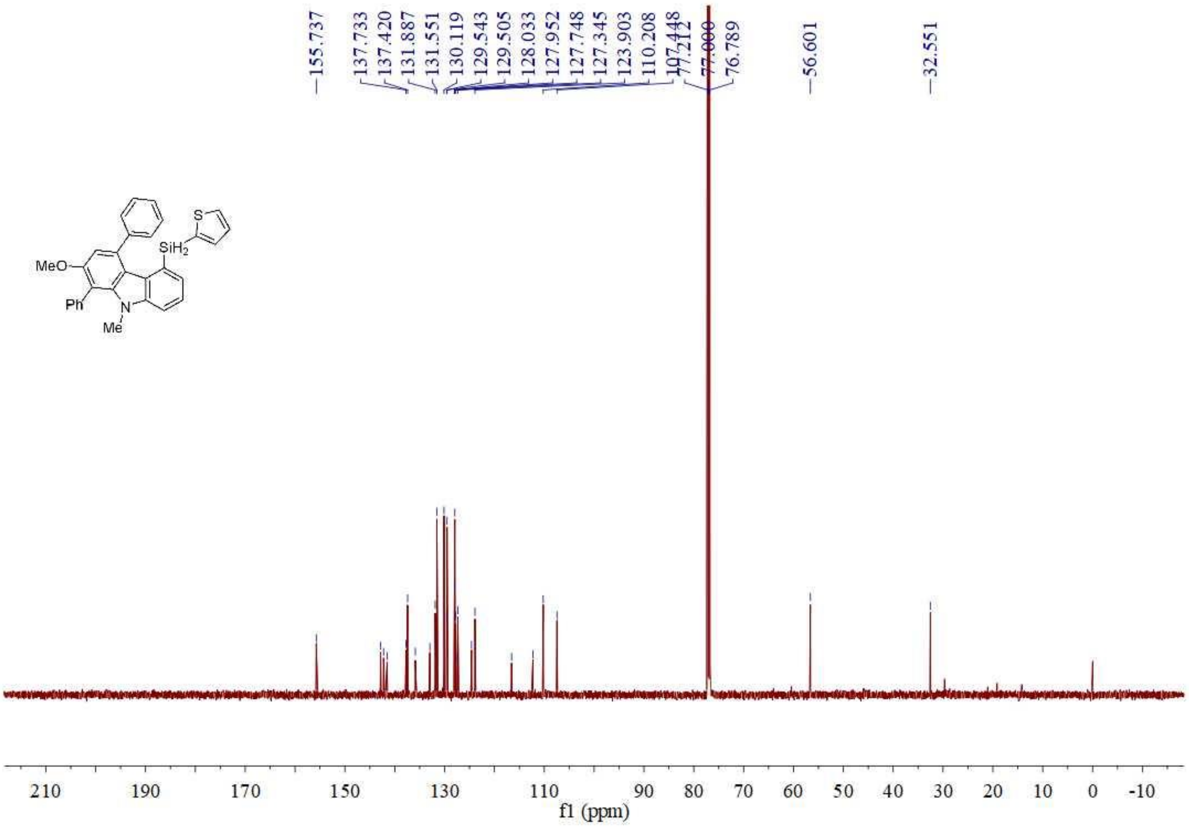
155.737
137.733
137.420
131.887
131.551
130.119
129.543
129.505
128.033
127.952
127.748
127.345
123.903
110.208
107.448
77.212
77.000
76.789
56.601
32.551
SiH2
S
MeO
Ph
N
Me
210 190 170 150 130 110 90 80 70 60 50 40 30 20 10 0 -10
f1 (ppm)

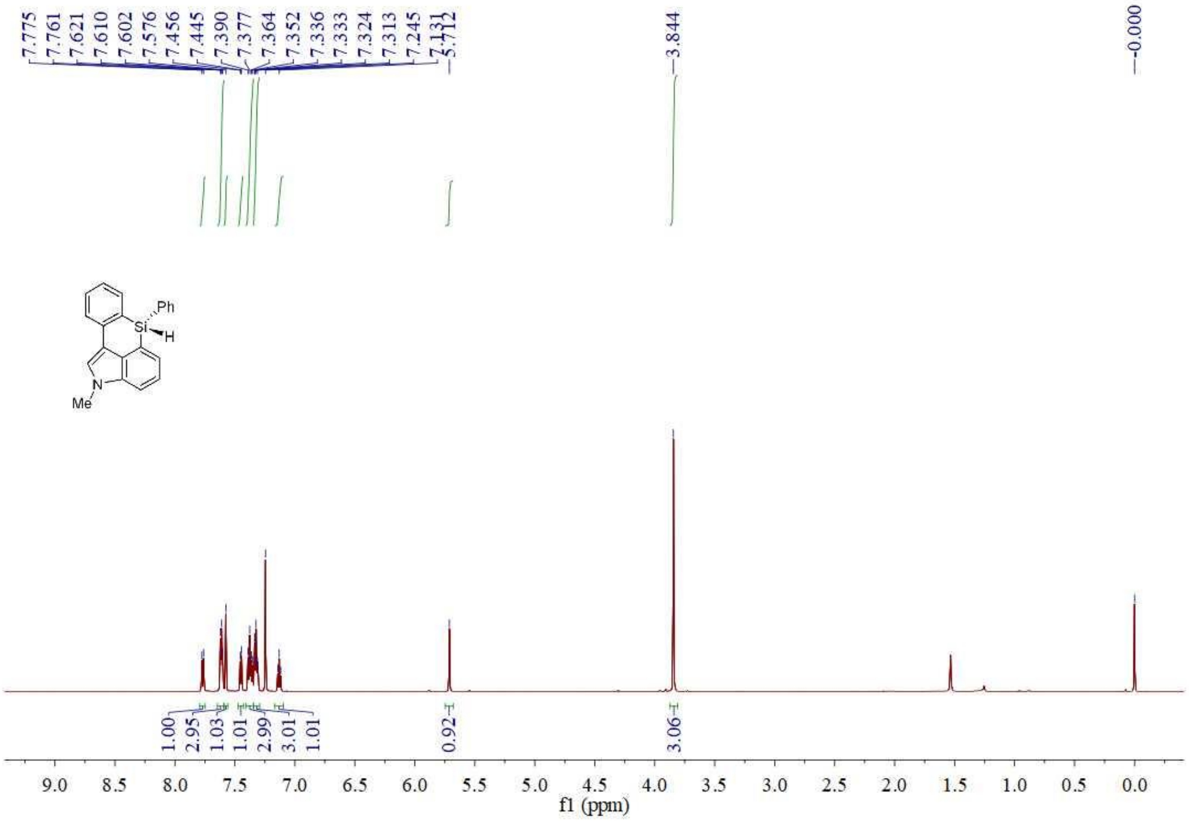
7.775
7.761
7.621
7.610
7.602
7.576
7.456
7.445
7.390
7.377
7.364
7.352
7.336
7.333
7.324
7.313
7.245
7.131
5.712
3.844
-0.000
Ph
Si
H
N
Me
1.00
2.95
1.03
1.01
2.99
3.01
1.01
0.92
3.06
9.0 8.5 8.0 7.5 7.0 6.5 6.0 5.5 5.0 4.5 4.0 3.5 3.0 2.5 2.0 1.5 1.0 0.5 0.0
f1 (ppm)

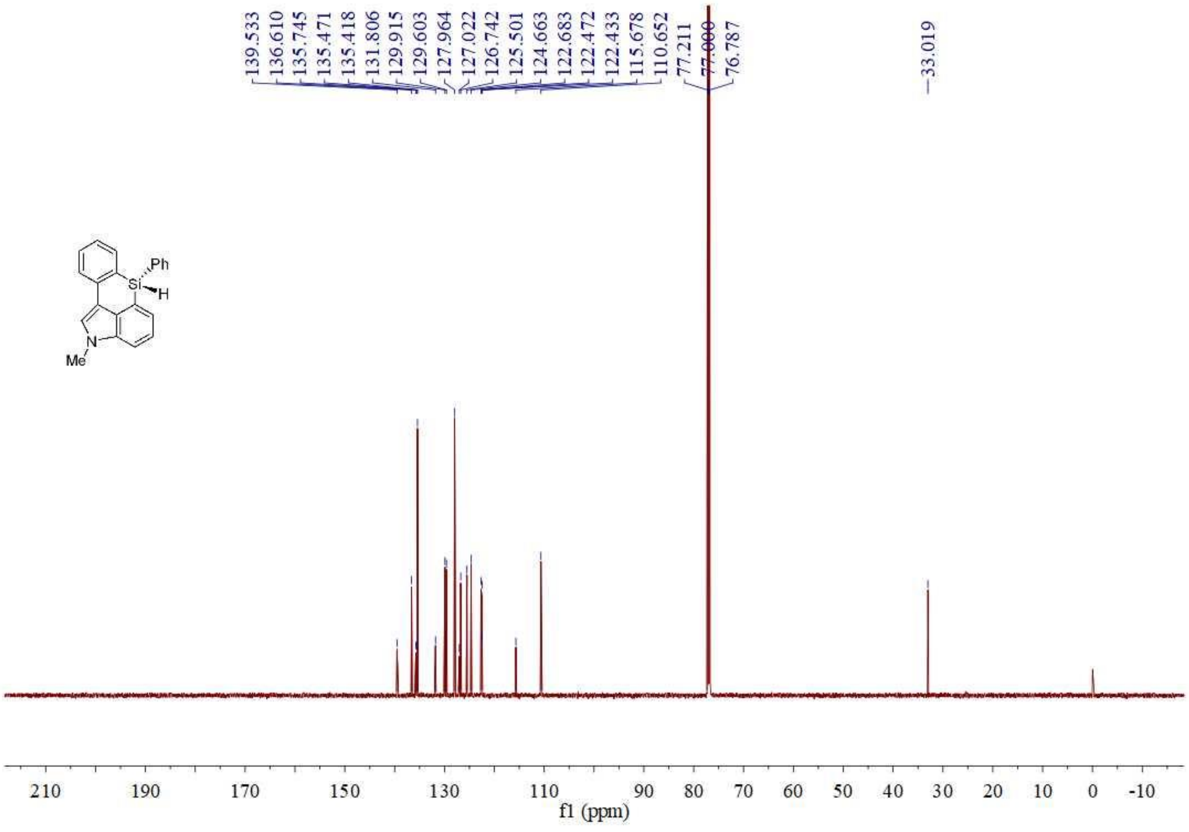
139.533
136.610
135.745
135.471
135.418
131.806
129.915
129.603
127.964
127.022
126.742
125.501
124.663
122.683
122.472
122.433
115.678
110.652
77.211
77.000
76.787
33.019
Ph
Si
H
N
Me
210 190 170 150 130 110 90 80 70 60 50 40 30 20 10 0 -10
f1 (ppm)

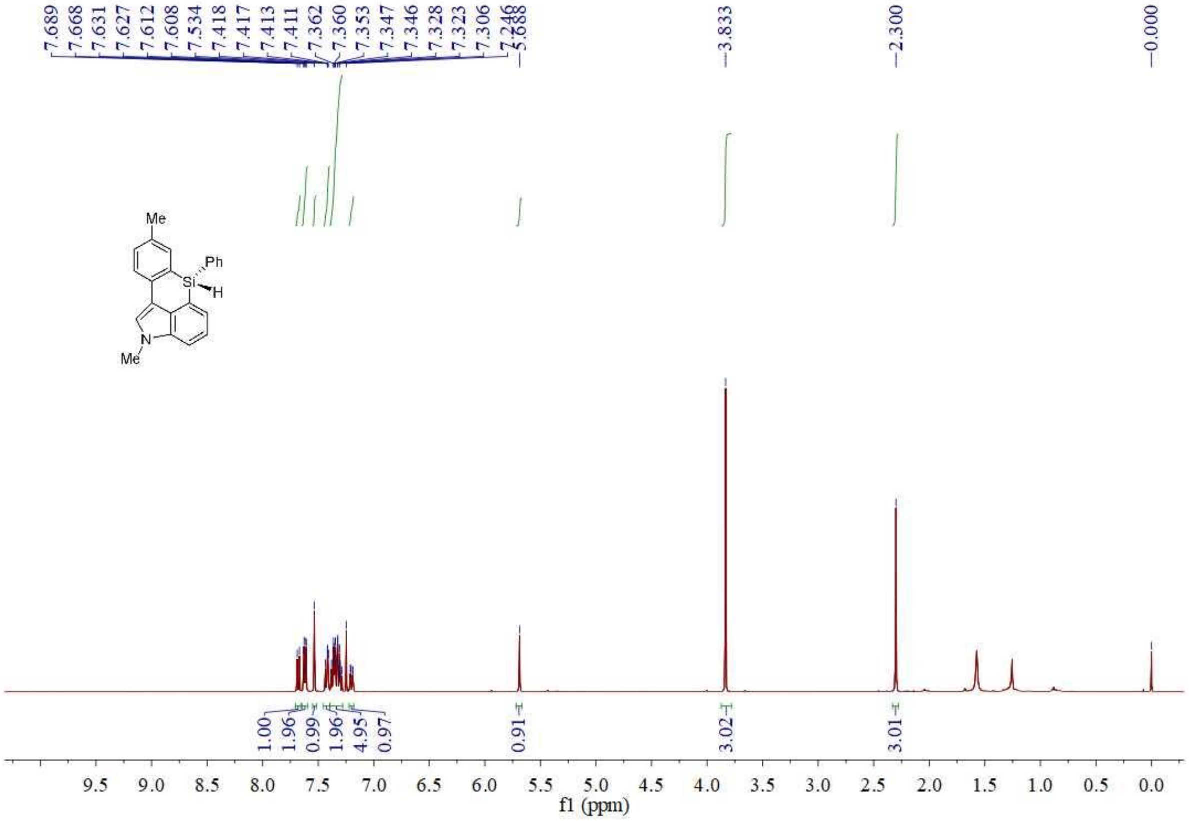
7.689
7.668
7.631
7.627
7.612
7.608
7.534
7.418
7.417
7.413
7.411
7.362
7.360
7.353
7.347
7.346
7.328
7.323
7.306
7.246
5.688
3.833
2.300
0.000
Me
Ph
Si
H
N
Me
1.00
1.96
0.99
1.96
4.95
0.97
0.91
3.02
3.01
9.5 9.0 8.5 8.0 7.5 7.0 6.5 6.0 5.5 5.0 4.5 4.0 3.5 3.0 2.5 2.0 1.5 1.0 0.5 0.0
f1 (ppm)

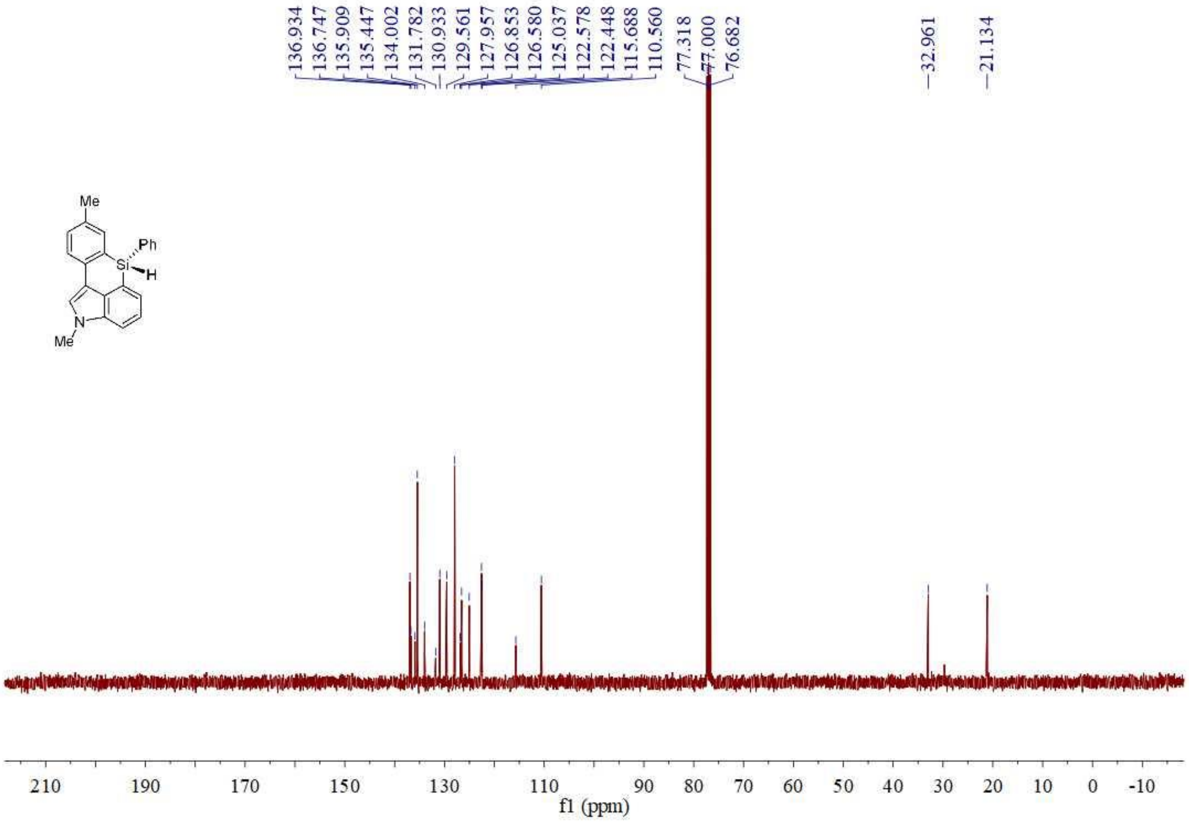
136.934
136.747
135.909
135.447
134.002
131.782
130.933
129.561
127.957
126.853
126.580
125.037
122.578
122.448
115.688
110.560
77.318
77.000
76.682
32.961
21.134
Me
Ph
Si
H
N
Me
210 190 170 150 130 110 90 80 70 60 50 40 30 20 10 0 -10
f1 (ppm)

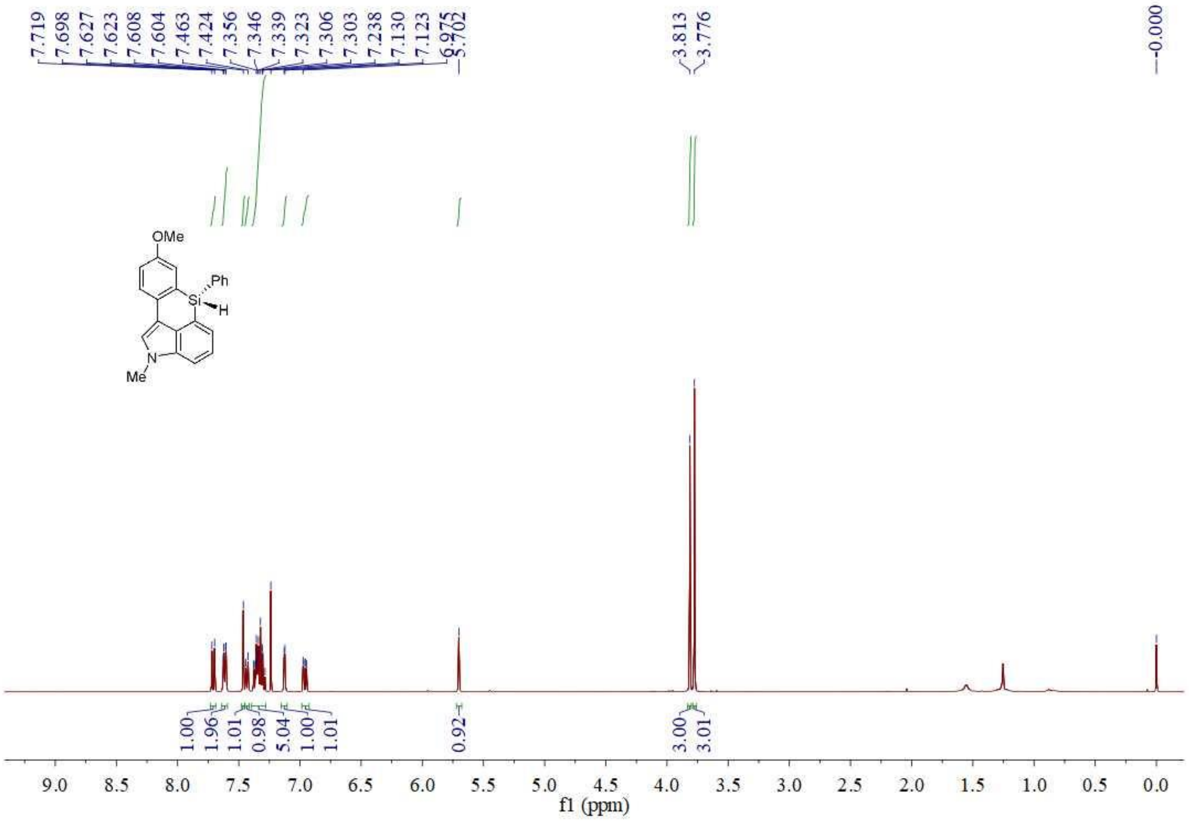
7.719
7.698
7.627
7.623
7.608
7.604
7.463
7.424
7.356
7.346
7.339
7.323
7.306
7.303
7.238
7.130
7.123
6.975
5.702
3.813
3.776
0.000
OMe
Si
Ph
H
N
Me
1.00
1.96
1.01
0.98
5.04
1.00
1.01
0.92
3.00
3.01
9.0 8.5 8.0 7.5 7.0 6.5 6.0 5.5 5.0 4.5 4.0 3.5 3.0 2.5 2.0 1.5 1.0 0.5 0.0
f1 (ppm)

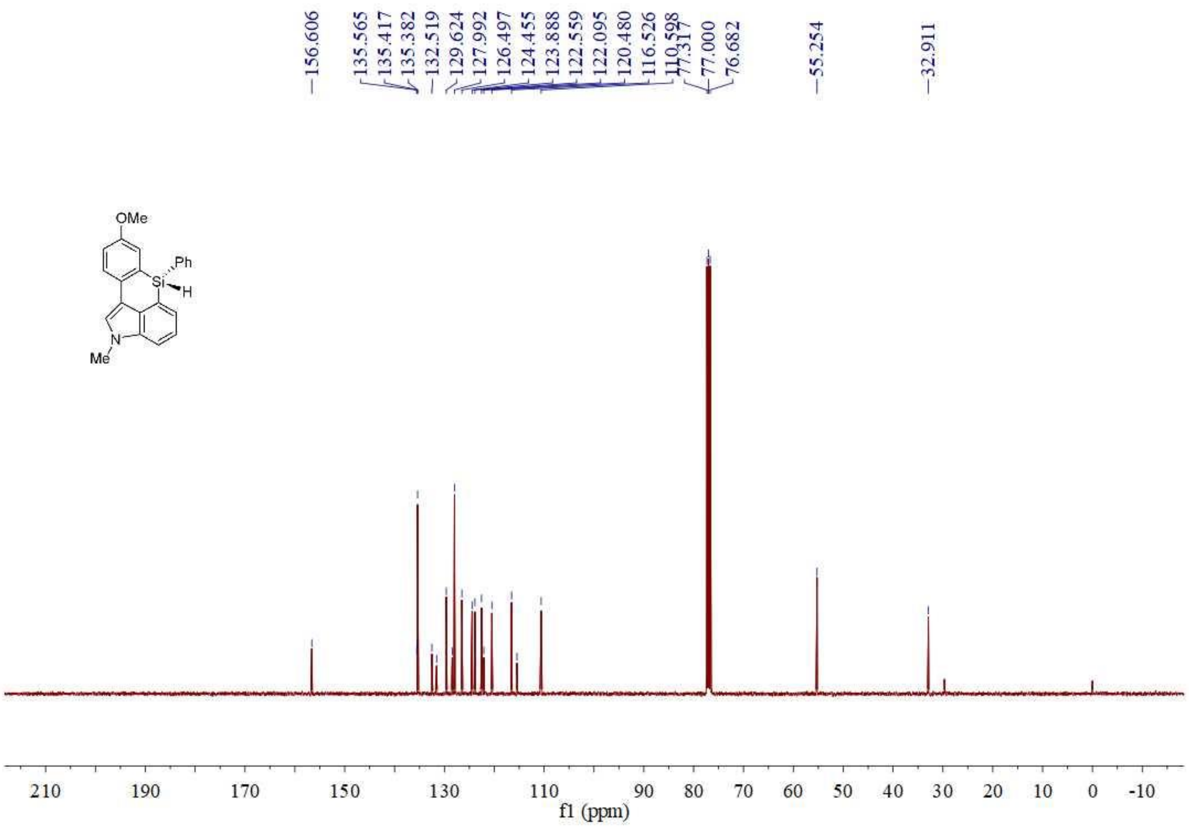
156.606
135.565
135.417
135.382
132.519
129.624
127.992
126.497
124.455
123.888
122.559
122.095
120.480
116.526
110.598
77.317
77.000
76.682
55.254
32.911
OMe
Si
Ph
H
N
Me
210 190 170 150 130 110 90 80 70 60 50 40 30 20 10 0 -10
f1 (ppm)

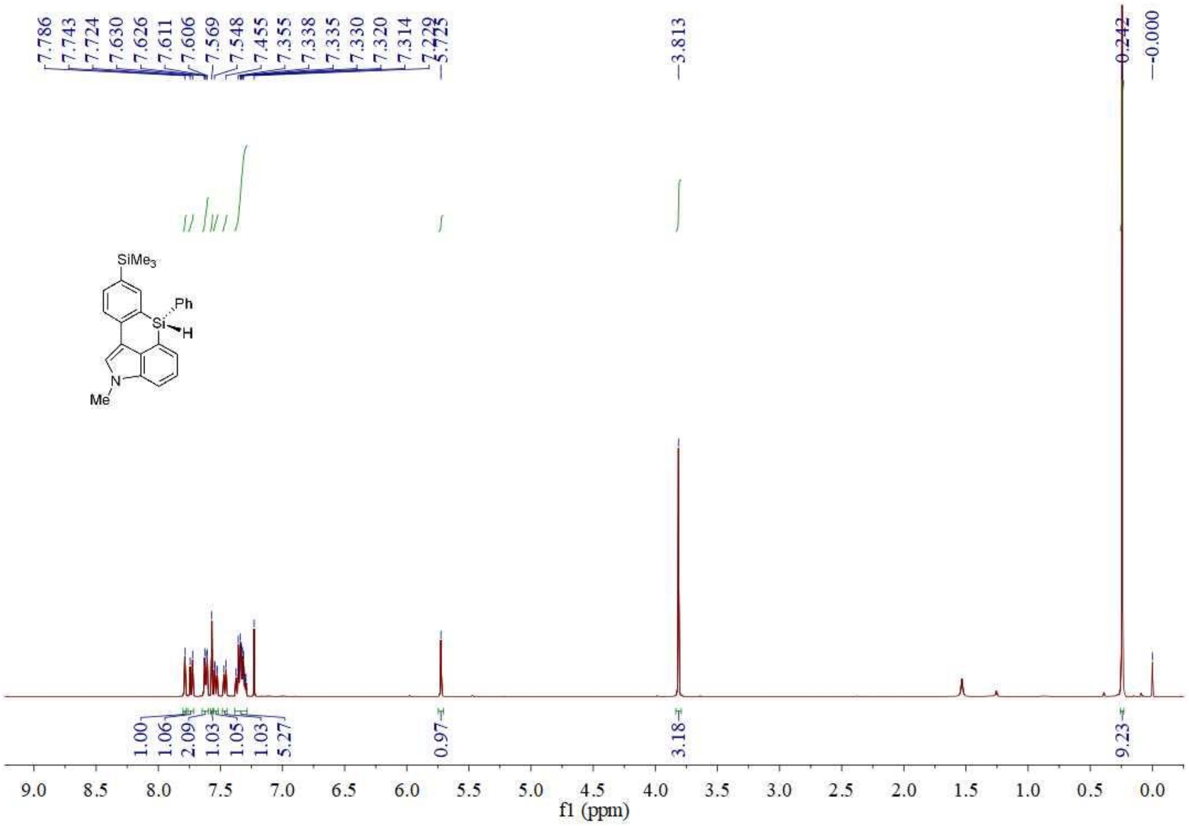
7.786
7.743
7.724
7.630
7.626
7.611
7.606
7.569
7.548
7.455
7.355
7.338
7.335
7.330
7.320
7.314
7.229
5.725
3.813
0.242
-0.000
SiMe3
Ph
Si
H
N
Me
1.00
1.06
2.09
1.03
1.05
1.03
5.27
0.97
3.18
9.23
f1 (ppm)

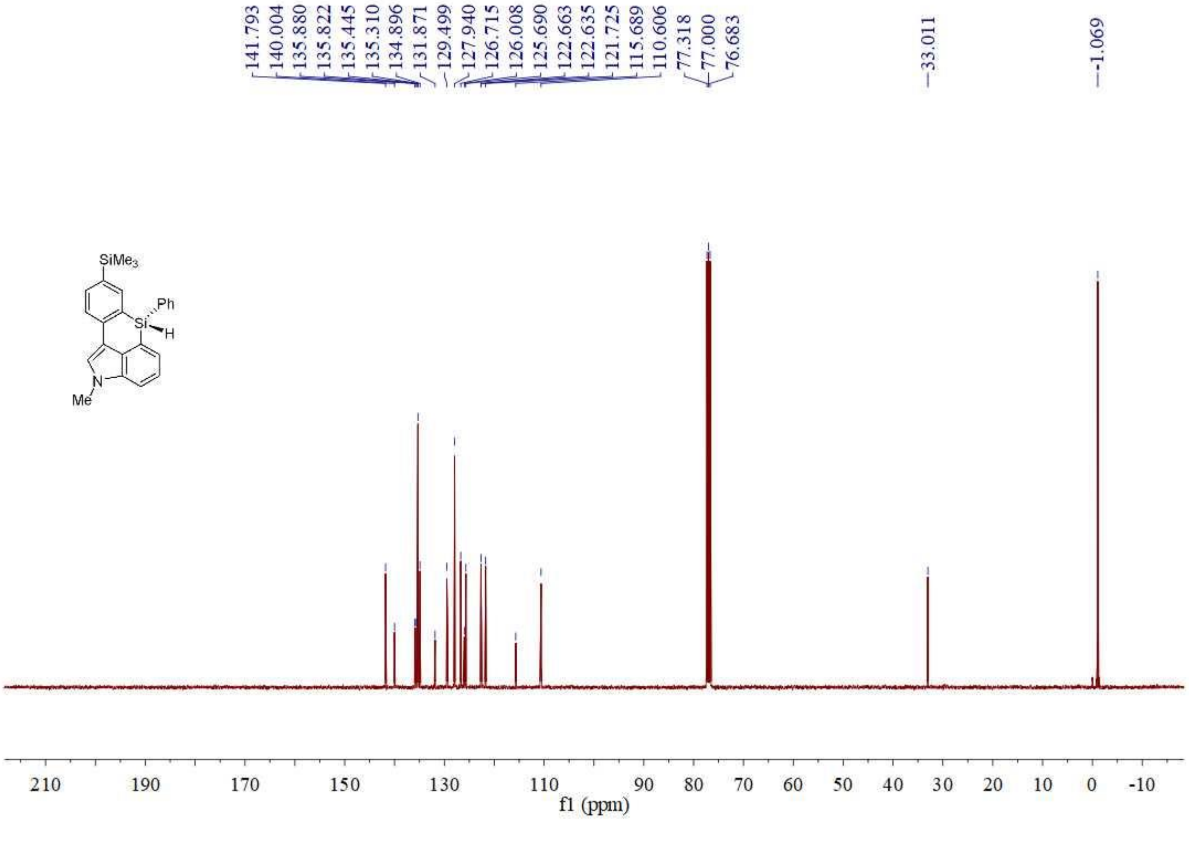
141.793
140.004
135.880
135.822
135.445
135.310
134.896
131.871
129.499
127.940
126.715
126.008
125.690
122.663
122.635
121.725
115.689
110.606
77.318
77.000
76.683
33.011
-1.069
SiMe3
Ph
Si
H
N
Me
f1 (ppm)

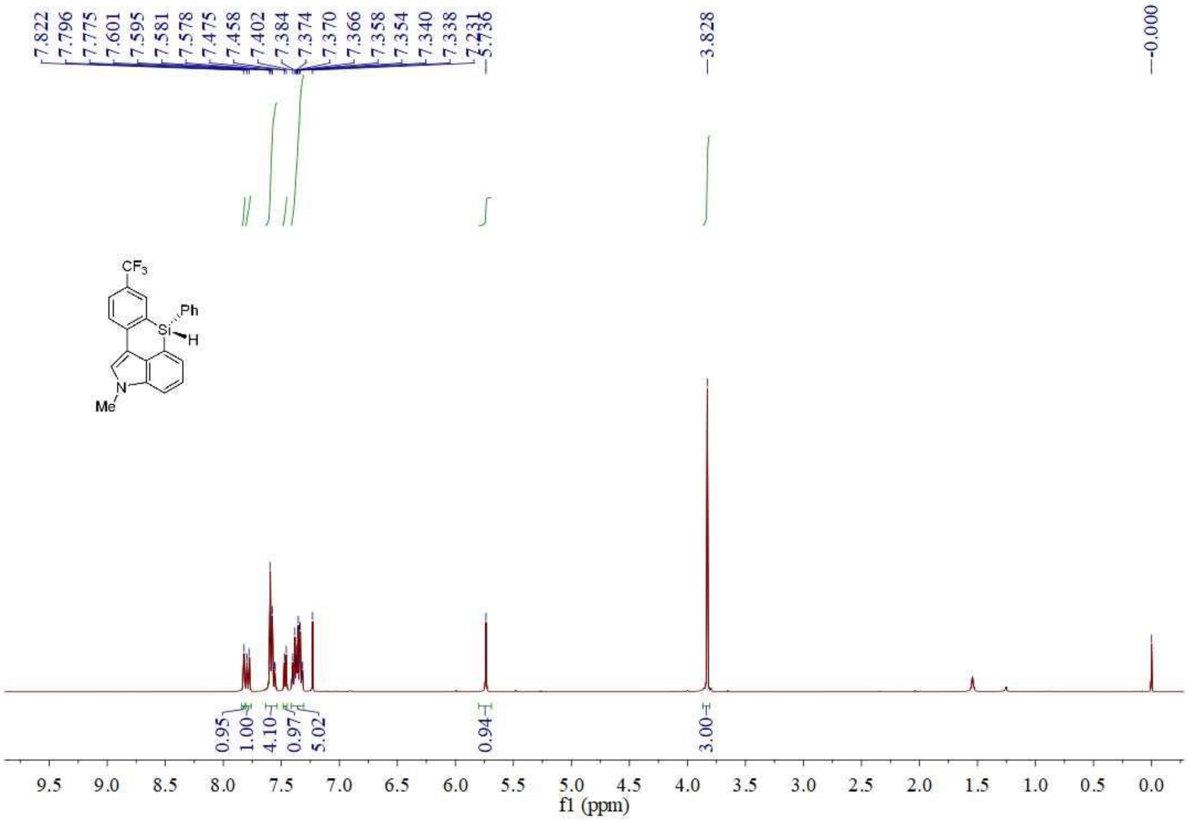
7.822
7.796
7.775
7.601
7.595
7.581
7.578
7.475
7.458
7.402
7.384
7.374
7.370
7.366
7.358
7.354
7.340
7.338
7.231
5.736
3.828
0.000
CF3
Ph
Si
H
N
Me
0.95
1.00
4.10
0.97
5.02
0.94
3.00
9.5 9.0 8.5 8.0 7.5 7.0 6.5 6.0 5.5 5.0 4.5 4.0 3.5 3.0 2.5 2.0 1.5 1.0 0.5 0.0
f1 (ppm)

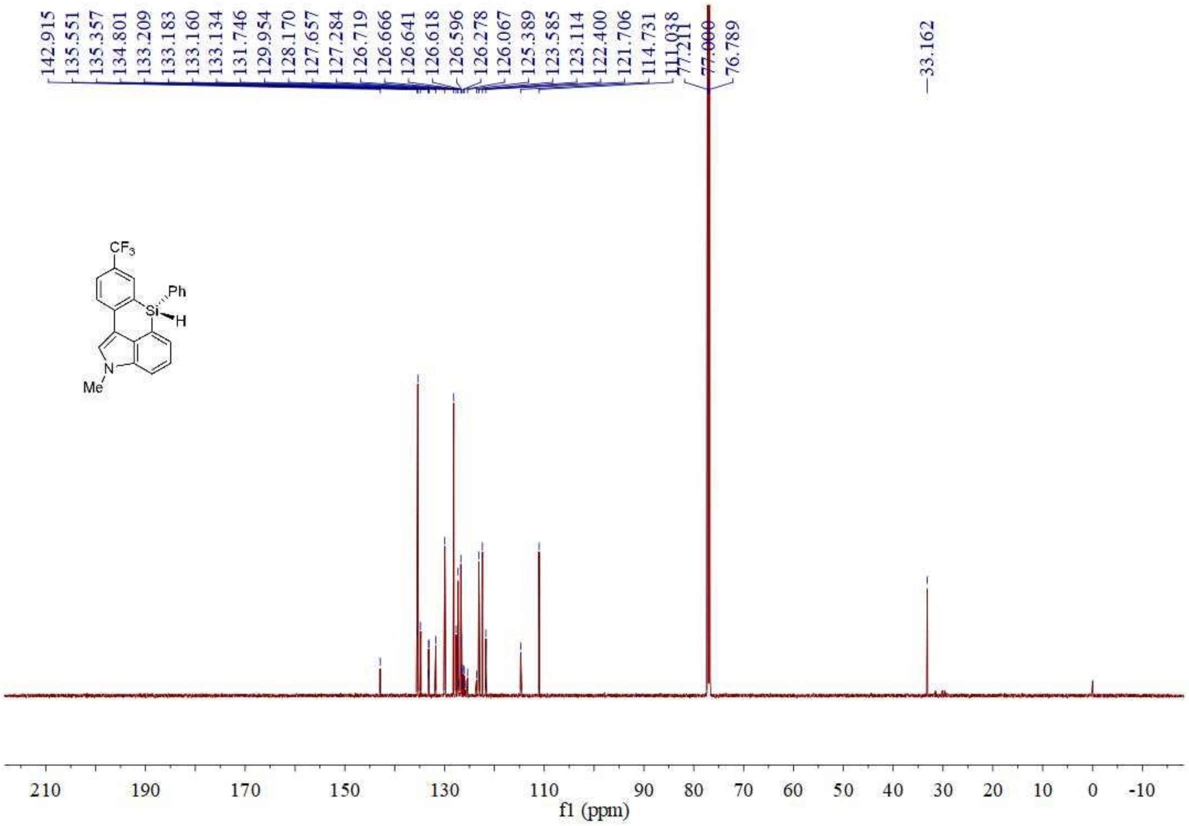
142.915
135.551
135.357
134.801
133.209
133.183
133.160
133.134
131.746
129.954
128.170
127.657
127.284
126.719
126.666
126.641
126.618
126.596
126.278
126.067
125.389
123.585
123.114
122.400
121.706
114.731
111.038
77.211
77.000
76.789
33.162
CF3
Ph
Si
H
N
Me
210 190 170 150 130 110 90 80 70 60 50 40 30 20 10 0 -10
f1 (ppm)

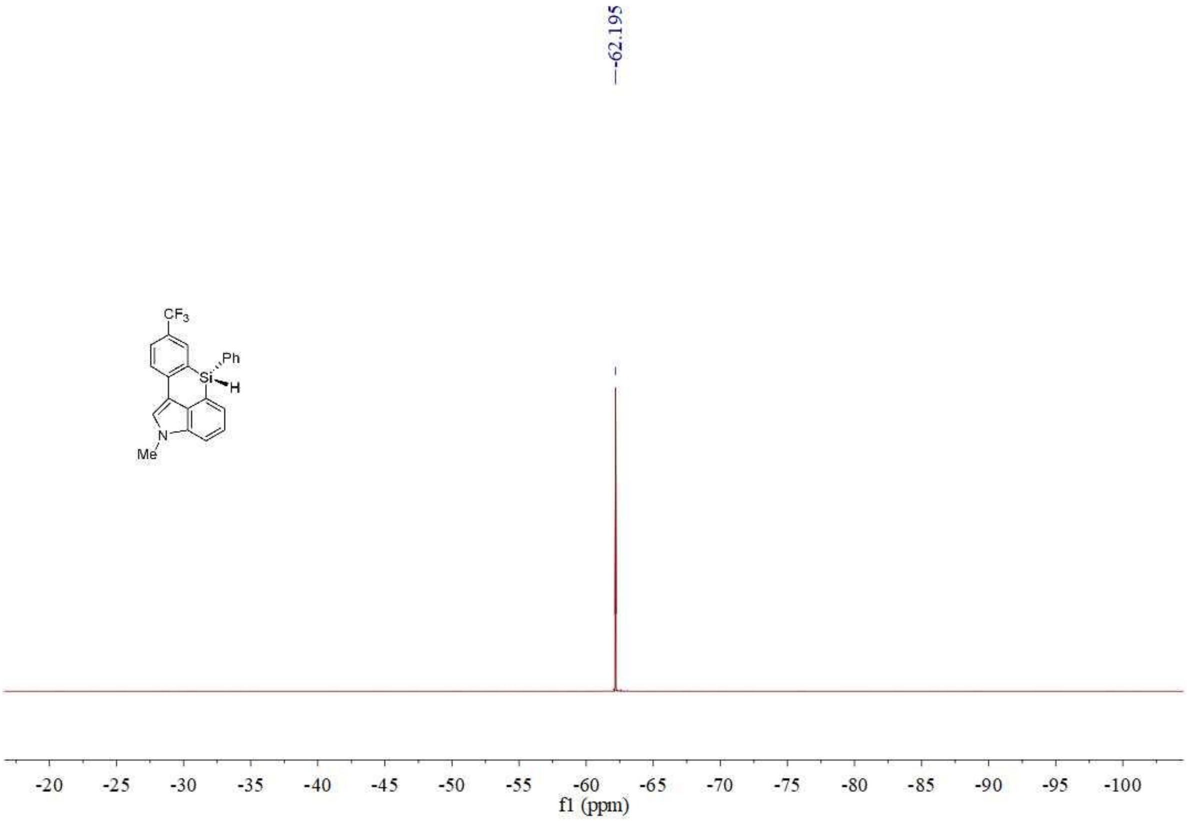

-62.195
CF3
Ph
Si
H
N
Me
-20
-25
-30
-35
-40
-45
-50
-55
-60
-65
-70
-75
-80
-85
-90
-95
-100
f1 (ppm)

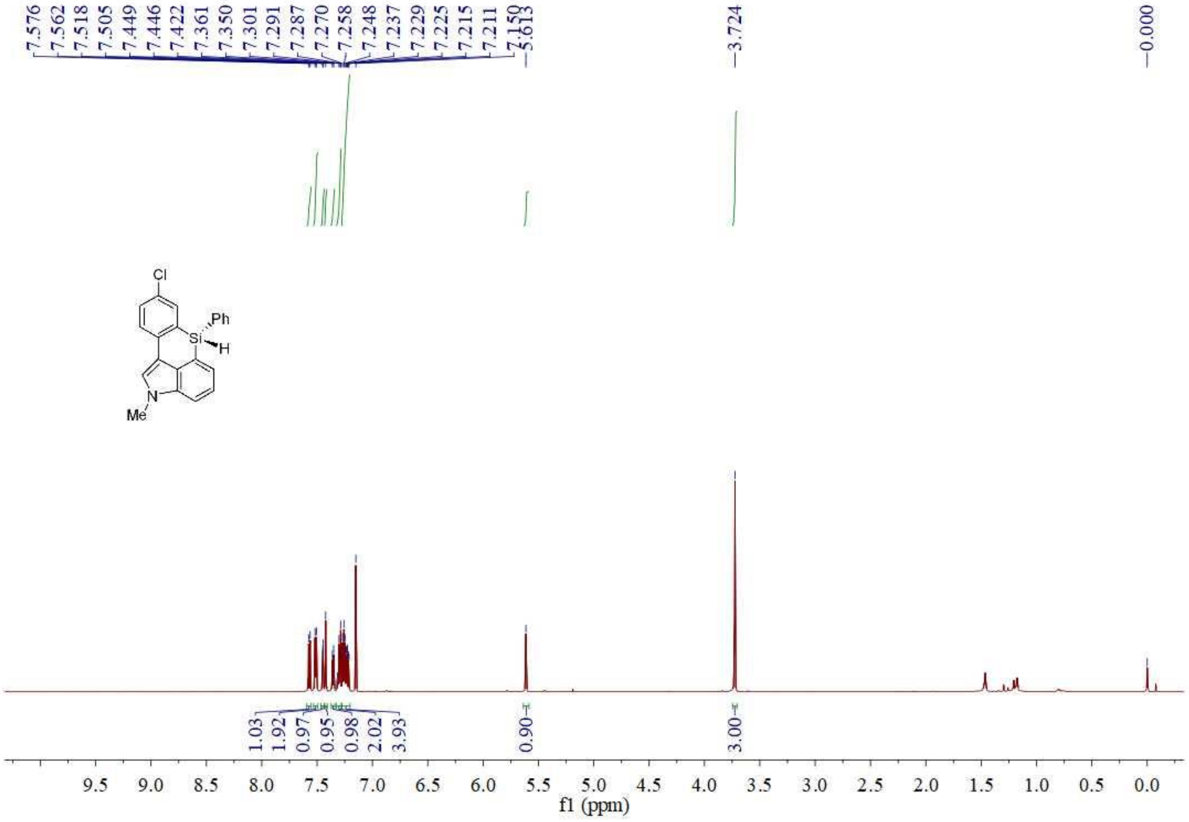
7.576
7.562
7.518
7.505
7.449
7.446
7.422
7.361
7.350
7.301
7.291
7.287
7.270
7.258
7.248
7.237
7.229
7.225
7.215
7.211
7.150
5.613
3.724
0.000
Cl
Ph
Si
H
N
Me
1.03
1.92
0.97
0.95
0.98
2.02
3.93
0.90
3.00
9.5 9.0 8.5 8.0 7.5 7.0 6.5 6.0 5.5 5.0 4.5 4.0 3.5 3.0 2.5 2.0 1.5 1.0 0.5 0.0
f1 (ppm)

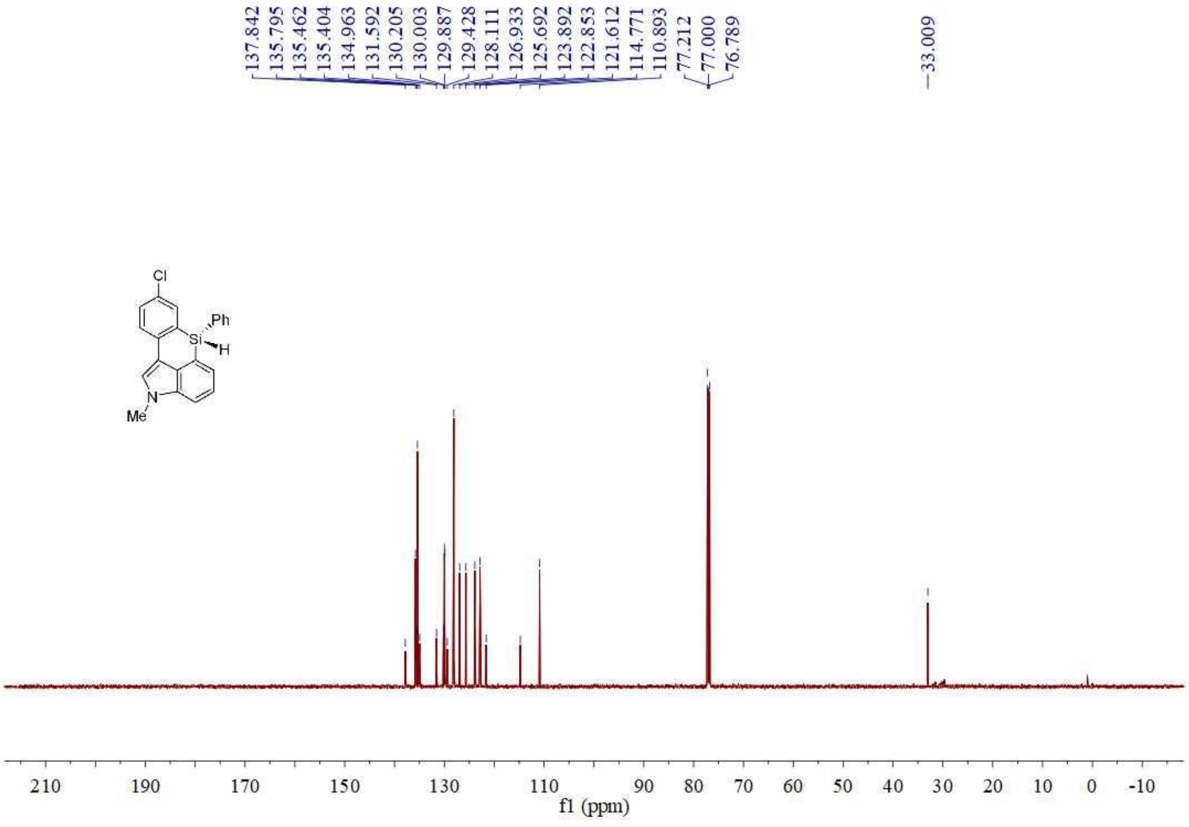
137.842
135.795
135.462
135.404
134.963
131.592
130.205
130.003
129.887
129.428
128.111
126.933
125.692
123.892
122.853
121.612
114.771
110.893
77.212
77.000
76.789
33.009
Cl
Ph
Si
H
N
Me
210 190 170 150 130 110 90 80 70 60 50 40 30 20 10 0 -10
f1 (ppm)

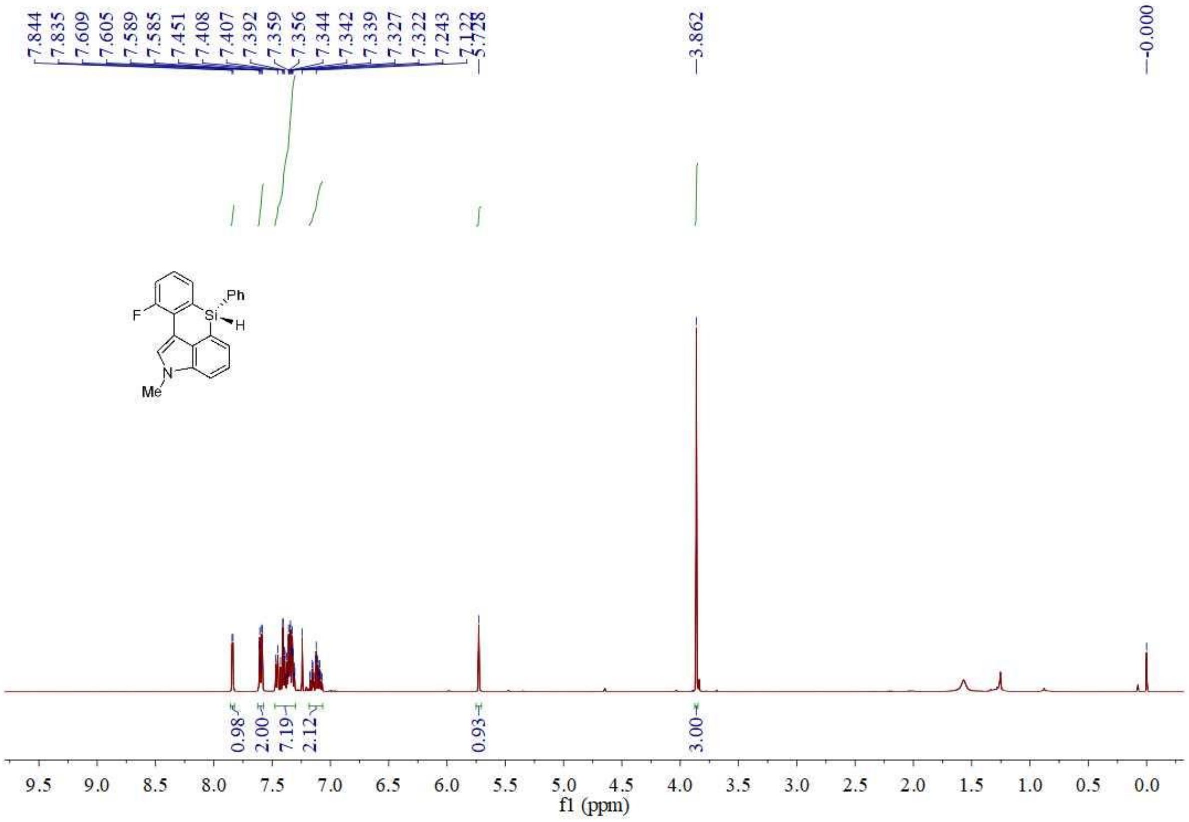
7.844
7.835
7.609
7.605
7.589
7.585
7.451
7.408
7.407
7.392
7.359
7.356
7.344
7.342
7.339
7.327
7.322
7.243
7.122
5.728
3.862
-0.000
Ph
F
Si
H
N
Me
0.98
2.00
7.19
2.12
0.93
3.00
9.5 9.0 8.5 8.0 7.5 7.0 6.5 6.0 5.5 5.0 4.5 4.0 3.5 3.0 2.5 2.0 1.5 1.0 0.5 0.0
f1 (ppm)

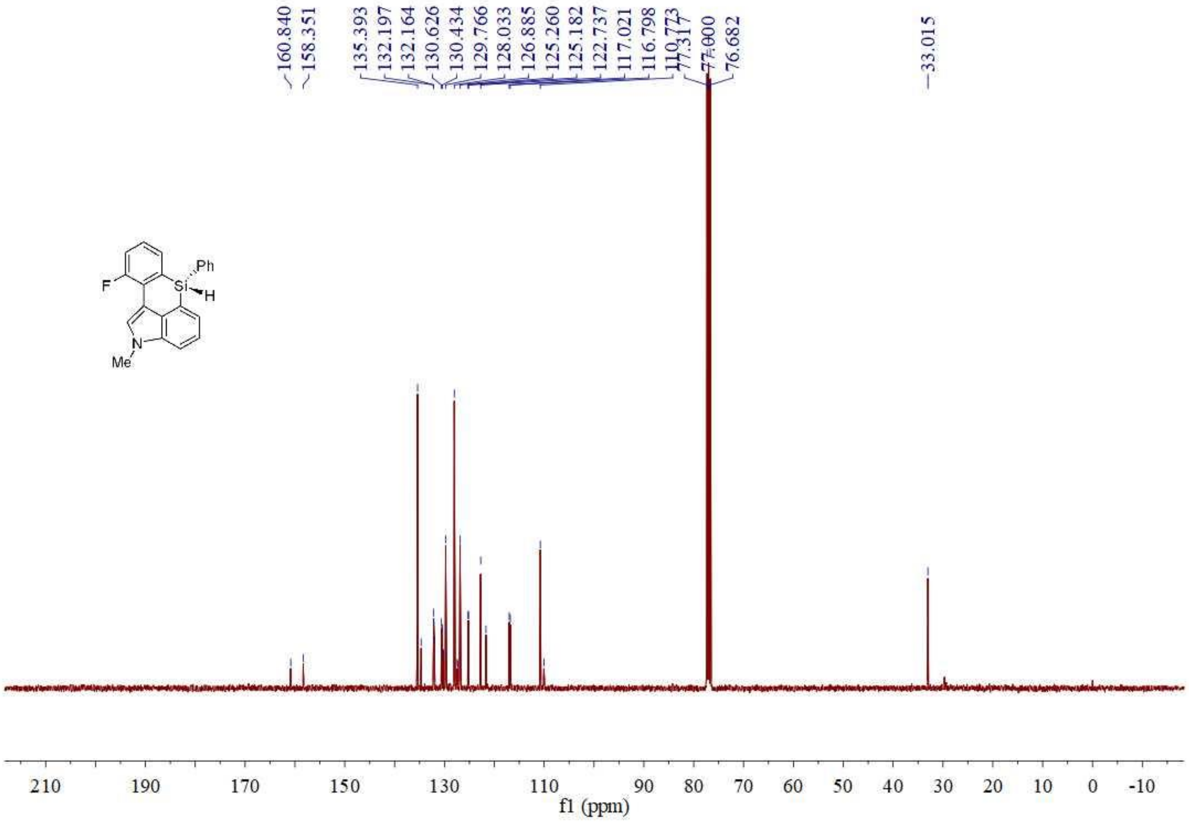
160.840
158.351
135.393
132.197
132.164
130.626
130.434
129.766
128.033
126.885
125.260
125.182
122.737
117.021
116.798
110.773
77.317
77.000
76.682
33.015
Ph
F
Si
H
N
Me
210 190 170 150 130 110 90 80 70 60 50 40 30 20 10 0 -10
f1 (ppm)

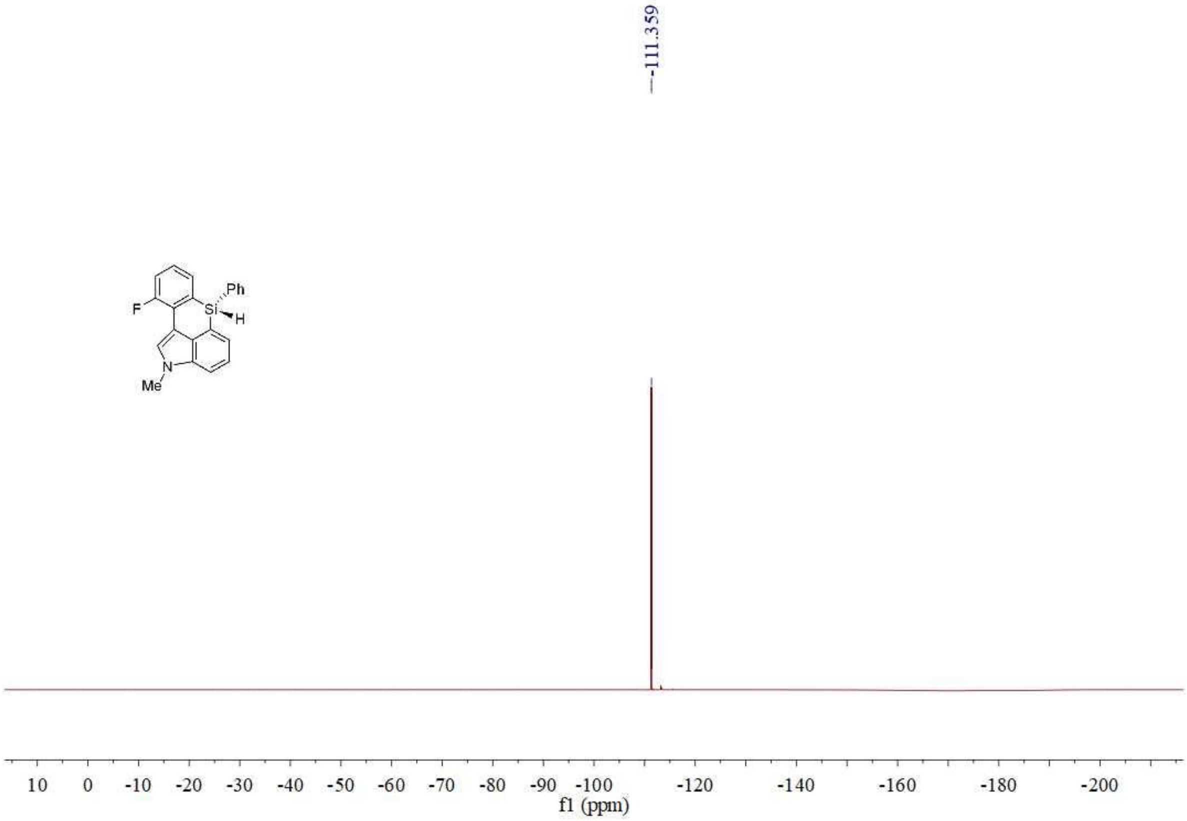

-111.359
F
Si
Ph
H
N
Me
10
0
-10
-20
-30
-40
-50
-60
-70
-80
-90
-100
-120
-140
-160
-180
-200
f1 (ppm)

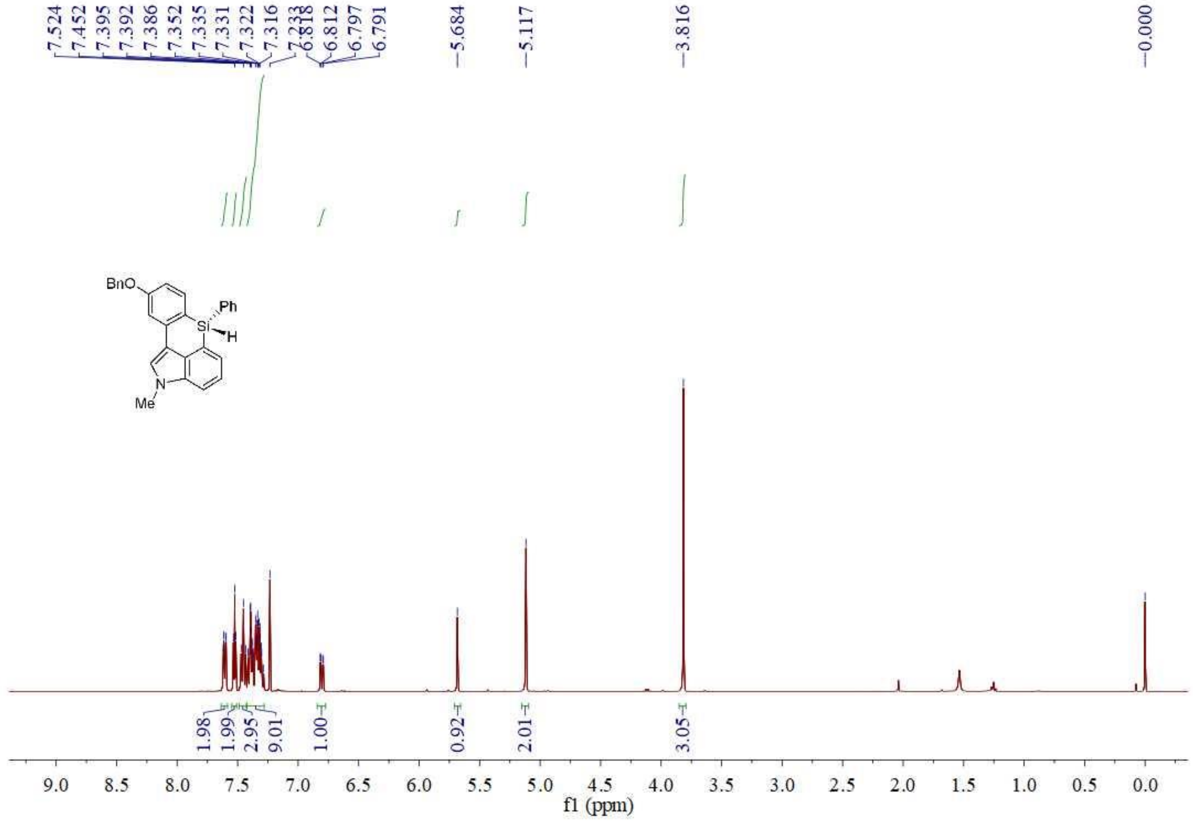
7.524
7.452
7.395
7.392
7.386
7.352
7.335
7.331
7.322
7.316
7.233
6.818
6.812
6.797
6.791
5.684
5.117
3.816
0.000
BnO
Si
Ph
H
N
Me
1.98
1.99
2.95
9.01
1.00
0.92
2.01
3.05
9.0 8.5 8.0 7.5 7.0 6.5 6.0 5.5 5.0 4.5 4.0 3.5 3.0 2.5 2.0 1.5 1.0 0.5 0.0
f1 (ppm)

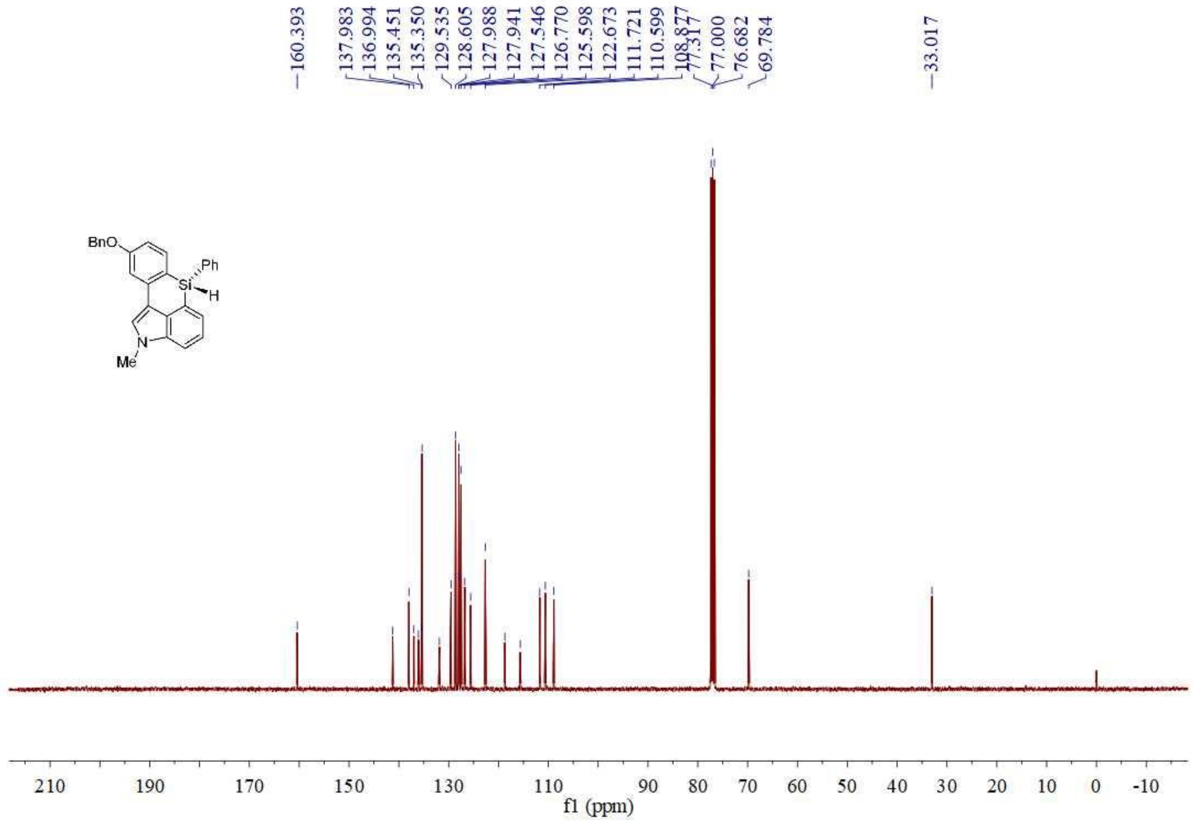
160.393
137.983
136.994
135.451
135.350
129.535
128.605
127.988
127.941
127.546
126.770
125.598
122.673
111.721
110.599
108.877
77.317
77.000
76.682
69.784
33.017
BnO
Si
Ph
H
N
Me
210 190 170 150 130 110 90 80 70 60 50 40 30 20 10 0 -10
f1 (ppm)

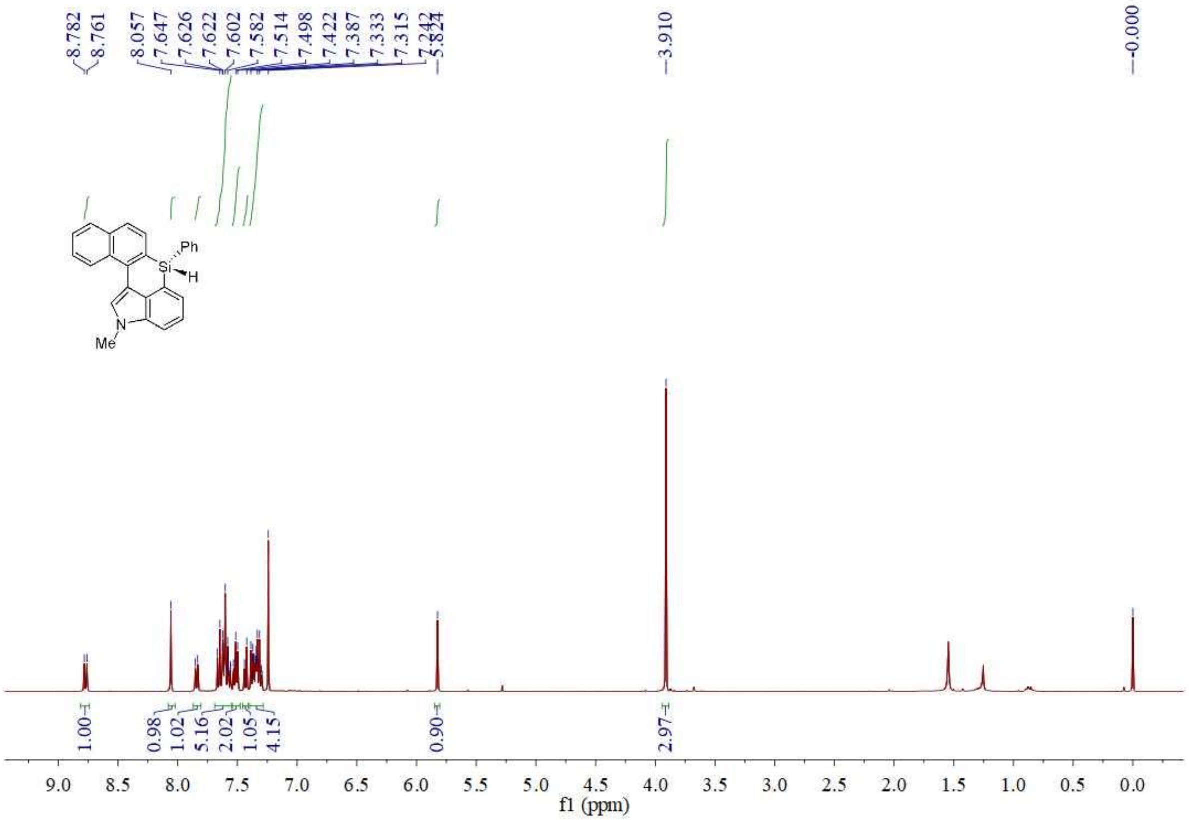

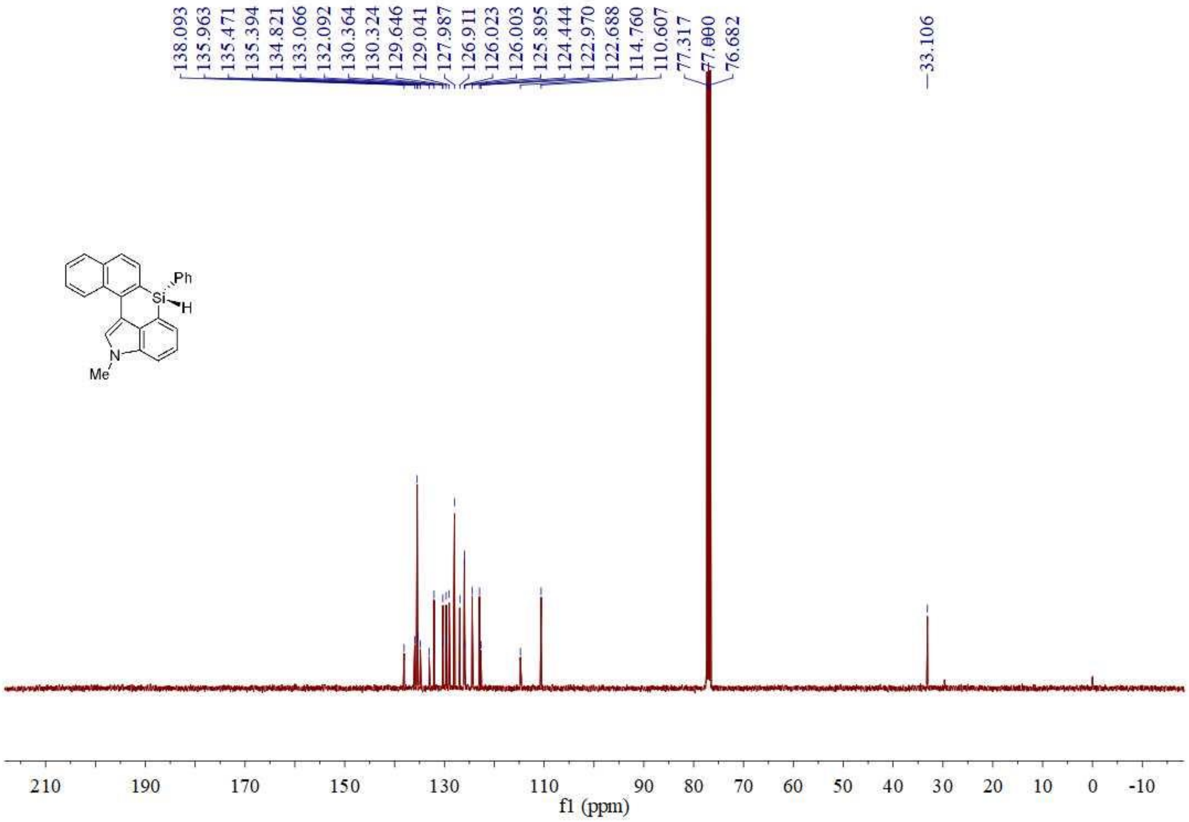

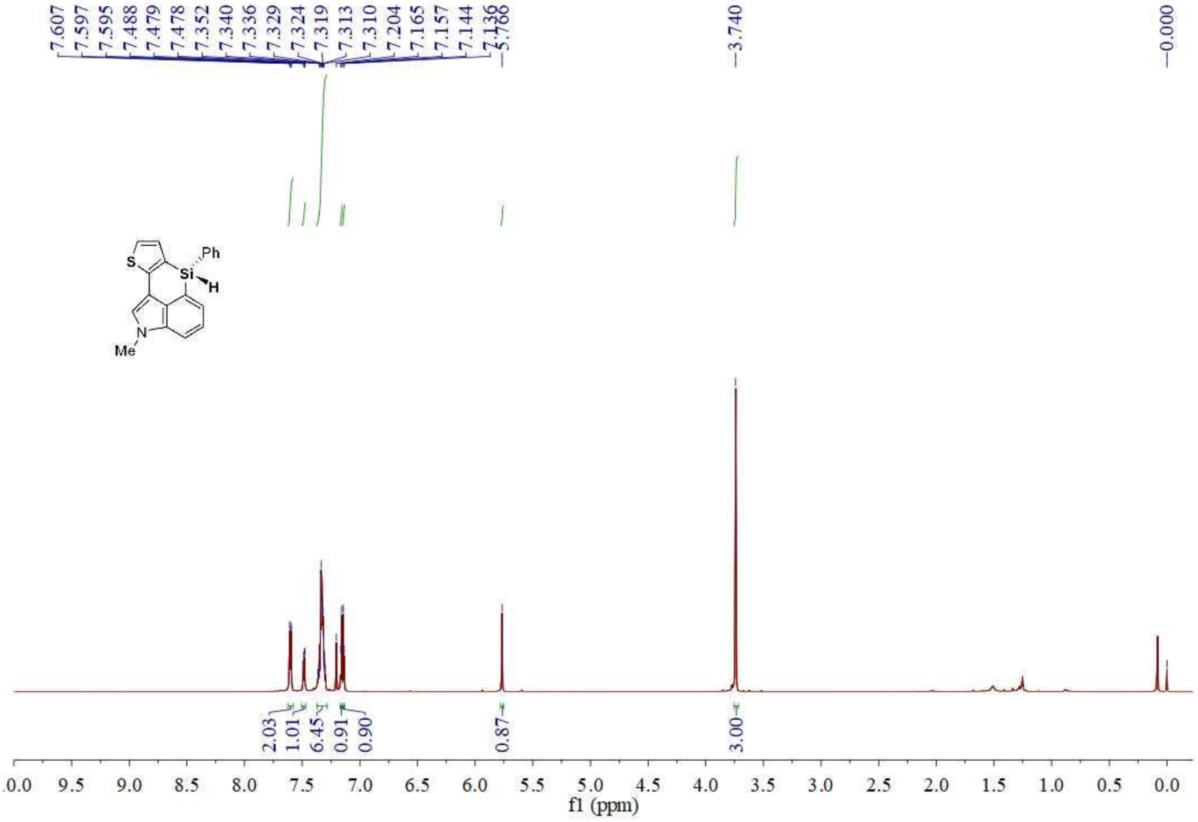

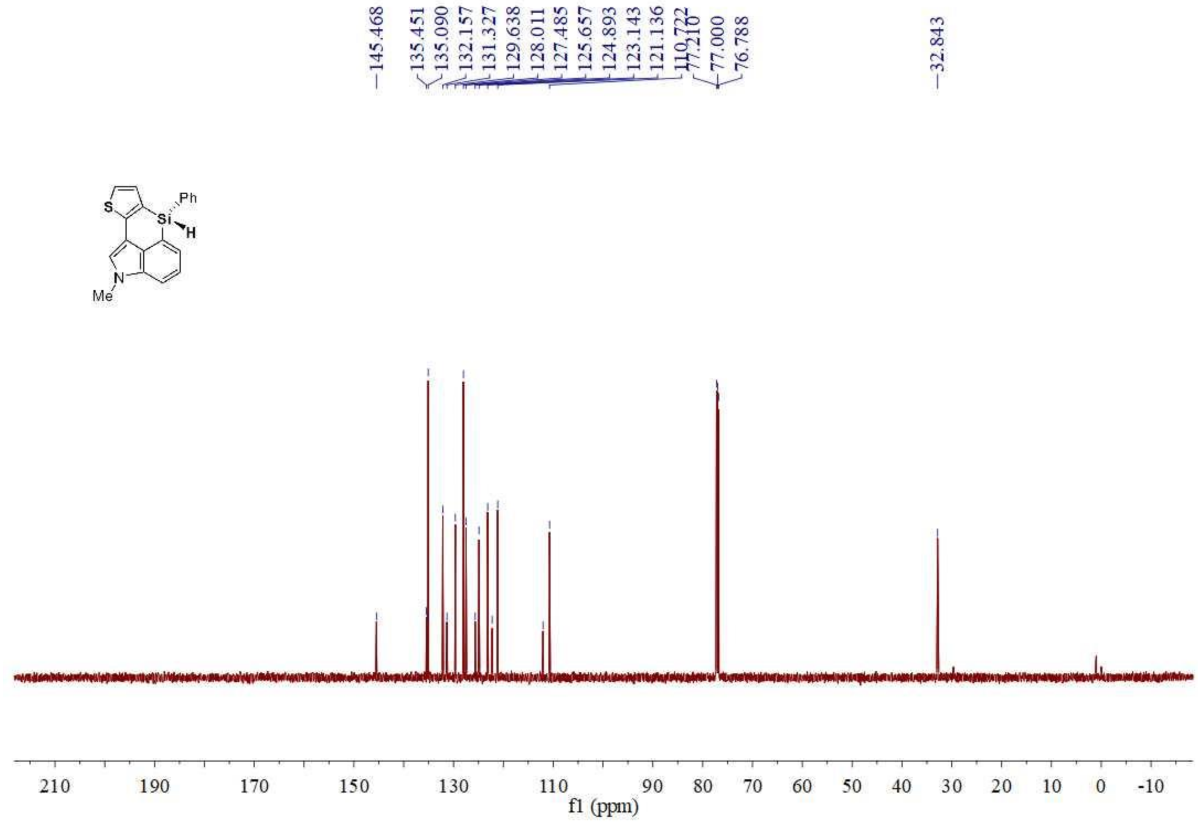

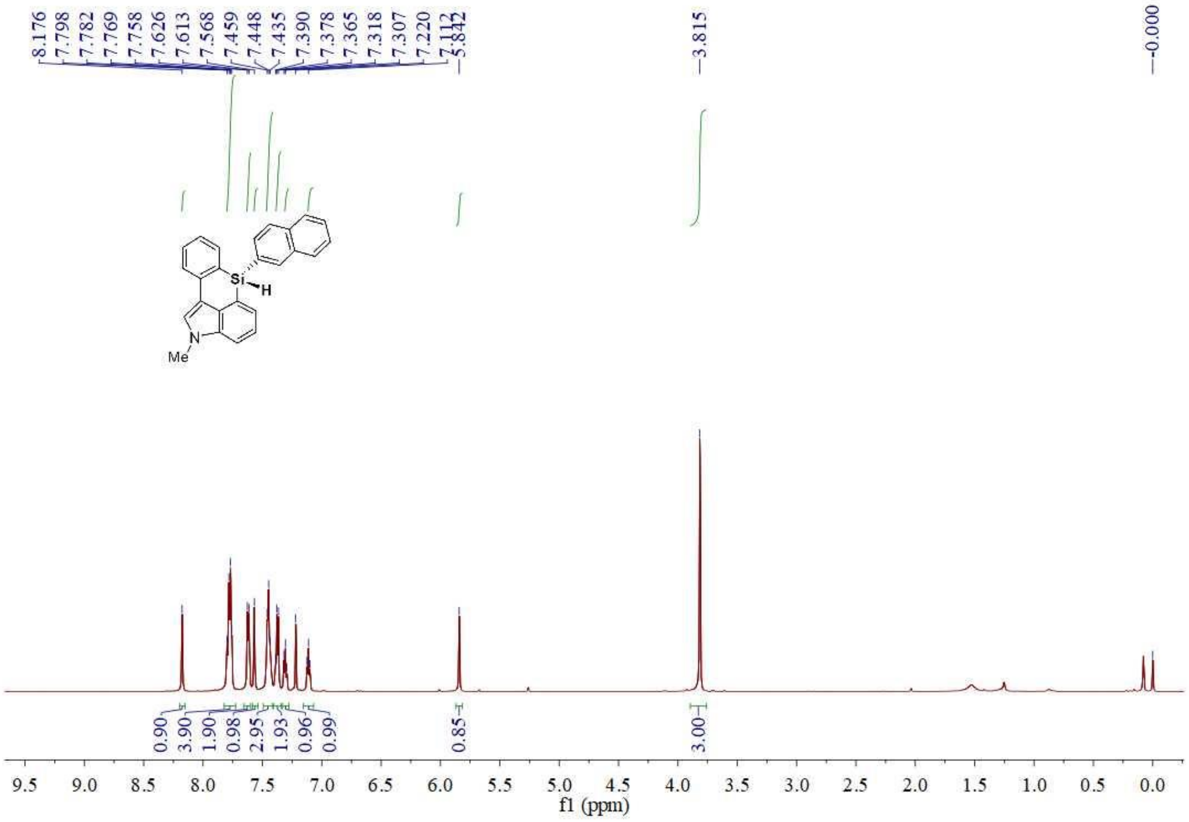

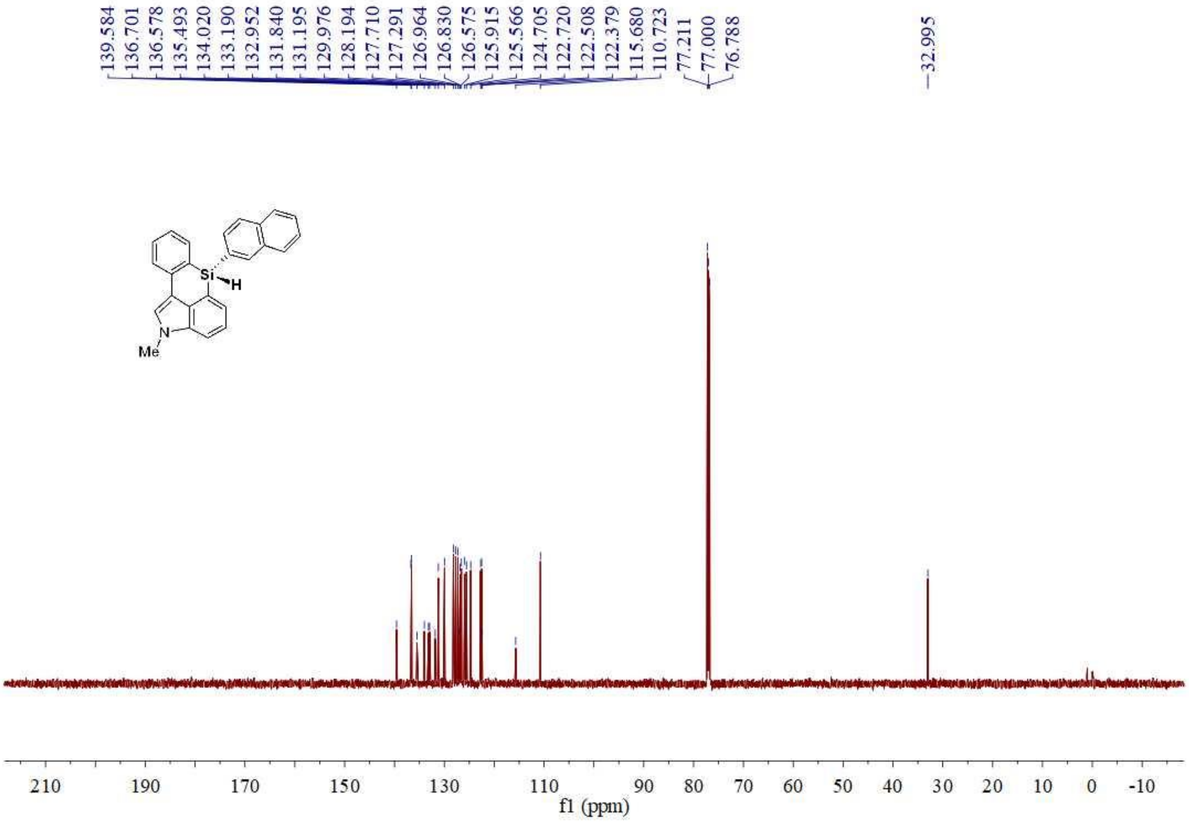

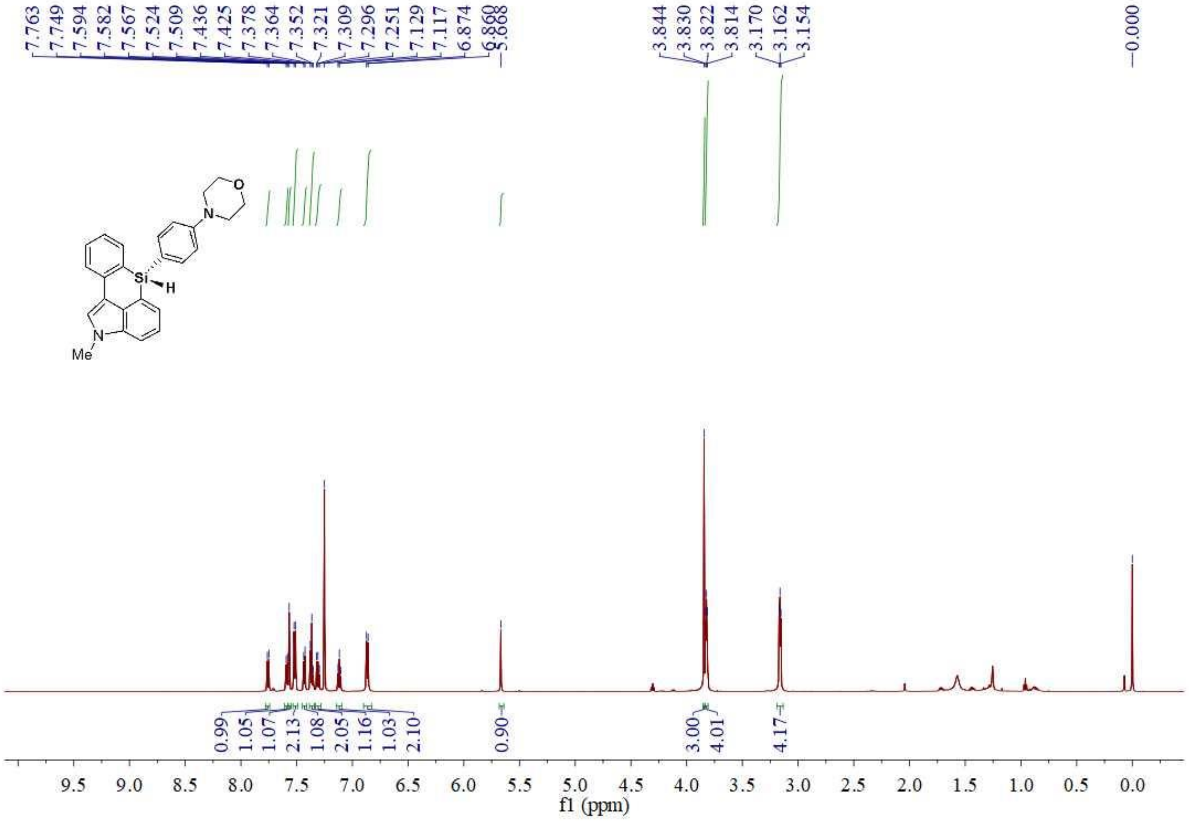
7.763
7.749
7.594
7.582
7.567
7.524
7.509
7.436
7.425
7.378
7.364
7.352
7.321
7.309
7.296
7.251
7.129
7.117
6.874
6.860
5.688
3.844
3.830
3.822
3.814
3.170
3.162
3.154
0.000
O
N
Si
H
N
Me
0.99
1.05
1.07
2.13
1.08
2.05
1.16
1.03
2.10
0.90
3.00
4.01
4.17
f1 (ppm)

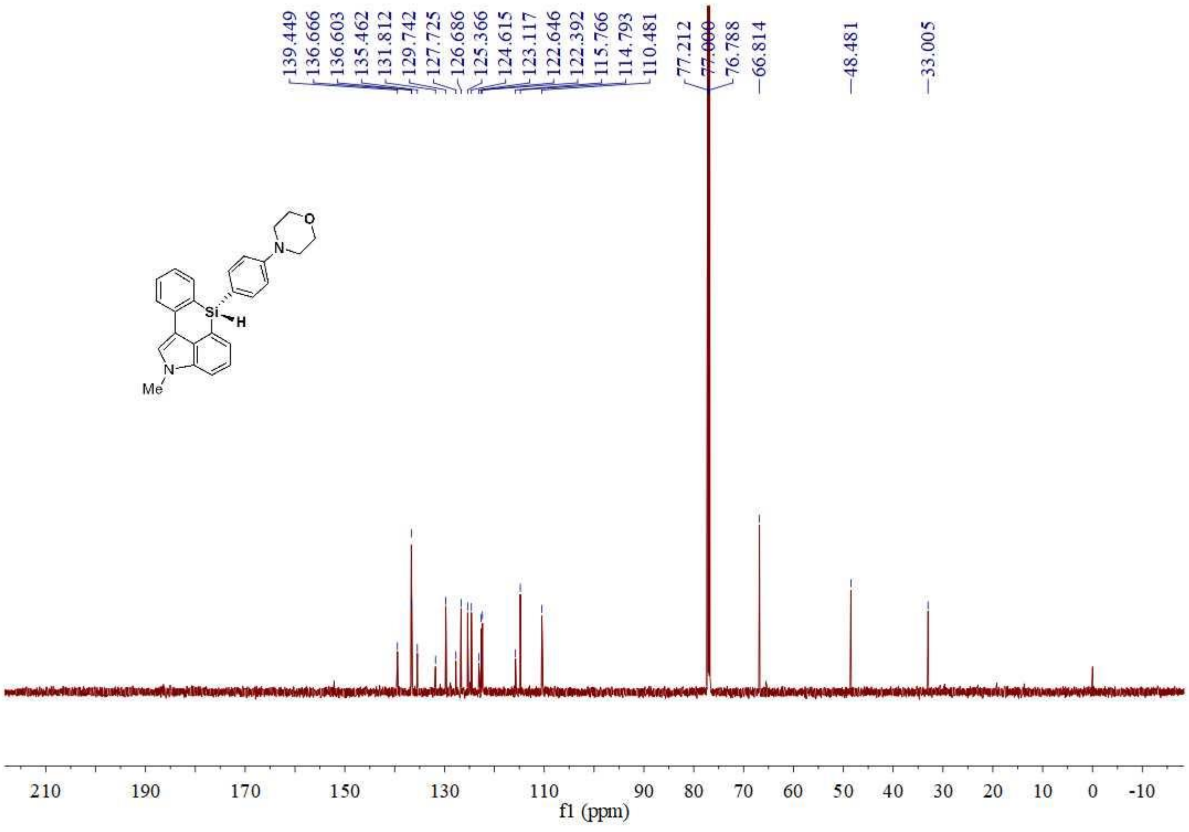
139.449
136.666
136.603
135.462
131.812
129.742
127.725
126.686
125.366
124.615
123.117
122.646
122.392
115.766
114.793
110.481
77.212
77.000
76.788
66.814
48.481
33.005
O
N
Si
H
N
Me
f1 (ppm)

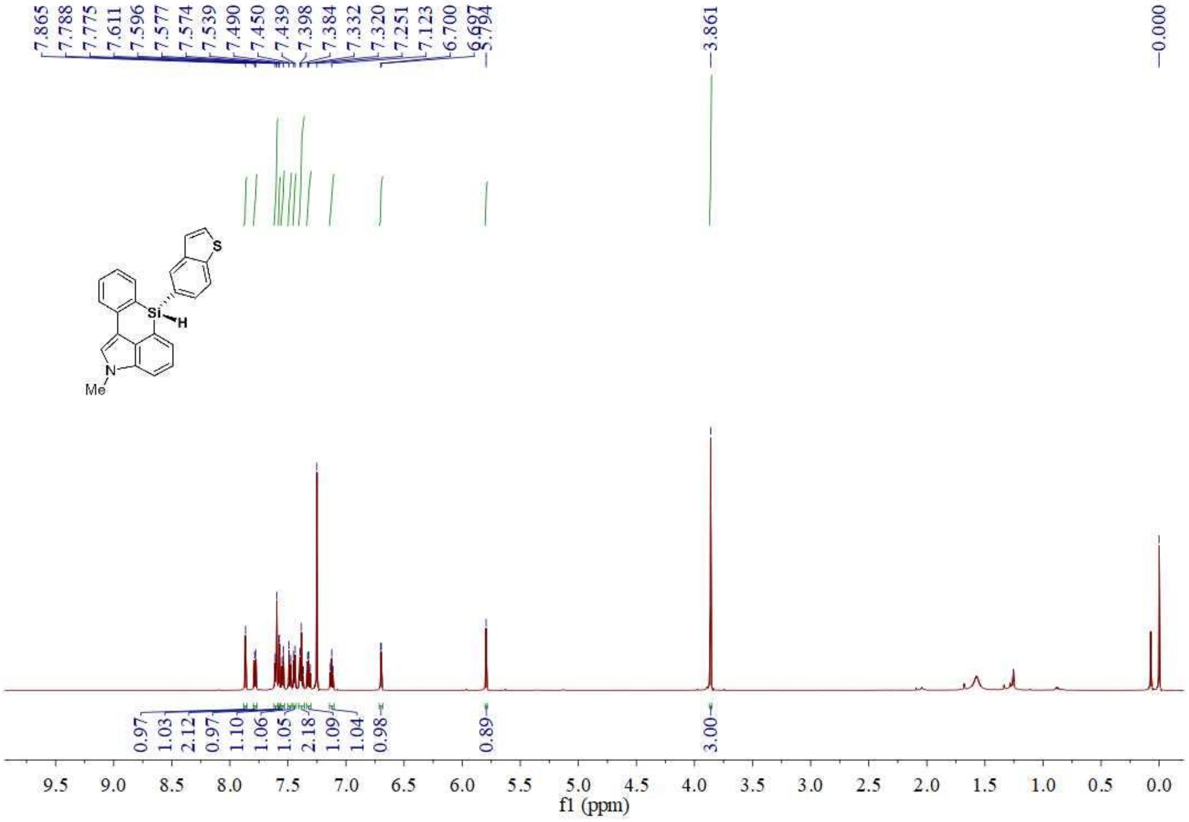

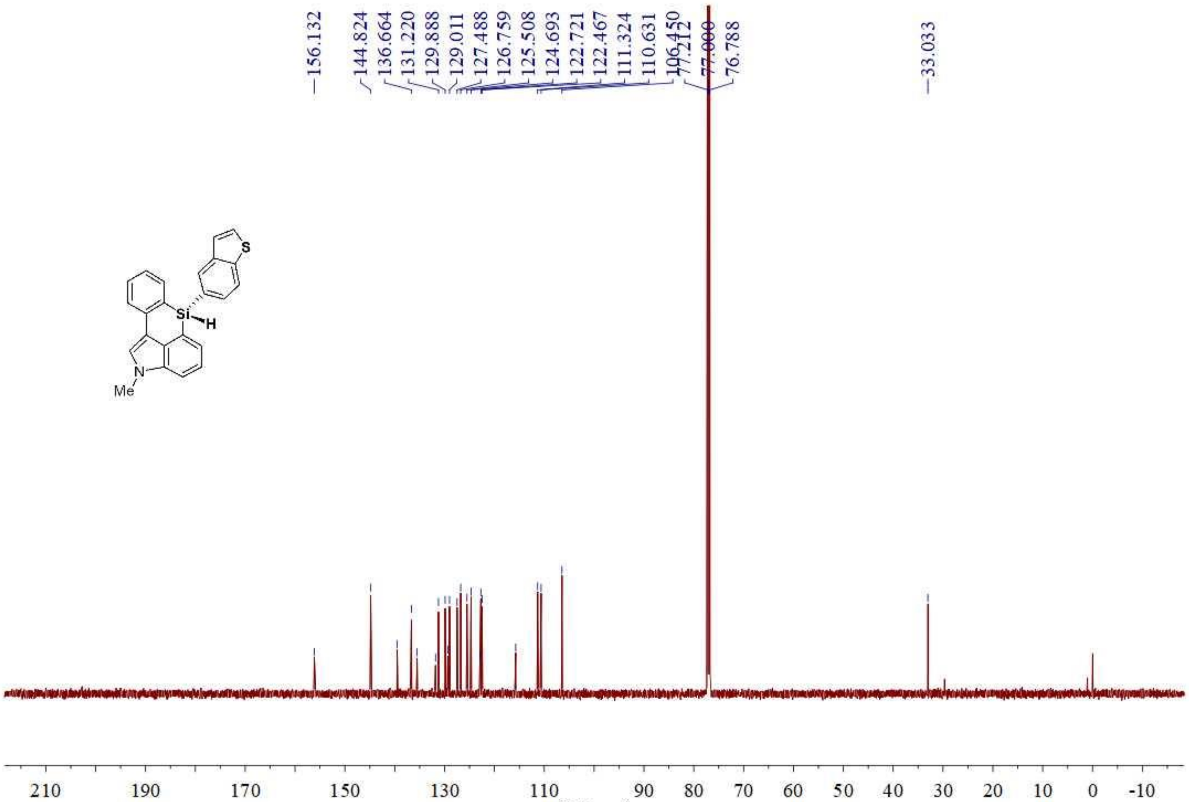

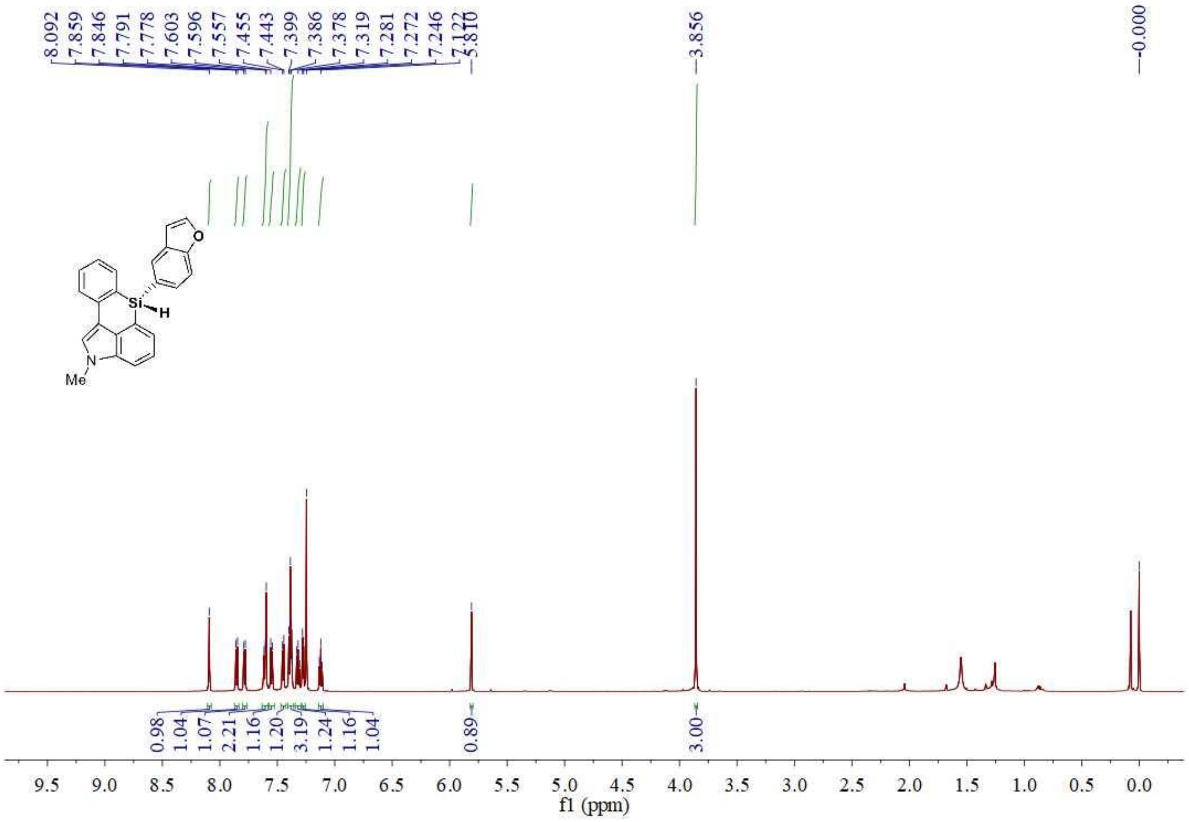
8.092
7.859
7.846
7.791
7.778
7.603
7.596
7.557
7.455
7.443
7.399
7.386
7.378
7.319
7.281
7.272
7.246
7.122
5.810
3.856
0.000
Si
H
O
N
Me
0.98
1.04
1.07
2.21
1.16
1.20
3.19
1.24
1.16
1.04
0.89
3.00
9.5 9.0 8.5 8.0 7.5 7.0 6.5 6.0 5.5 5.0 4.5 4.0 3.5 3.0 2.5 2.0 1.5 1.0 0.5 0.0
f1 (ppm)

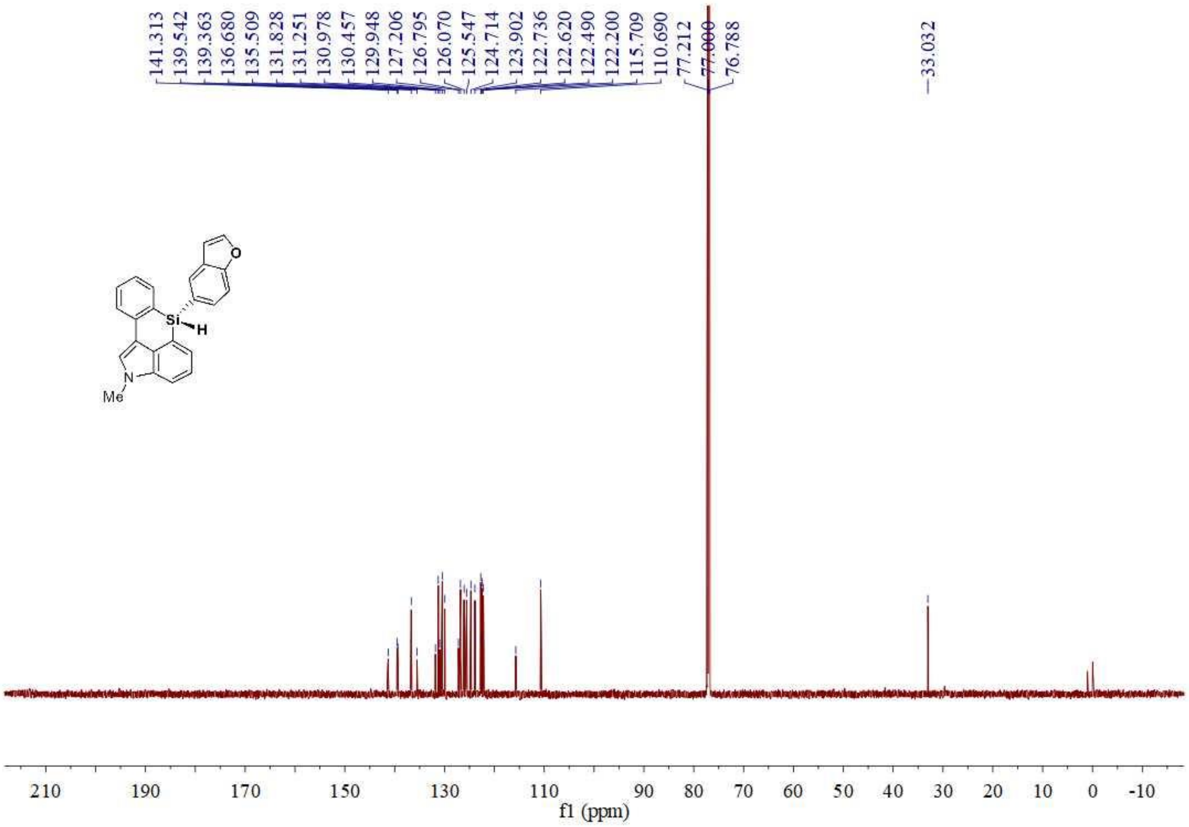
141.313
139.542
139.363
136.680
135.509
131.828
131.251
130.978
130.457
129.948
127.206
126.795
126.070
125.547
124.714
123.902
122.736
122.620
122.490
122.200
115.709
110.690
77.212
77.000
76.788
33.032
Si
H
O
N
Me
210 190 170 150 130 110 90 80 70 60 50 40 30 20 10 0 -10
f1 (ppm)

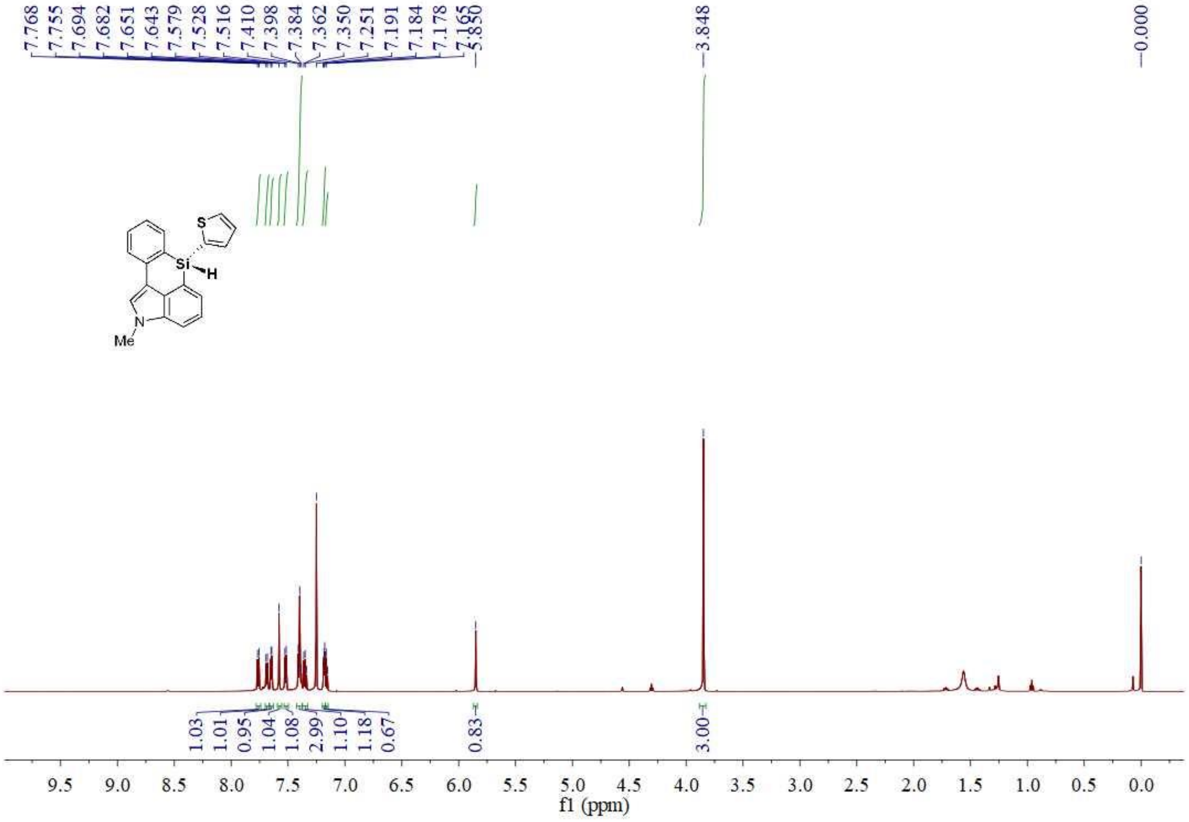
7.768
7.755
7.694
7.682
7.651
7.643
7.579
7.528
7.516
7.410
7.398
7.384
7.362
7.350
7.251
7.191
7.184
7.178
7.165
5.850
3.848
0.000
1.03
1.01
0.95
1.04
1.08
2.99
1.10
1.18
0.67
0.83
3.00
f1 (ppm)

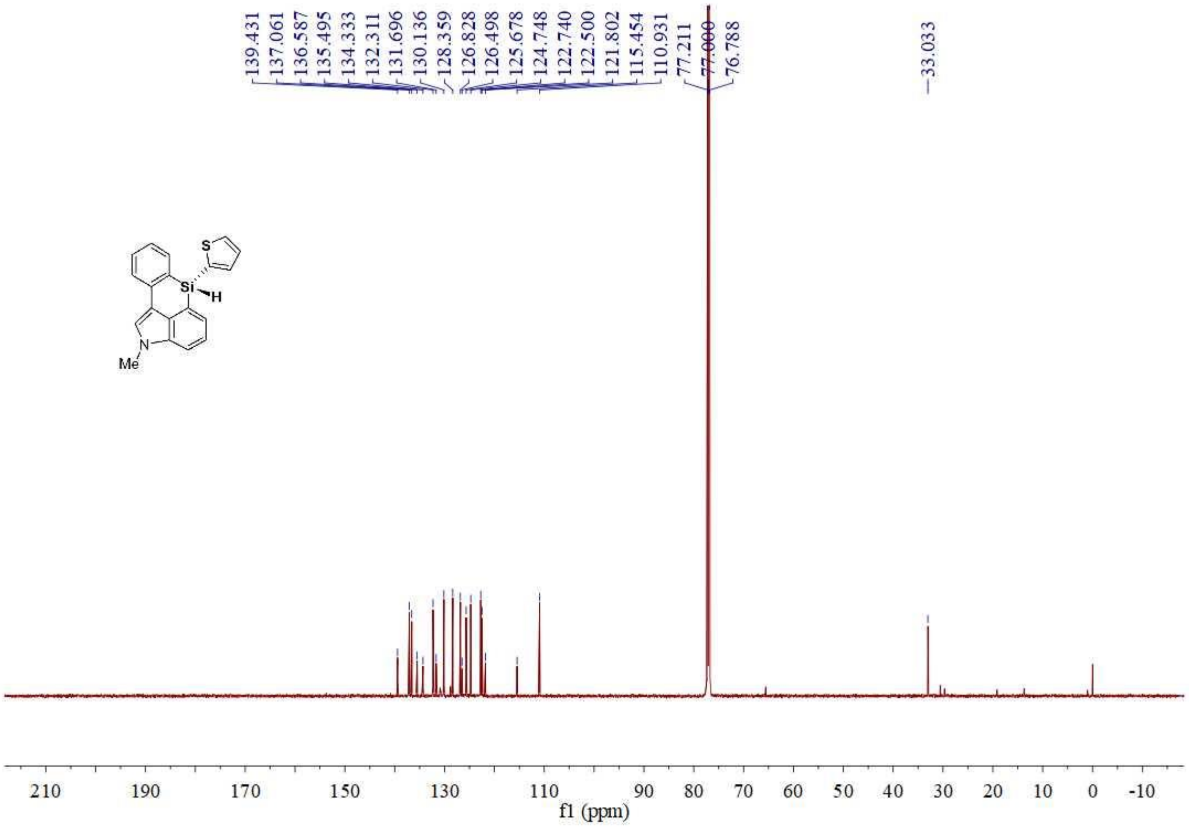
139.431
137.061
136.587
135.495
134.333
132.311
131.696
130.136
128.359
126.828
126.498
125.678
124.748
122.740
122.500
121.802
115.454
110.931
77.211
77.000
76.788
33.033
f1 (ppm)

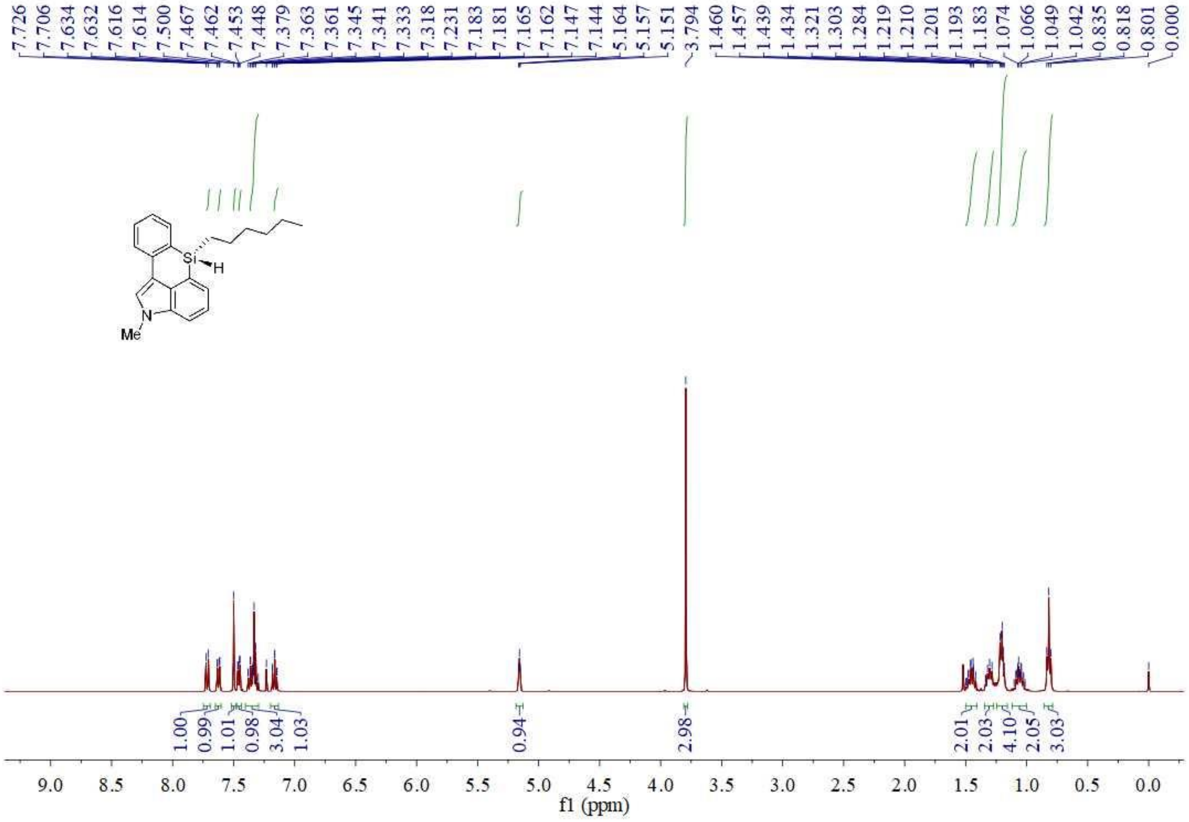

7.726 7.706 7.634 7.632 7.616 7.614 7.500 7.467 7.462 7.453 7.448 7.379 7.363 7.361 7.345 7.341 7.333 7.318 7.231 7.183 7.181 7.165 7.162 7.147 7.144 5.164 5.157 5.151 3.794 1.460 1.457 1.439 1.434 1.321 1.303 1.284 1.219 1.210 1.201 1.193 1.183 1.074 1.066 1.049 1.042 0.835 0.818 0.801 0.000
Si
H
N
Me
1.00 0.99 1.01 0.98 3.04 1.03 0.94 2.98 2.01 2.03 4.10 2.05 3.03
9.0 8.5 8.0 7.5 7.0 6.5 6.0 5.5 5.0 4.5 4.0 3.5 3.0 2.5 2.0 1.5 1.0 0.5 0.0
f1 (ppm)

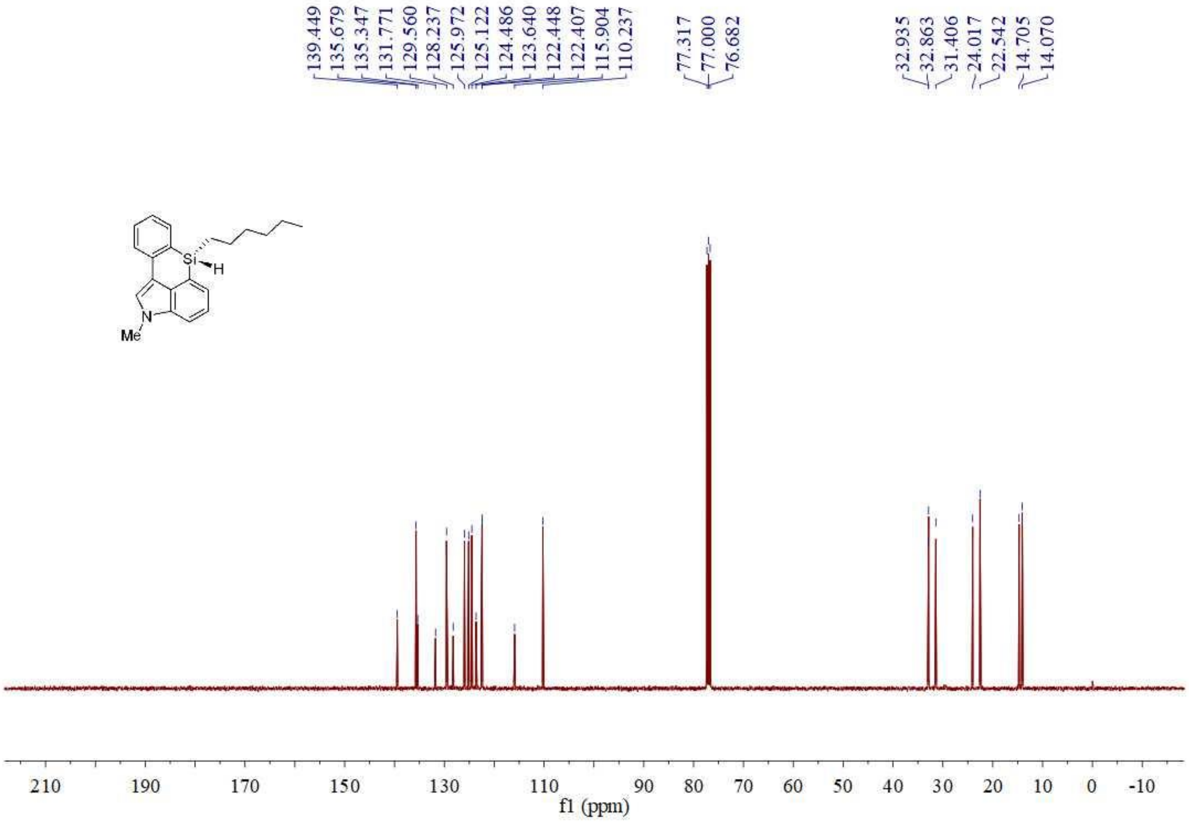

139.449 135.679 135.347 131.771 129.560 128.237 125.972 125.122 124.486 123.640 122.448 122.407 115.904 110.237 77.317 77.000 76.682 32.935 32.863 31.406 24.017 22.542 14.705 14.070
Si
H
N
Me
210 190 170 150 130 110 90 80 70 60 50 40 30 20 10 0 -10
f1 (ppm)

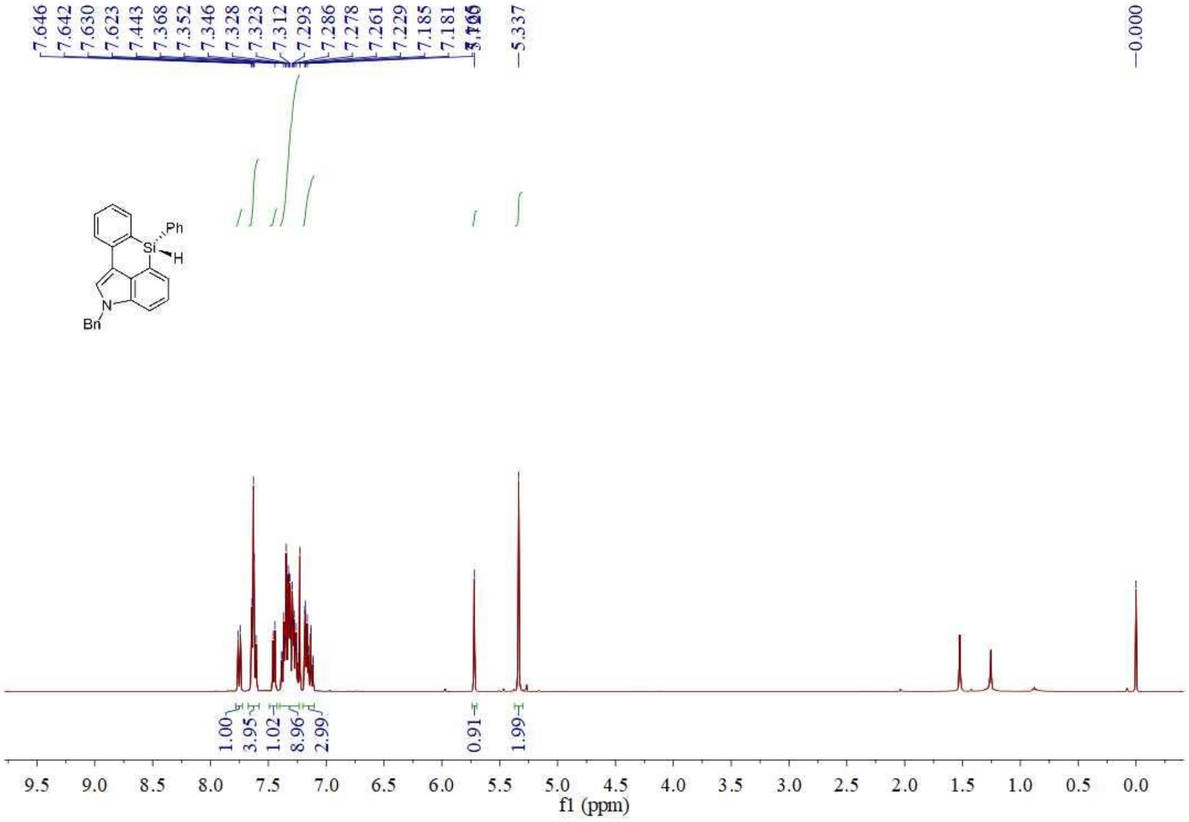

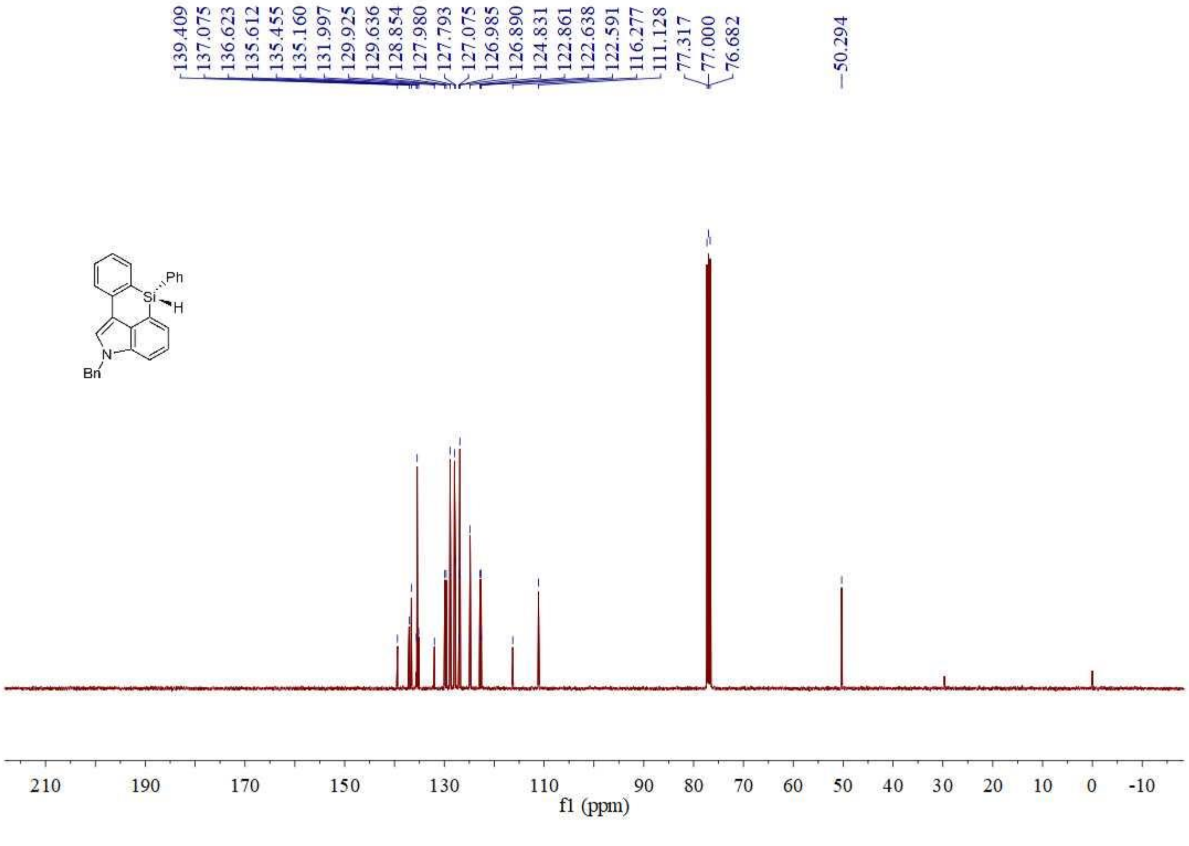

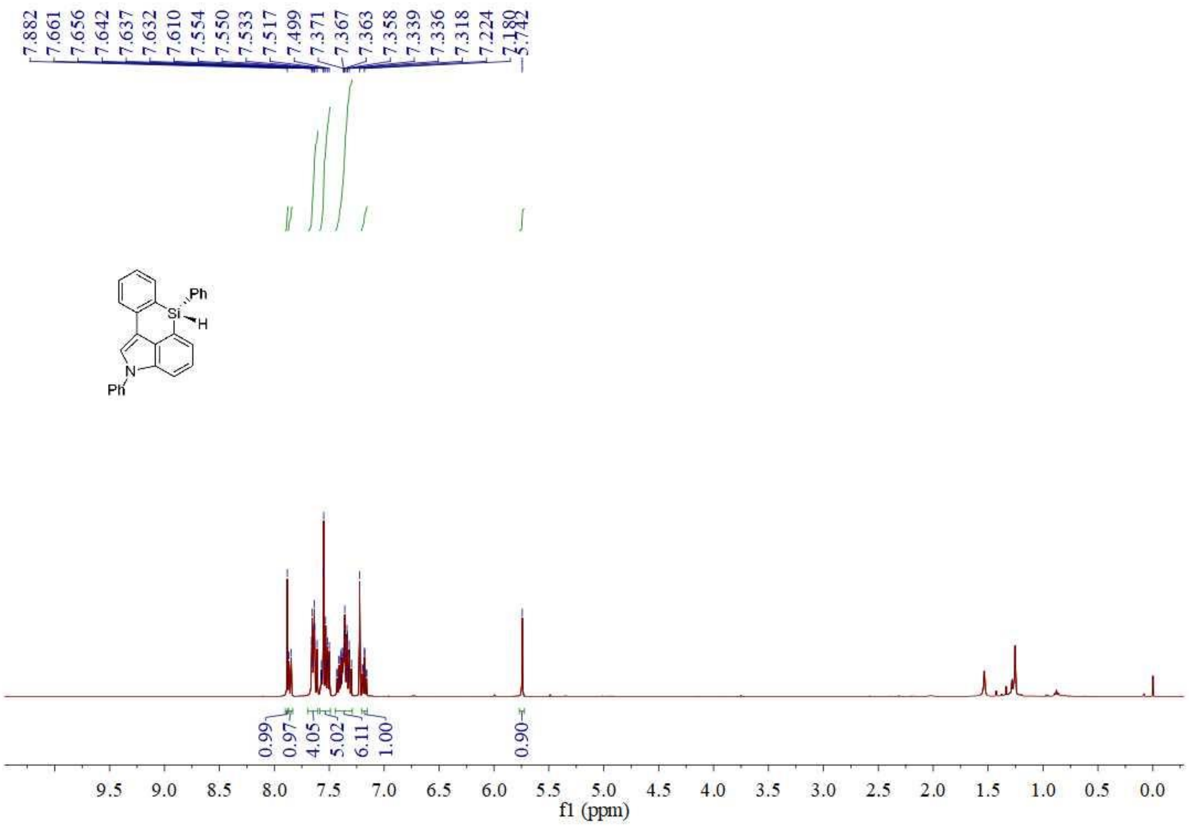
7.882
7.661
7.656
7.642
7.637
7.632
7.610
7.554
7.550
7.533
7.517
7.499
7.371
7.367
7.363
7.358
7.339
7.336
7.318
7.224
7.180
5.742
Ph
Si
H
N
Ph
0.99
0.97
4.05
5.02
6.11
1.00
0.90
9.5 9.0 8.5 8.0 7.5 7.0 6.5 6.0 5.5 5.0 4.5 4.0 3.5 3.0 2.5 2.0 1.5 1.0 0.5 0.0
f1 (ppm)

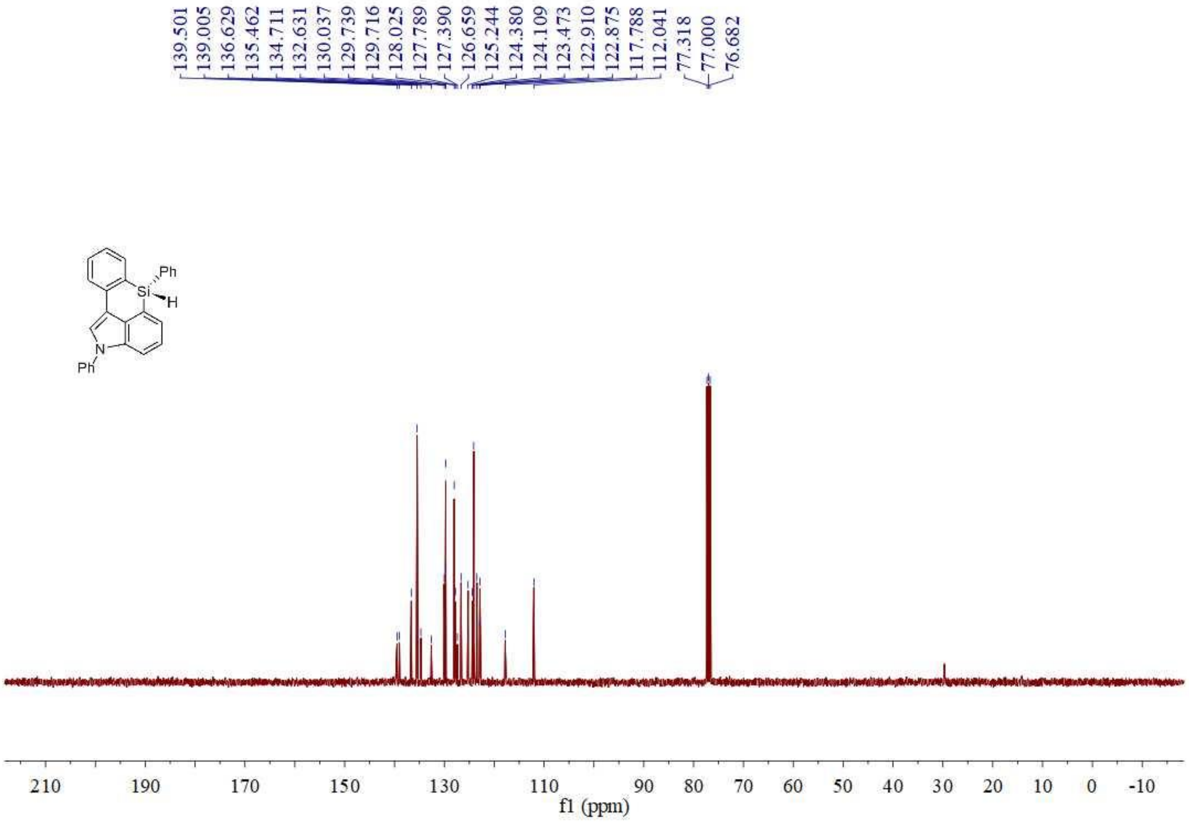
139.501
139.005
136.629
135.462
134.711
132.631
130.037
129.739
129.716
128.025
127.789
127.390
126.659
125.244
124.380
124.109
123.473
122.910
122.875
117.788
112.041
77.318
77.000
76.682
Ph
Si
H
N
Ph
210 190 170 150 130 110 90 80 70 60 50 40 30 20 10 0 -10
f1 (ppm)

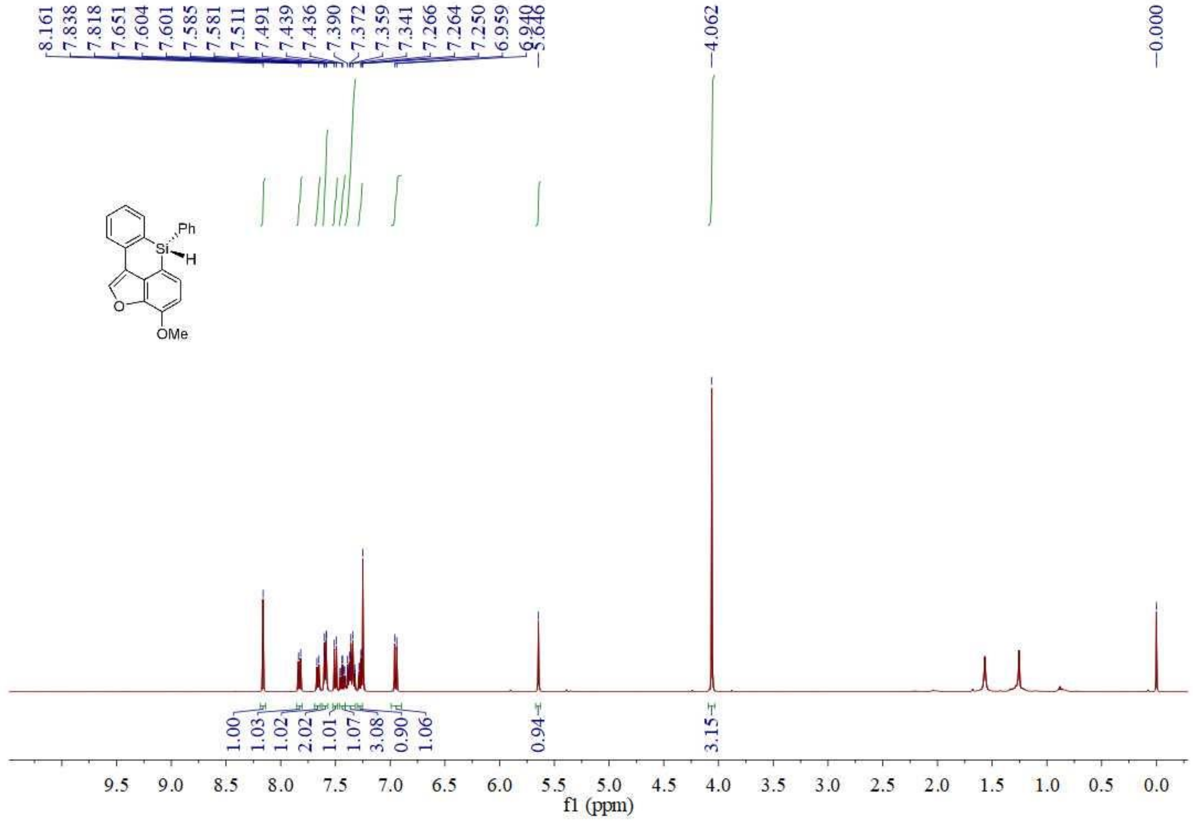
8.161
7.838
7.818
7.651
7.604
7.601
7.585
7.581
7.511
7.491
7.439
7.436
7.390
7.372
7.359
7.341
7.266
7.264
7.250
6.959
6.940
5.646
4.062
0.000
Ph
Si
H
O
OMe
1.00
1.03
1.02
2.02
1.01
1.07
3.08
0.90
1.06
0.94
3.15
9.5 9.0 8.5 8.0 7.5 7.0 6.5 6.0 5.5 5.0 4.5 4.0 3.5 3.0 2.5 2.0 1.5 1.0 0.5 0.0
f1 (ppm)

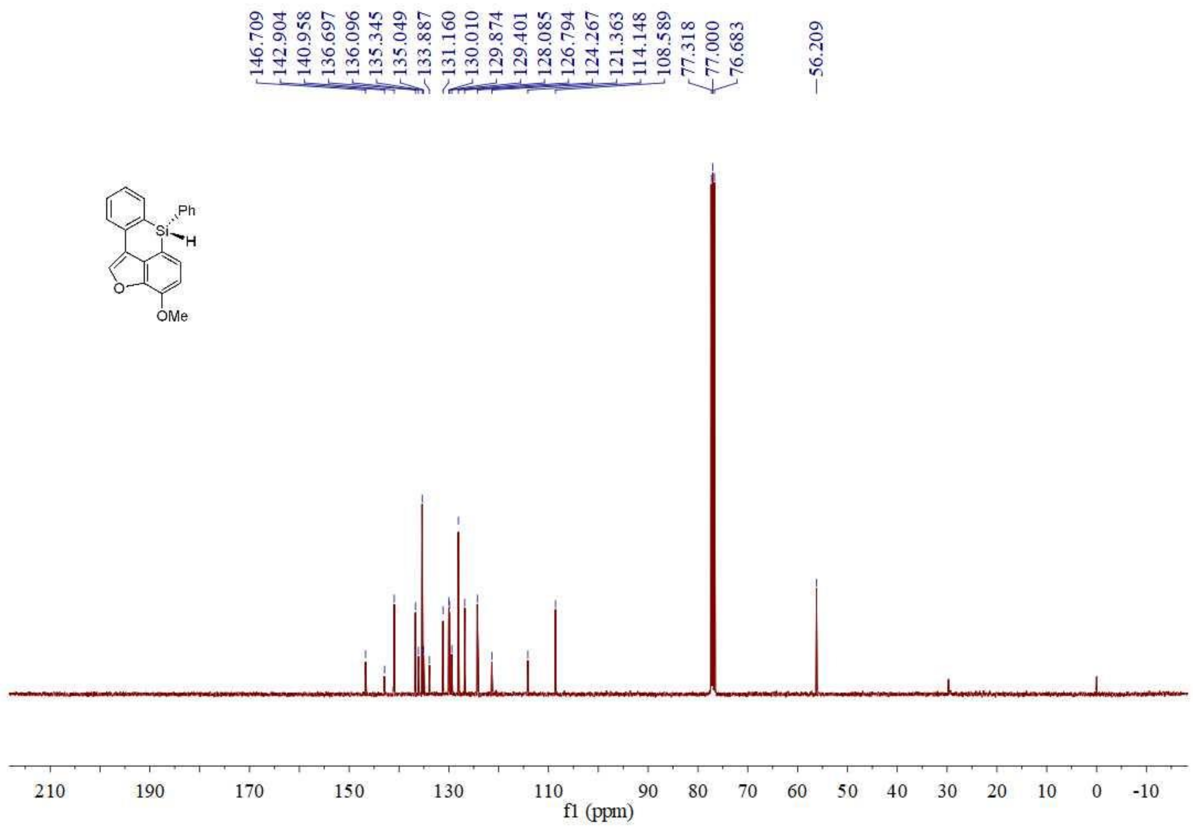
146.709
142.904
140.958
136.697
136.096
135.345
135.049
133.887
131.160
130.010
129.874
129.401
128.085
126.794
124.267
121.363
114.148
108.589
77.318
77.000
76.683
56.209
Ph
Si
H
O
OMe
210 190 170 150 130 110 90 80 70 60 50 40 30 20 10 0 -10
f1 (ppm)

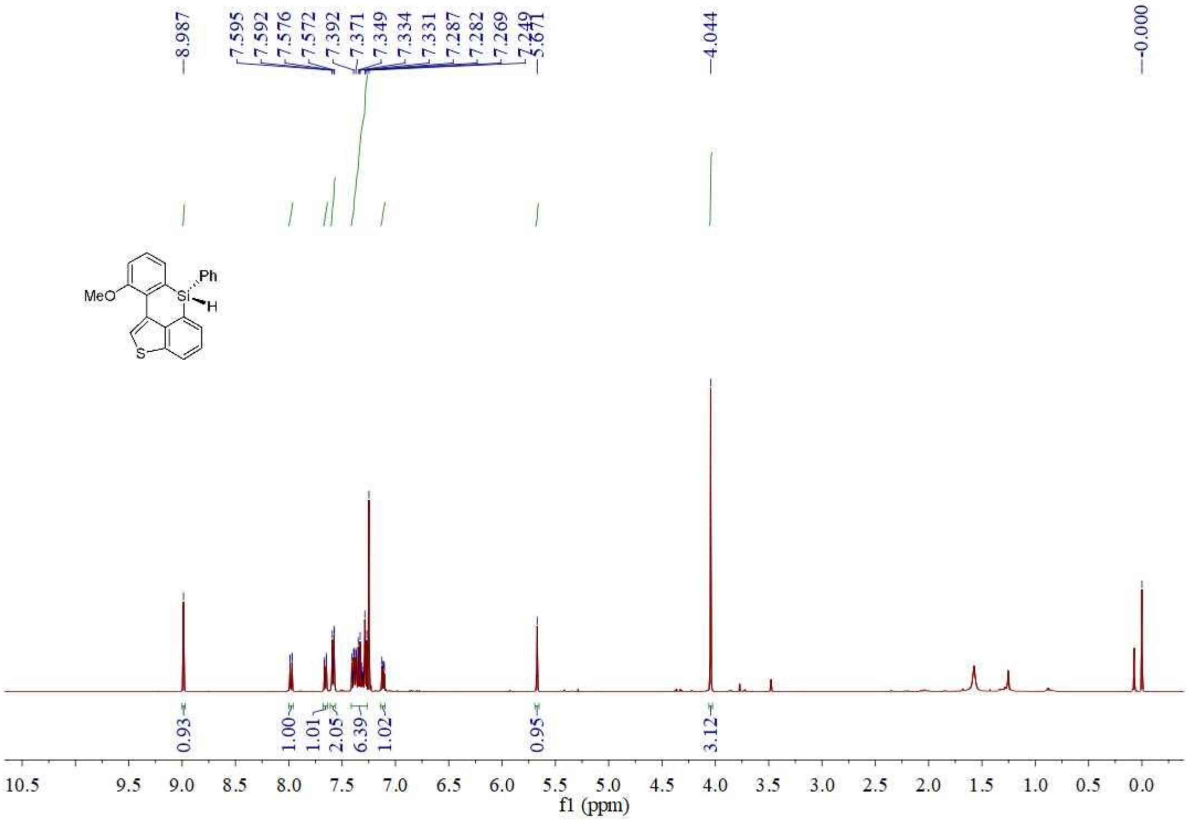
8.987
7.595
7.592
7.576
7.572
7.392
7.371
7.349
7.334
7.331
7.287
7.282
7.269
7.249
5.671
4.044
0.000
MeO
Si
Ph
H
S
0.93
1.00
1.01
2.05
6.39
1.02
0.95
3.12
10.5 9.5 9.0 8.5 8.0 7.5 7.0 6.5 6.0 5.5 5.0 4.5 4.0 3.5 3.0 2.5 2.0 1.5 1.0 0.5 0.0
f1 (ppm)

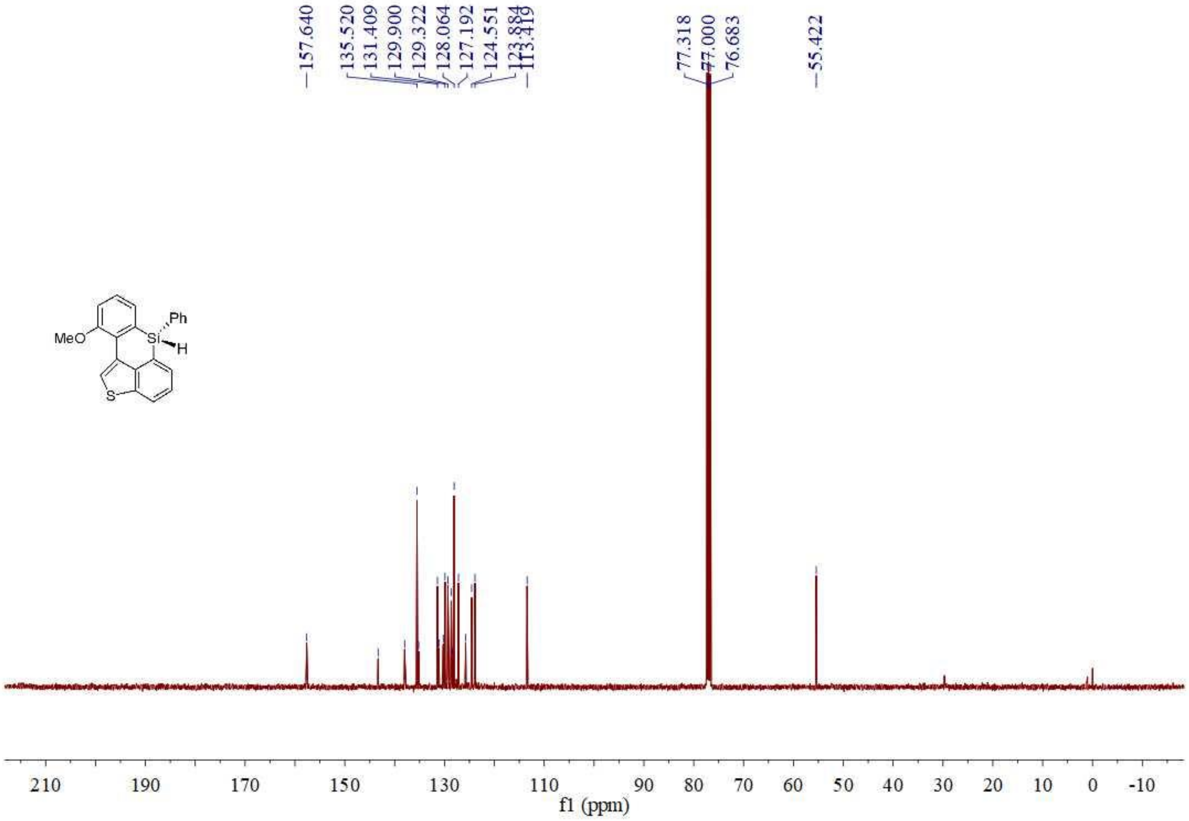
157.640
135.520
131.409
129.900
129.322
128.064
127.192
124.551
123.884
113.419
77.318
77.000
76.683
55.422
MeO
Si
Ph
H
S
210 190 170 150 130 110 90 80 70 60 50 40 30 20 10 0 -10
f1 (ppm)

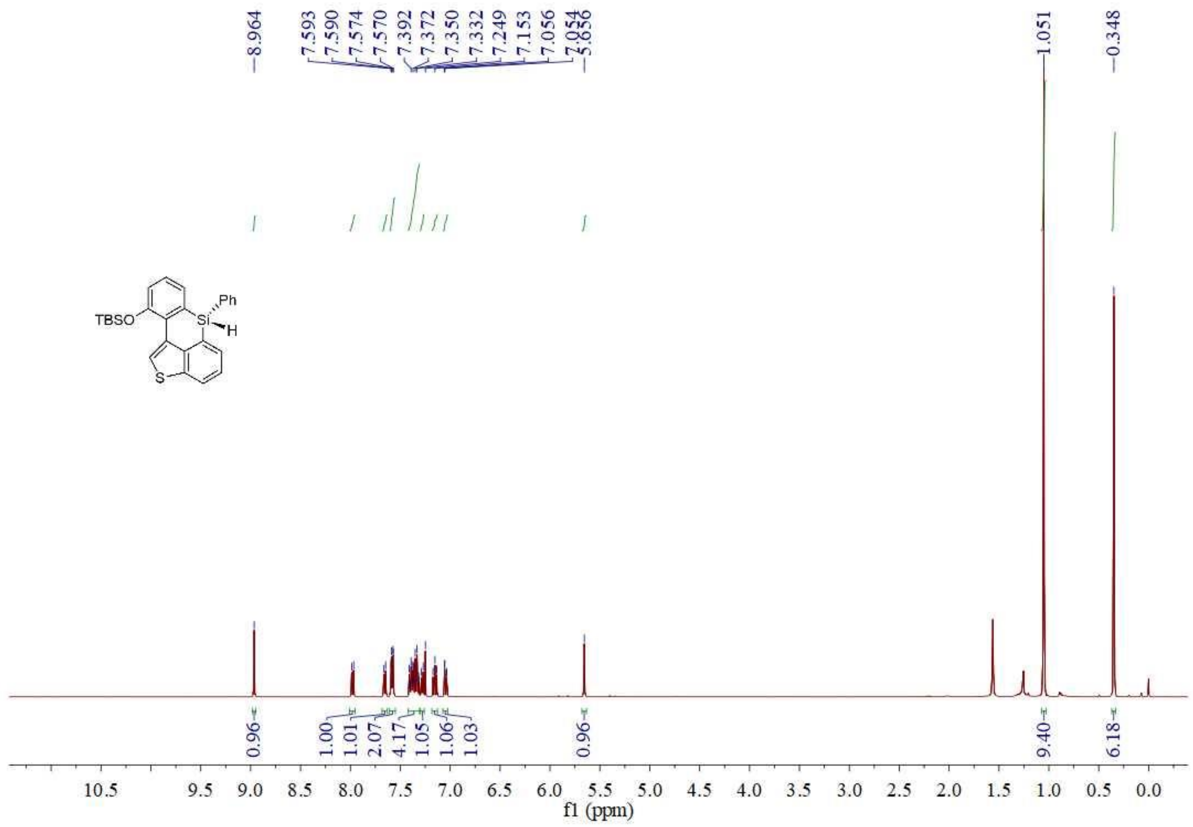

-8.964
7.593
7.590
7.574
7.570
7.392
7.372
7.350
7.332
7.249
7.153
7.056
7.054
-5.656
-1.051
-0.348
TBSO
Si
Ph
H
S
0.96
1.00
1.01
2.07
4.17
1.05
1.06
1.03
0.96
9.40
6.18
f1 (ppm)

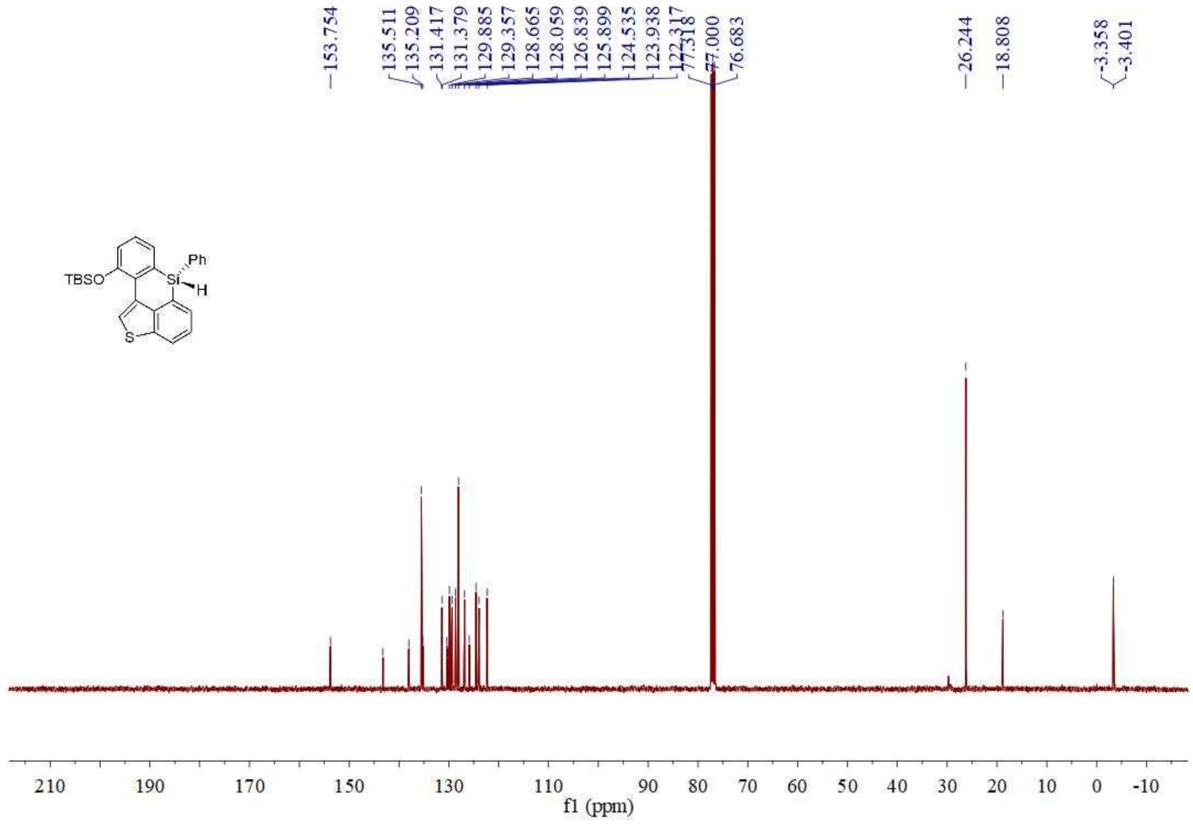

-153.754
135.511
135.209
131.417
131.379
129.885
129.357
128.665
128.059
126.839
125.899
124.535
123.938
122.317
77.318
77.000
76.683
-26.244
-18.808
-3.358
-3.401
TBSO
Si
Ph
H
S
f1 (ppm)

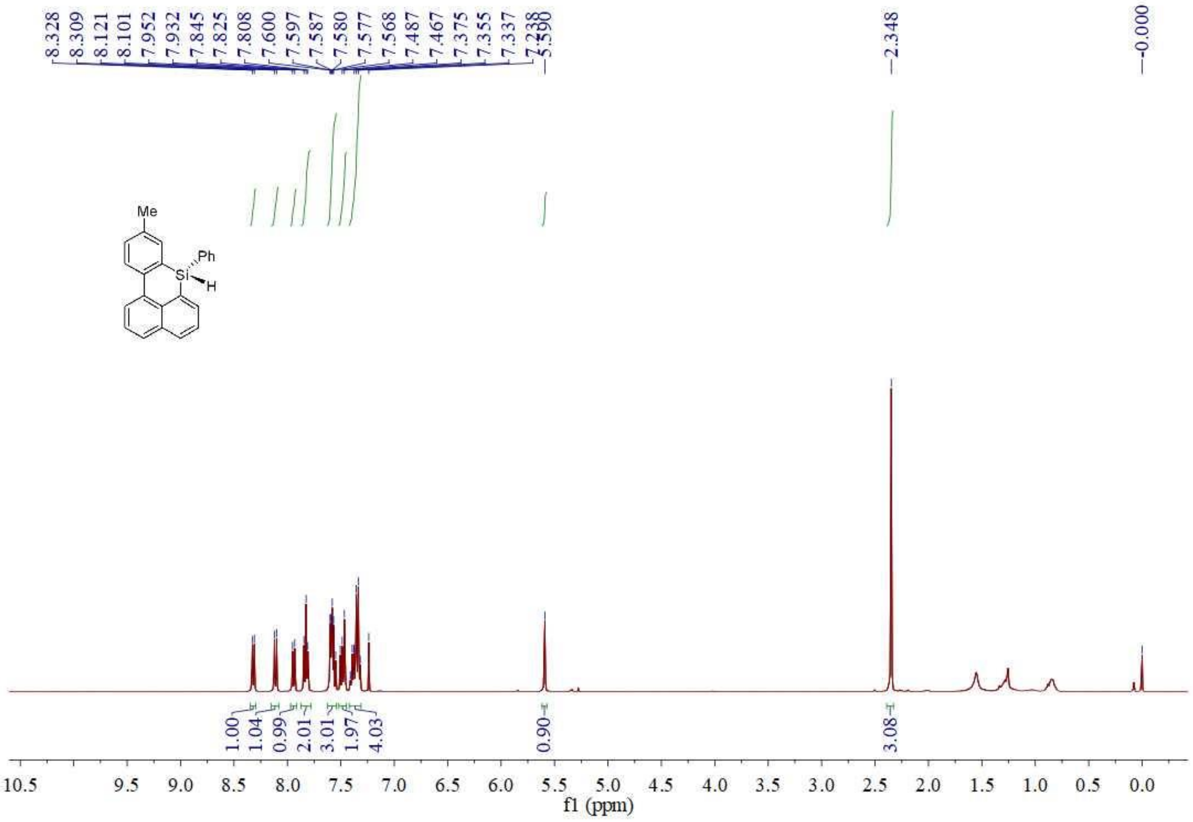
8.328
8.309
8.121
8.101
7.952
7.932
7.845
7.825
7.808
7.600
7.597
7.587
7.580
7.577
7.568
7.487
7.467
7.375
7.355
7.337
7.238
5.590
2.348
0.000
Me
Si
Ph
H
1.00
1.04
0.99
2.01
3.01
1.97
4.03
0.90
3.08
10.5 9.5 9.0 8.5 8.0 7.5 7.0 6.5 6.0 5.5 5.0 4.5 4.0 3.5 3.0 2.5 2.0 1.5 1.0 0.5 0.0
f1 (ppm)

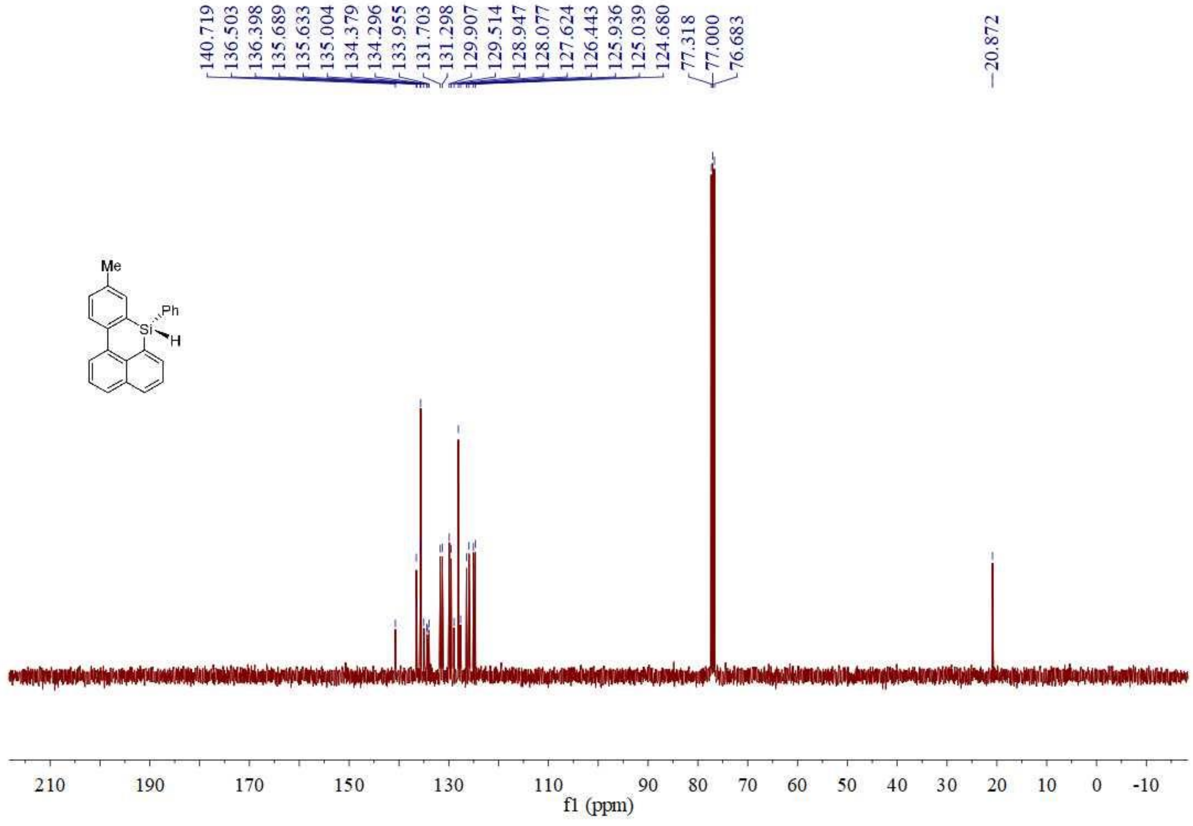
140.719
136.503
136.398
135.689
135.633
135.004
134.379
134.296
133.955
131.703
131.298
129.907
129.514
128.947
128.077
127.624
126.443
125.936
125.039
124.680
77.318
77.000
76.683
20.872
Me
Si
Ph
H
210 190 170 150 130 110 90 80 70 60 50 40 30 20 10 0 -10
f1 (ppm)

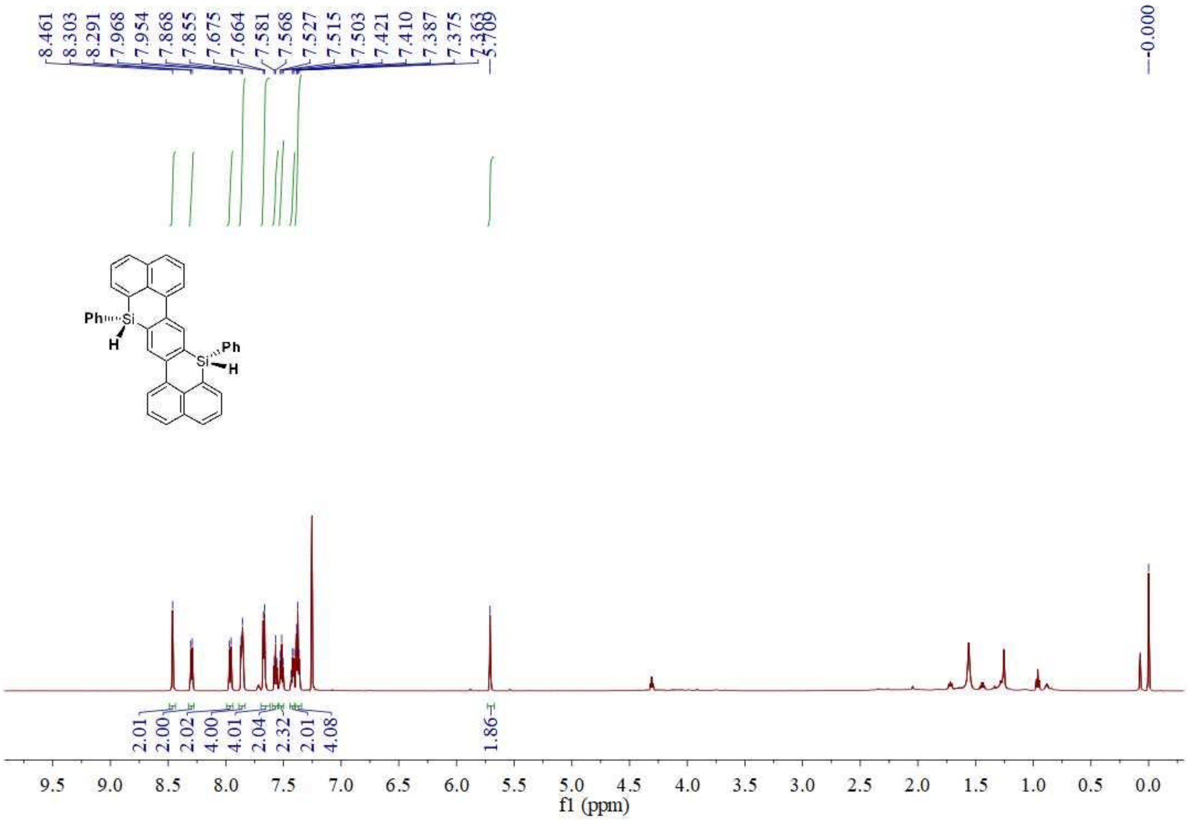
8.461
8.303
8.291
7.968
7.954
7.868
7.855
7.675
7.664
7.581
7.568
7.527
7.515
7.503
7.421
7.410
7.387
7.375
7.363
5.709
0.000
Ph
Si
H
Si
Ph
H
2.01
2.00
2.02
4.00
4.01
2.04
2.32
2.01
4.08
1.86
9.5 9.0 8.5 8.0 7.5 7.0 6.5 6.0 5.5 5.0 4.5 4.0 3.5 3.0 2.5 2.0 1.5 1.0 0.5 0.0
f1 (ppm)

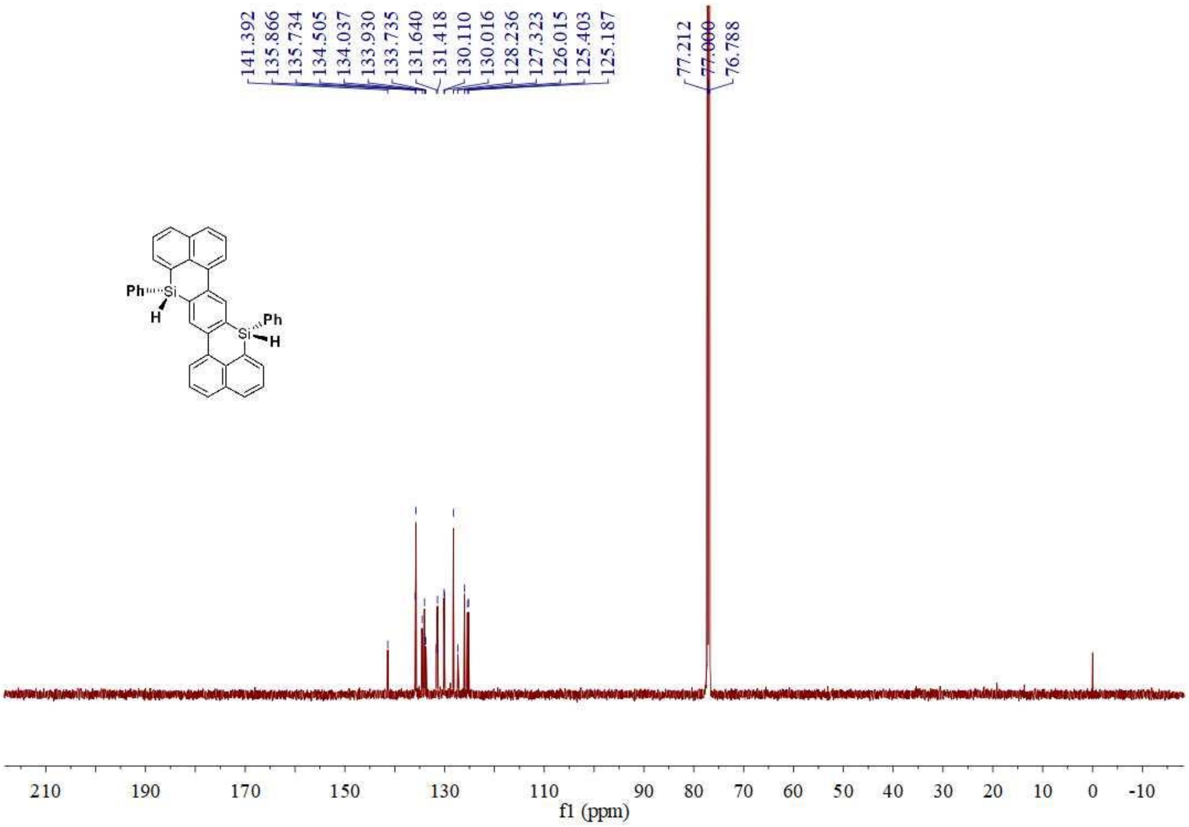
141.392
135.866
135.734
134.505
134.037
133.930
133.735
131.640
131.418
130.110
130.016
128.236
127.323
126.015
125.403
125.187
77.212
77.000
76.788
Ph
Si
H
Si
Ph
H
210 190 170 150 130 110 90 80 70 60 50 40 30 20 10 0 -10
f1 (ppm)

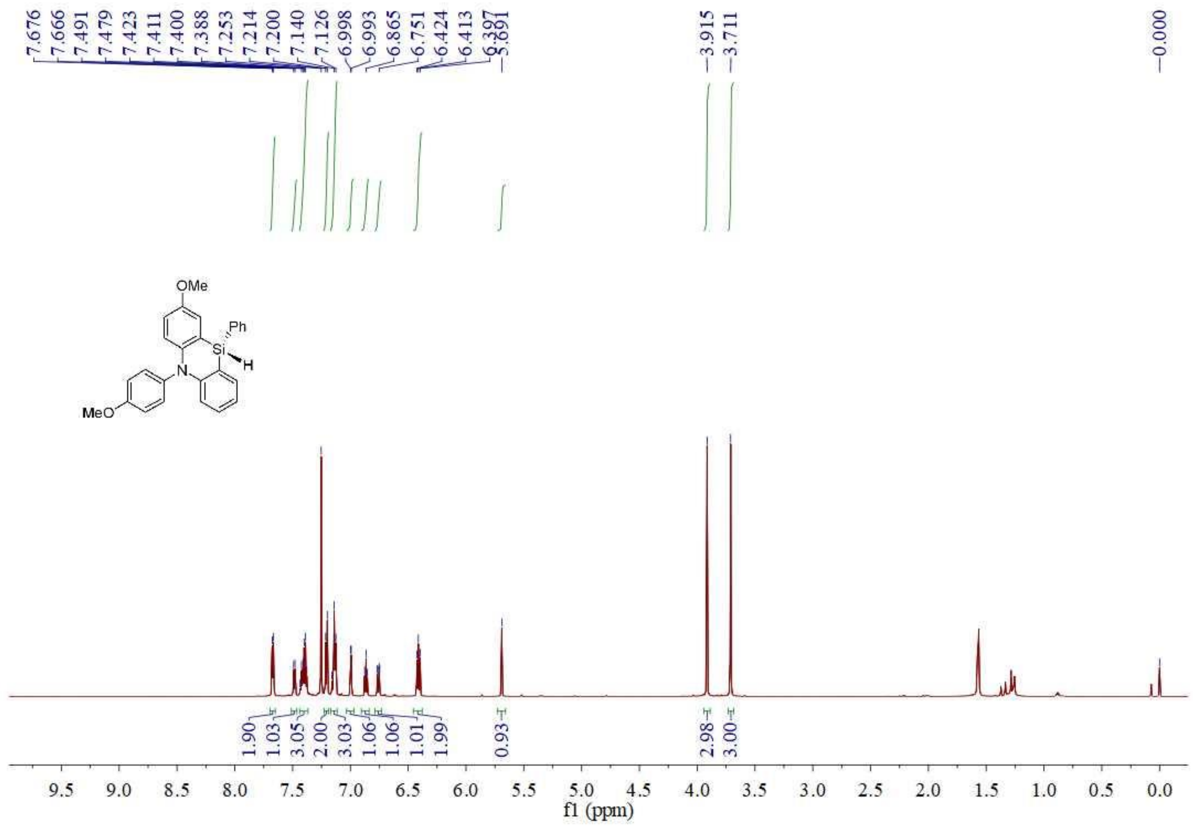

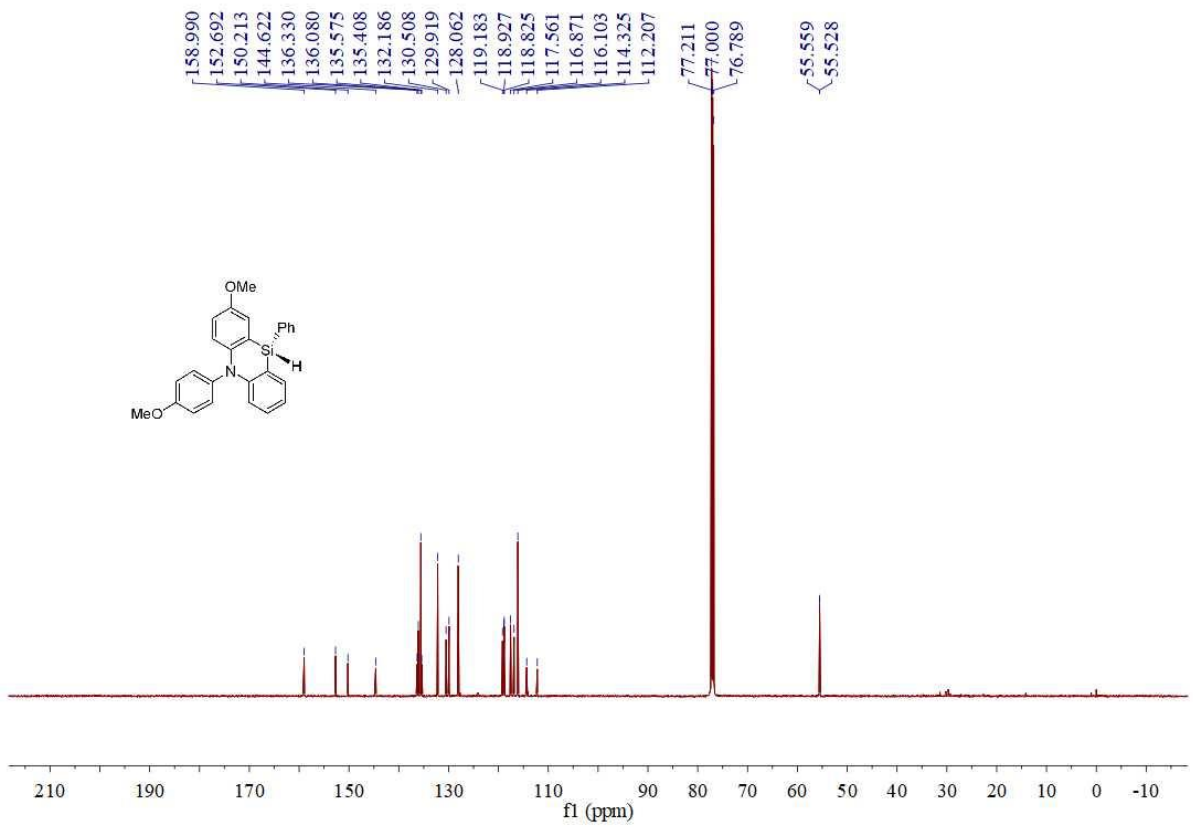

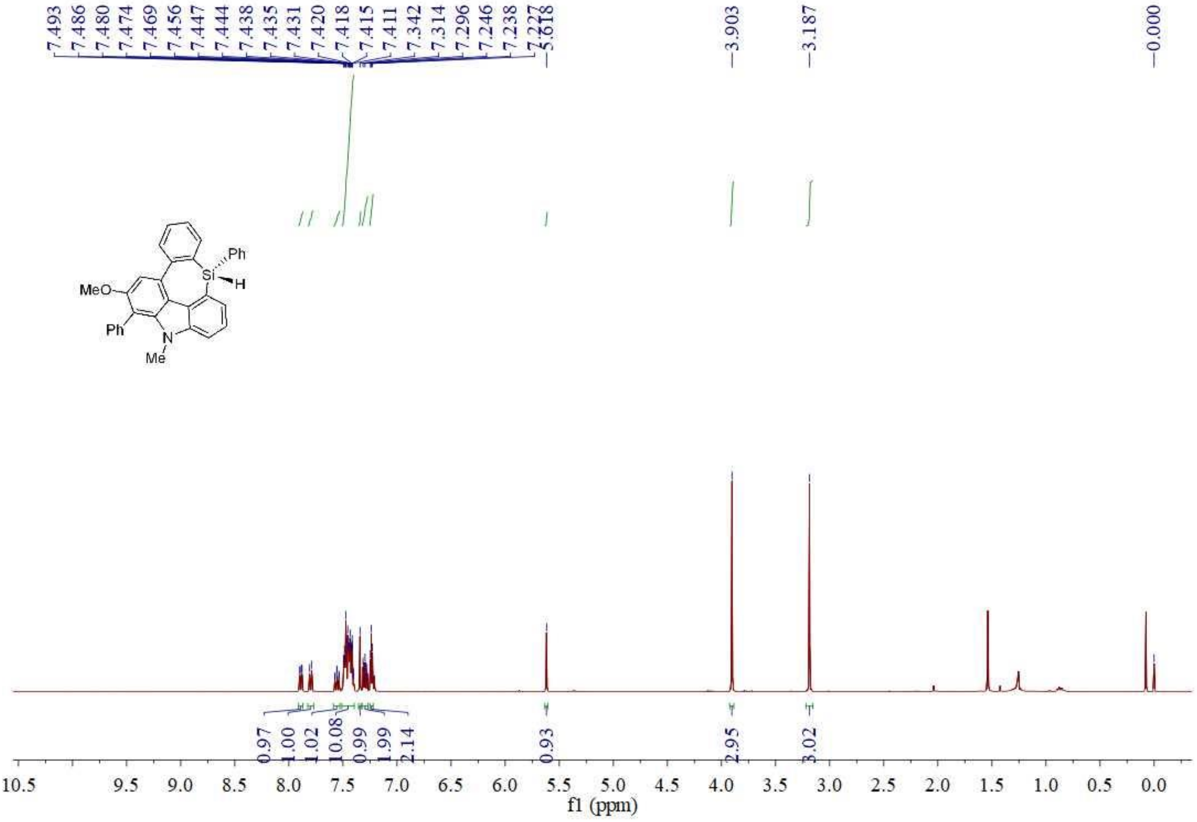

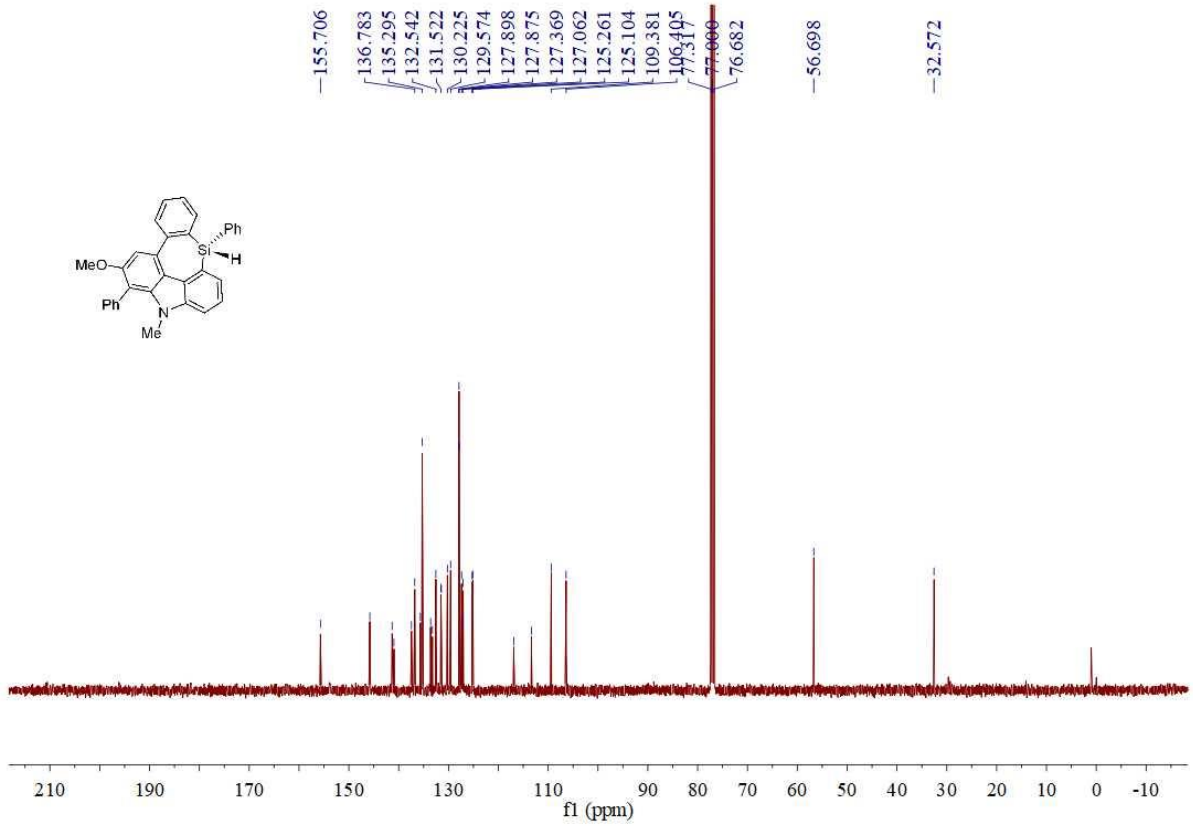

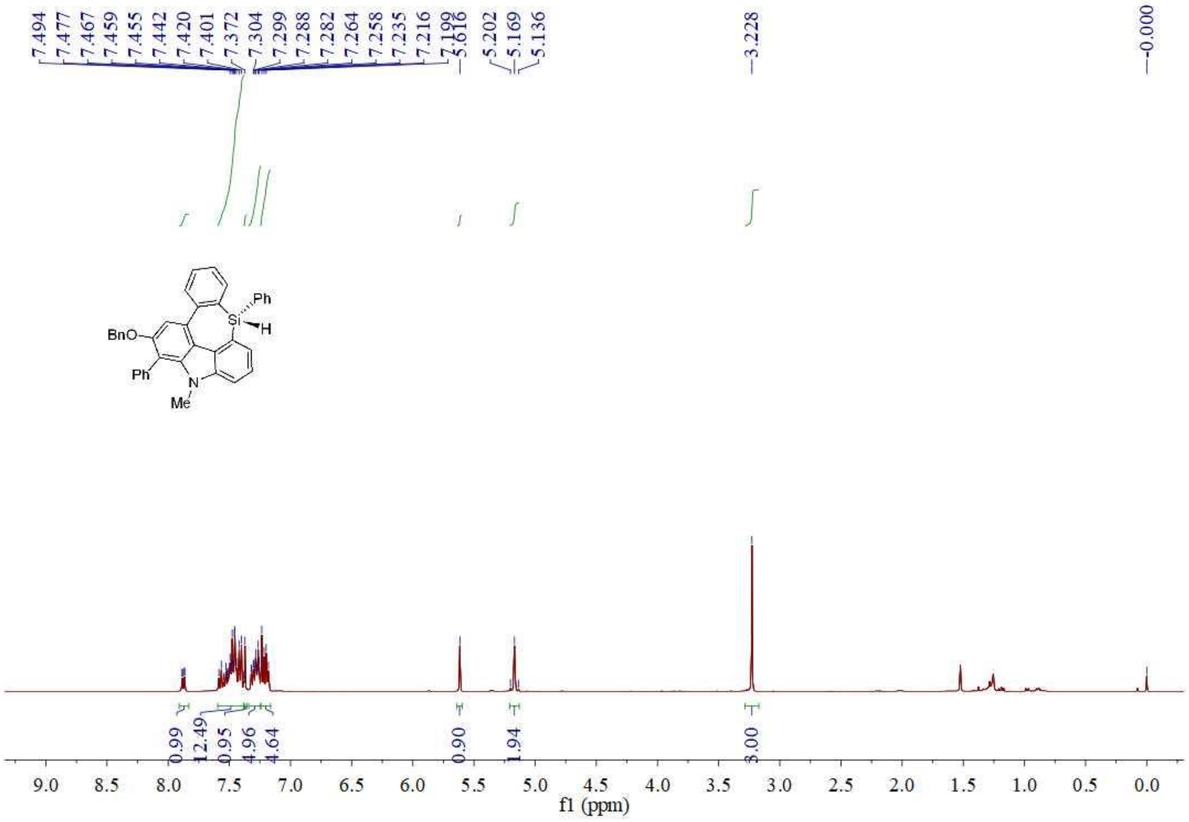

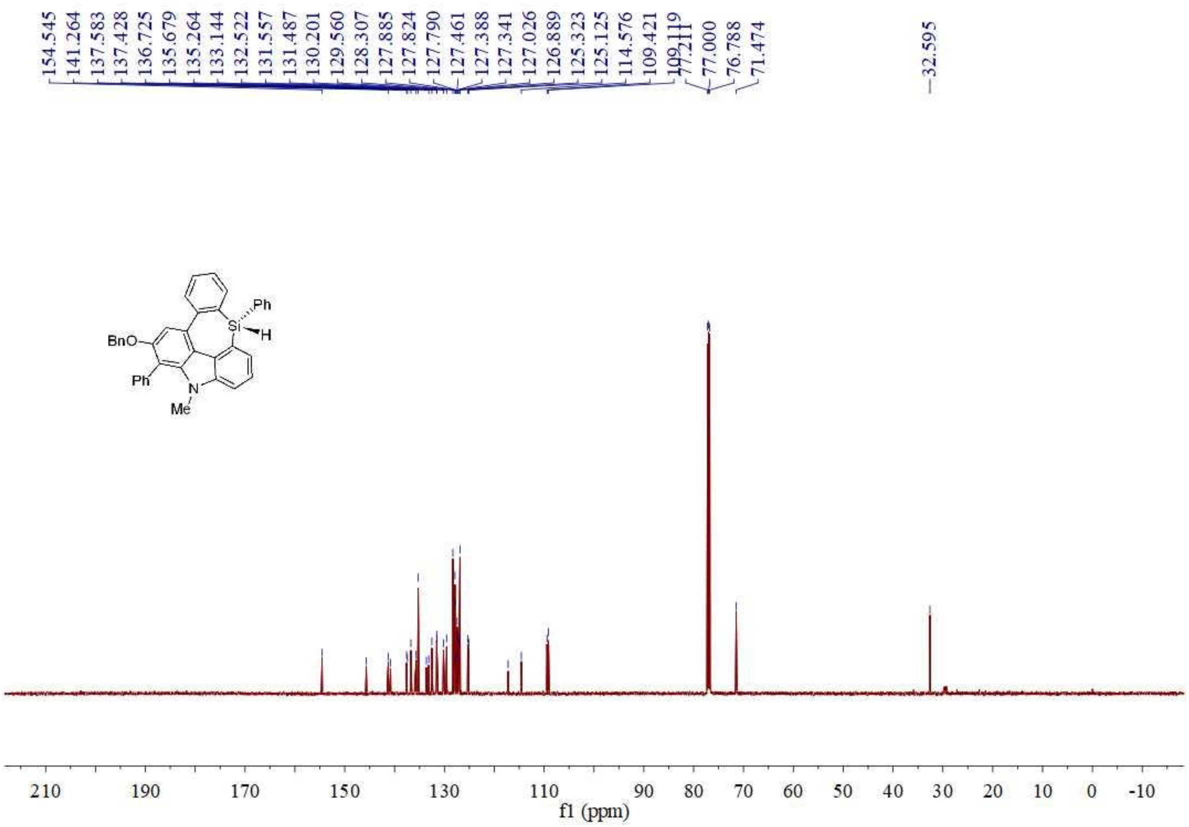

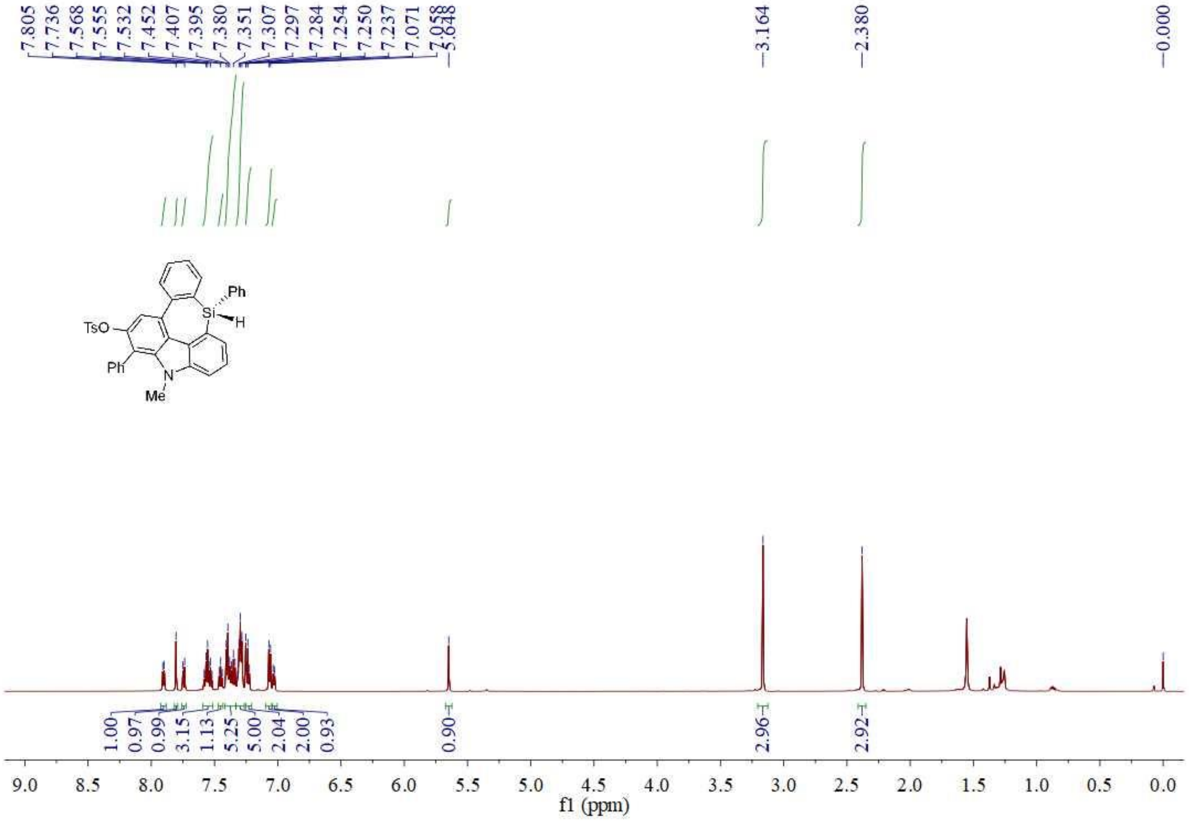
7.805
7.736
7.568
7.555
7.532
7.452
7.407
7.395
7.380
7.351
7.307
7.297
7.284
7.254
7.250
7.237
7.071
7.058
5.648
3.164
2.380
0.000
Ph
Si
H
TsO
Ph
N
Me
1.00
0.97
0.99
3.15
1.13
5.25
5.00
2.04
2.00
0.93
0.90
2.96
2.92
9.0 8.5 8.0 7.5 7.0 6.5 6.0 5.5 5.0 4.5 4.0 3.5 3.0 2.5 2.0 1.5 1.0 0.5 0.0
f1 (ppm)

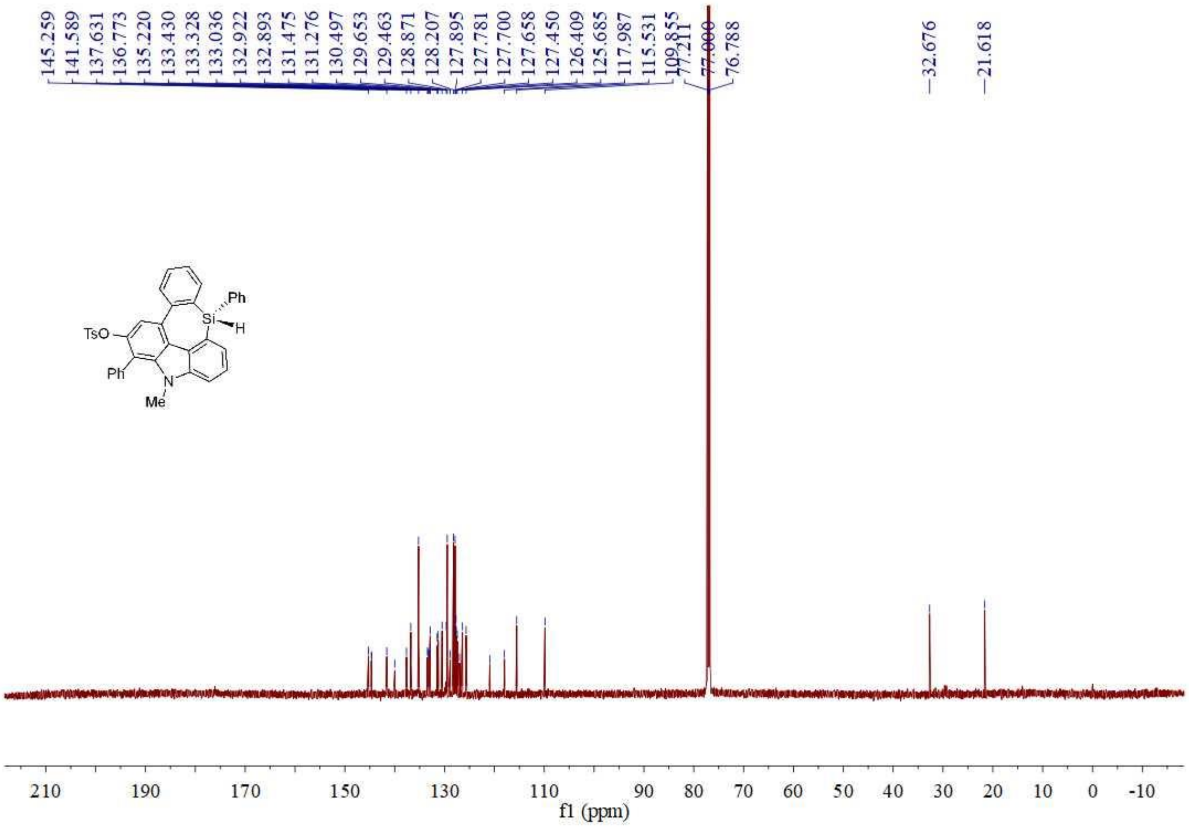
145.259
141.589
137.631
136.773
135.220
133.430
133.328
133.036
132.922
132.893
131.475
131.276
130.497
129.653
129.463
128.871
128.207
127.895
127.781
127.700
127.658
127.450
126.409
125.685
117.987
115.531
109.855
77.211
77.000
76.788
32.676
21.618
Ph
Si
H
TsO
Ph
N
Me
210 190 170 150 130 110 90 80 70 60 50 40 30 20 10 0 -10
f1 (ppm)

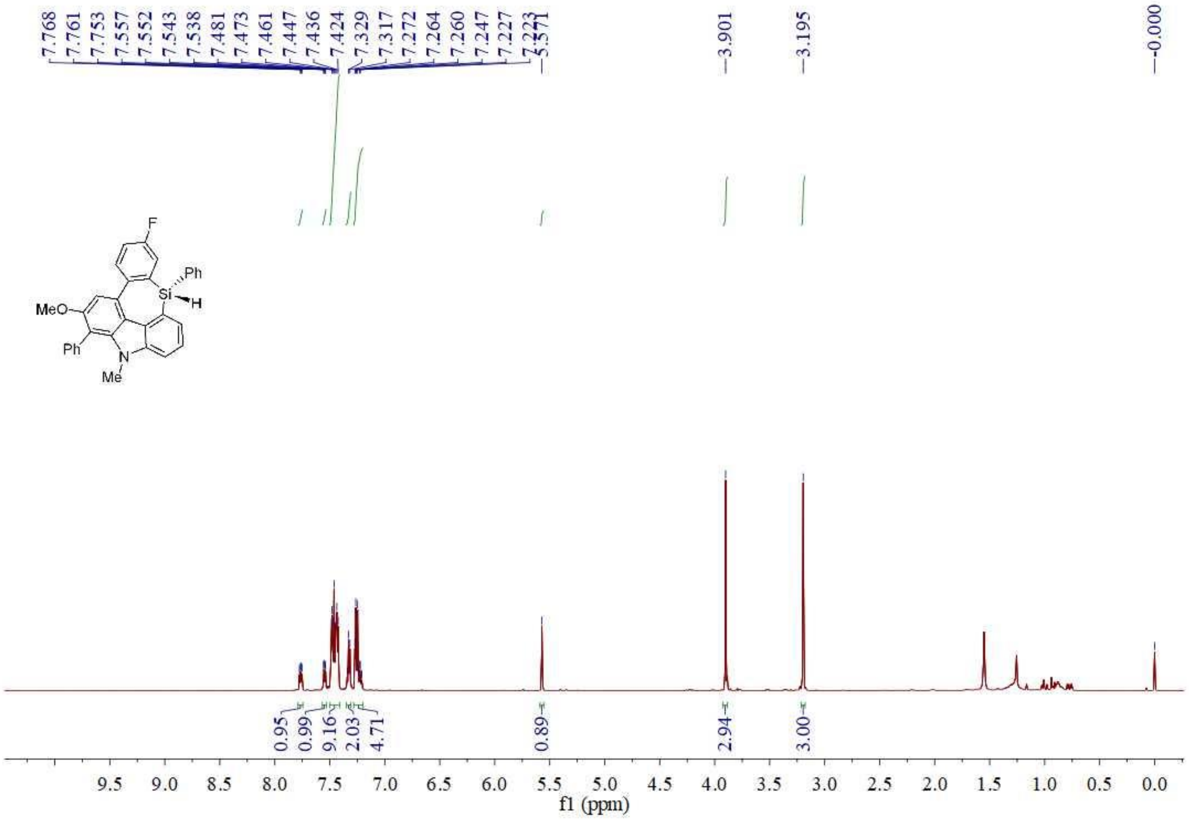
7.768
7.761
7.753
7.557
7.552
7.543
7.538
7.481
7.473
7.461
7.447
7.436
7.424
7.329
7.317
7.272
7.264
7.260
7.247
7.227
7.223
5.571
3.901
3.195
0.000
F
Ph
Si
H
MeO
Ph
N
Me
0.95
0.99
9.16
2.03
4.71
0.89
2.94
3.00
9.5 9.0 8.5 8.0 7.5 7.0 6.5 6.0 5.5 5.0 4.5 4.0 3.5 3.0 2.5 2.0 1.5 1.0 0.5 0.0
f1 (ppm)

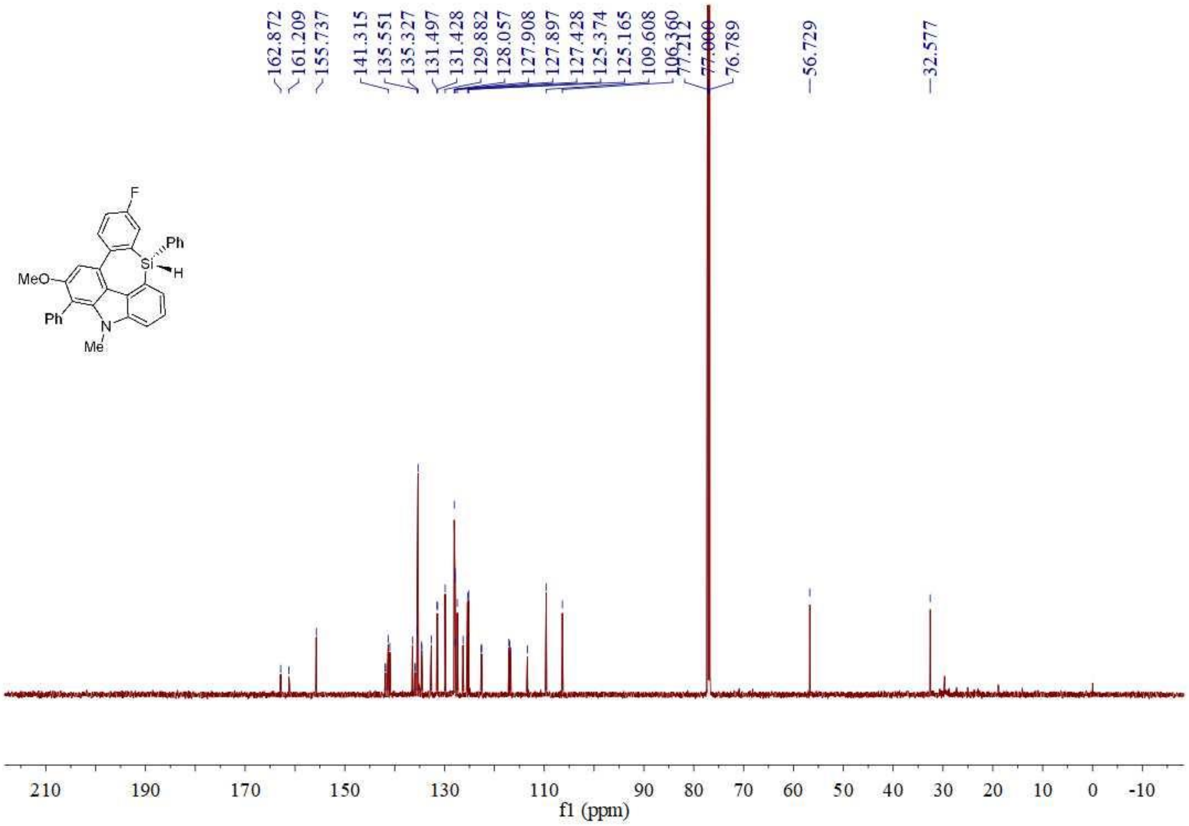
162.872
161.209
155.737
141.315
135.551
135.327
131.497
131.428
129.882
128.057
127.908
127.897
127.428
125.374
125.165
109.608
106.360
77.212
77.000
76.789
56.729
32.577
F
Ph
Si
H
MeO
Ph
N
Me
210 190 170 150 130 110 90 80 70 60 50 40 30 20 10 0 -10
f1 (ppm)

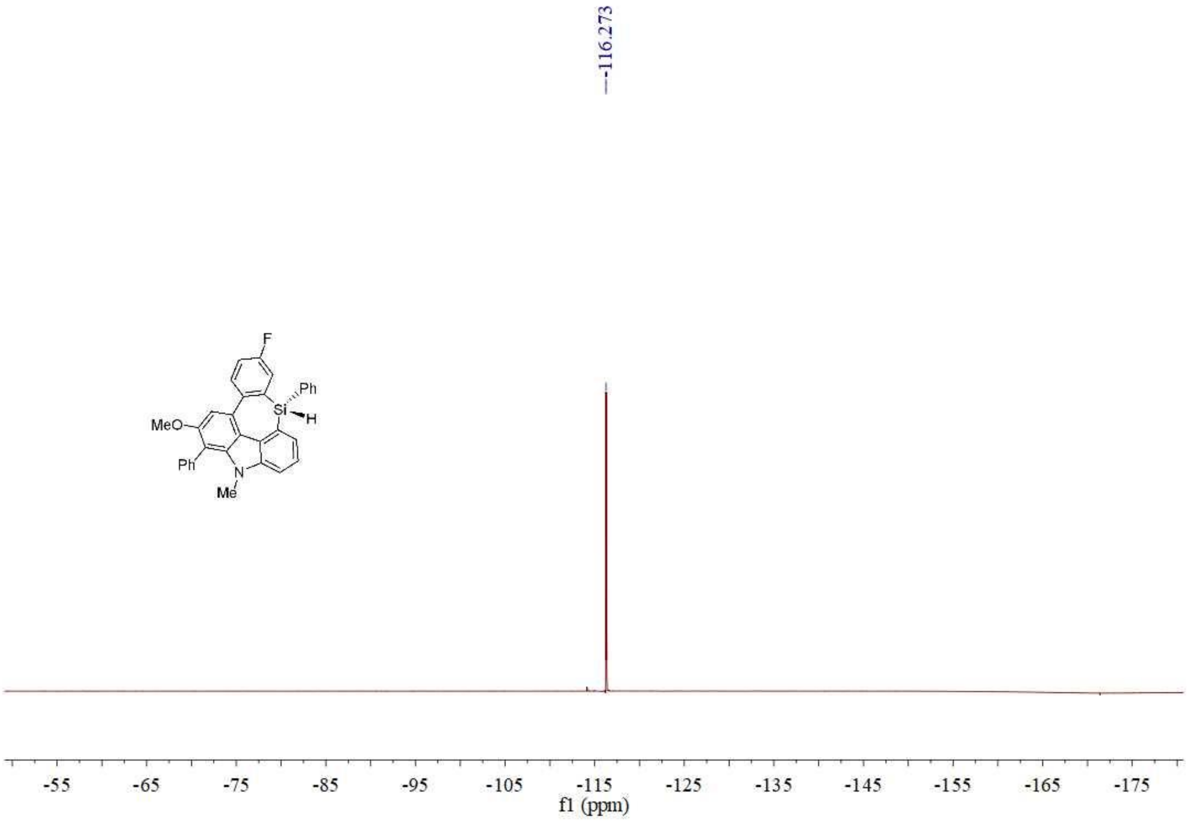

-116.273
F
Ph
Si
H
MeO
Ph
N
Me
-55
-65
-75
-85
-95
-105
-115
-125
-135
-145
-155
-165
-175
f1 (ppm)

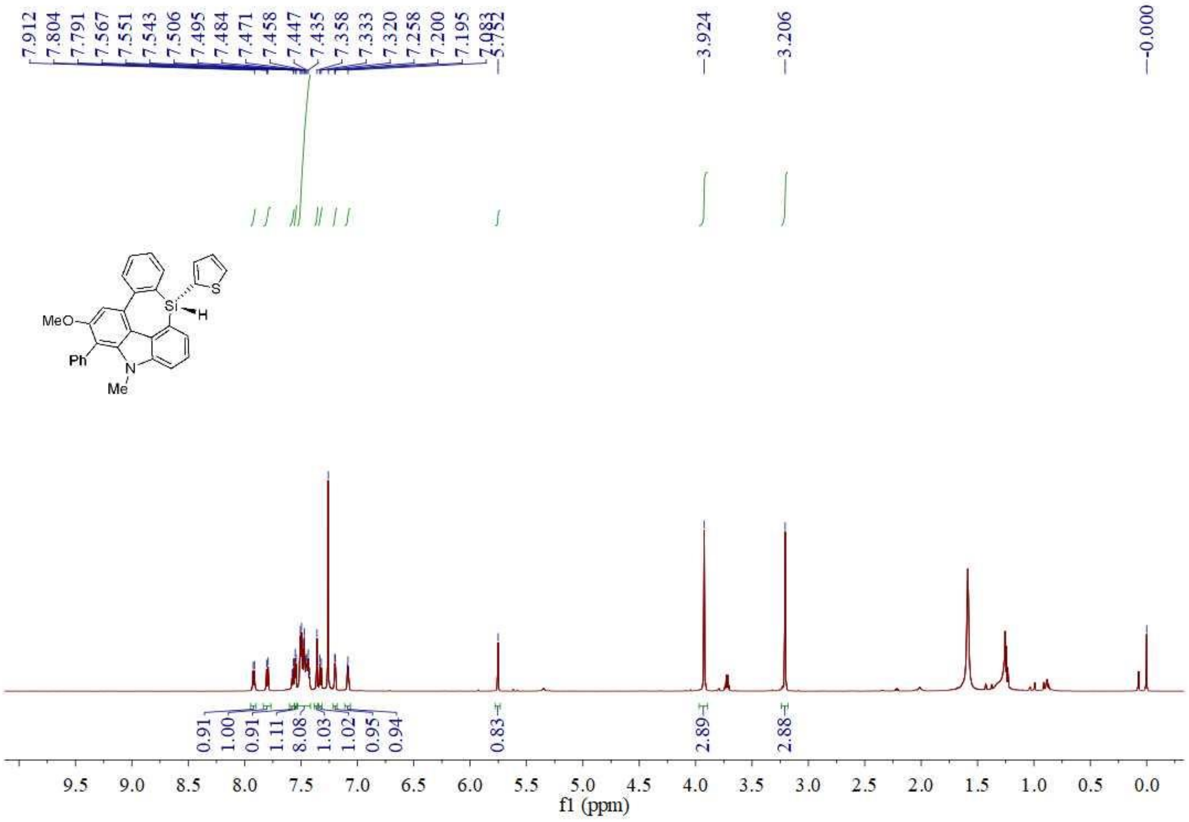
7.912
7.804
7.791
7.567
7.551
7.543
7.506
7.495
7.484
7.471
7.458
7.447
7.435
7.358
7.333
7.320
7.258
7.200
7.195
7.083
5.752
3.924
3.206
0.000
MeO
Si
H
S
Ph
N
Me
0.91
1.00
0.91
1.11
8.08
1.03
1.02
0.95
0.94
0.83
2.89
2.88
9.5 9.0 8.5 8.0 7.5 7.0 6.5 6.0 5.5 5.0 4.5 4.0 3.5 3.0 2.5 2.0 1.5 1.0 0.5 0.0
f1 (ppm)

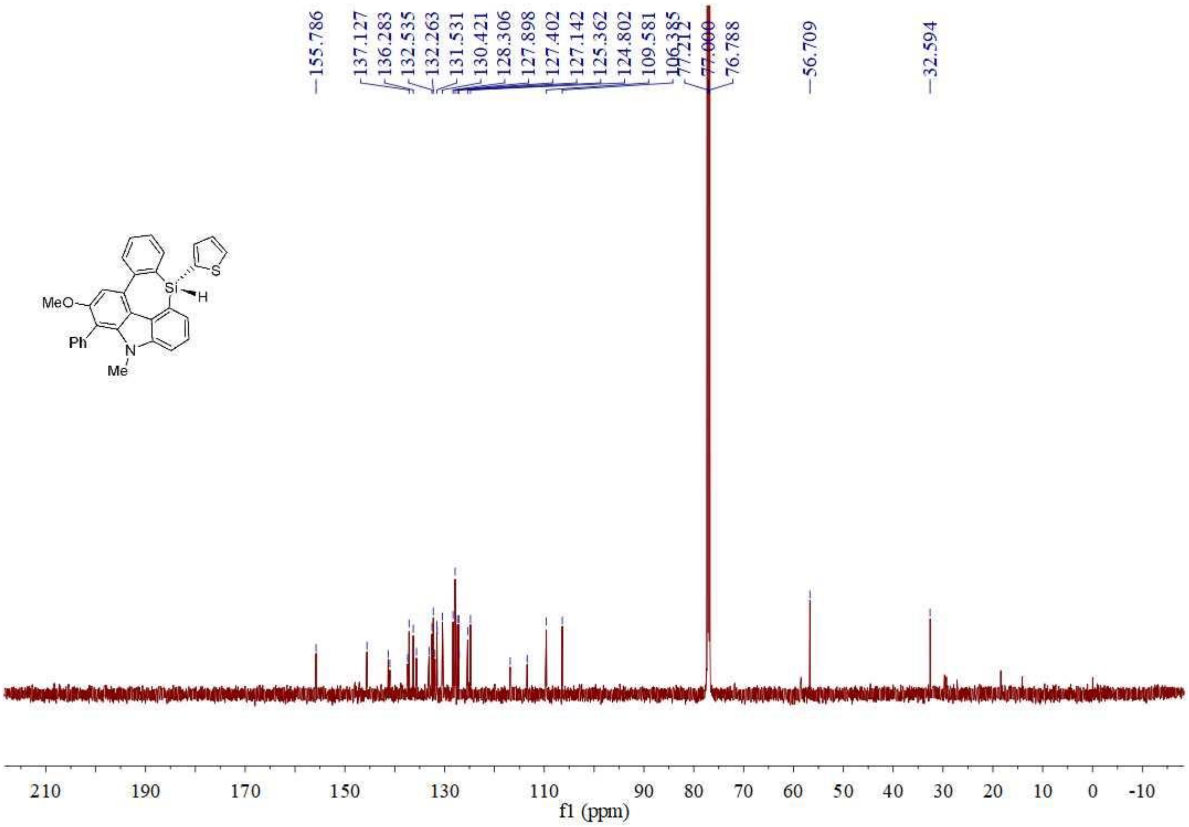
155.786
137.127
136.283
132.535
132.263
131.531
130.421
128.306
127.898
127.402
127.142
125.362
124.802
109.581
106.385
77.212
77.000
76.788
56.709
32.594
MeO
Si
H
S
Ph
N
Me
210 190 170 150 130 110 90 80 70 60 50 40 30 20 10 0 -10
f1 (ppm)

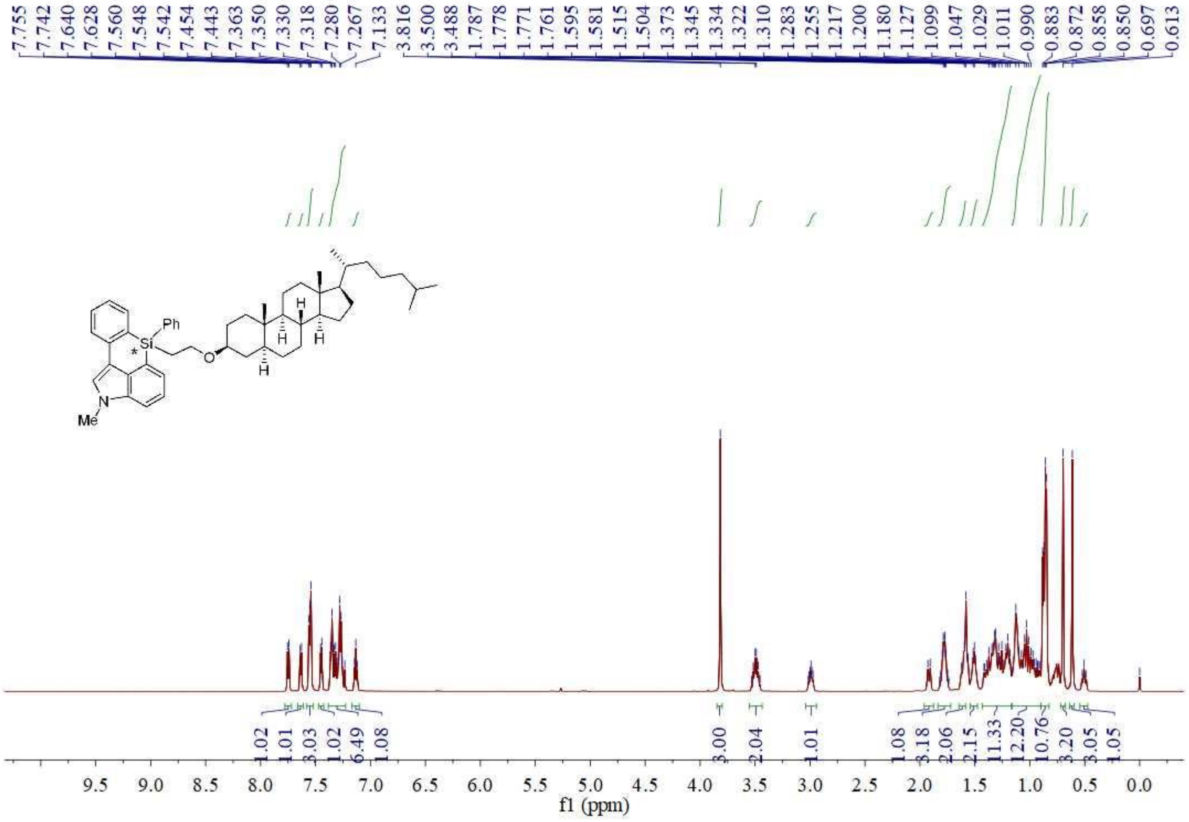
7.755 7.742 7.640 7.628 7.560 7.548 7.542 7.454 7.443 7.363 7.350 7.330 7.318 7.280 7.267 7.133 3.816 3.500 3.488 1.787 1.778 1.771 1.761 1.595 1.581 1.515 1.504 1.373 1.345 1.334 1.322 1.310 1.283 1.255 1.217 1.200 1.180 1.127 1.099 1.047 1.029 1.011 0.990 0.883 0.872 0.858 0.850 0.697 0.613
Ph
Si
*
N
Me
O
H
1.02 1.01 3.03 1.02 6.49 1.08 3.00 2.04 1.01 1.08 3.18 2.06 2.15 11.33 12.20 10.76 3.20 3.05 1.05
9.5 9.0 8.5 8.0 7.5 7.0 6.5 6.0 5.5 5.0 4.5 4.0 3.5 3.0 2.5 2.0 1.5 1.0 0.5 0.0
f1 (ppm)

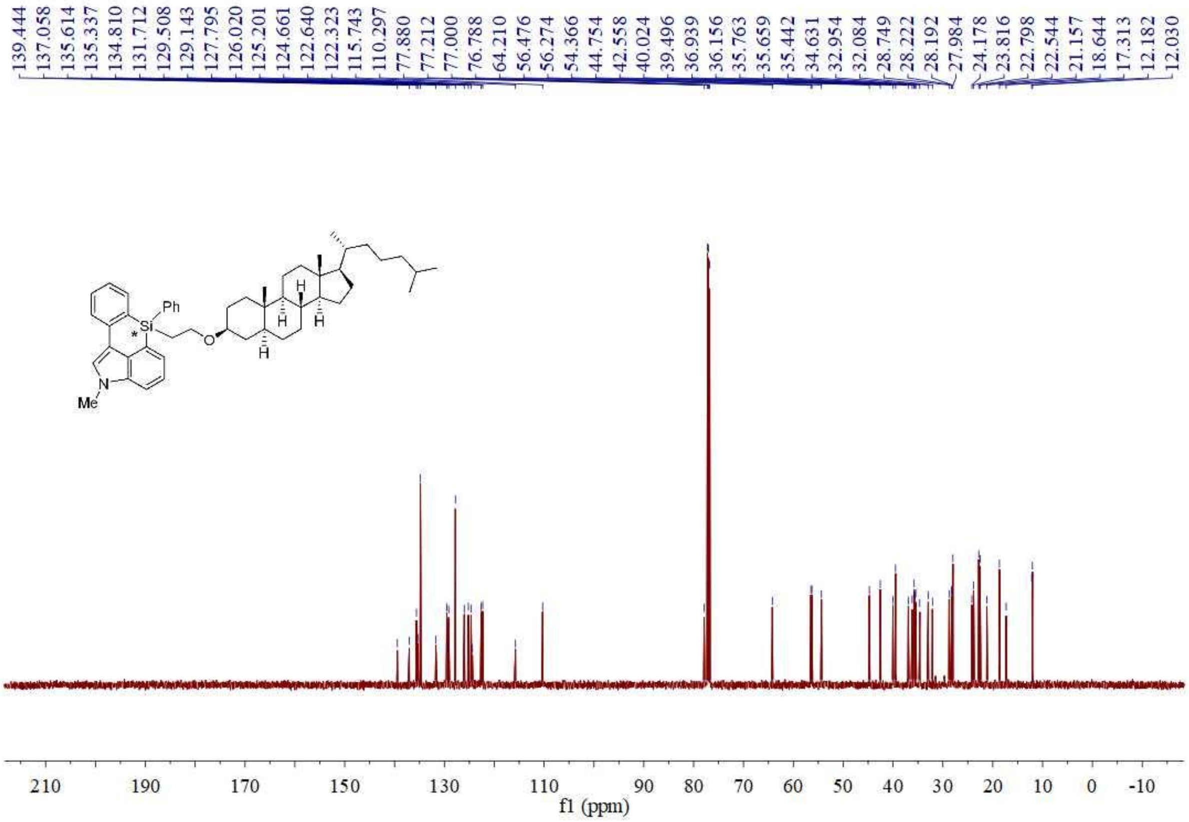
139.444 137.058 135.614 135.337 134.810 131.712 129.508 129.143 127.795 126.020 125.201 124.661 122.640 122.323 115.743 110.297 77.880 77.212 77.000 76.788 64.210 56.476 56.274 54.366 44.754 42.558 40.024 39.496 36.939 36.156 35.763 35.659 35.442 34.631 32.954 32.084 28.749 28.222 28.192 27.984 24.178 23.816 22.798 22.544 21.157 18.644 17.313 12.182 12.030
Ph
Si
*
N
Me
O
H
210 190 170 150 130 110 90 80 70 60 50 40 30 20 10 0 -10
f1 (ppm)

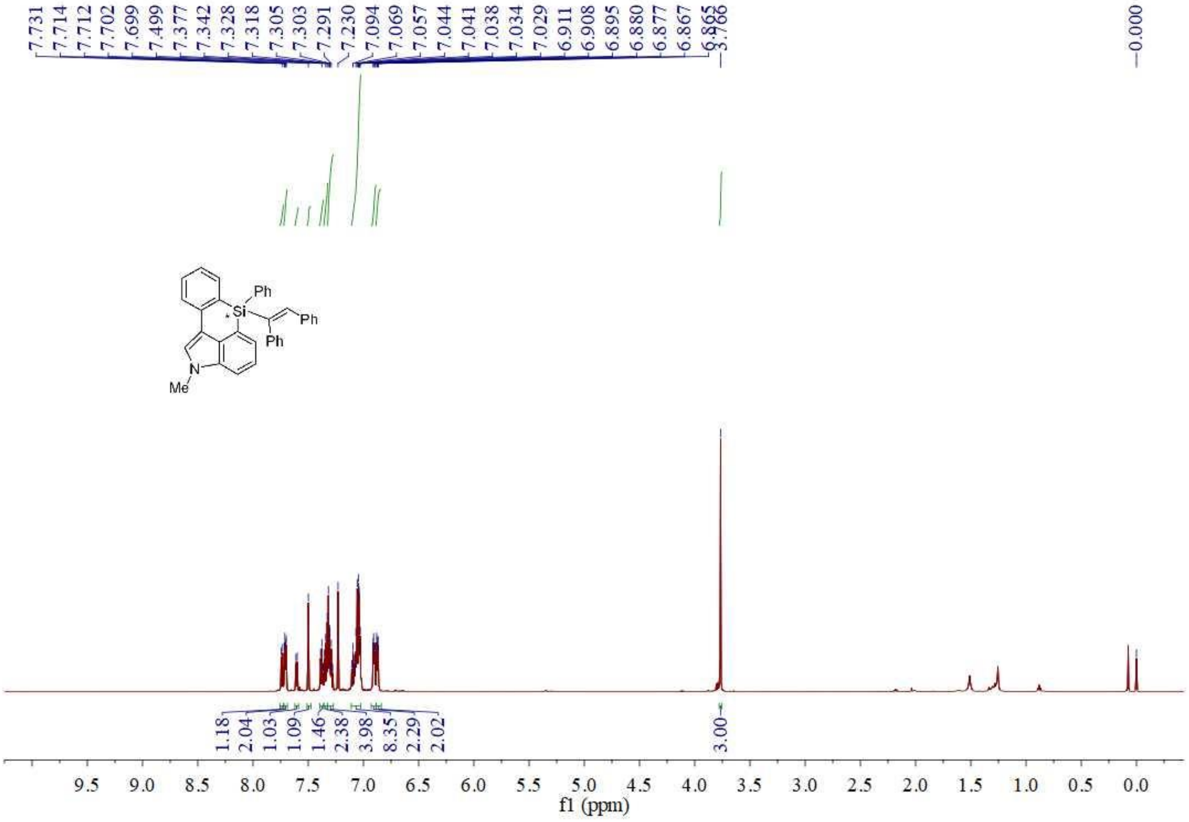
7.731 7.714 7.712 7.702 7.699 7.499 7.377 7.342 7.328 7.318 7.305 7.303 7.291 7.230 7.094 7.069 7.057 7.044 7.041 7.038 7.034 7.029 6.911 6.908 6.895 6.880 6.877 6.867 6.865 3.766
-0.000
Ph
Si
Ph
Ph
N
Me
1.18 2.04 1.03 1.09 1.46 2.38 3.98 8.35 2.29 2.02
3.00
9.5 9.0 8.5 8.0 7.5 7.0 6.5 6.0 5.5 5.0 4.5 4.0 3.5 3.0 2.5 2.0 1.5 1.0 0.5 0.0
f1 (ppm)

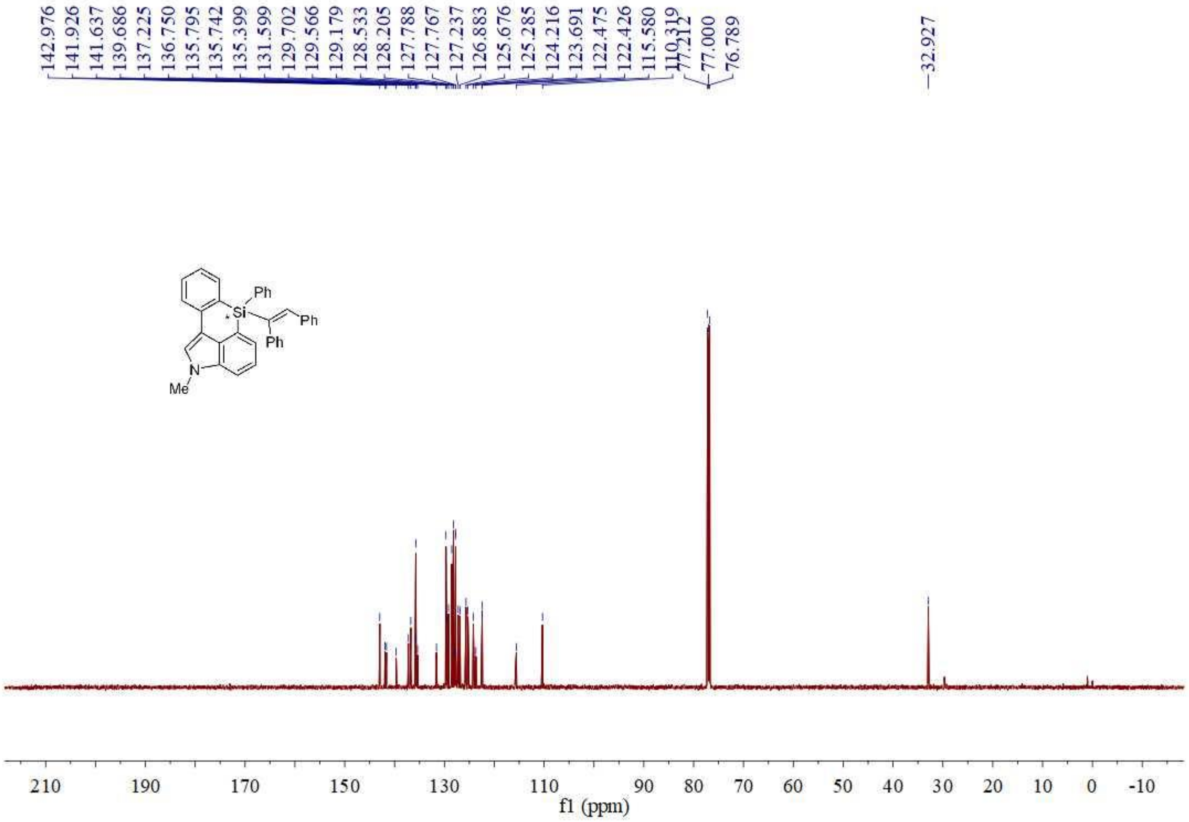
142.976 141.926 141.637 139.686 137.225 136.750 135.795 135.742 135.399 131.599 129.702 129.566 129.179 128.533 128.205 127.788 127.767 127.237 126.883 125.676 125.285 124.216 123.691 122.475 122.426 115.580 110.319 77.212 77.000 76.789
-32.927
Ph
Si
Ph
Ph
N
Me
210 190 170 150 130 110 90 80 70 60 50 40 30 20 10 0 -10
f1 (ppm)

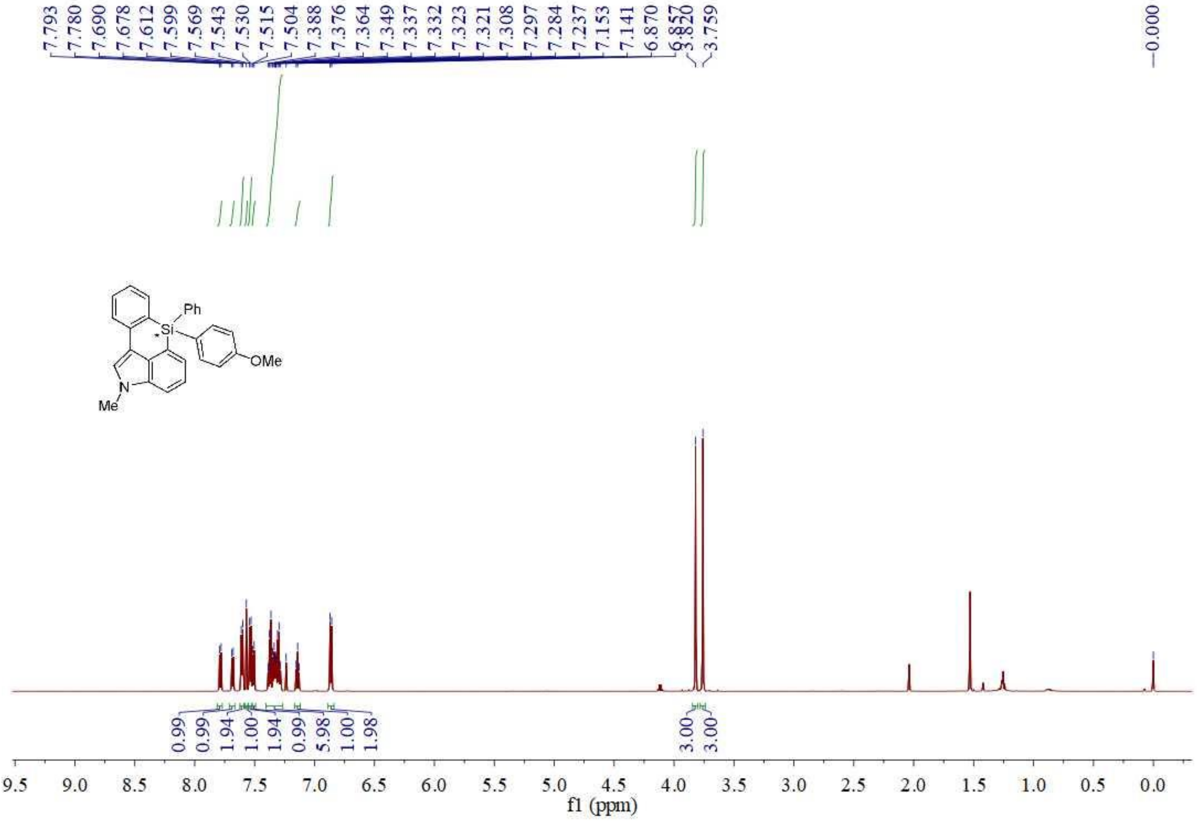

7.793
7.780
7.690
7.678
7.612
7.599
7.569
7.543
7.530
7.515
7.504
7.388
7.376
7.364
7.349
7.337
7.332
7.323
7.321
7.308
7.297
7.284
7.237
7.153
7.141
6.870
6.857
3.820
3.759
0.000
Ph
Si
OMe
N
Me
0.99
0.99
1.94
1.00
1.94
0.99
5.98
1.00
1.98
3.00
3.00
9.5 9.0 8.5 8.0 7.5 7.0 6.5 6.0 5.5 5.0 4.5 4.0 3.5 3.0 2.5 2.0 1.5 1.0 0.5 0.0
f1 (ppm)

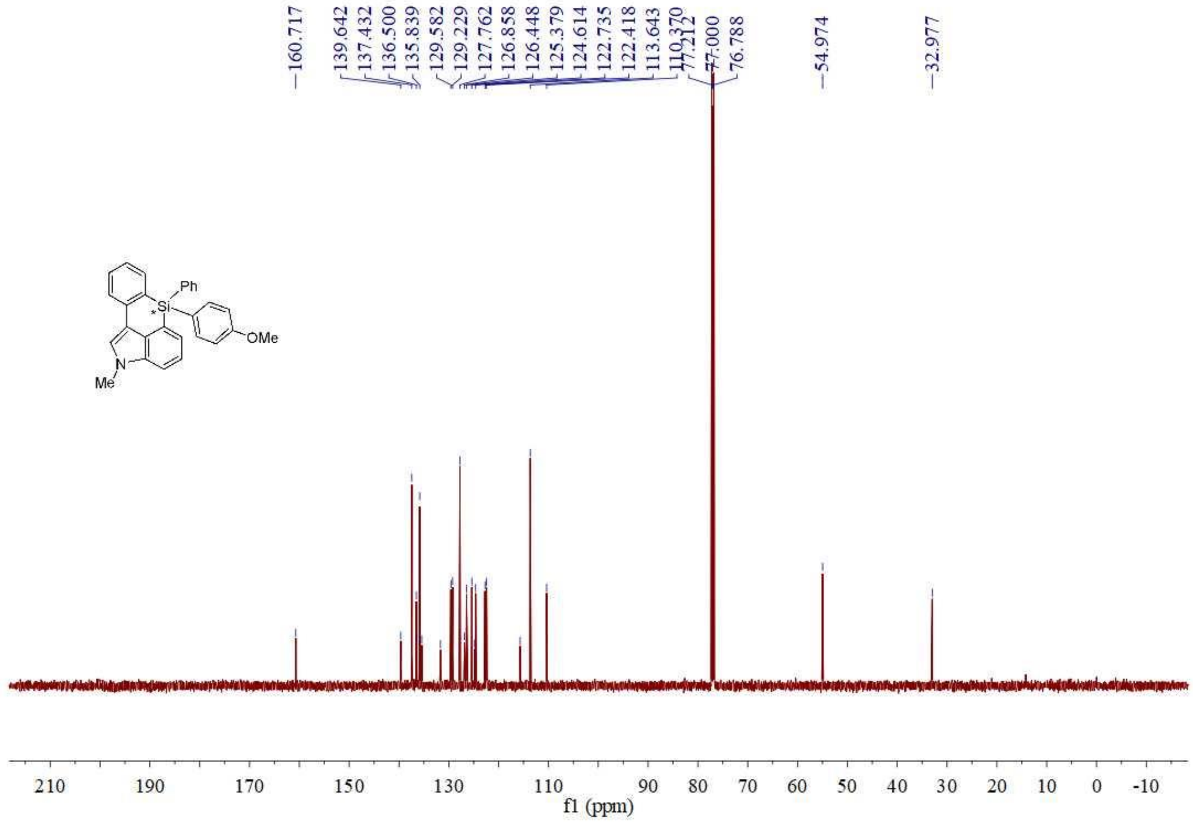

160.717
139.642
137.432
136.500
135.839
129.582
129.229
127.762
126.858
126.448
125.379
124.614
122.735
122.418
113.643
110.370
77.212
77.000
76.788
54.974
32.977
Ph
Si
OMe
N
Me
210 190 170 150 130 110 90 80 70 60 50 40 30 20 10 0 -10
f1 (ppm)

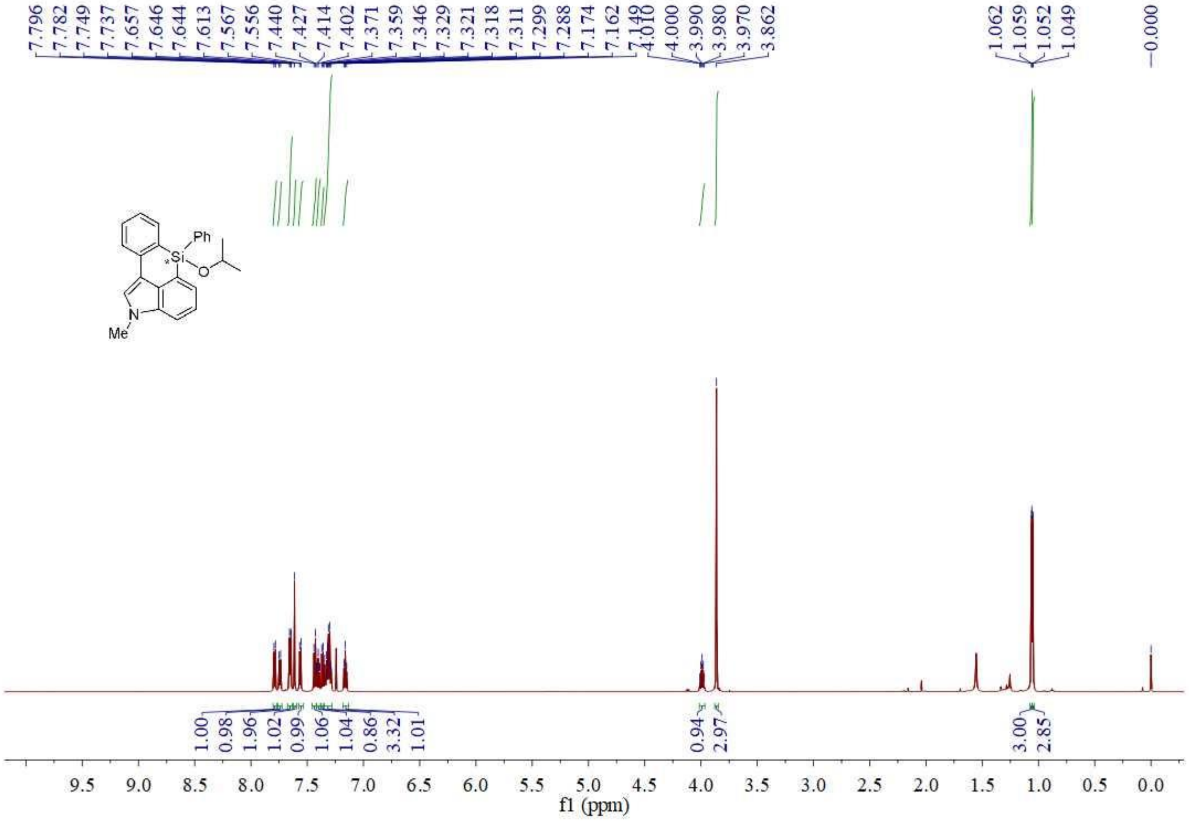

7.796 7.782 7.749 7.737 7.657 7.646 7.644 7.613 7.567 7.556 7.440 7.427 7.414 7.402 7.371 7.359 7.346 7.329 7.321 7.318 7.311 7.299 7.288 7.174 7.162 7.149 4.010 4.000 3.990 3.980 3.970 3.862
1.062 1.059 1.052 1.049
0.000
Ph
Si
*
O
N
Me
1.00 0.98 1.96 1.02 0.99 1.06 1.04 0.86 3.32 1.01
0.94 2.97
3.00 2.85
9.5 9.0 8.5 8.0 7.5 7.0 6.5 6.0 5.5 5.0 4.5 4.0 3.5 3.0 2.5 2.0 1.5 1.0 0.5 0.0
f1 (ppm)

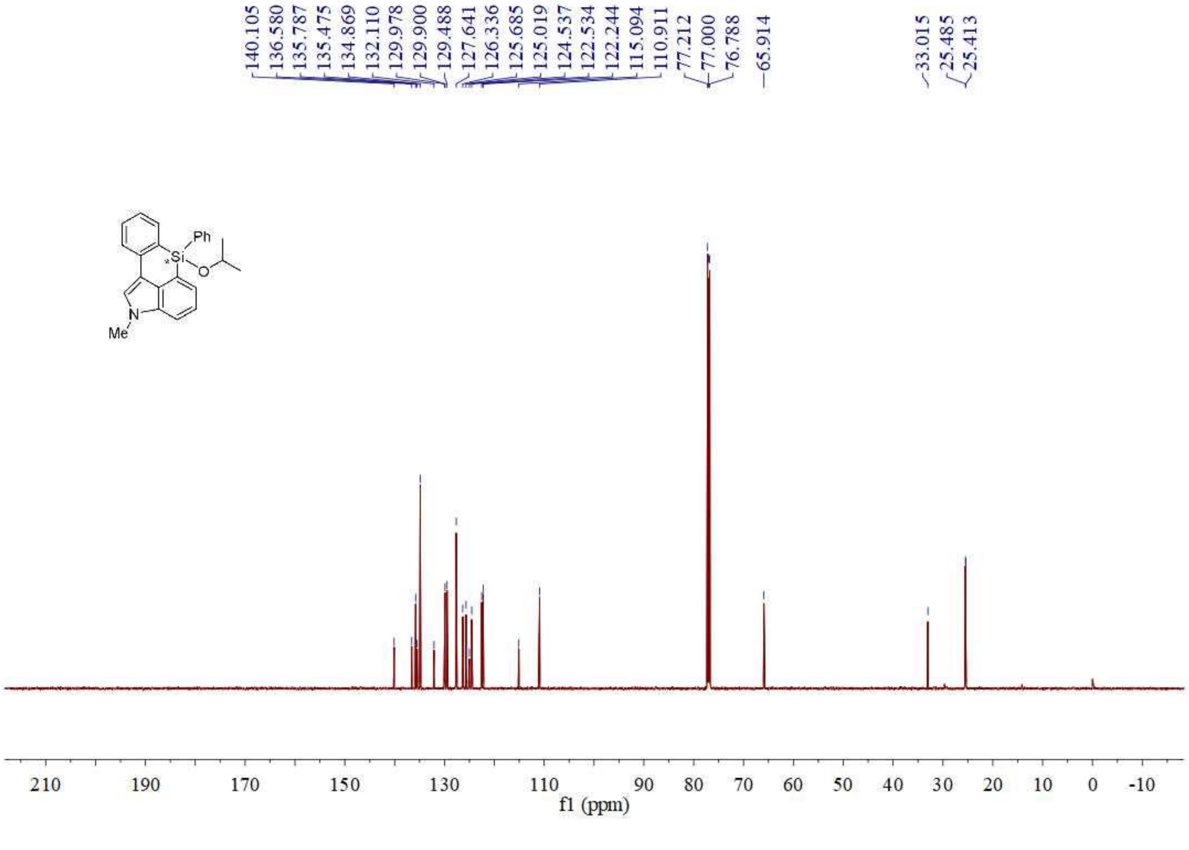

140.105 136.580 135.787 135.475 134.869 132.110 129.978 129.900 129.488 127.641 126.336 125.685 125.019 124.537 122.534 122.244 115.094 110.911
77.212 77.000 76.788
65.914
33.015
25.485 25.413
Ph
Si
*
O
N
Me
210 190 170 150 130 110 90 80 70 60 50 40 30 20 10 0 -10
f1 (ppm)

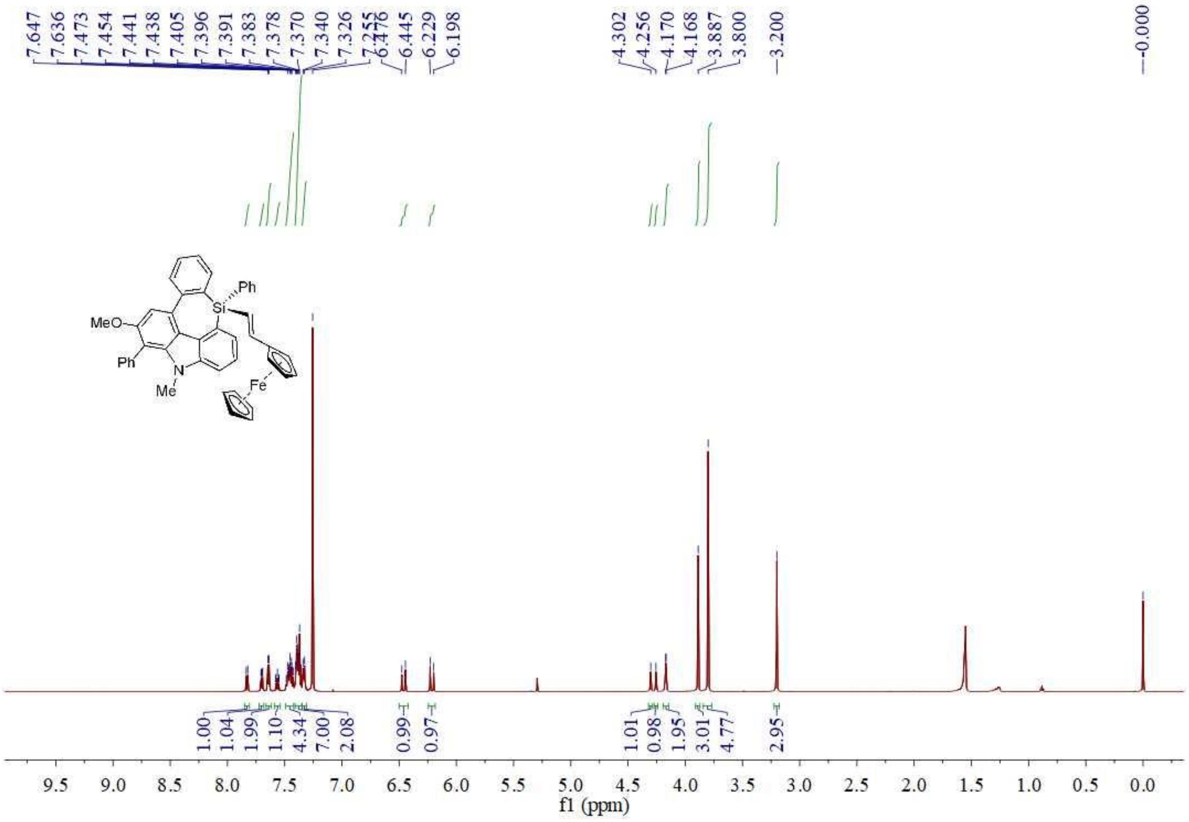
7.647
7.636
7.473
7.454
7.441
7.438
7.405
7.396
7.391
7.383
7.378
7.370
7.340
7.326
7.255
6.476
6.445
6.229
6.198
4.302
4.256
4.170
4.168
3.887
3.800
3.200
0.000
MeO
Ph
Si
Ph
N
Me
Fe
1.00
1.04
1.99
1.10
4.34
7.00
2.08
0.99
0.97
1.01
0.98
1.95
3.01
4.77
2.95
9.5 9.0 8.5 8.0 7.5 7.0 6.5 6.0 5.5 5.0 4.5 4.0 3.5 3.0 2.5 2.0 1.5 1.0 0.5 0.0
f1 (ppm)

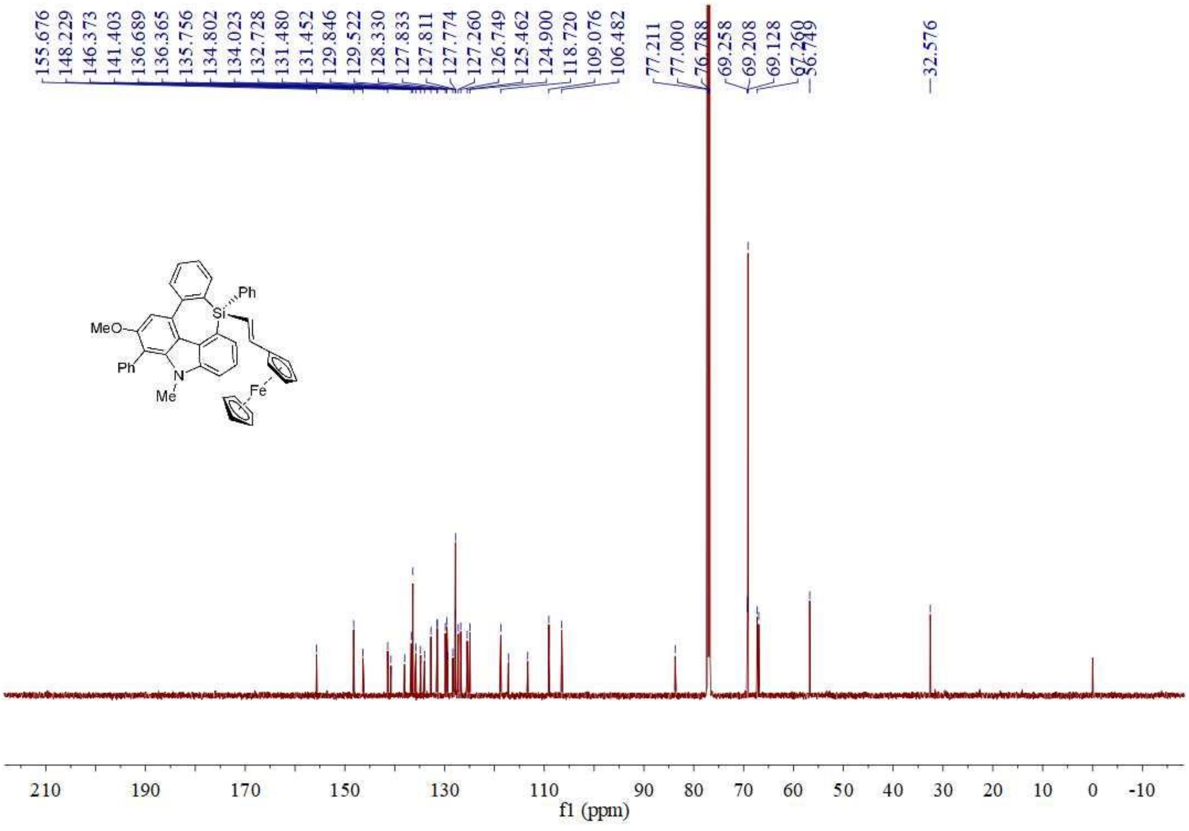
155.676
148.229
146.373
141.403
136.689
136.365
135.756
134.802
134.023
132.728
131.480
131.452
129.846
129.522
128.330
127.833
127.811
127.774
127.260
126.749
125.462
124.900
118.720
109.076
106.482
77.211
77.000
76.788
69.258
69.208
69.128
67.260
56.749
32.576
MeO
Ph
Si
Ph
N
Me
Fe
210 190 170 150 130 110 90 80 70 60 50 40 30 20 10 0 -10
f1 (ppm)

X. Chiral HPLC Spectra

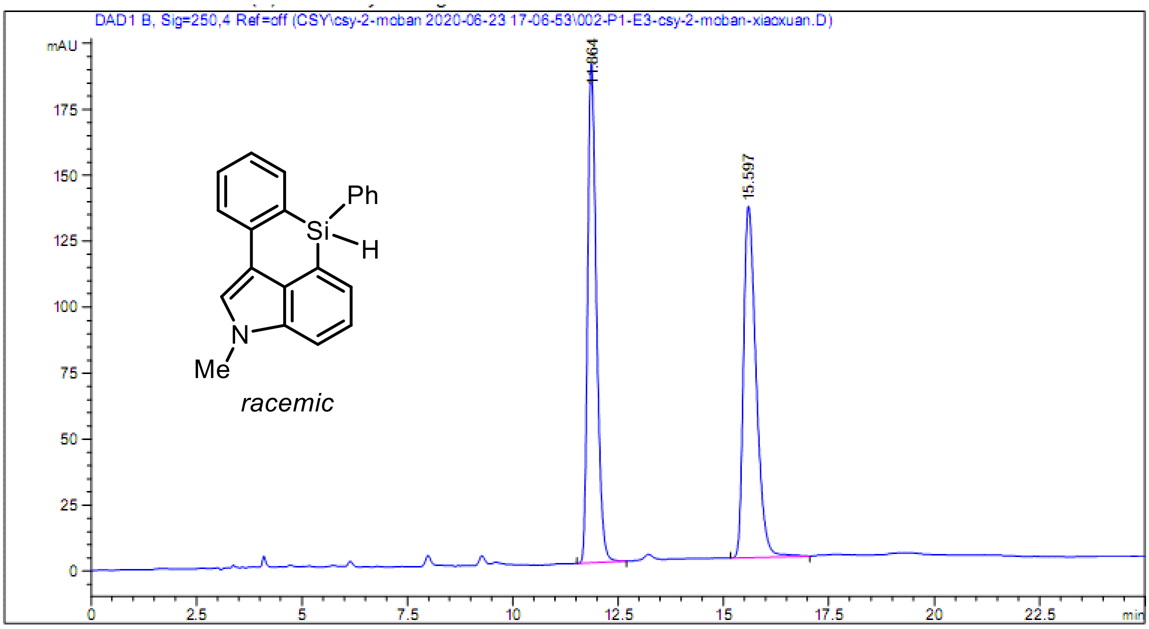

Peak #	RetTime [min]	Type	Width [min]	Area [mAU*s]	Height [mAU]	Area %
1	11.864	BB	0.2234	2748.67114	189.30153	49.7685
2	15.597	BB	0.3211	2774.23901	133.14737	50.2315

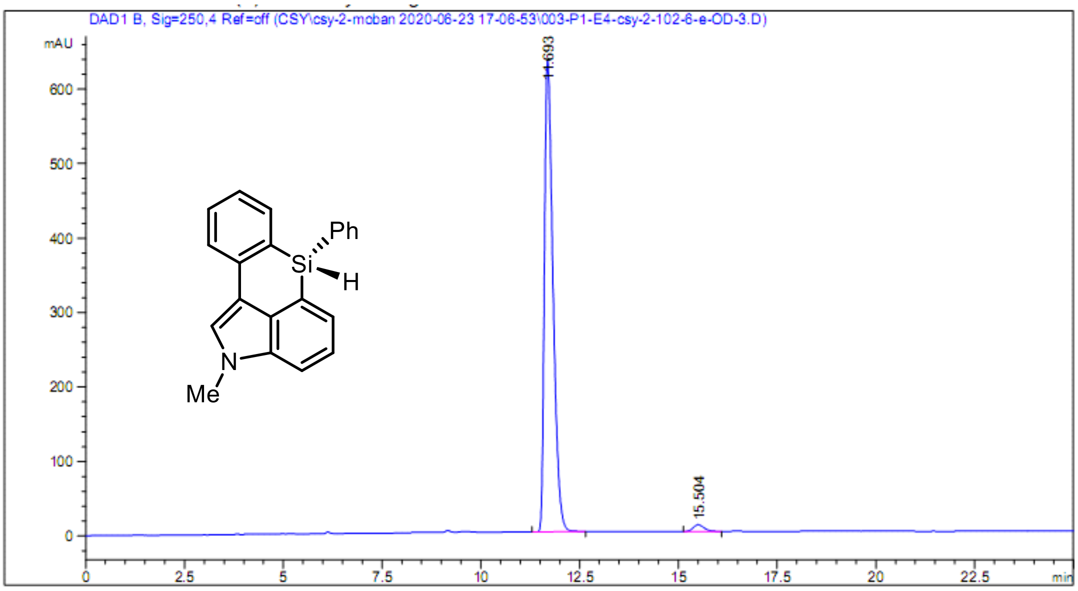

Peak #	RetTime [min]	Type	Width [min]	Area [mAU*s]	Height [mAU]	Area %
1	11.693	BB	0.2319	9557.10449	633.71014	98.2252
2	15.504	BB	0.2876	172.68571	8.92954	1.7748

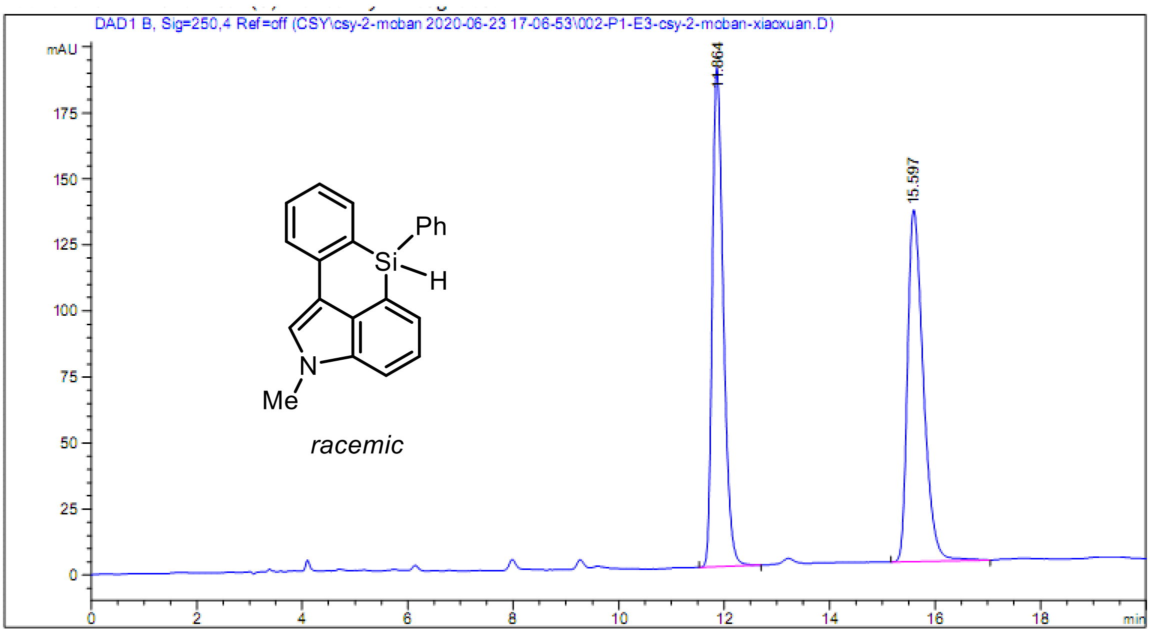

Peak #	RetTime [min]	Type	Width [min]	Area [mAU*s]	Height [mAU]	Area %
1	11.864	BB	0.2234	2748.67114	189.30153	49.7685
2	15.597	BB	0.3211	2774.23901	133.14737	50.2315

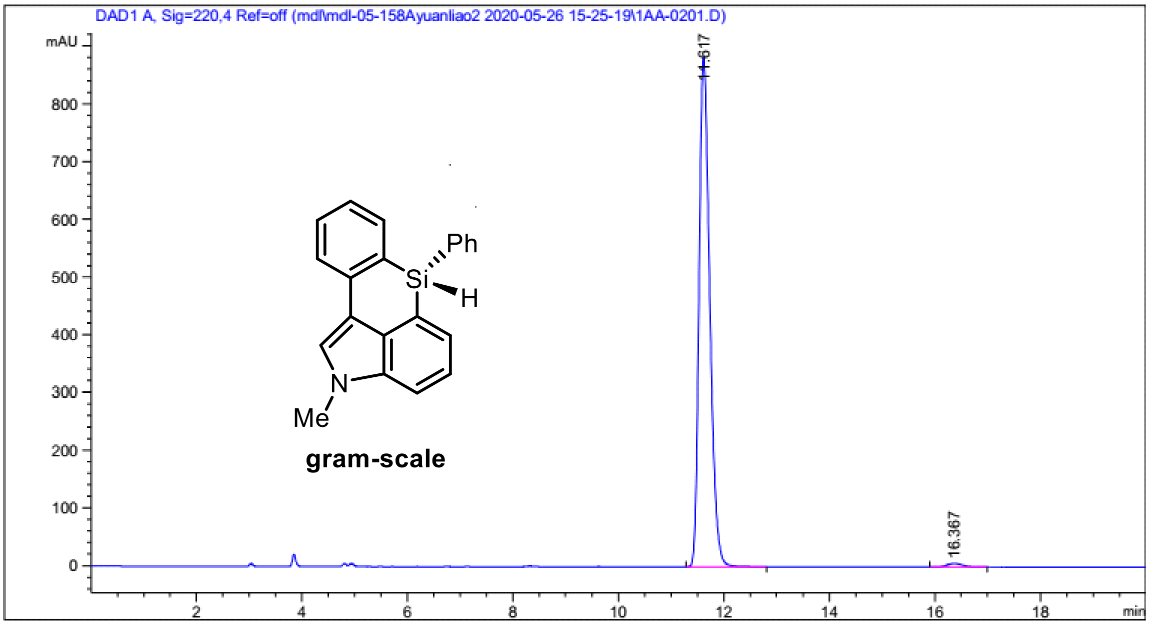

Peak #	RetTime [min]	Type	Width [min]	Area [mAU*s]	Height [mAU]	Area %
1	11.617	BB	0.2185	1.24229e4	881.03131	98.9602
2	16.367	BB	0.3095	130.52673	6.35764	1.0398

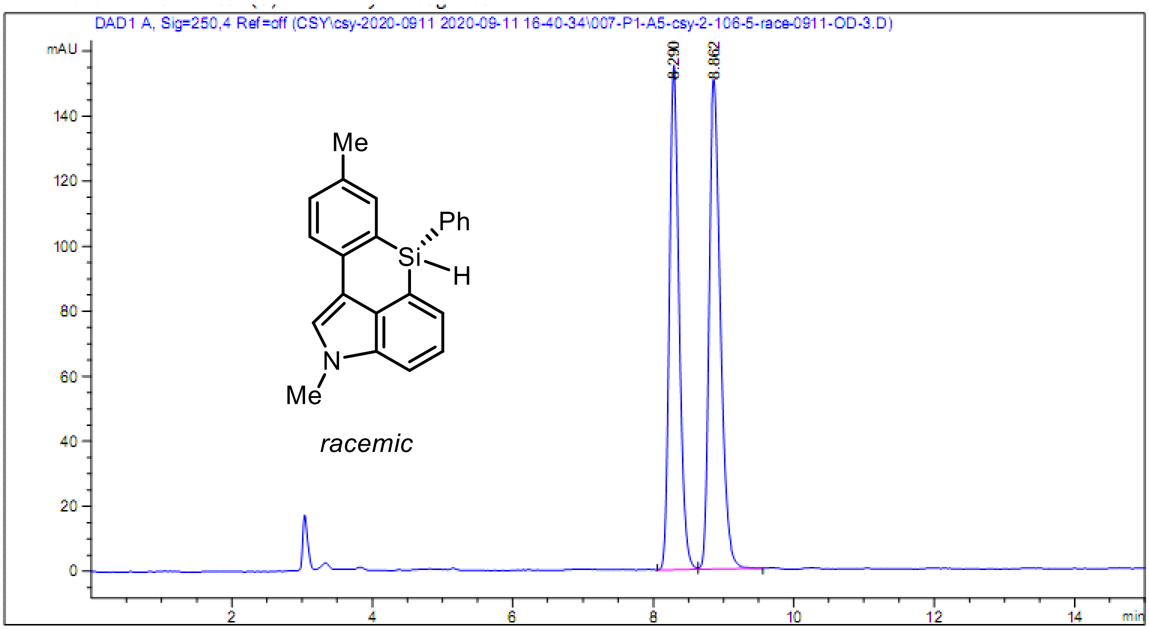

Peak #	RetTime [min]	Type	Width [min]	Area [mAU*s]	Height [mAU]	Area %
1	8.290	BV	0.1501	1518.10779	155.29291	47.2492
2	8.862	VB	0.1711	1694.87073	150.88129	52.7508

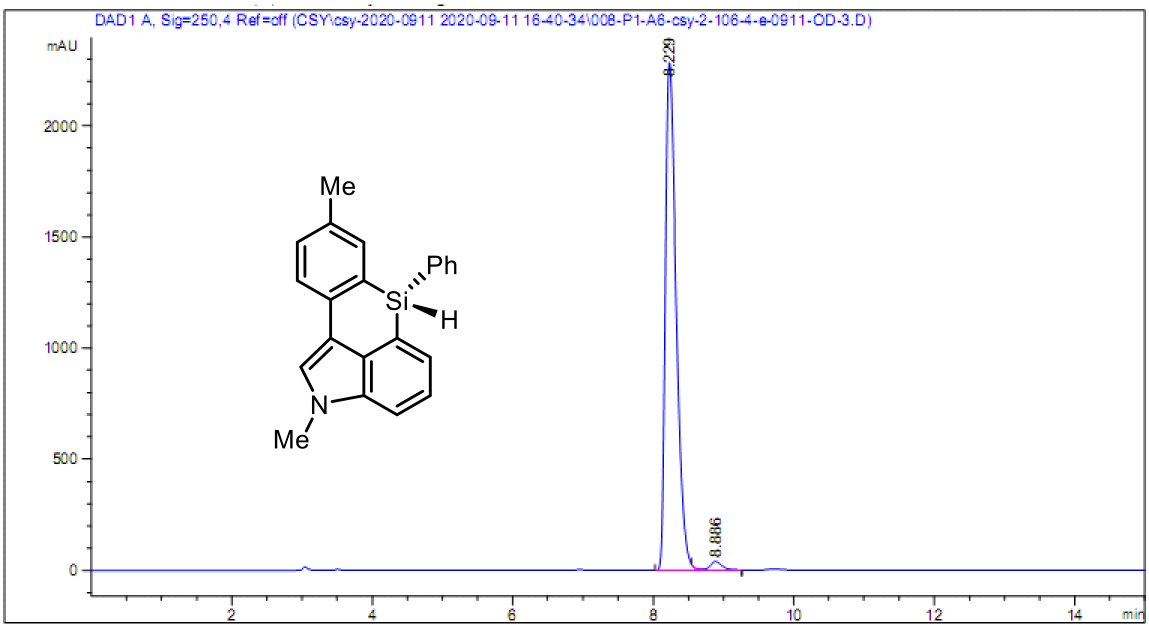

Peak #	RetTime [min]	Type	Width [min]	Area [mAU*s]	Height [mAU]	Area %
1	8.229	BV R	0.1652	2.45170e4	2284.09351	98.1966
2	8.886	VB E	0.1795	450.25708	38.20245	1.8034

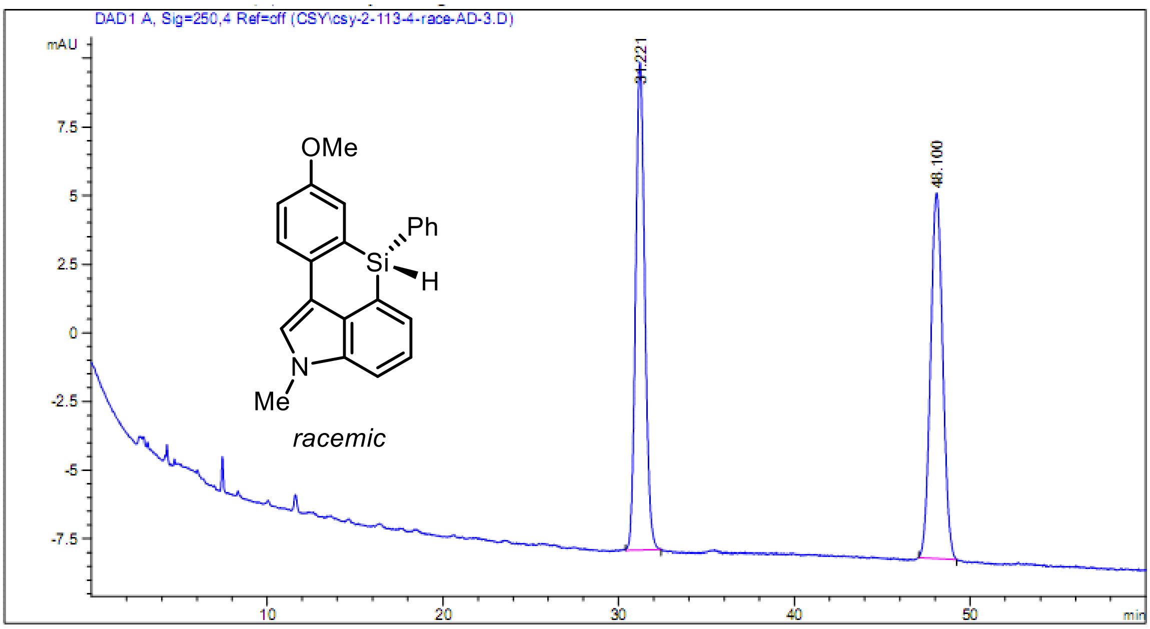

Peak #	RetTime [min]	Type	Width [min]	Area [mAU*s]	Height [mAU]	Area %
1	31.221	BB	0.5425	643.61505	17.77425	50.0317
2	48.100	BB	0.6218	642.79919	13.30128	49.9683

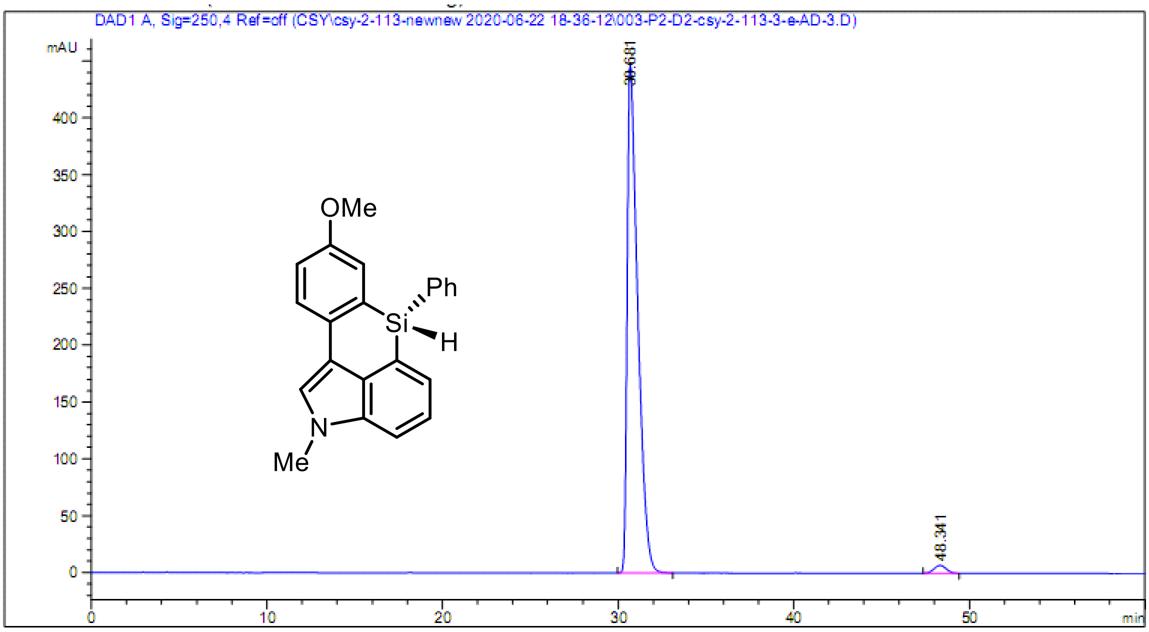

Peak #	RetTime [min]	Type	Width [min]	Area [mAU*s]	Height [mAU]	Area %
1	30.681	BB	0.6181	1.87256e4	447.62598	98.1724
2	48.341	BB	0.5916	348.59680	7.21593	1.8276

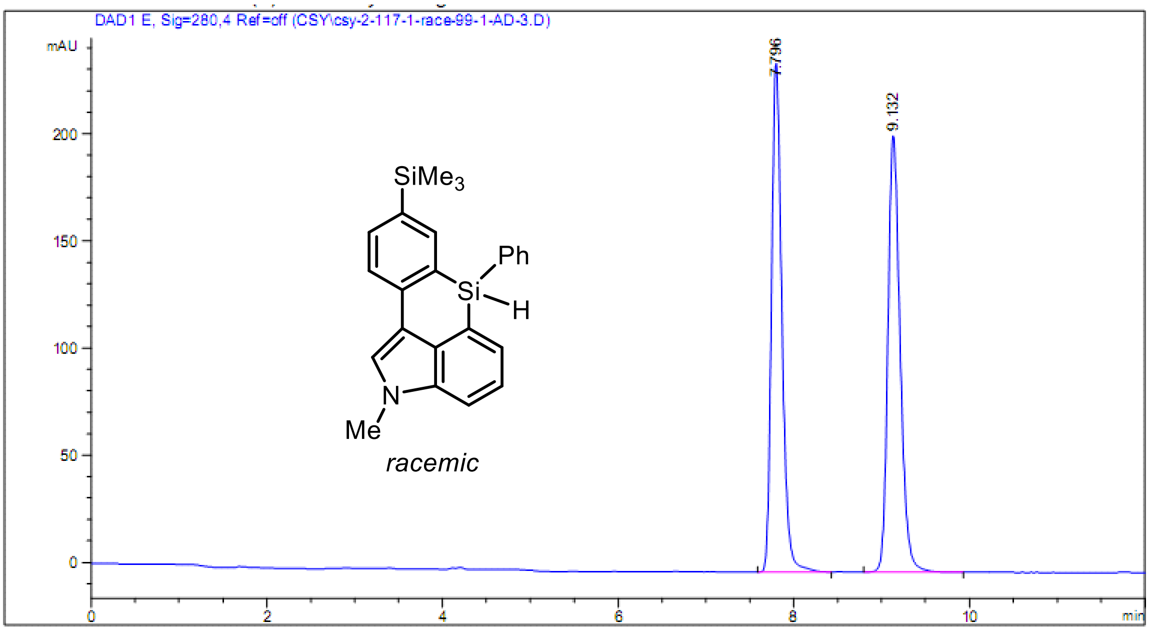

Peak #	RetTime [min]	Type	Width [min]	Area [mAU*s]	Height [mAU]	Area %
1	7.796	BB	0.1259	1949.95007	237.12775	49.7586
2	9.132	BB	0.1489	1968.86926	203.52560	50.2414

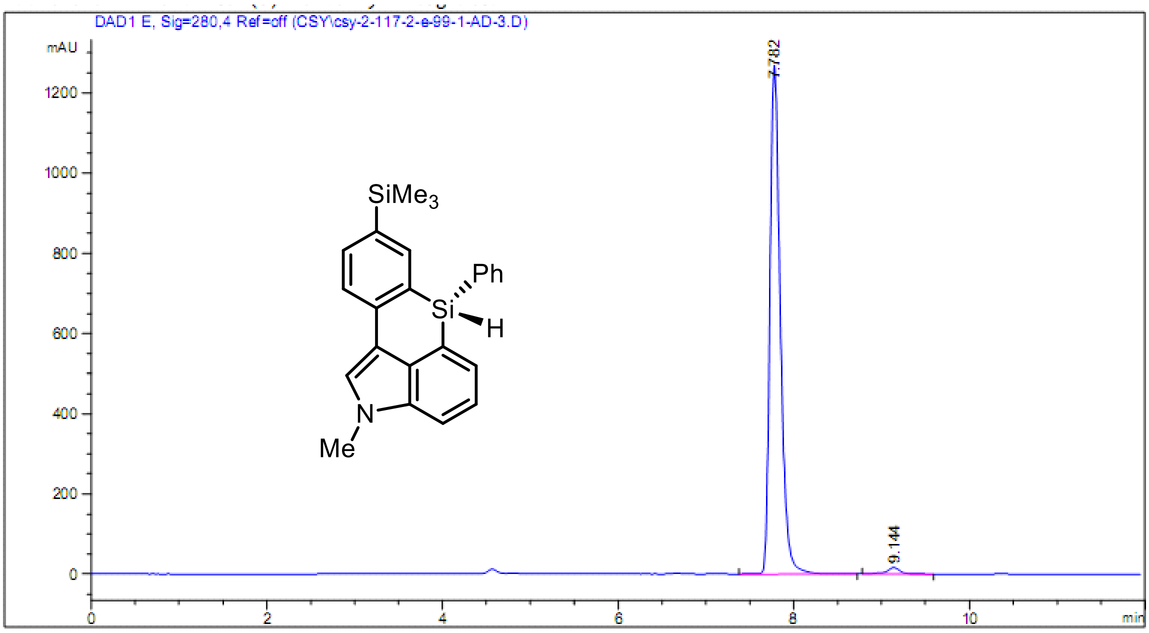

Peak #	RetTime [min]	Type	Width [min]	Area [mAU*s]	Height [mAU]	Area %
1	7.782	BB	0.1264	1.04826e4	1269.19507	98.3719
2	9.144	BB	0.1565	173.48804	16.25193	1.6281

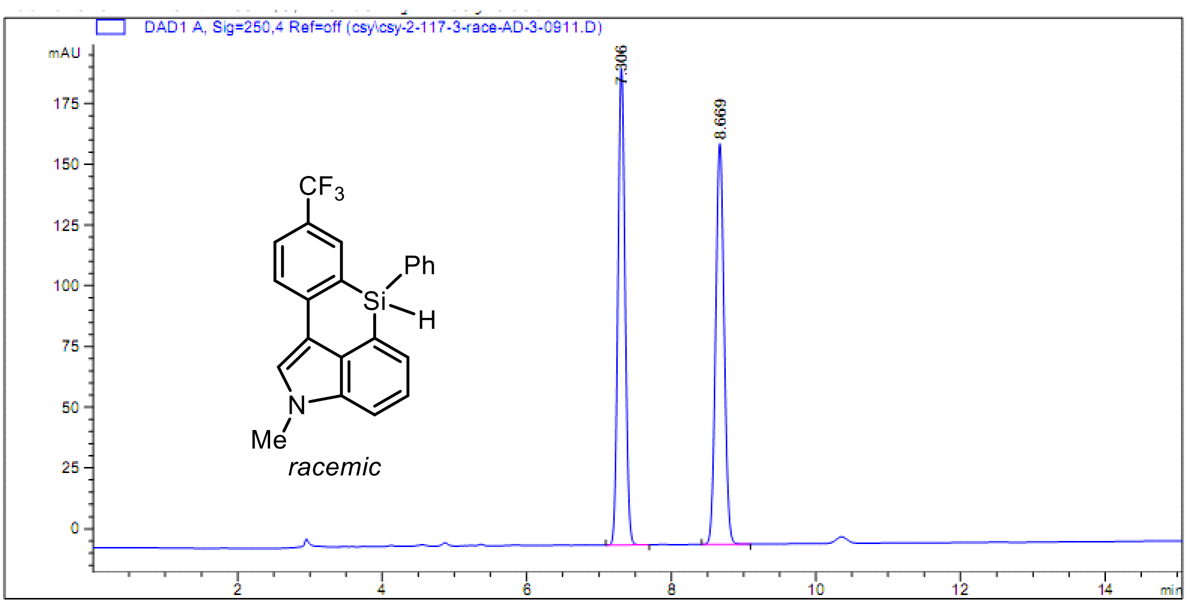

Peak #	RetTime [min]	Type	Width [min]	Area [mAU*s]	Height [mAU]	Area %
1	7.306	BB	0.1078	1342.07300	196.32816	49.9803
2	8.669	BB	0.1270	1343.13293	165.02065	50.0197

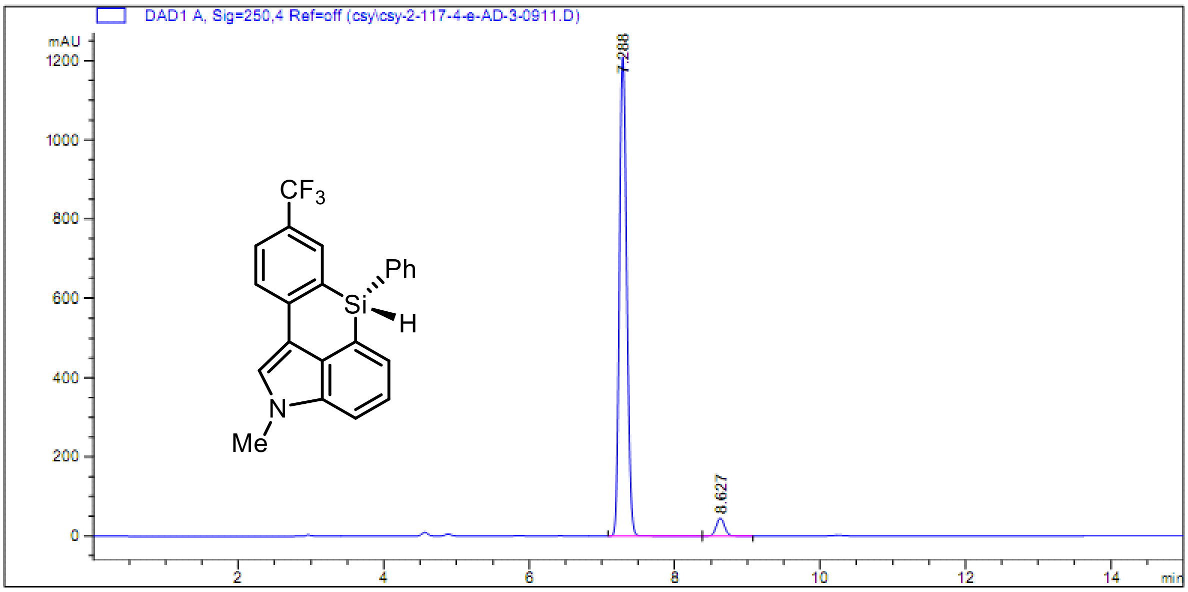

Peak #	RetTime [min]	Type	Width [min]	Area [mAU*s]	Height [mAU]	Area %
1	7.288	BB	0.1079	8300.38086	1212.13757	95.9021
2	8.627	BB	0.1227	354.67490	44.62615	4.0979

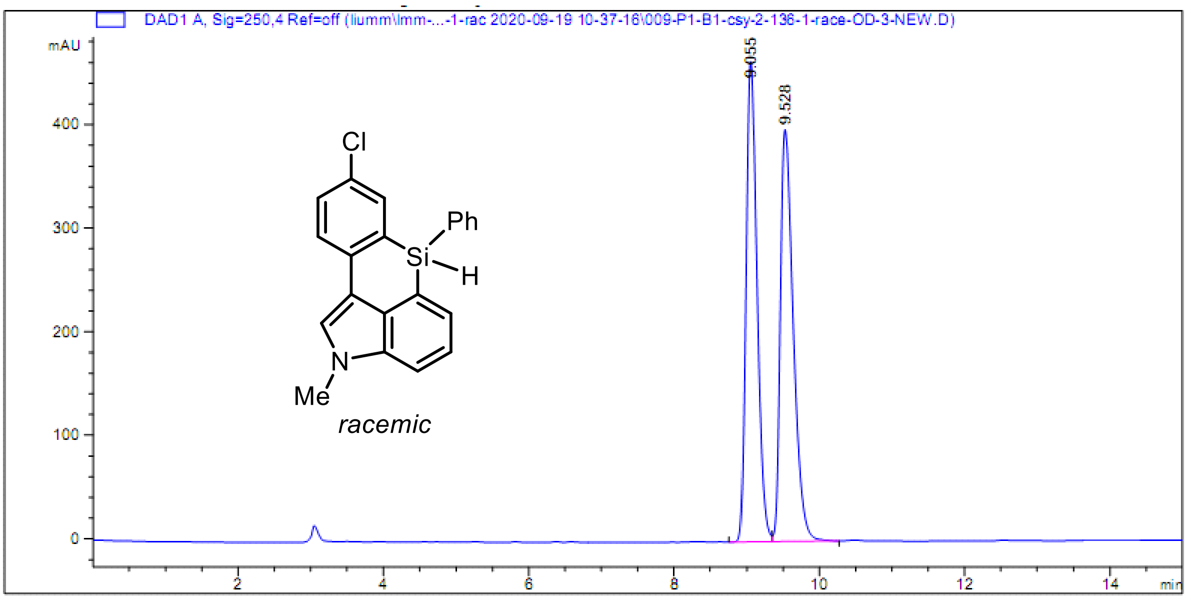

Peak #	RetTime [min]	Type	Width [min]	Area [mAU*s]	Height [mAU]	Area %
1	9.055	BV	0.1606	4800.50928	464.44989	49.5055
2	9.528	VB	0.1895	4896.41309	398.07642	50.4945

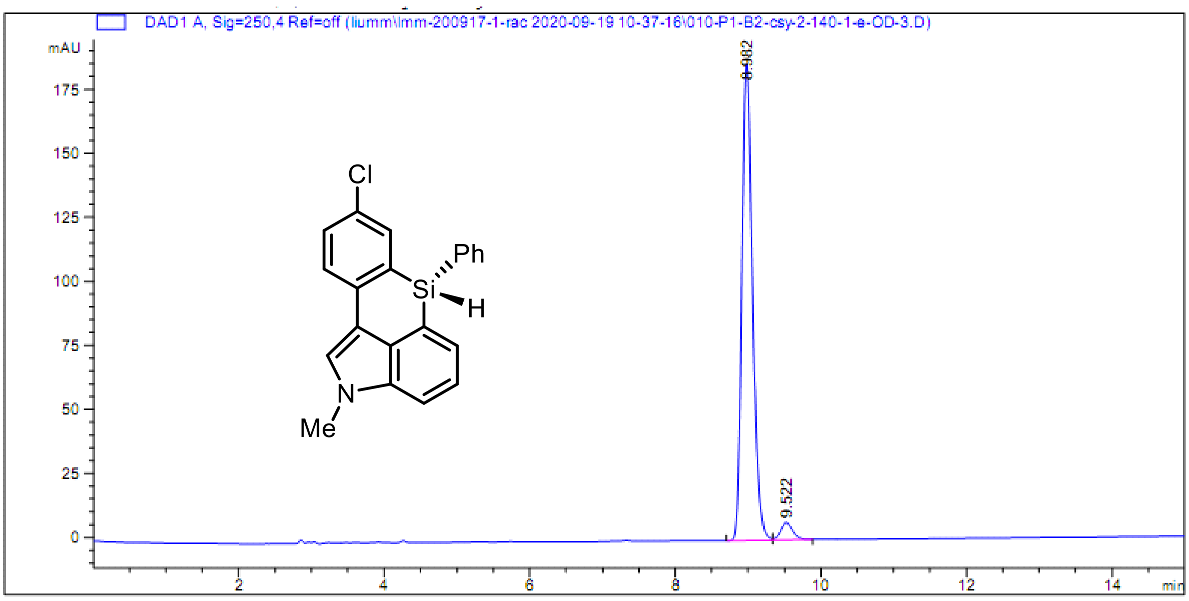

Peak #	RetTime [min]	Type	Width [min]	Area [mAU*s]	Height [mAU]	Area %
1	8.982	MF R	0.1691	1889.36548	186.19286	96.1528
2	9.522	FM R	0.1913	75.59692	6.58509	3.8472

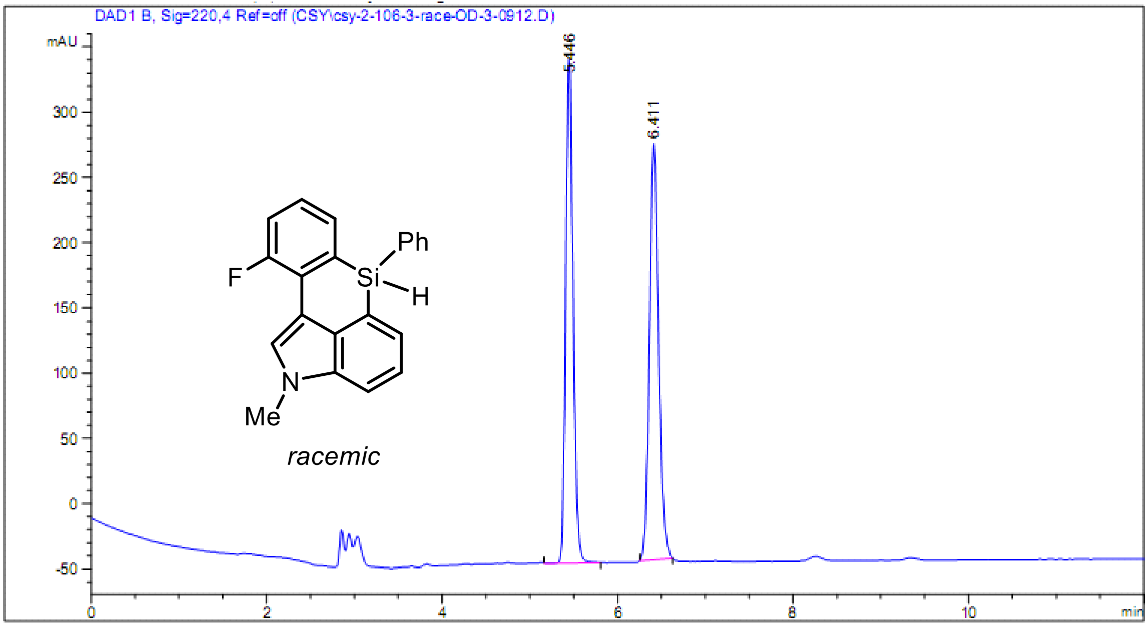

Peak #	RetTime [min]	Type	Width [min]	Area [mAU*s]	Height [mAU]	Area %
1	5.446	MM R	0.0968	2248.72388	387.00912	49.0353
2	6.411	MM R	0.1220	2337.20654	319.32523	50.9647

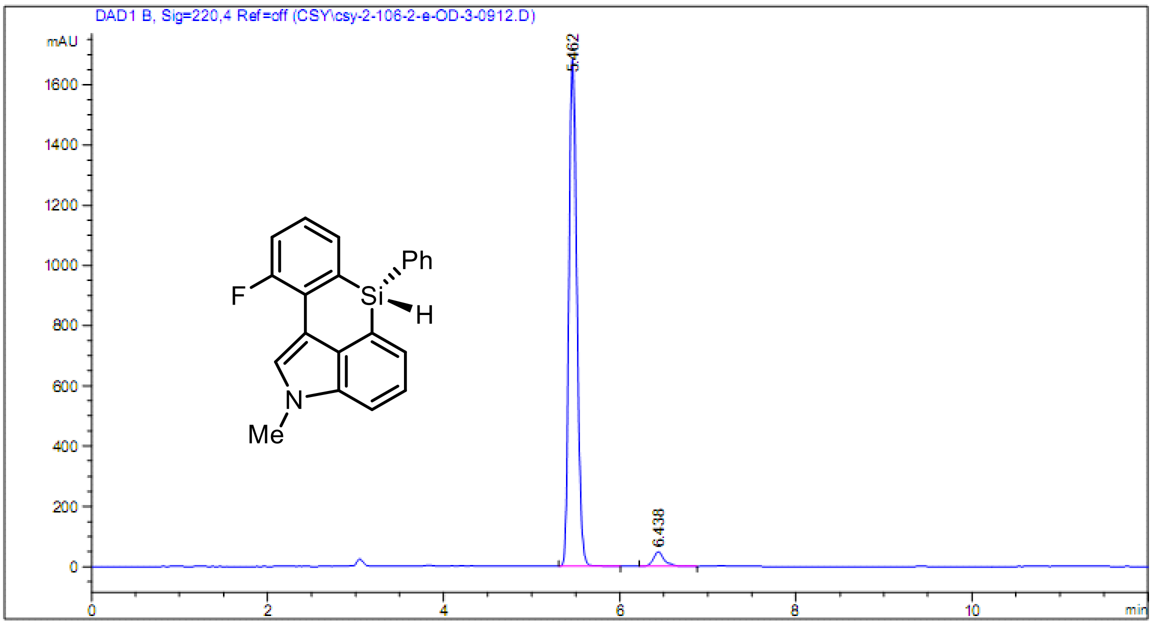

Peak #	RetTime [min]	Type	Width [min]	Area [mAU*s]	Height [mAU]	Area %
1	5.462	BB	0.0937	1.00798e4	1687.49207	95.8970
2	6.438	BB	0.1289	431.26443	48.88698	4.1030

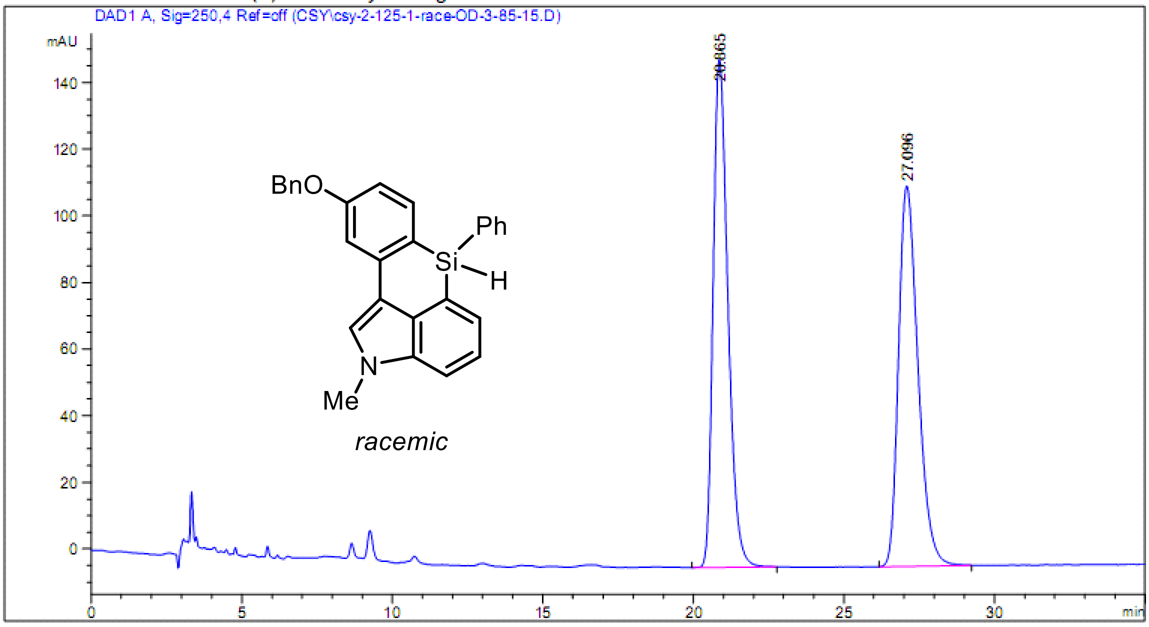

Peak #	RetTime [min]	Type	Width [min]	Area [mAU*s]	Height [mAU]	Area %
1	20.865	BB	0.5245	5215.26465	152.68034	50.3957
2	27.096	BB	0.6881	5133.36865	114.27600	49.6043

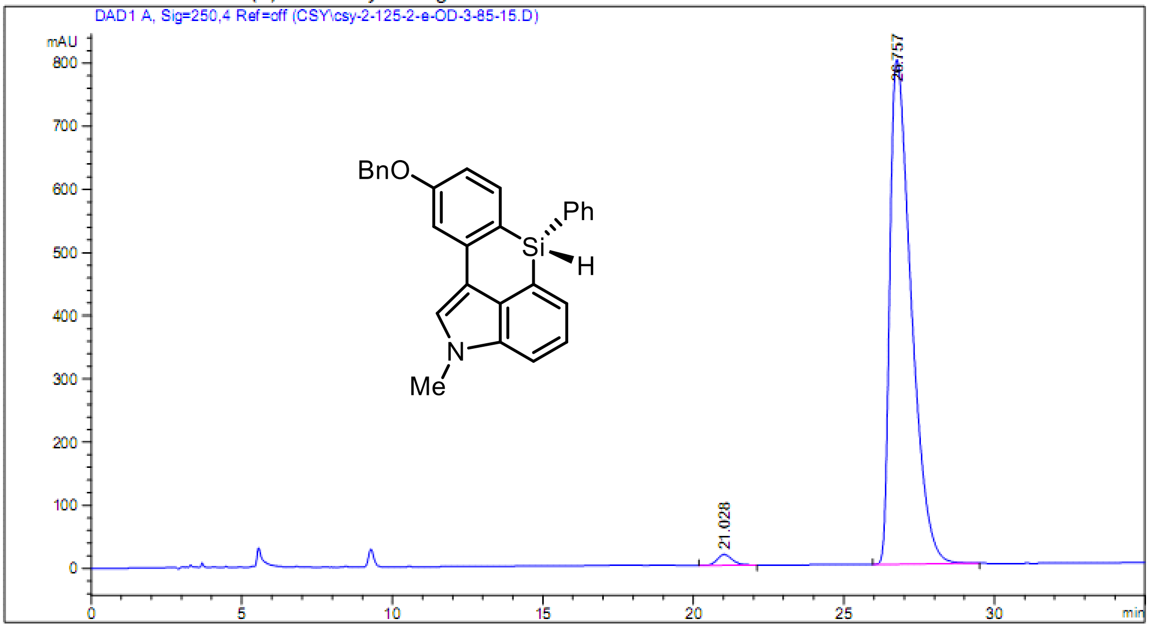

Peak #	RetTime [min]	Type	Width [min]	Area [mAU*s]	Height [mAU]	Area %
1	21.028	BB	0.5269	581.99030	16.93843	1.4547
2	26.757	BB	0.7475	3.94256e4	799.29529	98.5453

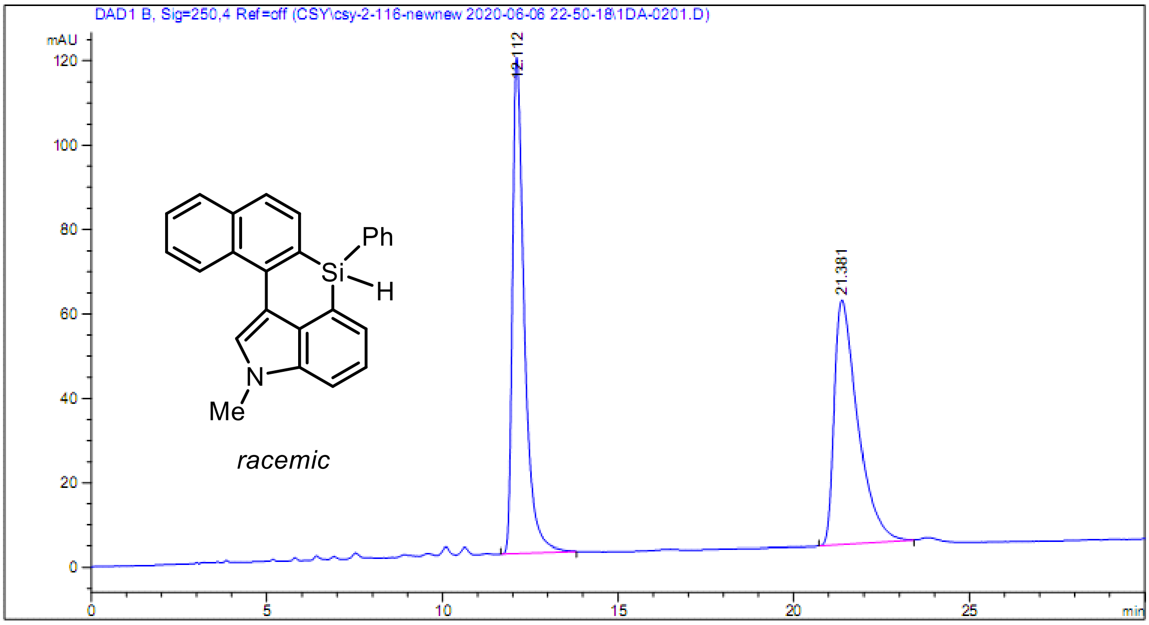

Peak #	RetTime [min]	Type	Width [min]	Area [mAU*s]	Height [mAU]	Area %
1	12.112	BB	0.3502	2759.41650	117.42313	50.5986
2	21.381	BB	0.6864	2694.12598	57.94521	49.4014

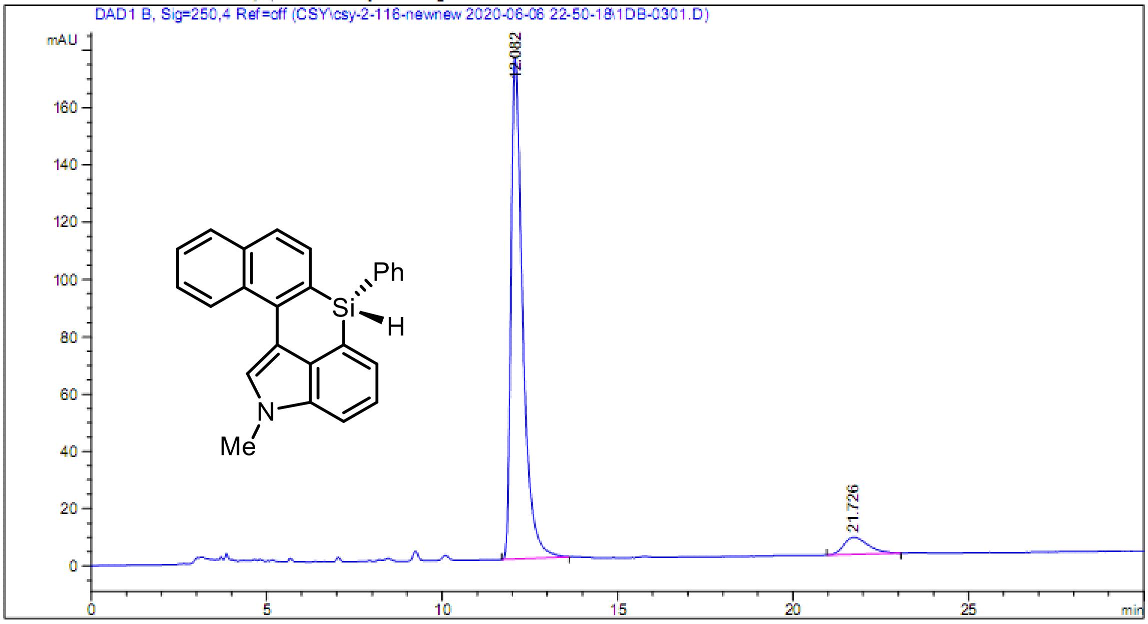

Peak #	RetTime [min]	Type	Width [min]	Area [mAU*s]	Height [mAU]	Area %
1	12.082	BB	0.3490	4066.16748	175.01926	93.3426
2	21.726	BB	0.5650	290.00824	6.05278	6.6574

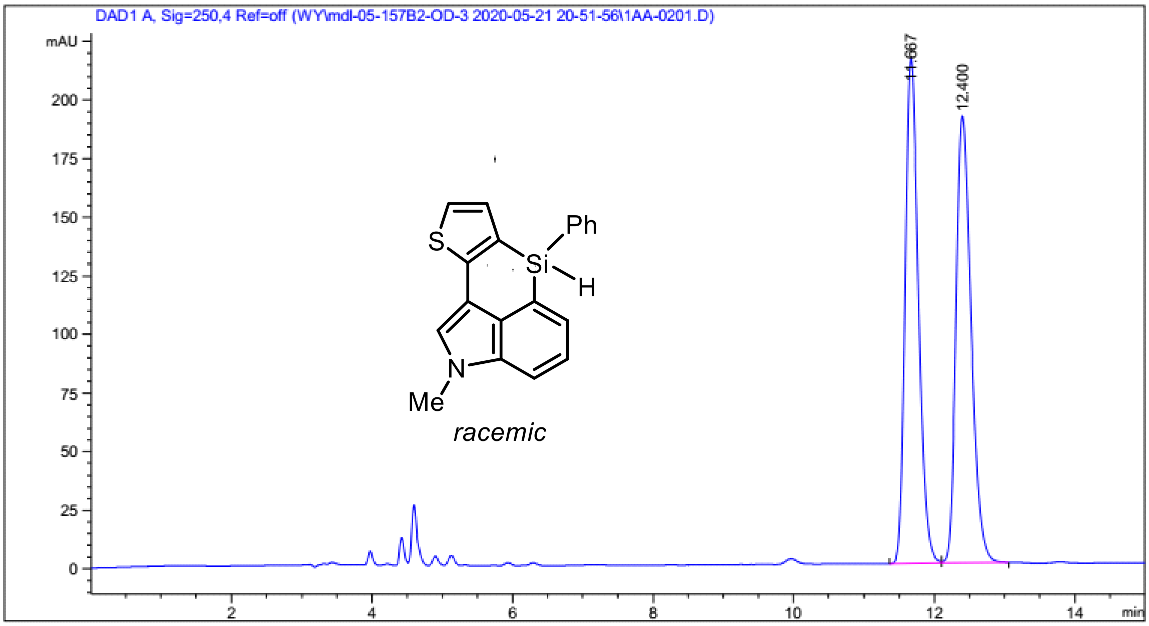

Peak #	RetTime [min]	Type	Width [min]	Area [mAU*s]	Height [mAU]	Area %
1	11.667	BV	0.2031	2829.31519	215.60249	49.8782
2	12.400	VB	0.2301	2843.13696	190.57463	50.1218

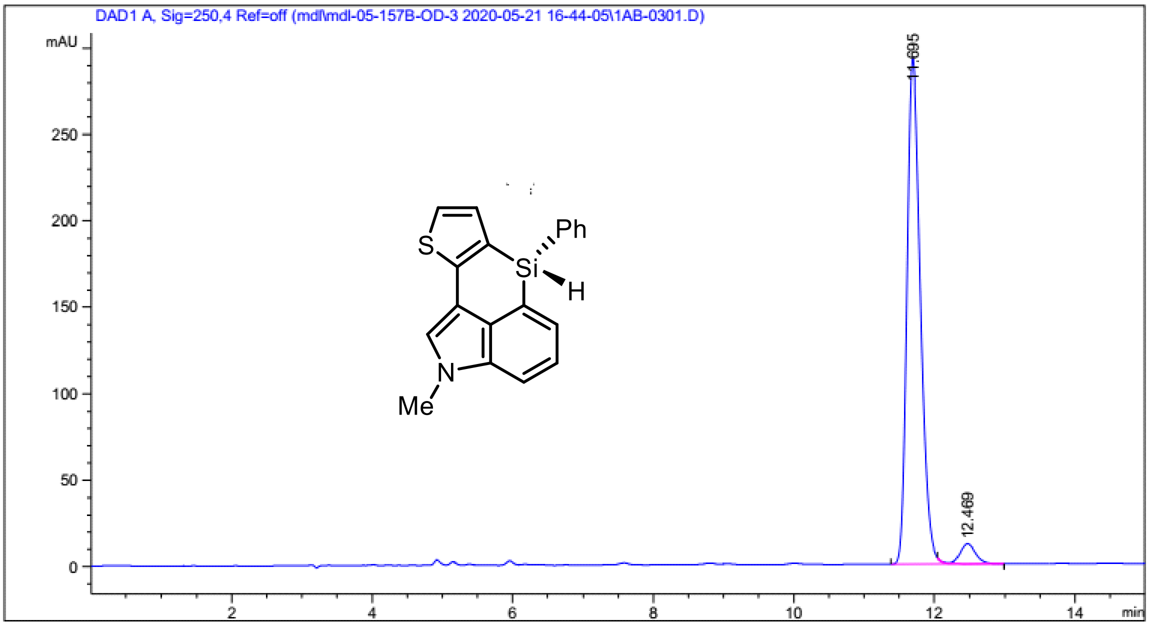

Peak #	RetTime [min]	Type	Width [min]	Area [mAU*s]	Height [mAU]	Area %
1	11.695	BV R	0.2074	3896.50171	292.56125	95.5281
2	12.469	VB E	0.2378	182.40567	11.83558	4.4719

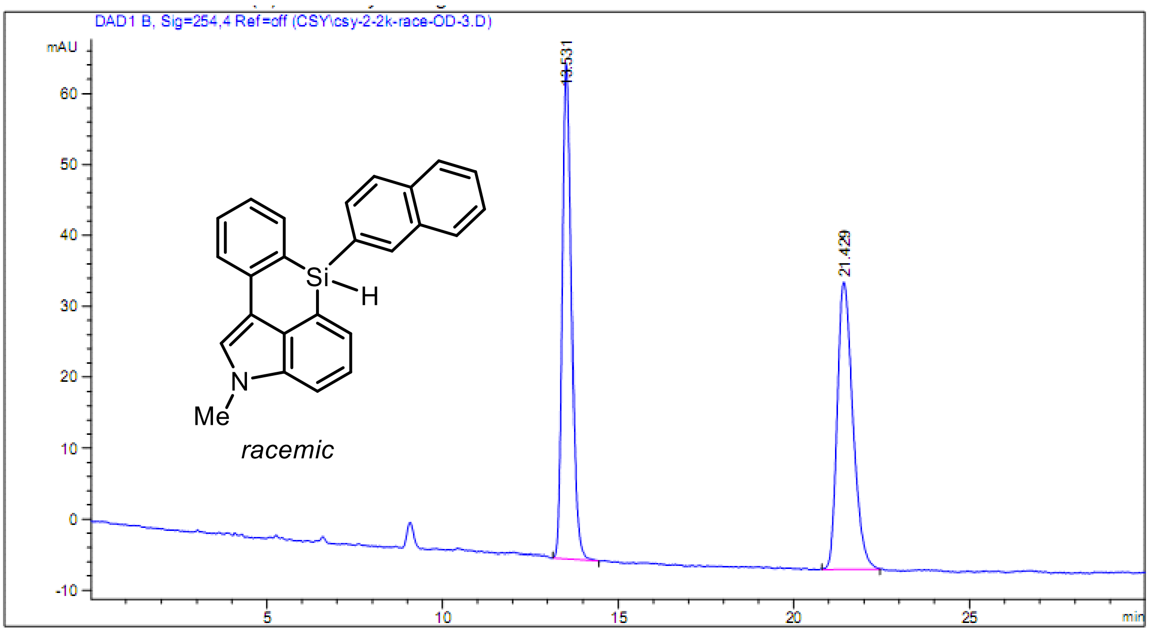

Peak #	RetTime [min]	Type	Width [min]	Area [mAU*s]	Height [mAU]	Area %
1	13.531	BB	0.2790	1260.36267	69.68239	50.2162
2	21.429	BB	0.4580	1249.51074	40.49051	49.7838

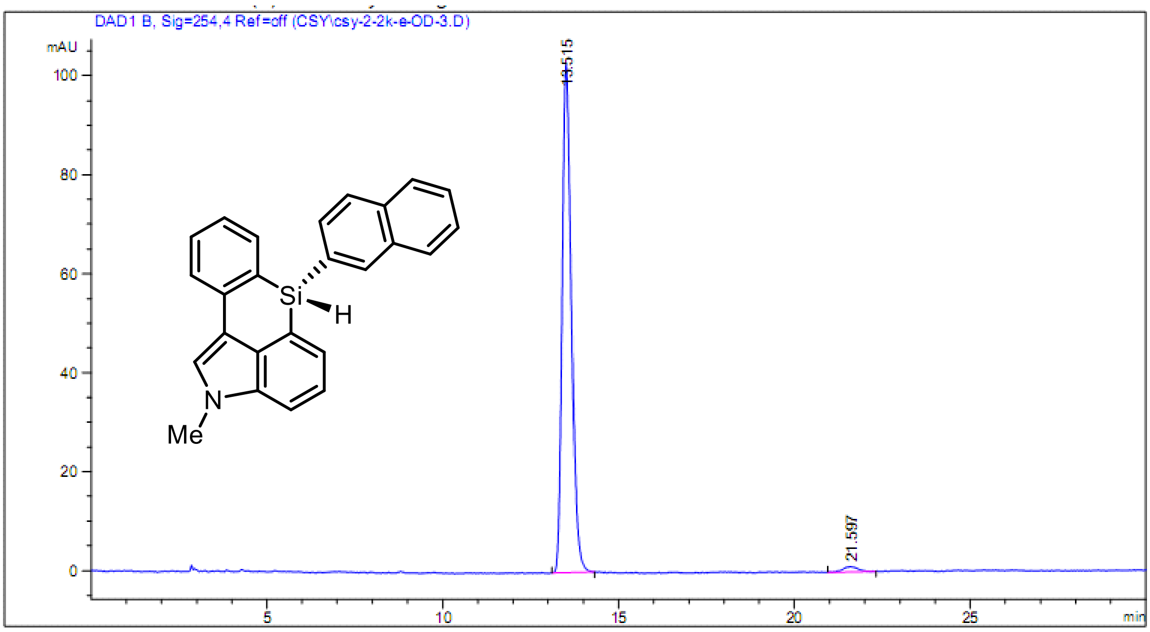

Peak #	RetTime [min]	Type	Width [min]	Area [mAU*s]	Height [mAU]	Area %
1	13.515	BB	0.2776	1843.20288	102.61981	98.2482
2	21.597	MM R	0.4971	32.86547	1.10195	1.7518

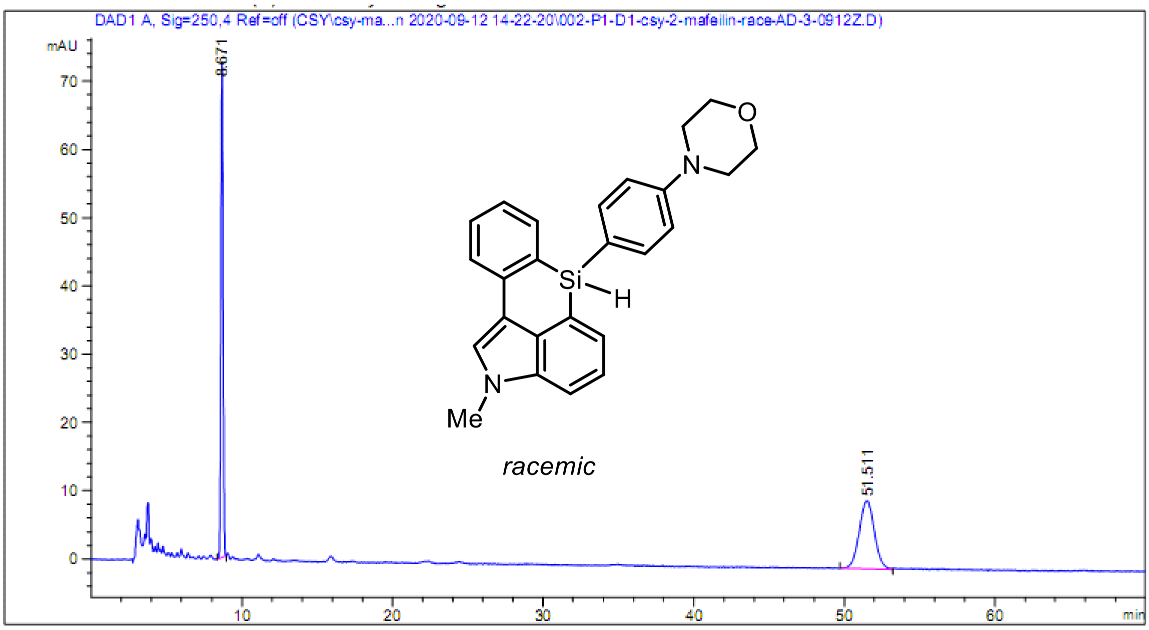

Peak #	RetTime [min]	Type	Width [min]	Area [mAU*s]	Height [mAU]	Area %
1	8.671	MM R	0.1631	708.26599	72.36091	50.4486
2	51.511	MM R	1.1708	695.66937	9.90293	49.5514

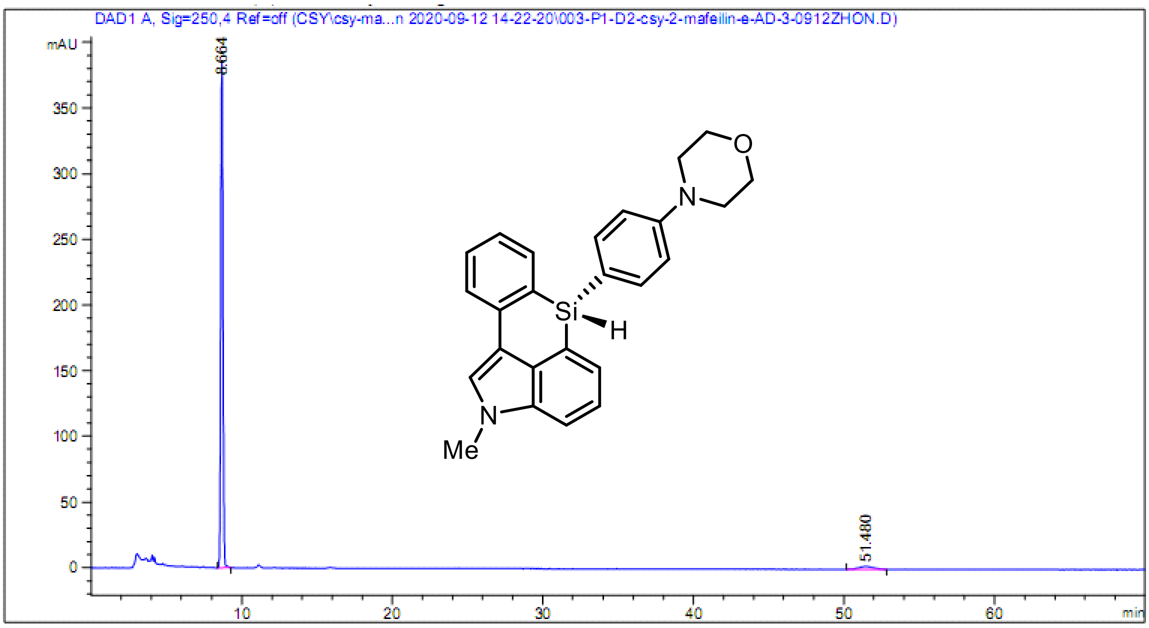

Peak #	RetTime [min]	Type	Width [min]	Area [mAU*s]	Height [mAU]	Area %
1	8.664	BB	0.1537	3819.60596	385.18637	96.2729
2	51.480	MM R	1.1735	147.87158	2.10011	3.7271

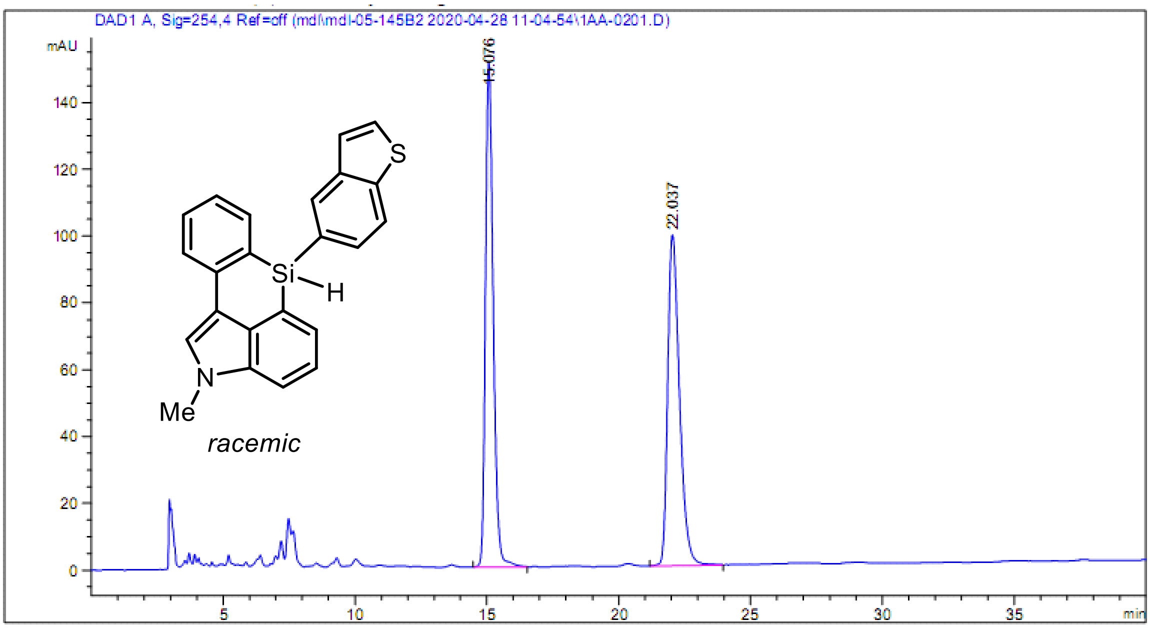

Peak #	RetTime [min]	Type	Width [min]	Area [mAU*s]	Height [mAU]	Area %
1	15.076	BB	0.3155	3098.71362	150.94339	50.1961
2	22.037	BB	0.4763	3074.50757	98.99536	49.8039

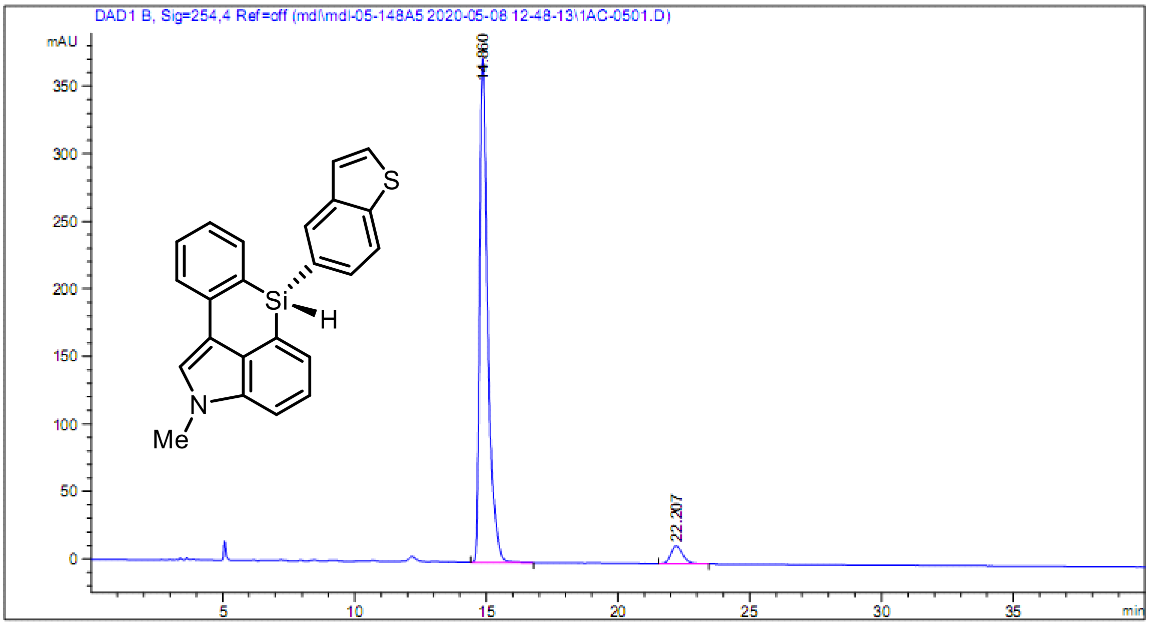

Peak #	RetTime [min]	Type	Width [min]	Area [mAU*s]	Height [mAU]	Area %
1	14.860	BB	0.3220	7994.50732	373.00394	95.0263
2	22.207	BB	0.4742	418.43558	13.25923	4.9737

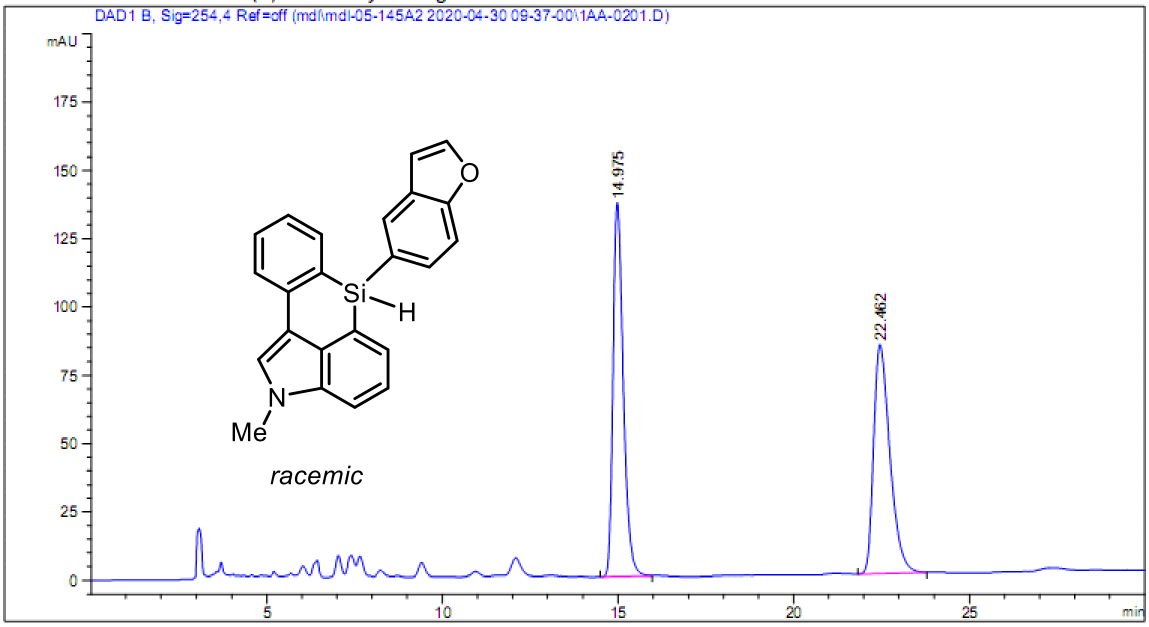

Peak #	RetTime [min]	Type	Width [min]	Area [mAU*s]	Height [mAU]	Area %
1	14.975	BB	0.3114	2784.14429	136.82396	50.6694
2	22.462	BB	0.4975	2710.58203	83.77981	49.3306

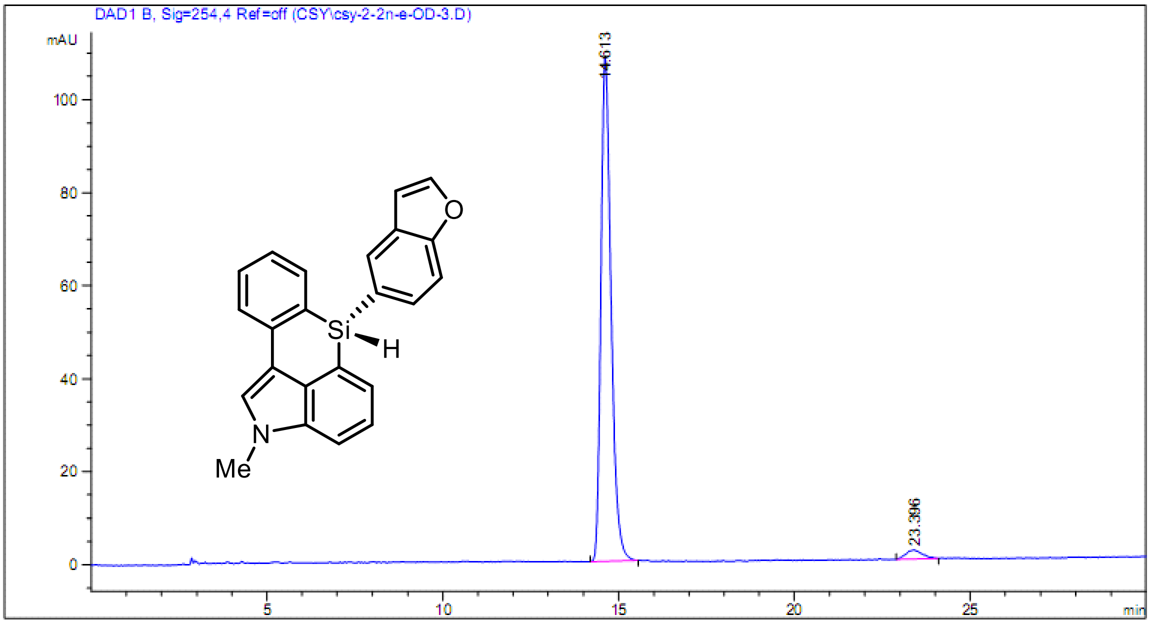

Peak #	RetTime [min]	Type	Width [min]	Area [mAU*s]	Height [mAU]	Area %
1	14.613	BB	0.3126	2177.18628	108.29329	97.2273
2	23.396	BB	0.3937	62.08735	1.89794	2.7727

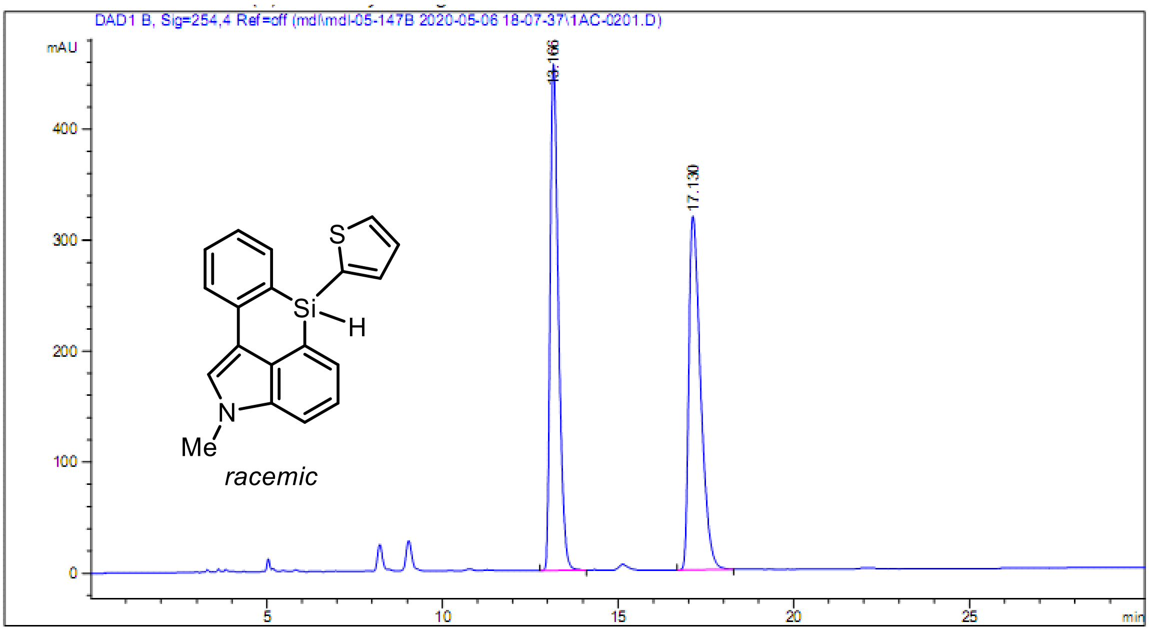

Peak #	RetTime [min]	Type	Width [min]	Area [mAU*s]	Height [mAU]	Area %
1	13.166	BB	0.2526	7448.05713	456.00995	49.9635
2	17.130	BB	0.3595	7458.94141	318.21225	50.0365

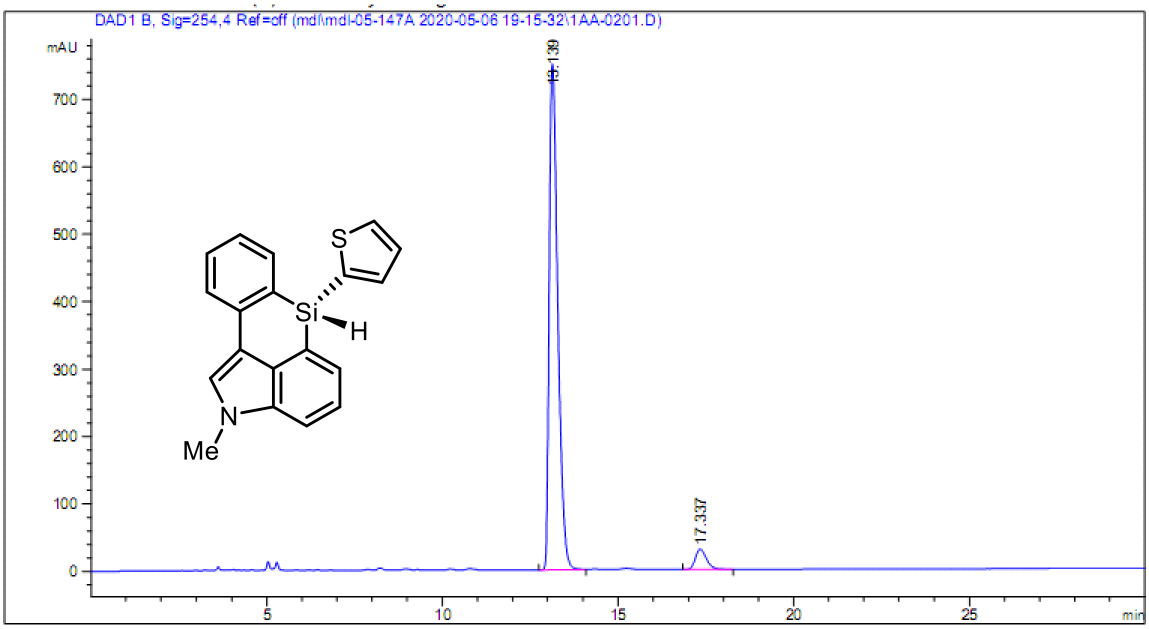

Peak #	RetTime [min]	Type	Width [min]	Area [mAU*s]	Height [mAU]	Area %
1	13.139	BB	0.2587	1.26506e4	750.36188	94.9847
2	17.337	BB	0.3408	667.96411	30.35104	5.0153

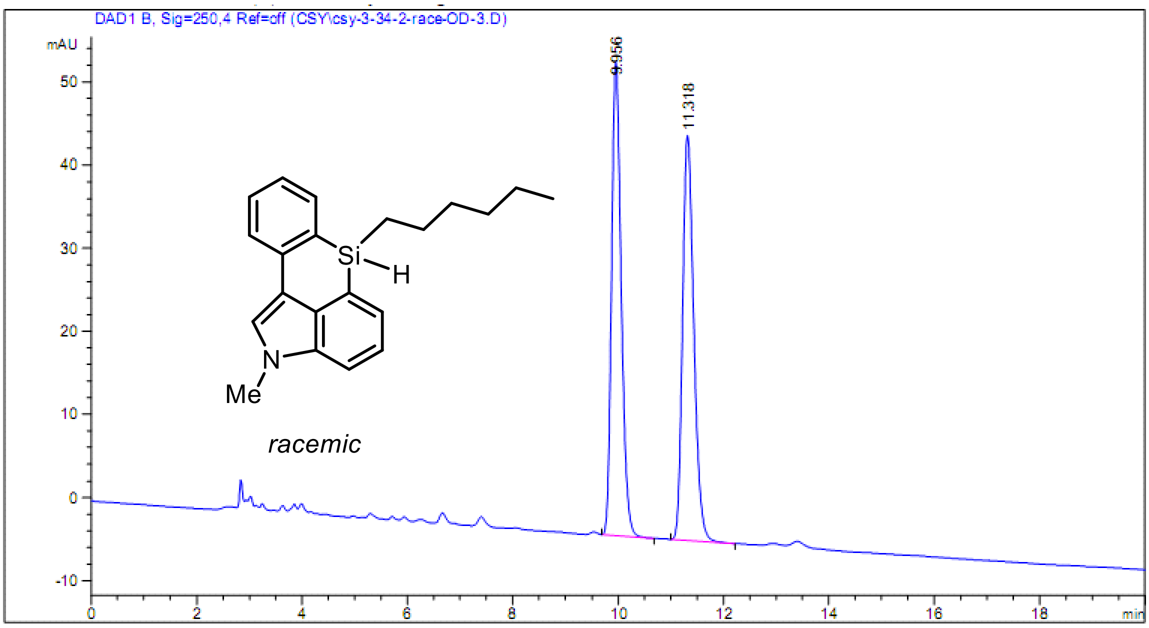

Peak #	RetTime [min]	Type	Width [min]	Area [mAU*s]	Height [mAU]	Area %
1	9.956	BB	0.1944	724.36407	56.94525	50.0376
2	11.318	BB	0.2292	723.27441	48.73058	49.9624

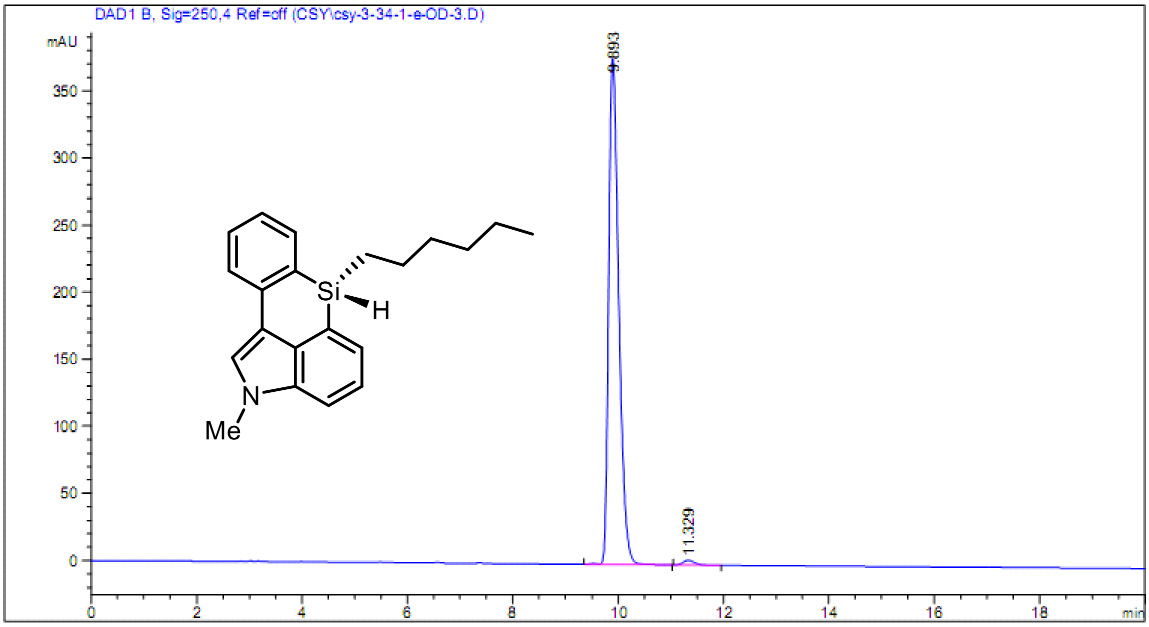

Peak #	RetTime [min]	Type	Width [min]	Area [mAU*s]	Height [mAU]	Area %
1	9.893	VB R	0.2031	5017.00146	377.26205	98.9572
2	11.329	BB	0.2269	52.86722	3.56813	1.0428

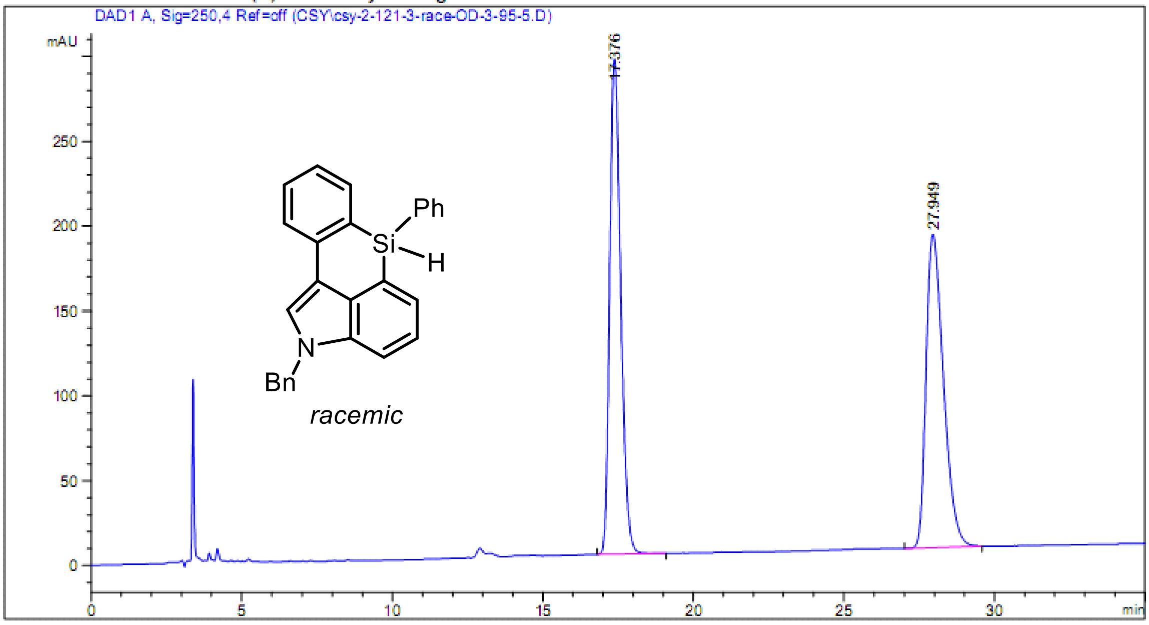

Peak #	RetTime [min]	Type	Width [min]	Area [mAU*s]	Height [mAU]	Area %
1	17.376	BB	0.3927	7420.21436	291.79440	50.0383
2	27.949	BB	0.6170	7408.84229	184.30550	49.9617

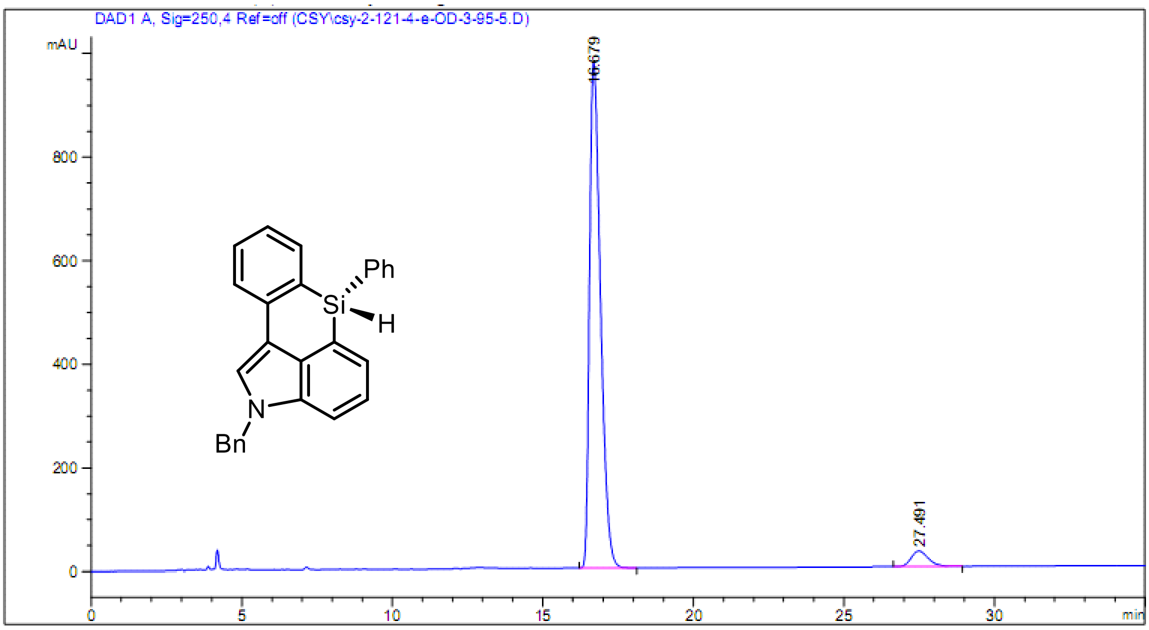

Peak #	RetTime [min]	Type	Width [min]	Area [mAU*s]	Height [mAU]	Area %
1	16.679	BB	0.3919	2.47828e4	977.09082	95.3658
2	27.491	BB	0.6022	1204.30151	30.26572	4.6342

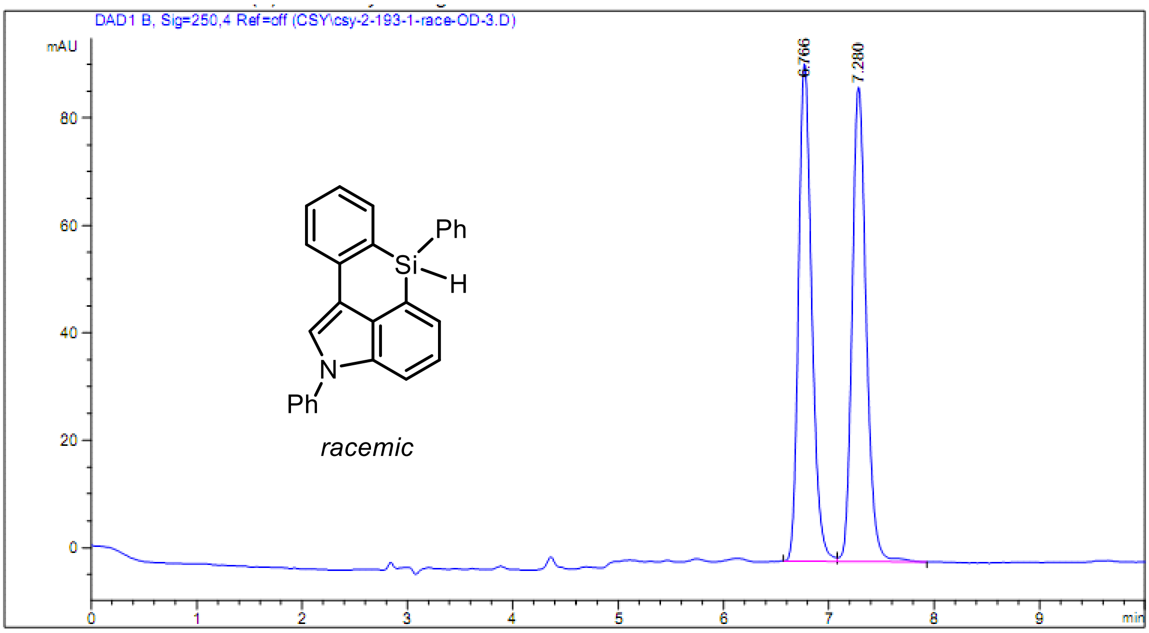

Peak #	RetTime [min]	Type	Width [min]	Area [mAU*s]	Height [mAU]	Area %
1	6.766	BV	0.1338	808.83246	92.67847	49.6423
2	7.280	VB	0.1423	820.48962	88.41254	50.3577

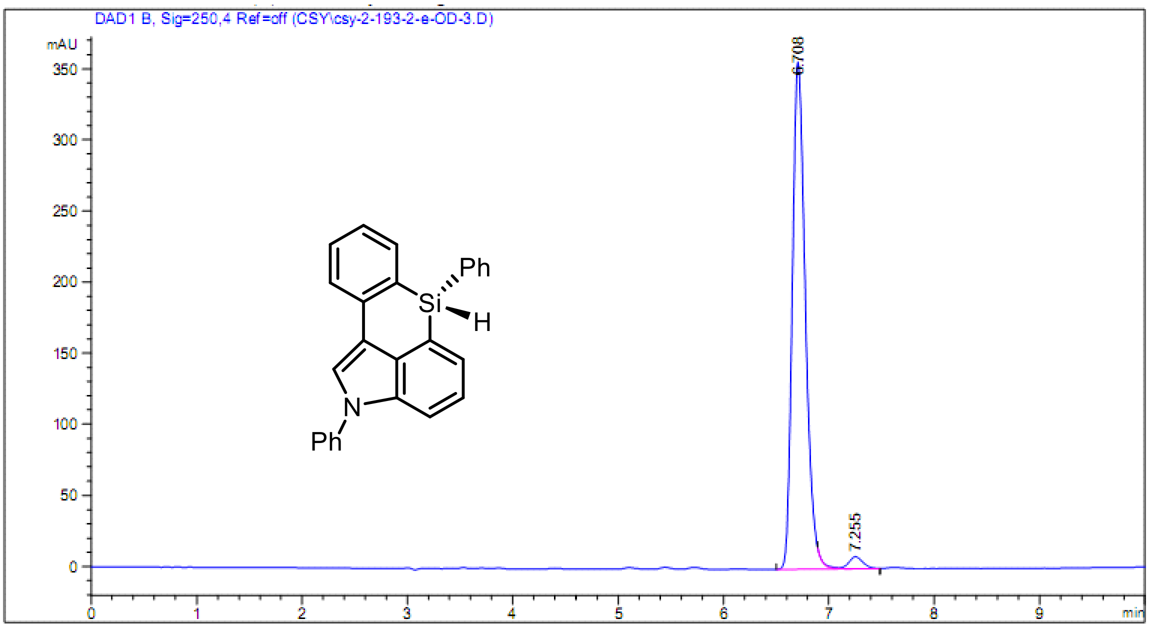

Peak #	RetTime [min]	Type	Width [min]	Area [mAU*s]	Height [mAU]	Area %
1	6.708	BV R	0.1326	3074.99512	356.76843	97.3233
2	7.255	VB E	0.1483	84.57084	8.48608	2.6767

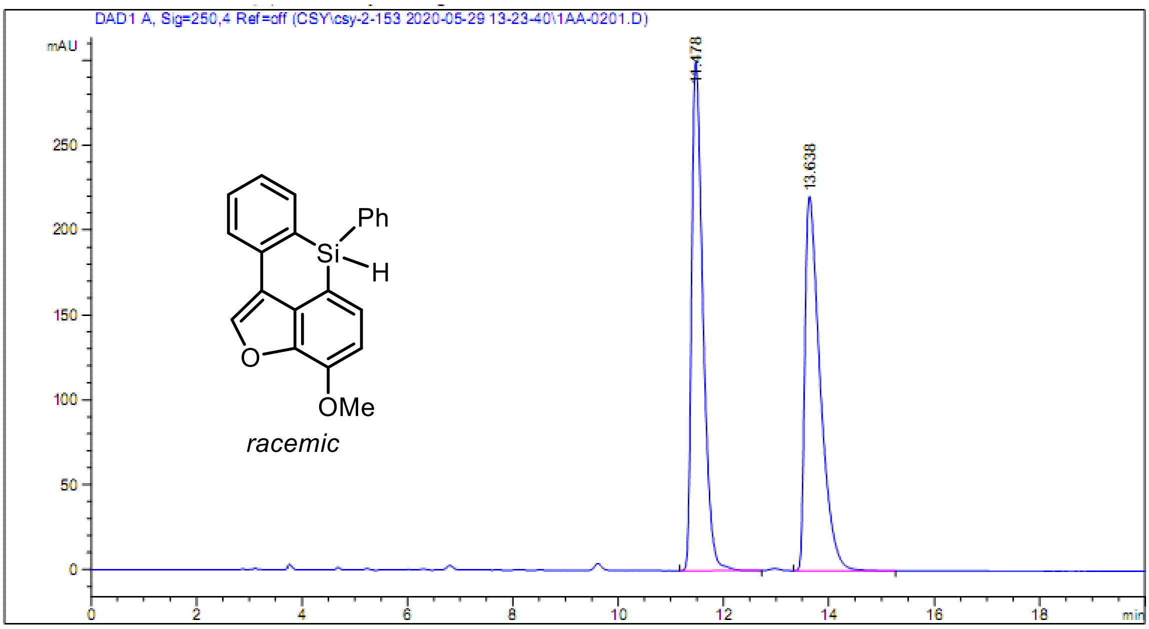

Peak #	RetTime [min]	Type	Width [min]	Area [mAU*s]	Height [mAU]	Area %
1	11.478	BB	0.2282	4472.62305	299.61514	50.1170
2	13.638	BB	0.3015	4451.73389	220.51158	49.8830

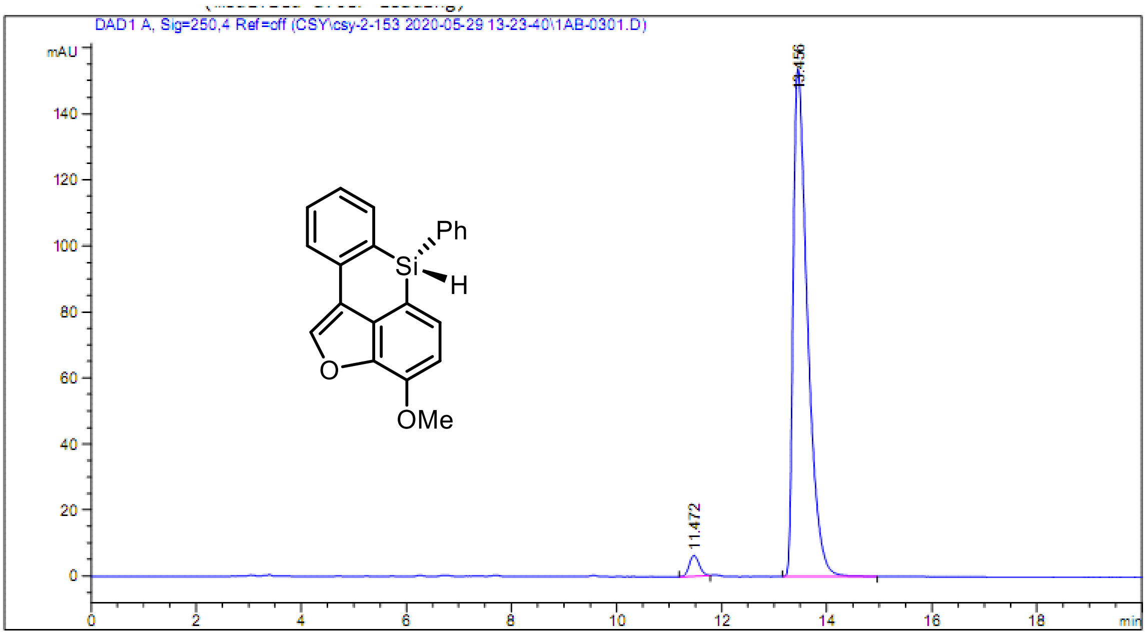

Peak #	RetTime [min]	Type	Width [min]	Area [mAU*s]	Height [mAU]	Area %
1	11.472	BB	0.2029	80.39582	6.13331	2.7024
2	13.456	BB	0.2836	2894.57349	153.77072	97.2976

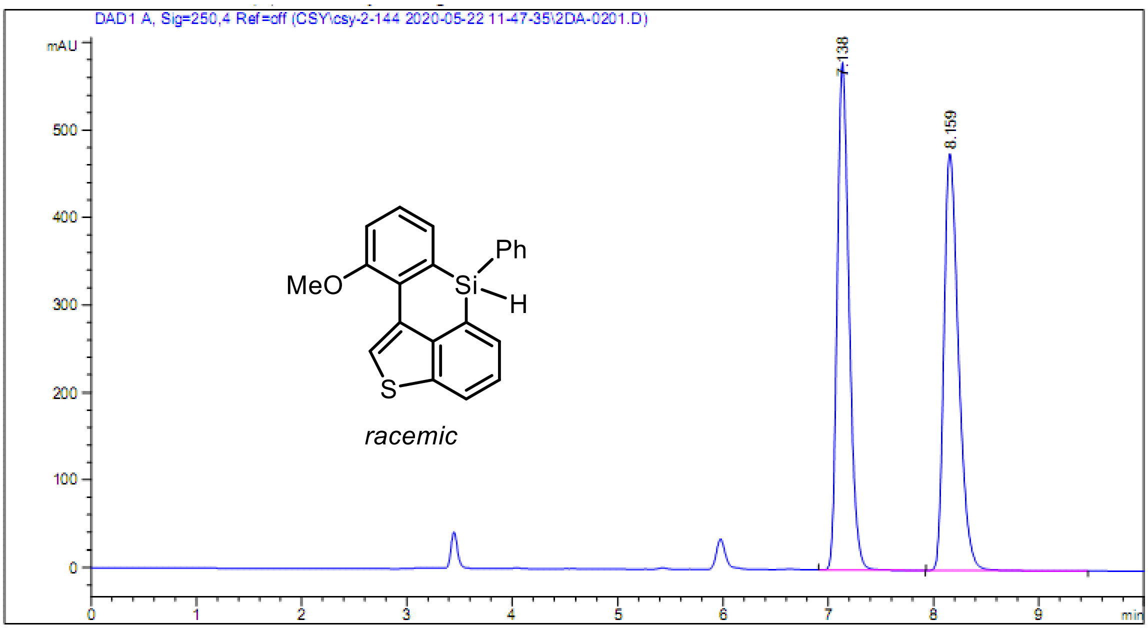

Peak #	RetTime [min]	Type	Width [min]	Area [mAU*s]	Height [mAU]	Area %
1	7.138	BB	0.1213	4541.96387	580.32104	49.7173
2	8.159	BB	0.1485	4593.61914	476.51880	50.2827

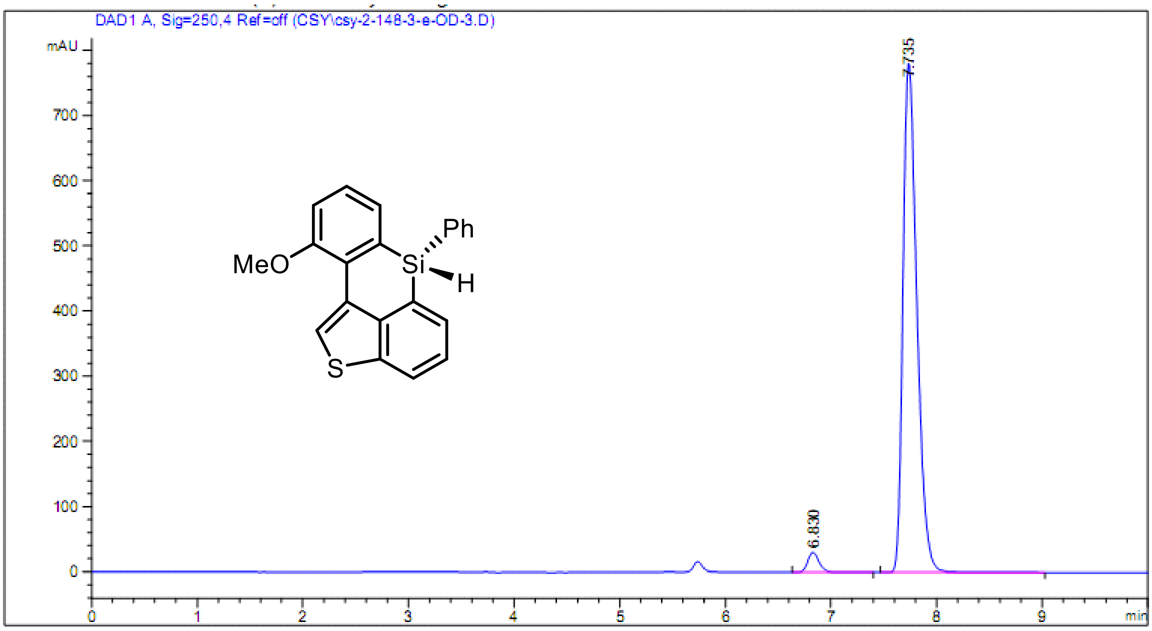

Peak #	RetTime [min]	Type	Width [min]	Area [mAU*s]	Height [mAU]	Area %
1	6.830	BB	0.1154	226.53676	30.23831	3.0672
2	7.735	BB	0.1409	7159.13916	781.38623	96.9328

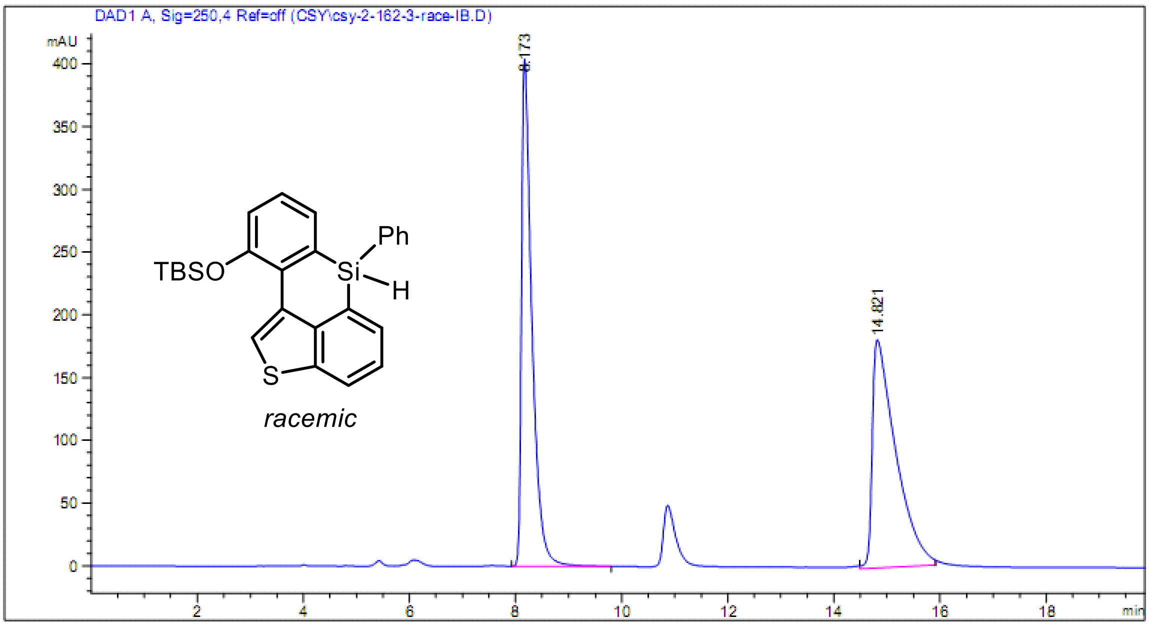

Peak #	RetTime [min]	Type	Width [min]	Area [mAU*s]	Height [mAU]	Area %
1	8.173	BB	0.1990	5510.95605	404.31238	49.6637
2	14.821	MM R	0.5120	5585.59717	181.81107	50.3363

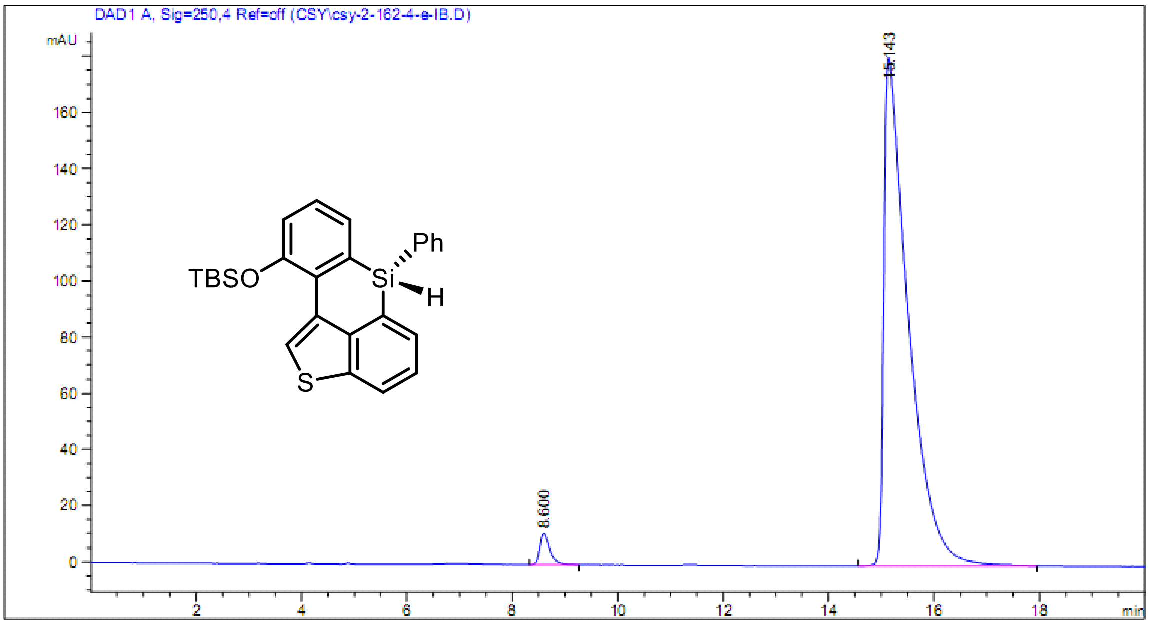

Peak #	RetTime [min]	Type	Width [min]	Area [mAU*s]	Height [mAU]	Area %
1	8.600	BB	0.2040	149.46031	11.03790	2.4953
2	15.143	BB	0.4549	5840.26318	180.78598	97.5047

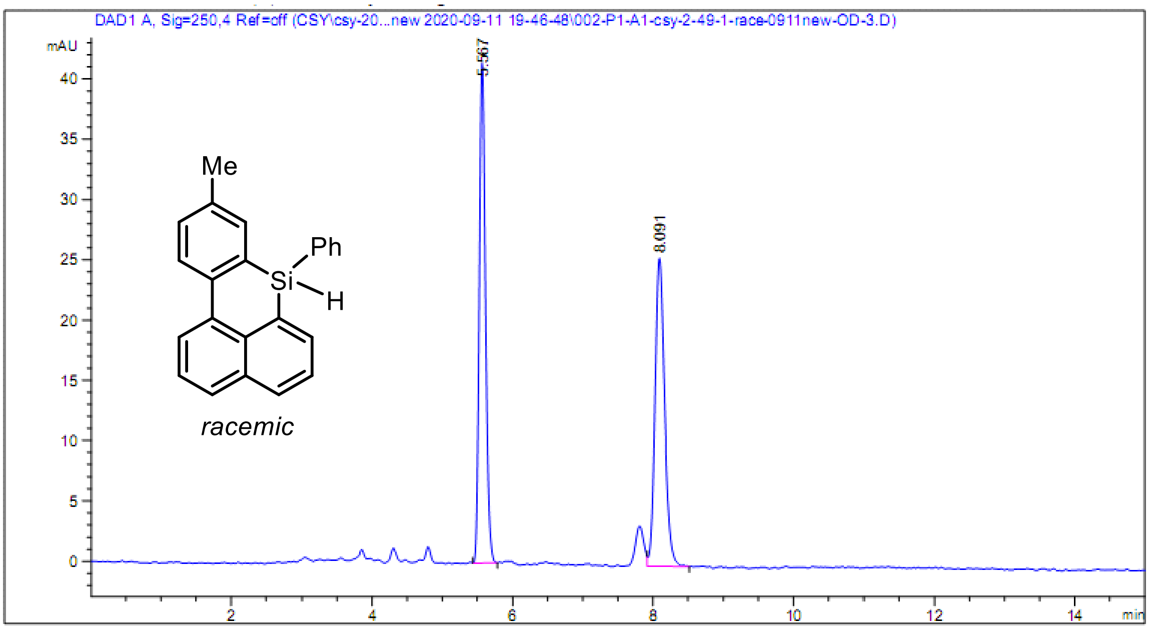

Peak #	RetTime [min]	Type	Width [min]	Area [mAU*s]	Height [mAU]	Area %
1	5.567	BB	0.0919	248.60840	41.50169	50.7409
2	8.091	FM R	0.1571	241.34807	25.60515	49.2591

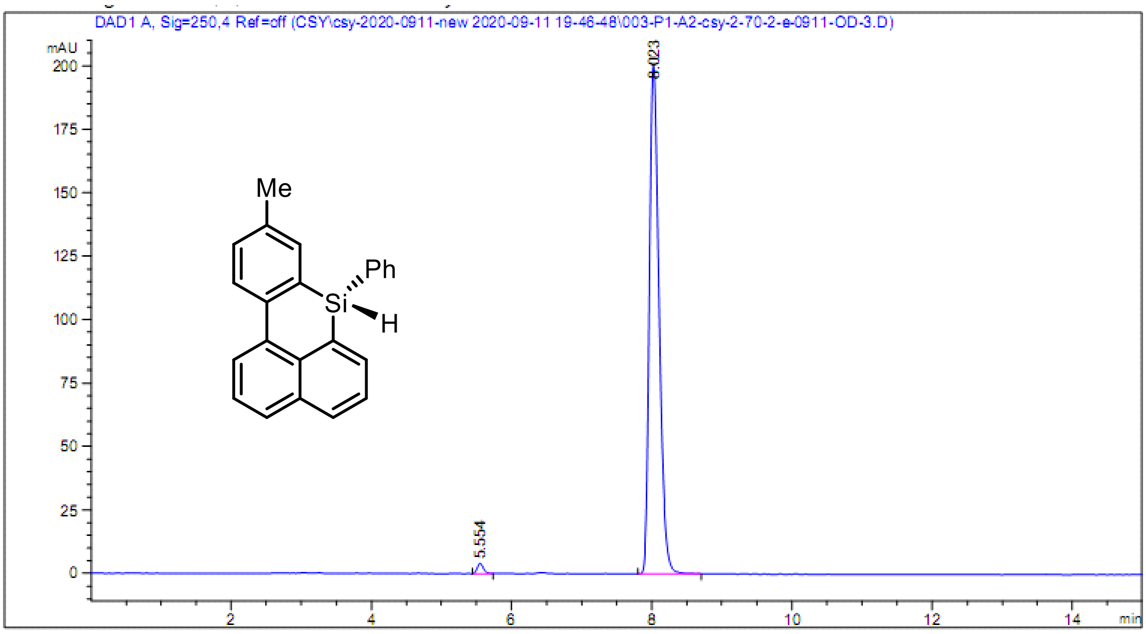

Peak #	RetTime [min]	Type	Width [min]	Area [mAU*s]	Height [mAU]	Area %
1	5.554	BB	0.0931	24.95003	4.09229	1.2962
2	8.023	BB	0.1464	1899.90369	200.72807	98.7038

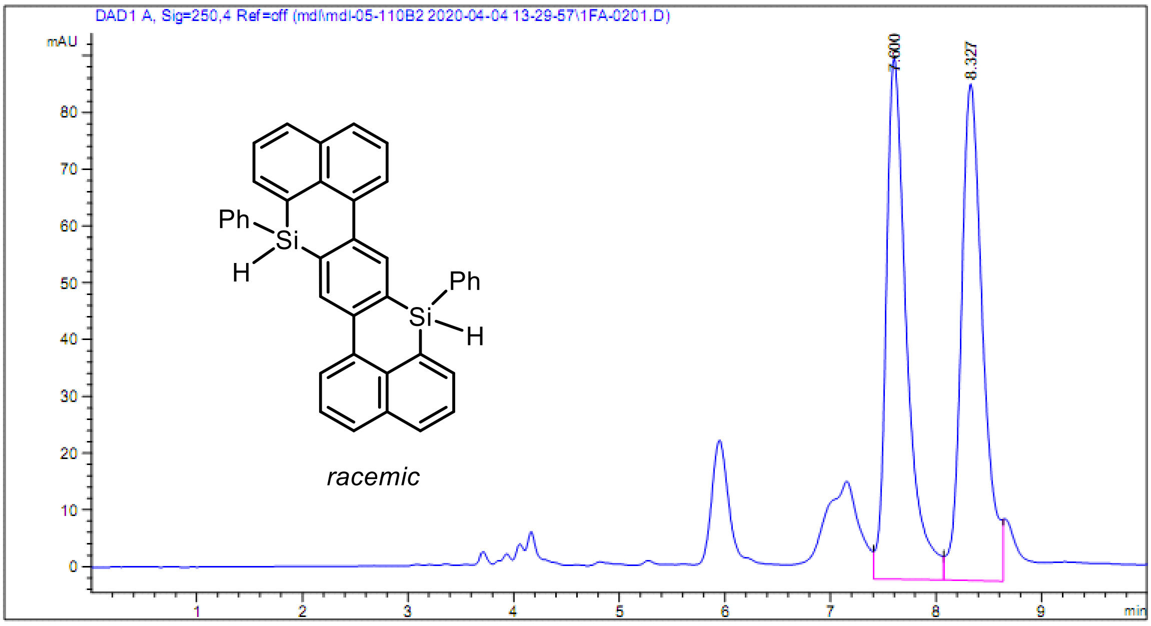

Peak #	RetTime [min]	Type	Width [min]	Area [mAU*s]	Height [mAU]	Area %
1	7.600	MF R	0.2258	1243.04468	91.74100	49.8210
2	8.327	FM R	0.2385	1251.97571	87.49155	50.1790

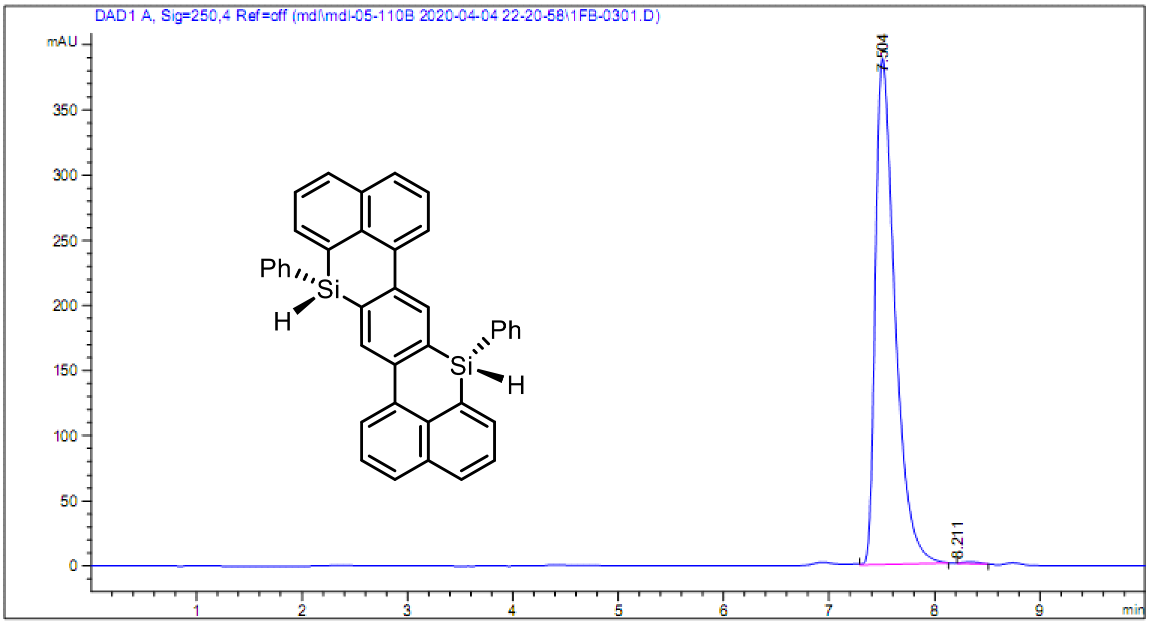

Peak #	RetTime [min]	Type	Width [min]	Area [mAU*s]	Height [mAU]	Area %
1	7.504	MM R	0.2155	5024.80908	388.55637	99.6710
2	8.211	MM R	0.1878	16.58517	3.91149e-1	0.3290

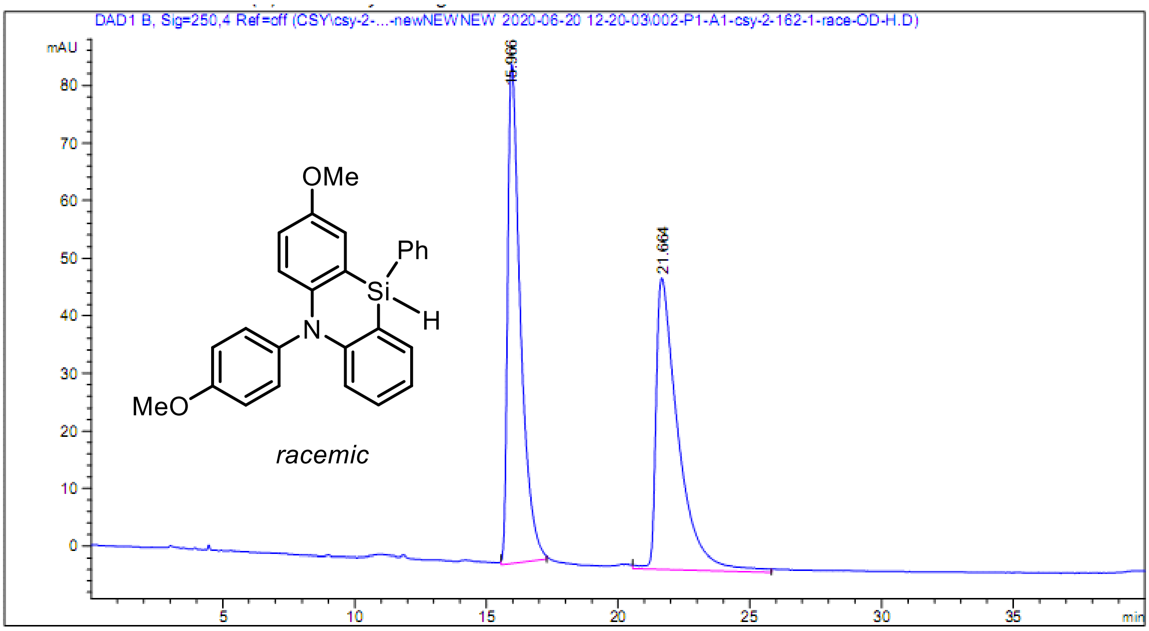

Peak #	RetTime [min]	Type	Width [min]	Area [mAU*s]	Height [mAU]	Area %
1	15.966	MM R	0.5644	2934.51294	86.65720	49.9035
2	21.664	MM R	0.9705	2945.85693	50.59251	50.0965

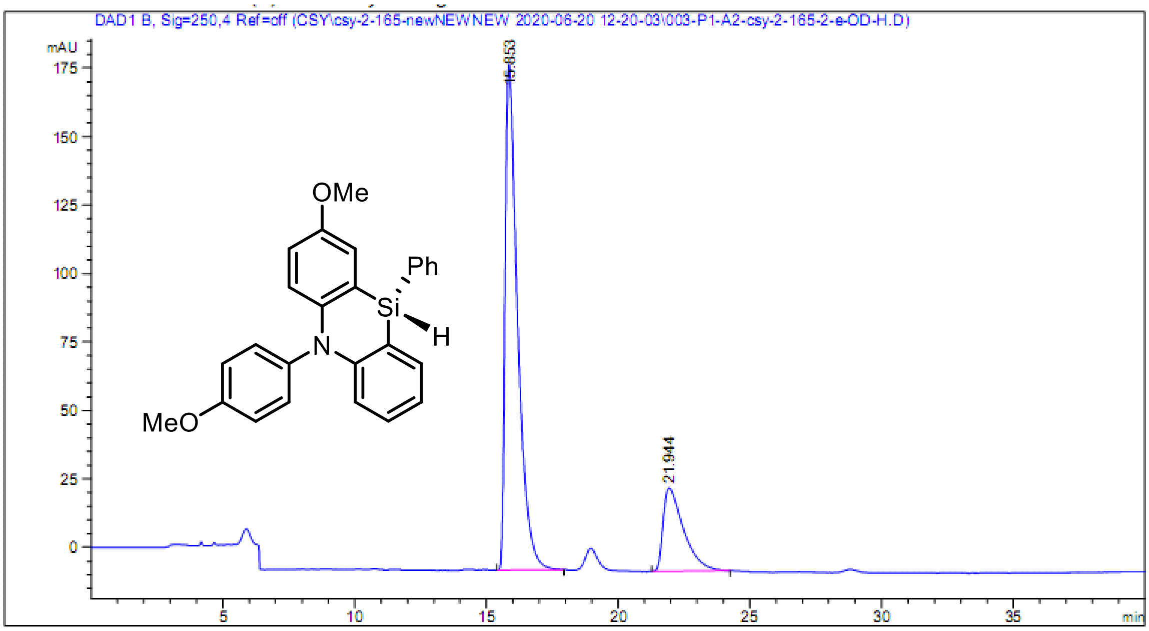

Peak #	RetTime [min]	Type	Width [min]	Area [mAU*s]	Height [mAU]	Area %
1	15.853	BB	0.4901	6080.81055	184.71217	78.7271
2	21.944	BB	0.7533	1643.10413	30.36423	21.2729

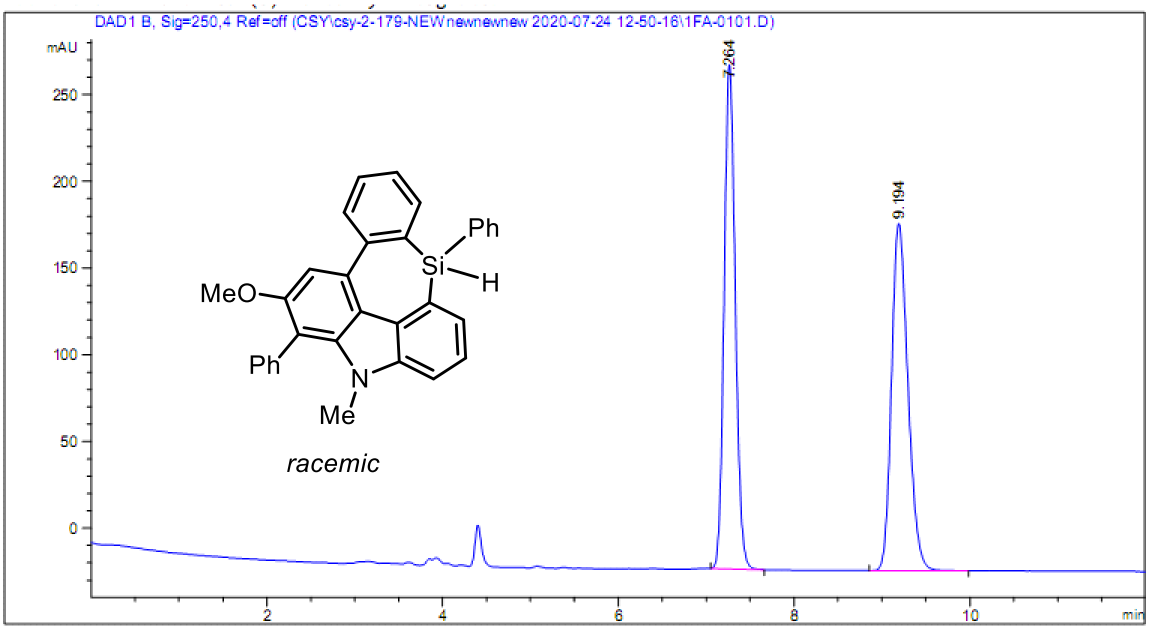

Peak #	RetTime [min]	Type	Width [min]	Area [mAU*s]	Height [mAU]	Area %
1	7.264	BB	0.1373	2576.33716	291.03967	49.7903
2	9.194	BB	0.1994	2598.04272	200.14909	50.2097

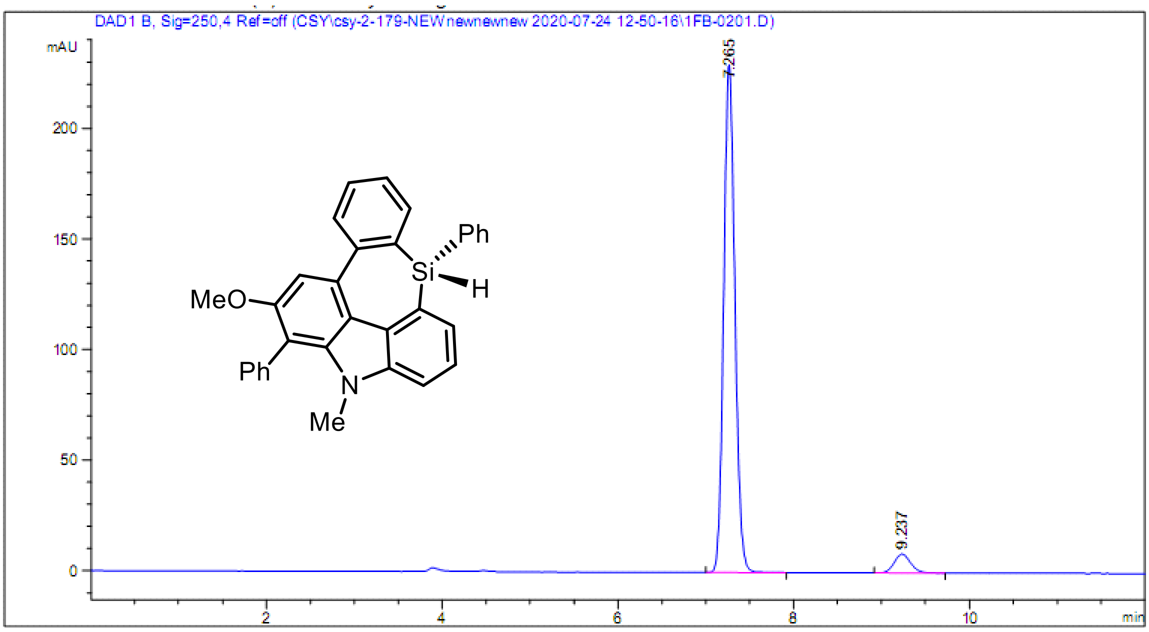

Peak #	RetTime [min]	Type	Width [min]	Area [mAU*s]	Height [mAU]	Area %
1	7.265	BB	0.1375	2039.20862	229.82327	94.7597
2	9.237	BB	0.2032	112.77008	8.58966	5.2403

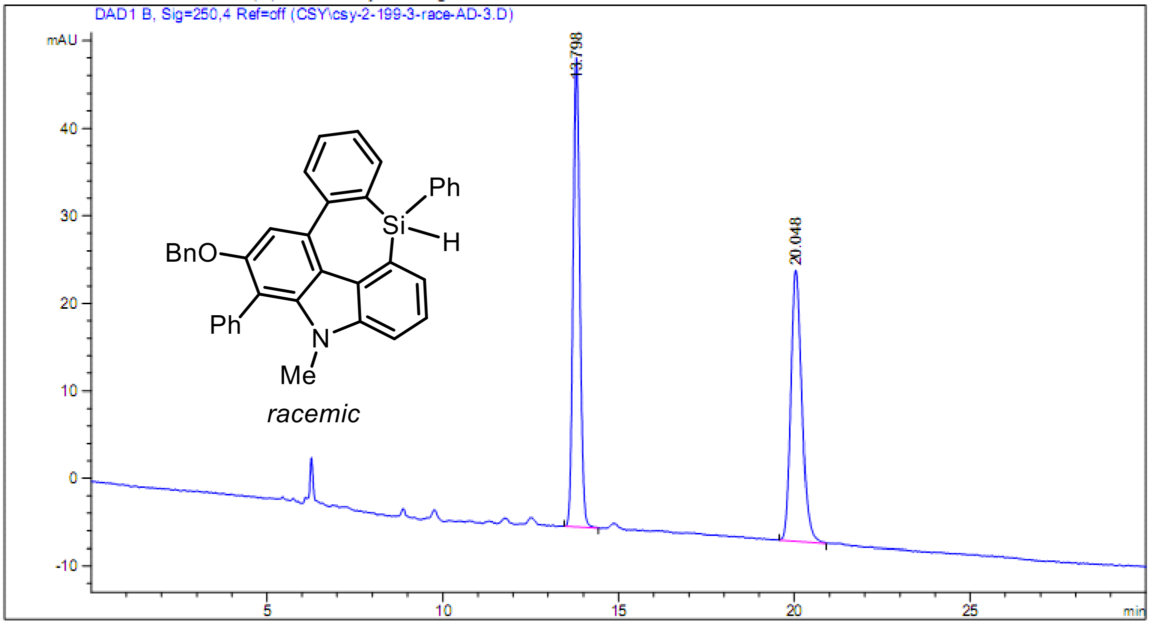

Peak #	RetTime [min]	Type	Width [min]	Area [mAU*s]	Height [mAU]	Area %
1	13.798	BB	0.1960	679.92151	53.59085	50.3228
2	20.048	BB	0.3304	671.19983	31.02085	49.6772

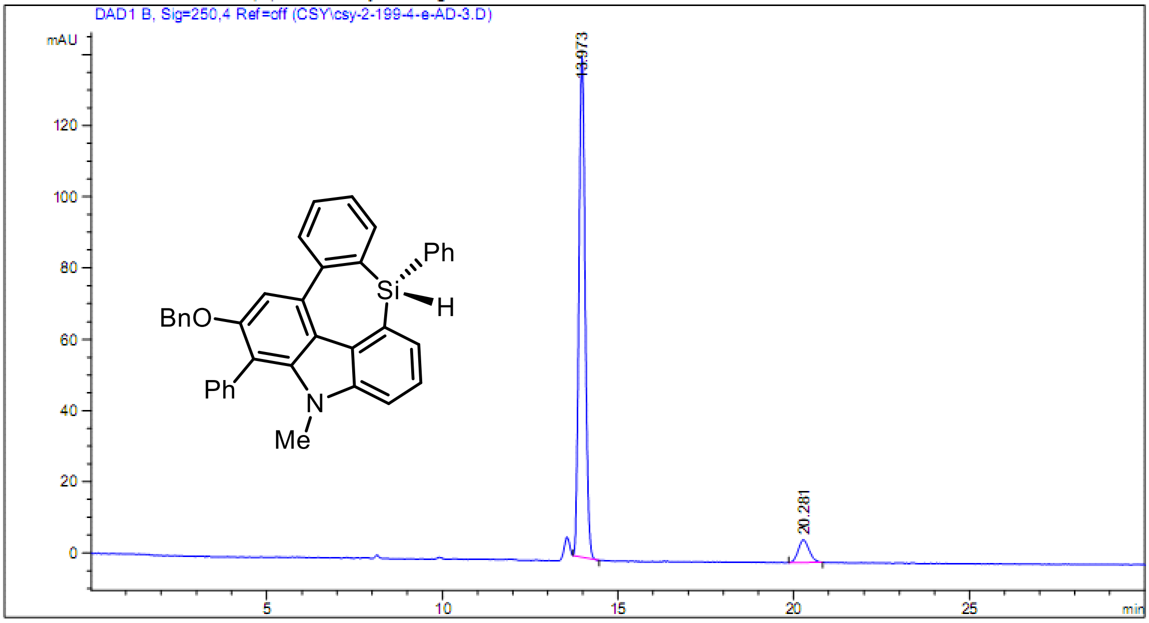

Peak #	RetTime [min]	Type	Width [min]	Area [mAU*s]	Height [mAU]	Area %
1	13.973	MM R	0.2059	1733.51147	140.33629	92.6183
2	20.281	BB	0.3001	138.16063	6.39350	7.3817

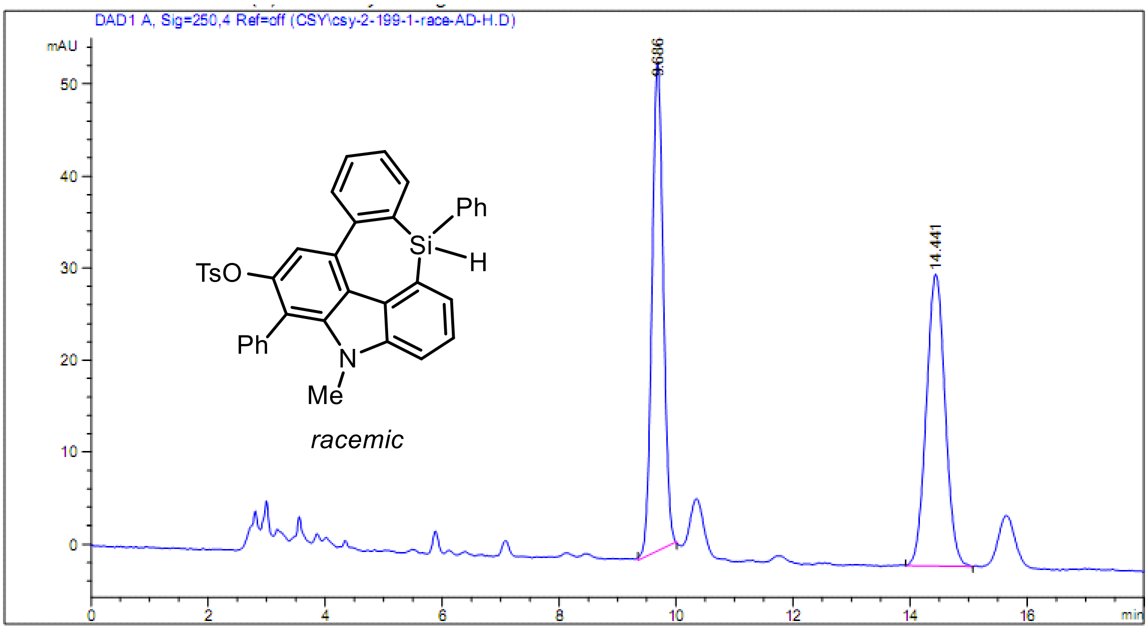

Peak #	RetTime [min]	Type	Width [min]	Area [mAU*s]	Height [mAU]	Area %
1	9.686	MM R	0.2233	710.06366	52.99446	50.0290
2	14.441	BB	0.3452	709.23907	31.68392	49.9710

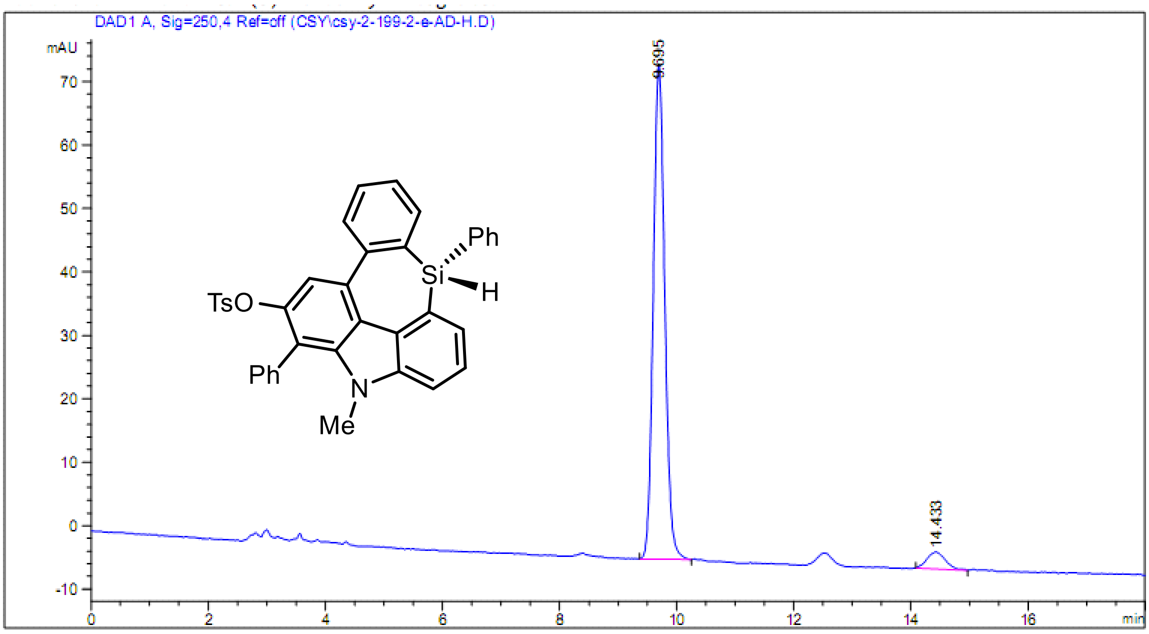

Peak #	RetTime [min]	Type	Width [min]	Area [mAU*s]	Height [mAU]	Area %
1	9.695	BB	0.2108	1059.71570	77.84142	94.8723
2	14.433	BB	0.2703	57.27629	2.66976	5.1277

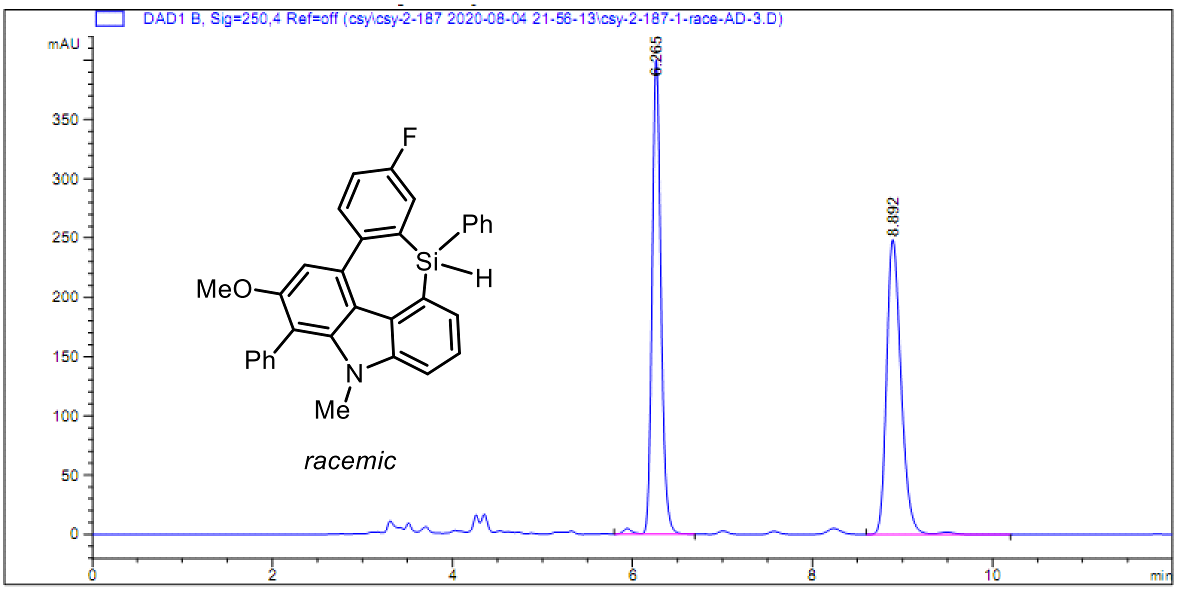

Peak #	RetTime [min]	Type	Width [min]	Area [mAU*s]	Height [mAU]	Area %
1	6.265	VB R	0.1031	2755.05566	400.98779	49.9272
2	8.892	BV R	0.1705	2763.09033	248.61623	50.0728

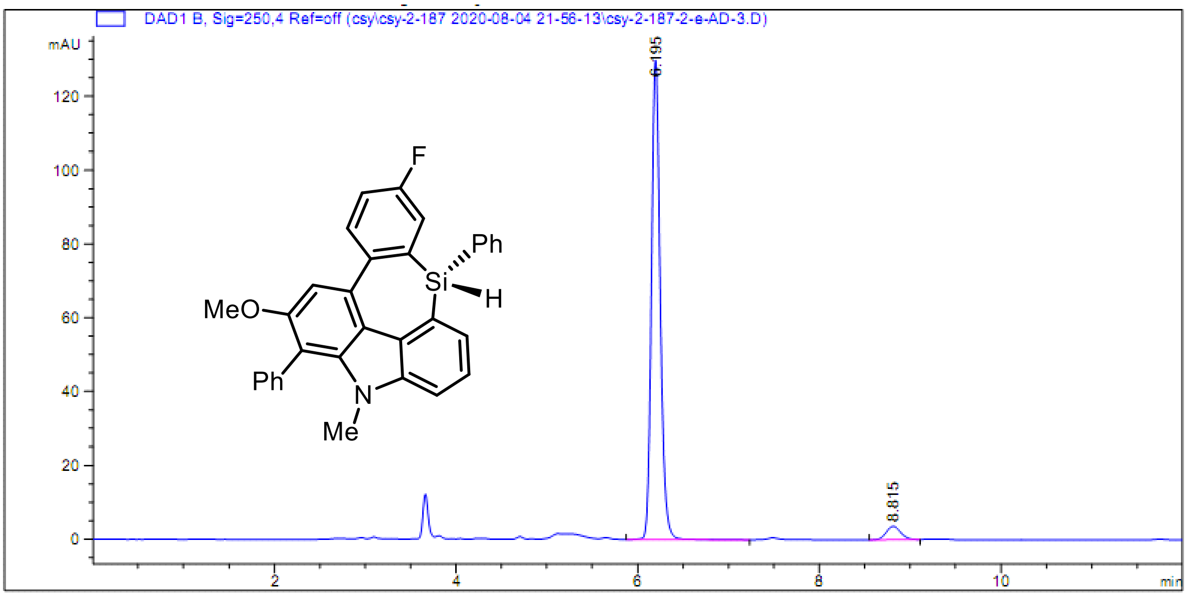

Peak #	RetTime [min]	Type	Width [min]	Area [mAU*s]	Height [mAU]	Area %
1	6.195	BB	0.1024	875.33569	130.20793	95.6773
2	8.815	BB	0.1695	39.54797	3.61764	4.3227

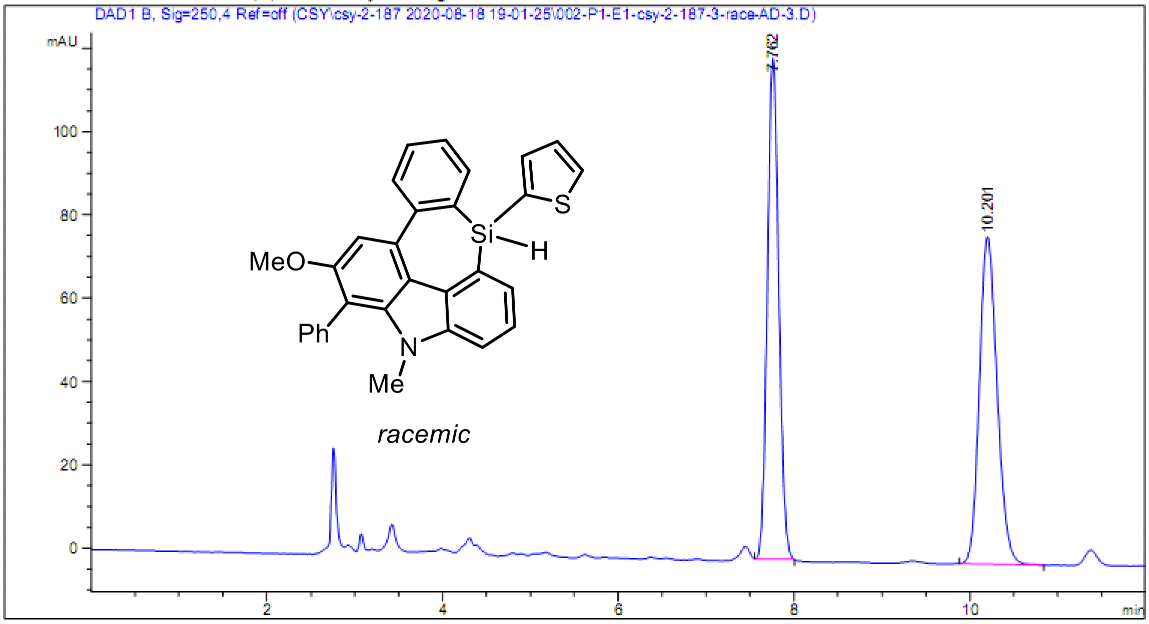

Peak #	RetTime [min]	Type	Width [min]	Area [mAU*s]	Height [mAU]	Area %
1	7.762	MM R	0.1503	1084.68604	120.26268	49.6360
2	10.201	BB	0.2175	1100.59607	78.53077	50.3640

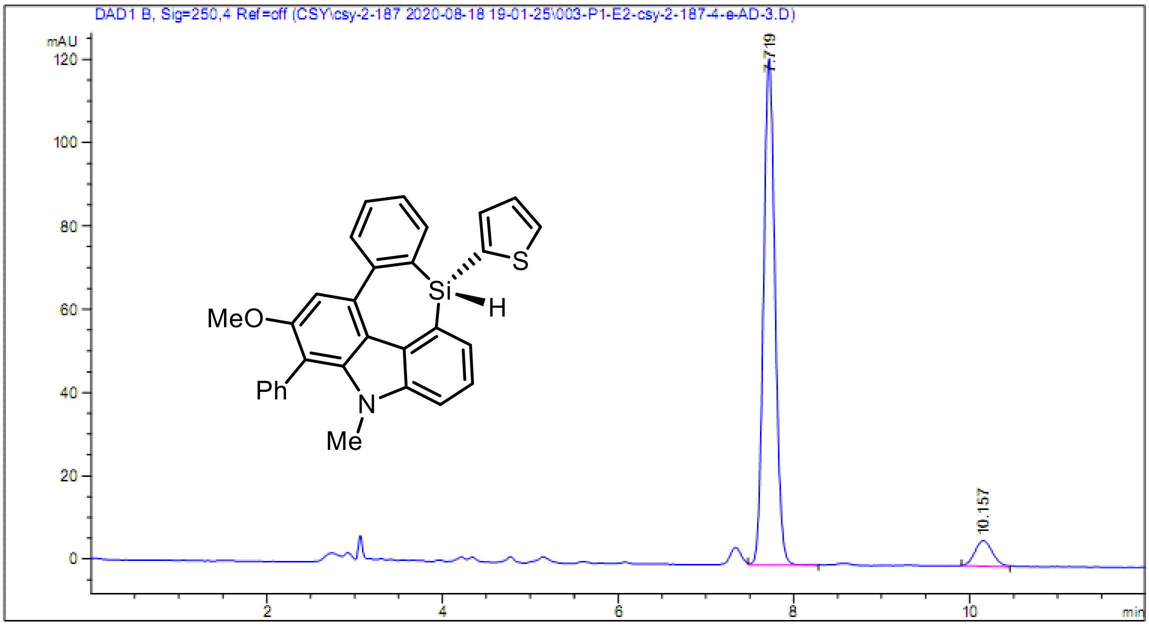

Peak #	RetTime [min]	Type	Width [min]	Area [mAU*s]	Height [mAU]	Area %
1	7.719	MM R	0.1518	1110.15161	121.87543	92.7628
2	10.157	MM R	0.2343	86.61270	6.16140	7.2372

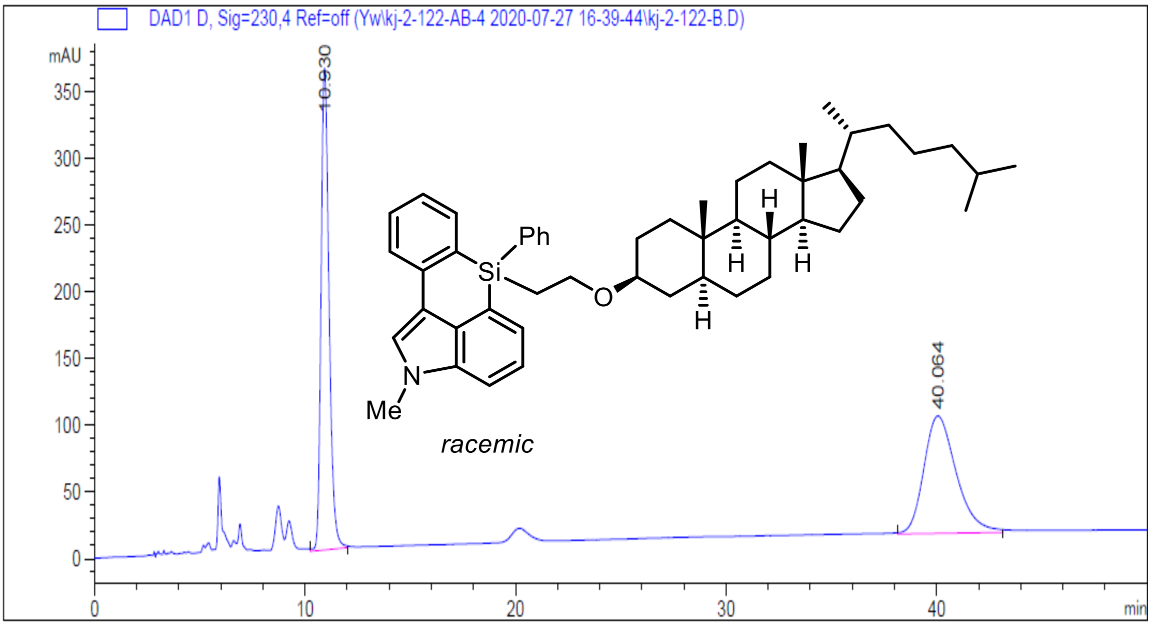

Peak #	RetTime [min]	Type	Width [min]	Area [mAU*s]	Height [mAU]	Area %
1	10.930	MM R	0.4386	9499.81543	361.00558	50.1861
2	40.064	MM R	1.7867	9429.35449	87.95641	49.8139

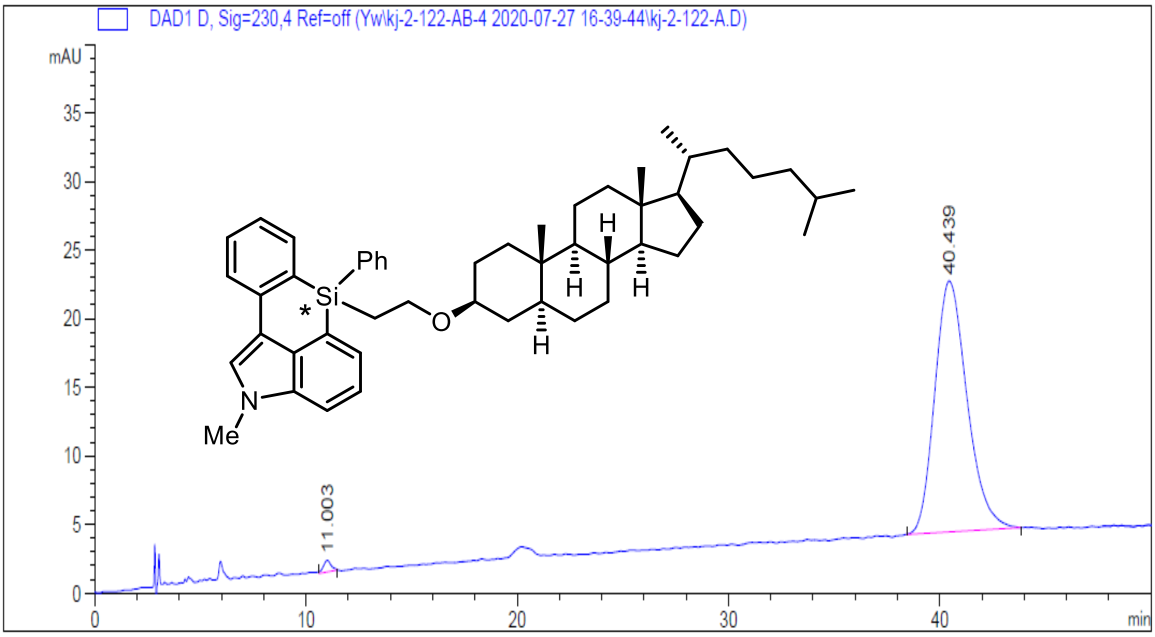

Peak #	RetTime [min]	Type	Width [min]	Area [mAU*s]	Height [mAU]	Area %
1	11.003	MM R	0.4205	21.30786	8.44549e-1	1.0829
2	40.439	BB	1.4579	1946.41211	18.28441	98.9171

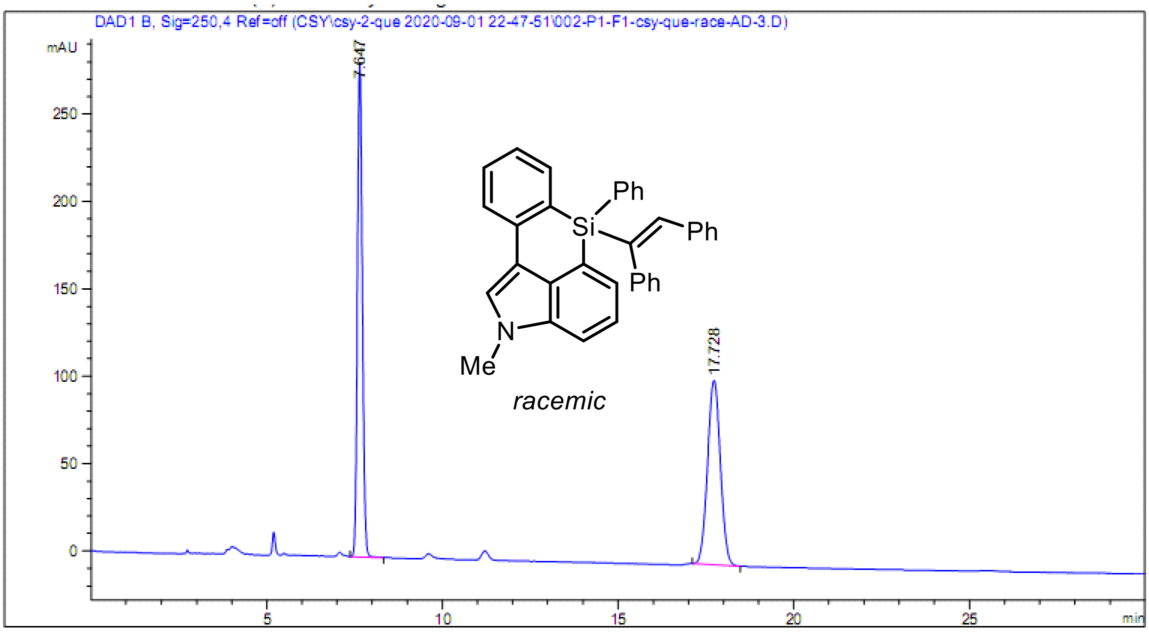

Peak #	RetTime [min]	Type	Width [min]	Area [mAU*s]	Height [mAU]	Area %
1	7.647	BB	0.1497	2750.28198	282.18976	50.5670
2	17.728	BB	0.3964	2688.60645	105.81007	49.4330

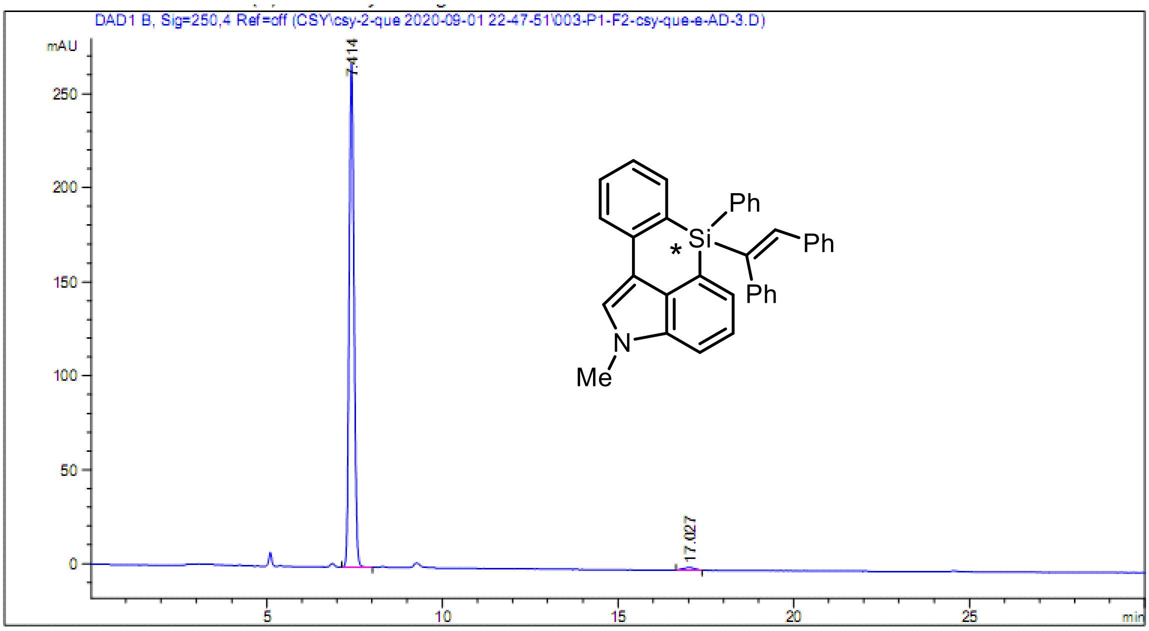

Peak #	RetTime [min]	Type	Width [min]	Area [mAU*s]	Height [mAU]	Area %
1	7.414	BB	0.1487	2537.22363	267.49890	98.8358
2	17.027	MM R	0.3863	29.88544	1.28938	1.1642

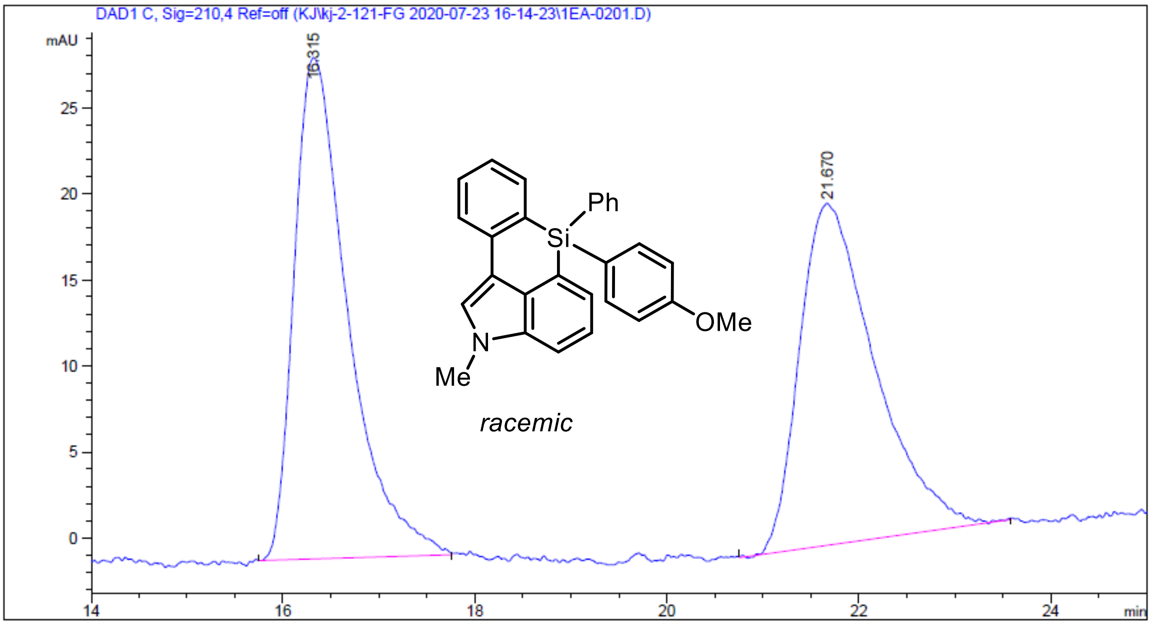

Peak #	RetTime [min]	Type	Width [min]	Area [mAU*s]	Height [mAU]	Area %
1	16.315	MM R	0.6477	1132.22729	29.13316	51.0594
2	21.670	MM R	0.9092	1085.24304	19.89431	48.9406

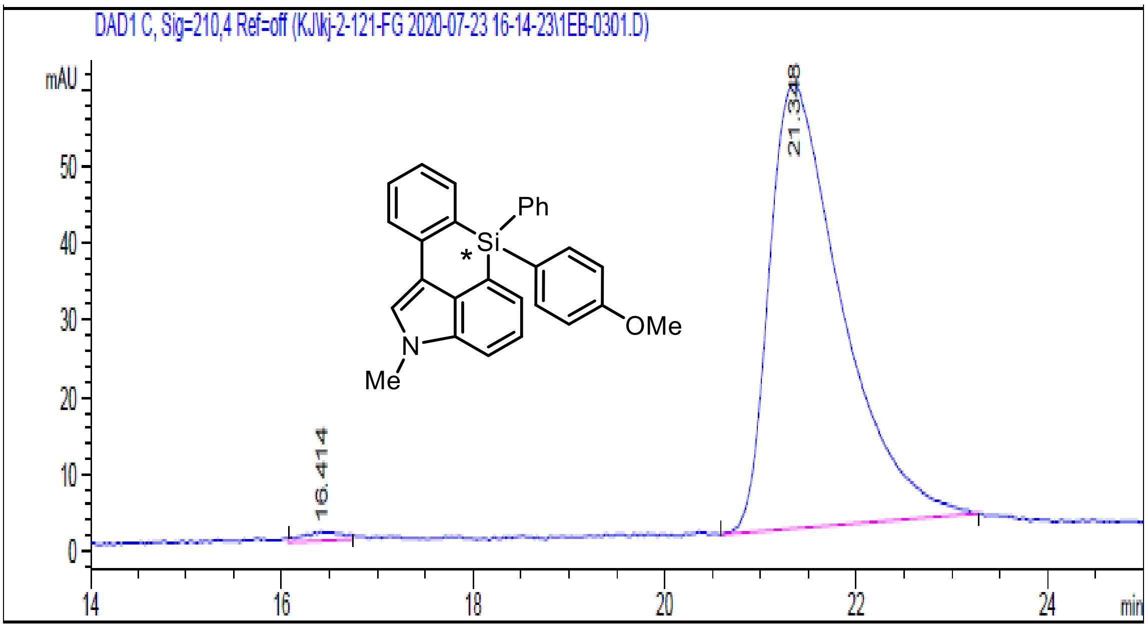

Peak #	RetTime [min]	Type	Width [min]	Area [mAU*s]	Height [mAU]	Area %
1	16.414	MM R	0.4816	33.65550	1.16463	1.0953
2	21.348	BB	0.7124	3039.07104	57.70648	98.9047

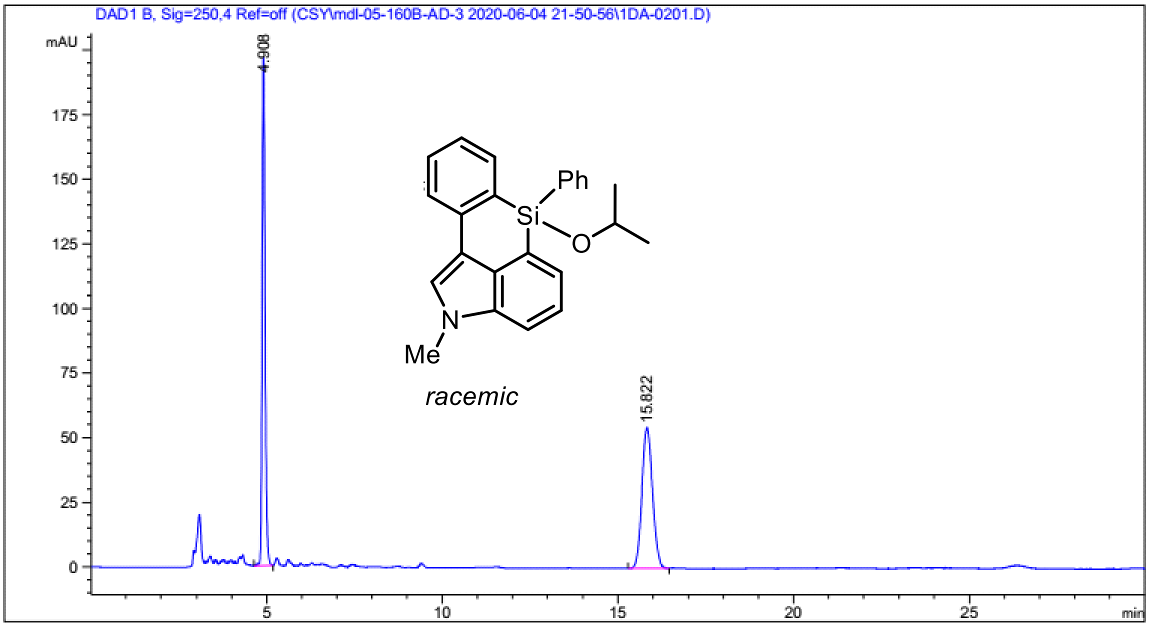

Peak #	RetTime [min]	Type	Width [min]	Area [mAU*s]	Height [mAU]	Area %
1	4.908	VB R	0.0898	1143.61926	196.71693	50.1565
2	15.822	BB	0.3283	1136.48157	54.27884	49.8435

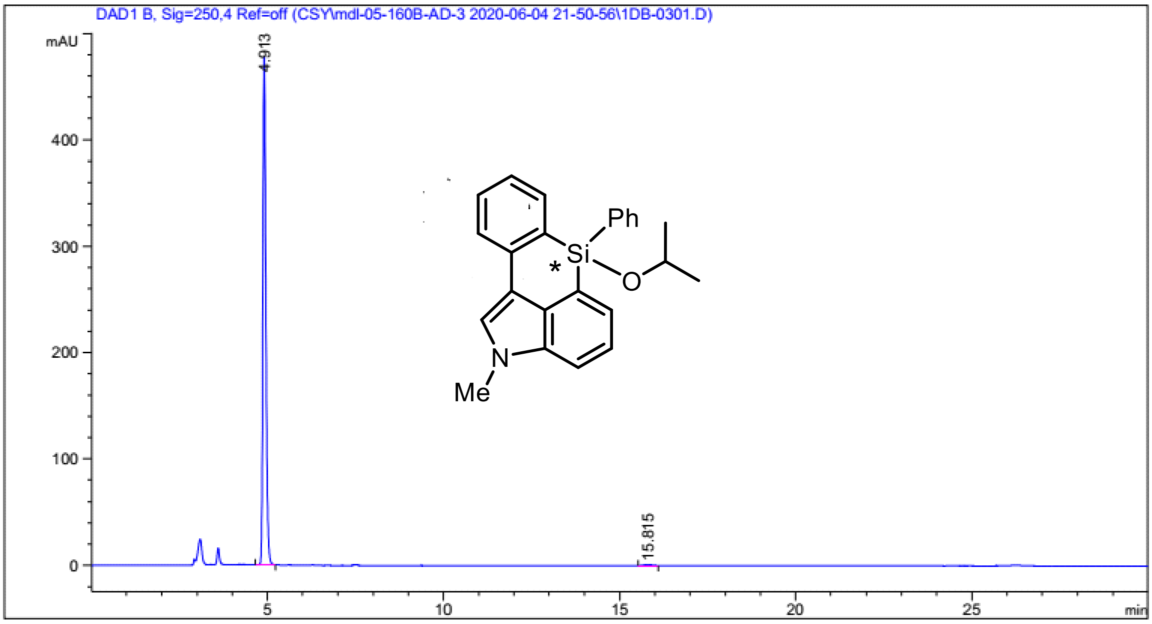

Peak #	RetTime [min]	Type	Width [min]	Area [mAU*s]	Height [mAU]	Area %
1	4.913	BB	0.0890	2830.44531	478.24933	99.0423
2	15.815	MM R	0.3889	27.36983	1.17311	0.9577

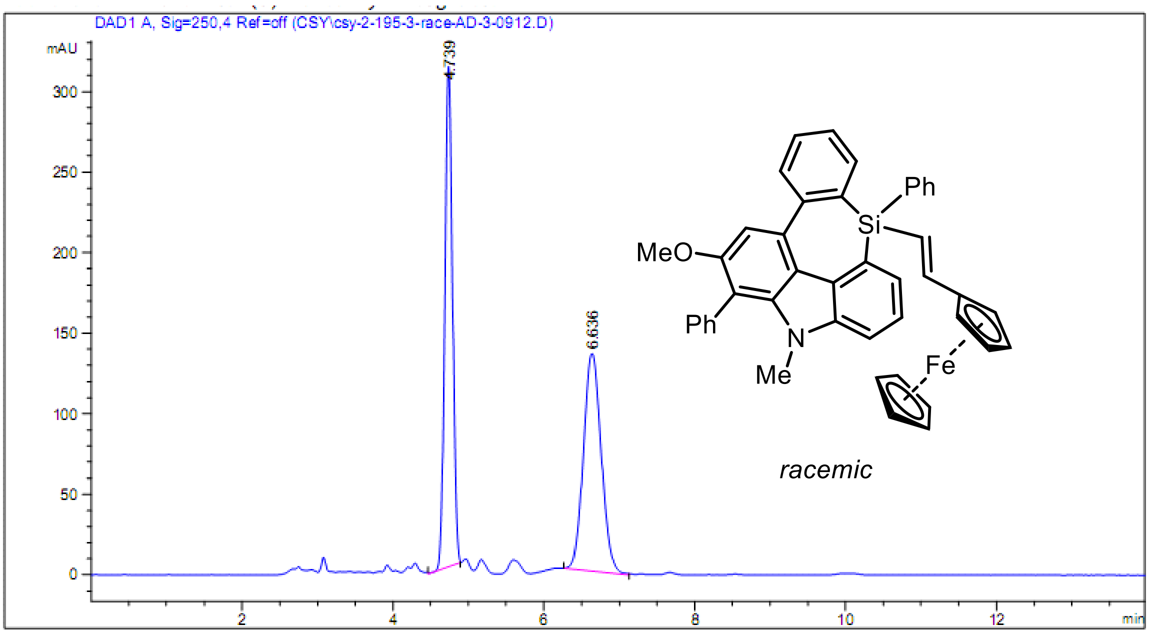

Peak #	RetTime [min]	Type	Width [min]	Area [mAU*s]	Height [mAU]	Area %
1	4.739	MM R	0.1182	2208.51758	311.33322	49.9800
2	6.636	MM R	0.2730	2210.28101	134.95793	50.0200

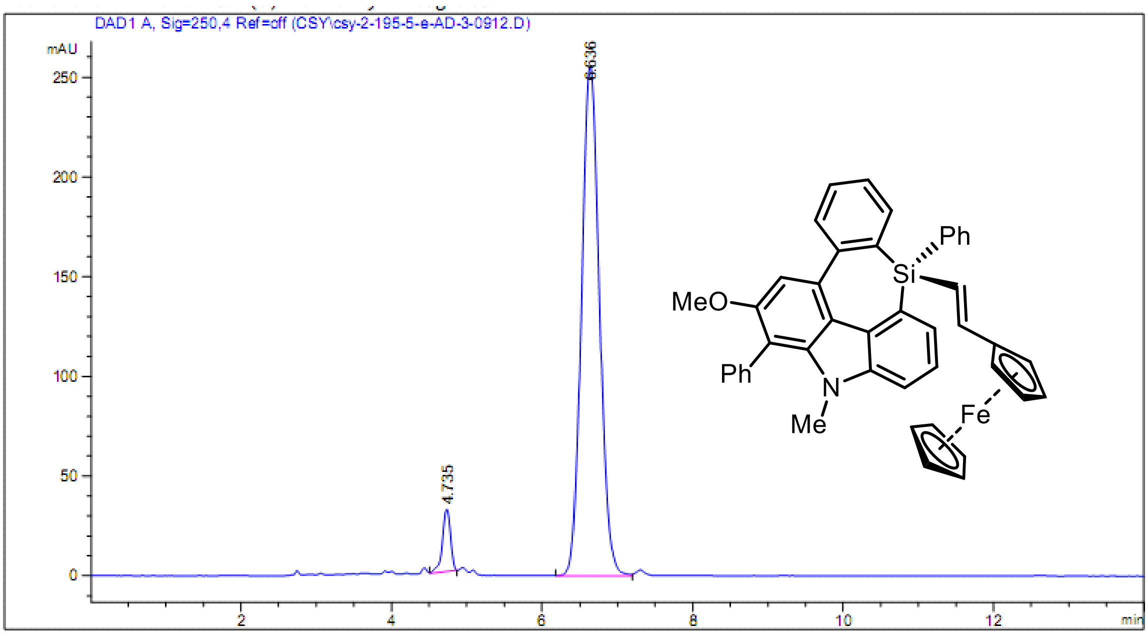

Peak #	RetTime [min]	Type	Width [min]	Area [mAU*s]	Height [mAU]	Area %
1	4.735	MM R	0.1234	233.05380	31.46772	5.2382
2	6.636	MM R	0.2751	4216.07861	255.40259	94.7618

XI. Supplementary References

1. Visco, M. D., Wieting, J. M. & Mattson, A. E. Carbon−silicon bond formation in the synthesis of benzylic silanes. *Org. Lett.* **18**, 2883-2885 (2016).

2. Yasutomi, Y., Suematsu, H. & Katsuki, T. Iridium(III)-catalyzed enantioselective Si−H bond insertion and formation of an enantioenriched silicon center. *J. Am. Chem. Soc.* **132**, 4510-4511 (2010).

3. Xu, D. *et al.* Enantiodivergent formation of C−P bonds: synthesis of P-chiral phosphines and methylphosphonate oligonucleotides. *J. Am. Chem. Soc.* **142**, 5785-5792 (2020).

4. Perato, S., Large, B., Lu, Q., Gaucher, A. & Prim, D. Pyridylmethylamine-palladium catalytic systems: a selective alternative in the C−H arylation of indole. *ChemCatChem*. **9**, 389-392 (2017).

5. Davies, W. & Middleton, S. Benxofuran derivatives formed by cyclisation of α-aryloxyacetophenones. *J. Chem. Soc.* 822-825 (1958).

6. Shrives, H. J., Fernandez-Salas, J. A., Hedtke, C., Pulis, A. P. & Procter, D. J. Regioselective synthesis of C3 alkylated and arylated benzothiophenes. *Nat. Commun*. **8**, 14801-14807 (2017).

7. Ducos, P., Liautard, V., Robert, F. & Landais, Y. Chiral memory in silylium ions. *Chem. Eur. J.* **21**, 11573-11578 (2015).

8. Qiao, Y. *et al.* Copper-catalyzed successive C−C bond formations on indoles or pyrrole: a convergent synthesis of symmetric and unsymmetric hydroxyl substituted N−H carbazoles. *Adv. Synth. Catal*. **360**, 2138-2143 (2018).

9. Ma, W., Liu, L. C., An, K., He, T. & He, W. Rh-catalyzed syntheses of chiral monohydrosilanes via intramolecular C−H functionalization of dihydrosilanes. *Angew. Chem., Int. Ed.* doi:10.1002/anie.202013041 (2020).

10. Dominguez, Z. *et al.* Azabora[5]helicene charge-transfer dyes show efficient and spectrally variable circularly polarized luminescence. *Chem. Eur. J.* **24**, 12660-12668 (2018).

11. Ito, M., Kawasaki, R., Kanyiva, K. S. & Shibata, T. Construction of a polycyclic conjugated system containing a dibenzazepine moiety by cationic gold(I)-catalyzed cycloisomerization. *Eur. J. Org. Chem*. 5234-5237 (2016).

12. Igawa, K., Yoshihiro, D., Ichikawa, N., Kokan, N. & Tomooka, K. Catalytic enantioselective synthesis of alkenylhydrosilanes. *Angew. Chem., Int. Ed.* **51**, 12745-12748 (2012).
